# *gem*-Difluorovinyl and trifluorovinyl Michael acceptors in the synthesis of α,β-unsaturated fluorinated and nonfluorinated amides

**DOI:** 10.3762/bjoc.20.247

**Published:** 2024-11-15

**Authors:** Monika Bilska-Markowska, Marcin Kaźmierczak, Wojciech Jankowski, Marcin Hoffmann

**Affiliations:** 1 Faculty of Chemistry, Adam Mickiewicz University in Poznań, Uniwersytetu Poznańskiego 8, 61-614 Poznań, Poland; 2 Center for Advanced Technologies, Adam Mickiewicz University in Poznań, Uniwersytetu Poznańskiego 10, 61-614 Poznań, Poland

**Keywords:** *gem*-difluorovinyl Michael acceptors, Michael addition, trifluorovinyl Michael acceptors, α,β-unsaturated amides

## Abstract

The incorporation of fluorine atoms within the structure of organic compounds is known to exert a significant impact on their electronic properties, thereby modulating their reactivity in diverse chemical transformations. In the context of our investigation, we observed a striking illustration of this phenomenon. A Michael addition involving *gem*-difluorovinyl and trifluorovinyl acceptors was successfully achieved, demonstrating high stereoselectivity. This selectivity was further elucidated through theoretical calculations. Using this methodology, a series of new α,β-unsaturated amides, both fluorinated and nonfluorinated, were synthesized.

## Introduction

The Michael reaction, characterized by the addition of stable carbon nucleophiles to unsaturated compounds with electron-withdrawing groups, is a cornerstone in constructing carbon–carbon and carbon–heteroatom bonds [1]. It is instrumental in synthesizing natural products [2–5] and pharmaceuticals [6], underlining its significance in organic chemistry. Recent advancements have broadened the scope of Michael donors and acceptors to encompass fluorine-containing compounds, enhancing the reaction's utility in synthesizing fluorinated derivatives [7–8]. Shibata and colleagues pioneered the use of fluorinated Michael donors, notably achieving enantioselective addition of 1-fluorobis(phenylsulfonyl)methane (FBSM) to α,β*-*unsaturated ketones with cinchona alkaloids [9]. Fluorinated Michael acceptors usually contain one fluorine atom or a trifluoromethyl group in the structure [10–12]. There are also known examples of *gem*-difluoroalkenes being used as Michael acceptors [13–17]. The Michael addition with fluorinated acceptors finds application in the synthesis of, among others, fluorinated amino acids, which can be a structural motif in many biologically active compounds [18]. There are also known studies on the incorporation of highly reactive fluorinated Michael acceptors into peptide structures, which can act as the link between an active molecule and its cellular target [19–20]. Such endeavors hint at the potential of fluorinated acceptors in designing fluorinated peptidomimetics, an area attracting global research interest [21–24].

In our laboratory, we have explored the synthesis of 3,3,3-trifluoro- and 2,3,3,3-tetrafluoro-*N*-substituted propanamides, contributing to the field of fluorinated amides [25]. We have also investigated deprotonation at the α position of other fluorinated carbonyl derivatives as a route to new building blocks [26]. Despite the known instability of trifluoromethylated carbanions [27], their catalytic application has yielded valuable products [28–30]. *gem*-Difluoroalkenes and trifluoroalkenes are excellent acceptors in the Michael addition reactions. There are also known examples of the use of *gem*-difluoroalkenes and trifluoroalkenes in reactions with Grignard reagents [13,31]. Although, similar compounds are reported to be unstable molecules that are prone to decomposition under reaction conditions [32–33].

**Scheme 1:**
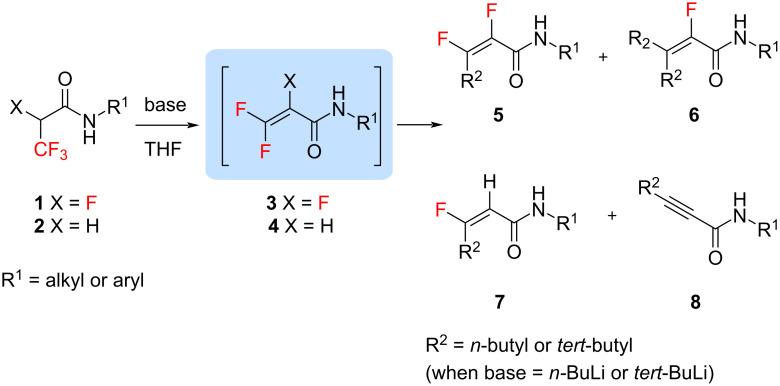
Generation of *gem*-difluorovinyl and trifluorovinyl Michael acceptors and their use in the synthesis of α,β*-*unsaturated fluoroamides.

The goal of this work was the formation of *gem*-difluoro- and trifluorovinyl Michael acceptors by using organolithium reagents (Scheme 1), revealing new avenues in fluorinated unsaturated amide synthesis, which are present in numerous natural products, pharmaceuticals, and polymers [34–38]. The obtained α,β-unsaturated amides may represent promising structural motifs for further synthesis, e.g., via pericyclic reactions or nucleophilic additions.

## Results and Discussion

We commenced our research by screening the nature of the base to generate carbanion at *alpha* position. We chose 2,3,3,3-tetrafluoro-*N*-heptylpropanamide, obtained according to the procedure developed earlier in our laboratory [25], for our model reaction. The reactions were carried out under inert gas conditions in anhydrous solvents (THF or DCM) at −78 °C for 3 h, using several bases and electrophiles (Table 1). The use of electrophiles in the first test reactions was to confirm the generation of a carbanion, which was to be evidenced by a substitution reaction at the *alpha* position. We started testing the different bases with lithium bis(trimethylsilyl)amide [39]. The reactions did not take place in the presence of LiHMDS (Table 1, entries 1 and 2), using either benzyl bromide or methyl iodide as electrophiles. Next, TiCl_4_ as metal enolate mediator was applied. In the presence of both, Et_3_N as well as *N,N,N′,N′-*tetramethyl-1,3-propanediamine no reaction was observed (Table 1, entries 3–6) [40]. With titanium chloride and *n*-BuLi, low conversion of the starting material and obtained product *Z*-**9a** was characteristic (Table 1, entry 7). A slightly higher reactivity was achieved when the BF_3_·(OEt_2_) was used instead of TiCl_4_ (Table 1, entry 8) [28]. The reactions were monitored by ^19^F NMR of the crude mixtures. The full conversion was reached by applying exclusively *n*-BuLi, but the formed product was not the anticipated α-substituted compound (Table 1, entry 9). The NMR analysis revealed that the obtained compounds were Michael addition products. The formation of the presented compounds (Table 1) was due to the earlier generation of *gem*-difluoroalkenes by the elimination of one of the fluorine atoms from the CF_3_ group, proving that both *gem*-difluoroalkenes and the double bond of product *Z-***9a** were excellent Michael acceptors. This confirmed that electrophiles were not involved in the reaction. We therefore focused only on using *n*-BuLi, which, as it turned out, acted as both the base and Michael's donor (Table 1, entry 10).

**Table 1:** Optimization of reaction conditions.

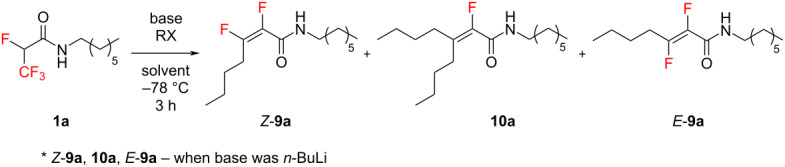				

Entry	Base/Lewis acid, base	Electrophile RX, RCHO	Solvent	Result

1	LiHMDS, 2 equiv	BnBr, 2 equiv	THF	n. r.^a^
2	LiHMDS, 2 equiv	MeI, 2 equiv	THF	n. r.
3	Et_3_N, 3 equiv TiCl_4_, 1.2 equiv	MeI, 2 equiv	DCM	n. r.
4	TMPDA, 1.2 equiv TiCl_4_, 1.2 equiv	MeI, 2 equiv	DCM	n. r.
5	Et_3_N, 3 equiv TiCl_4_, 1.2 equiv	PhCHO, 2 equiv	DCM	n. r.
6	TMPDA, 1.2 equiv TiCl_4_, 1.2 equiv	PhCHO, 2 equiv	DCM	n. r.
7	*n*-BuLi, 4 equiv TiCl_4_, 1.2 equiv	PhCHO, 2 equiv	THF	traces of *Z*-**9a**^b^
8	*n*-BuLi, 4 equiv BF_3_·OEt_2_, 1.2 equiv	PhCHO, 2 equiv	THF	1:2.8:0.3:traces (**1a**/*Z*-**9a/10a/***E-***9a**)^b^
9	*n*-BuLi, 4 equiv	MeI, 2 equiv	THF	0:1:0.15:traces (**1a**/*Z*-**9a**/**10a**/*E*-**9a**)^b^
10	*n*-BuLi, 4 equiv	–	THF	0:1:0.1:0 (**1a**/*Z*-**9a**/**10a**/*E*-**9a**)^b^

^a^No reaction. ^b^Determined by ^19^F NMR spectroscopy of crude reaction mixtures.

**Scheme 2:**
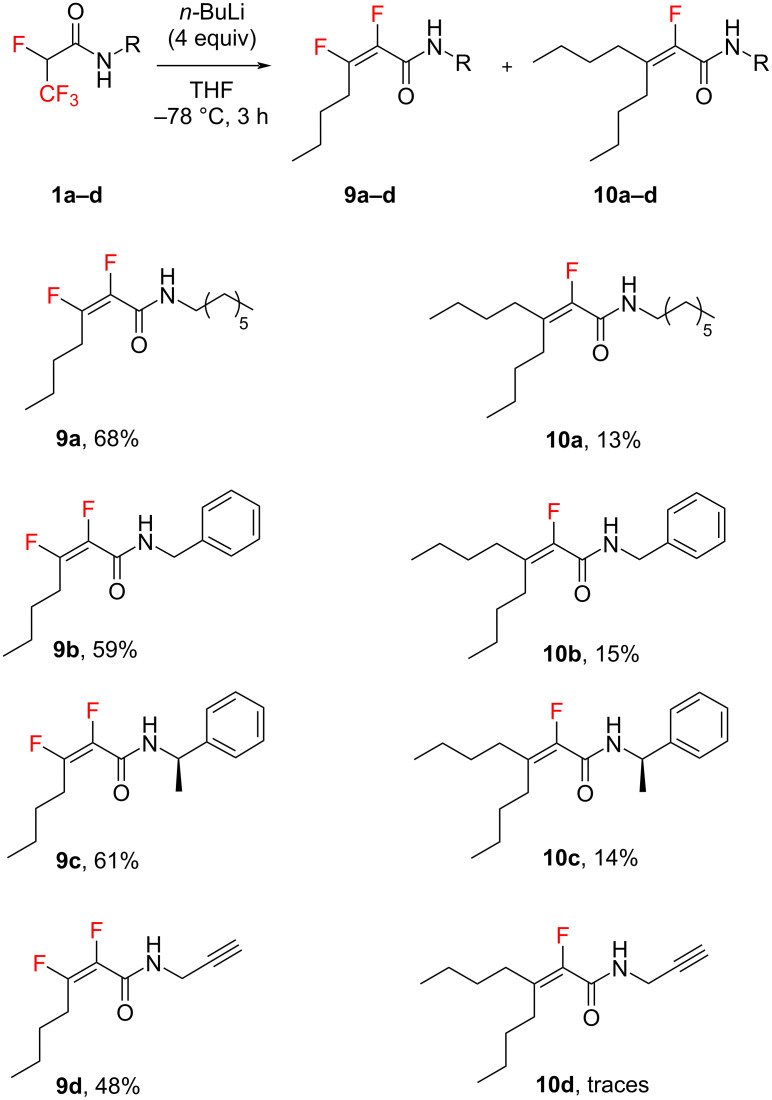
Formation of α,β-difluorinated and α-fluorinated α,β-unsaturated amides.

Having the optimized conditions in hand, we subjected other 2,3,3,3-tetrafluoropropanamides to the same process. Both amides substituted by electron-withdrawing and electron-donating groups proved to be suitable substrates for this reaction, providing the corresponding Michael addition products. These highly stable compounds were isolated after purification on silica gel in good yields (Scheme 2) and characterized by spectroscopic methods. The reaction proceeded with very high *Z*-stereoselectivity (Scheme 2, compounds **9a**–**d**). In the ^19^F NMR spectra of crude mixtures, only trace amounts of *E*-isomer of products **9** were identified. The fluorine atom signals of **9a**–**d** were located at approximately −112 ppm (triplets, *J* ≈ 27 Hz, F_β_) and at −155 to −159 ppm (multiplets, F_α_). The stereochemistry was determined by ^19^F{^1^H} NMR spectroscopy. The observed coupling constants *J* ≈ 2 Hz between vinylic fluorine atoms were typical and confirmed that the *Z* isomers were obtained predominantly [41]. The vicinal coupling constant between the F_β_ and H_γ_ atoms amounted approximately 27 Hz in cases of **9a**–**d**. Based on these findings, we concluded that the dihedral angle between the F_β_ and H_γ_ atoms is approximately 150° [42–45]. These data were consistent with DFT calculations (see Supporting Information File 1). The reaction of the amide **1d** with *n*-BuLi resulted in a surprising outcome. In this case, product **9d** and only traces of the expected product **10d** were received, as indicated by the ^19^F NMR spectrum of the crude reaction mixture. Interestingly, for this reaction we also observed that the *Z*-**9d**/*E*-**9d** products were obtained in a ratio of 1:0.2. However, the main *Z*-isomer was only isolated and fully characterized.

**Scheme 3:**
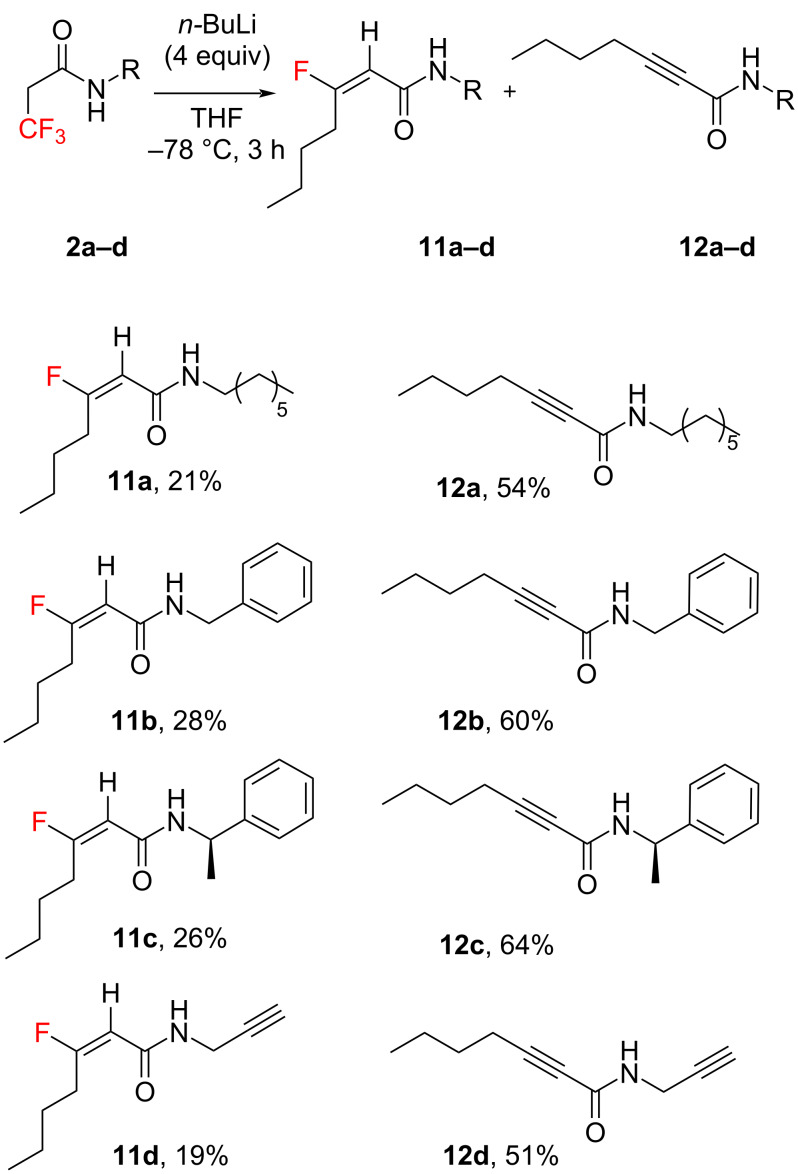
Formation of β-fluorinated and nonfluorinated α,β-unsaturated amides.

In our subsequent investigation of the 3,3,3-trifluoropropanamides substrate **2a**–**d** scope, we observed that *gem*-difluoroalkenes produced β-fluoro-unsaturated amides **11a**–**d** (Scheme 3). In these reactions, we used conditions previously optimised for derivatives **1a**–**d** (*n*-BuLi 4 equiv, THF, −78 °C, 3 h). The amides **11a**–**d** preferred HF elimination over engaging in another Michael reaction, leading to the formation of products **12a**–**d** as illustrated in Scheme 3. This outcome suggests a significant role of the fluorine atom at the *alpha* position, where its electron-withdrawing effect likely influenced the feasibility of the following Michael addition for compounds **9a**–**d** (Scheme 2). Interestingly, such a reaction pathway was absent for derivatives **11a**–**d**, where the *alpha*-positioned proton exhibited a low p*K*_a_, favouring an easy elimination reaction. This is supported by the higher yields of products **12a**–**d** compared to their **11a**–**d** counterparts. The exclusive formation of *E* isomers in compounds **11a**–**d** was confirmed by the observed coupling constants (*J* ≈ 21 Hz) between the vinylic proton and the fluorine atom [41]. Moreover, the vicinal coupling constants between the F_β_ and H*_γ_* atoms ranging from 25–26 Hz for **11a**–**d** suggest a dihedral angle of approximately 170° between these atoms [42–45]. These findings are in alignment with DFT calculations (see Supporting Information File 1) and corroborate data for compounds **9a**–**d**.

**Scheme 4:**
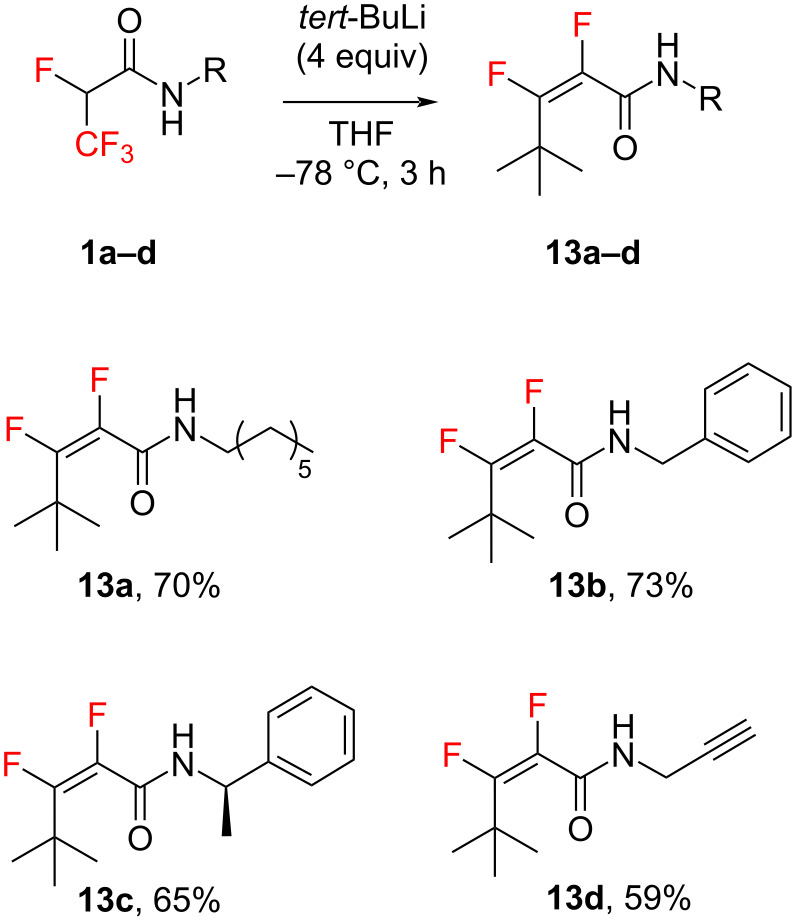
Michael addition of **1a**–**d** with *tert*-BuLi.

**Scheme 5:**
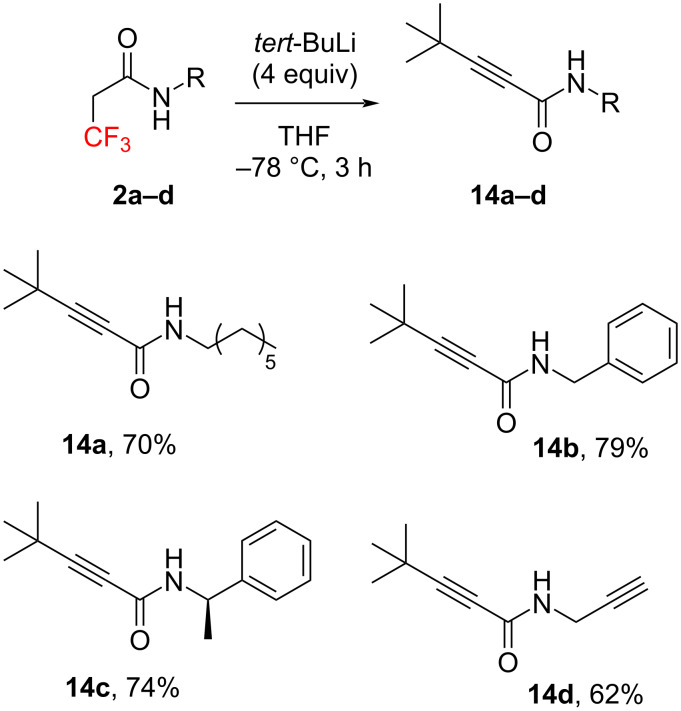
Michael addition of **2a**–**d** with *tert*-BuLi.

We further decided to use *tert*-BuLi in our research, considering its role as a stronger base and simultaneously as a weak nucleophile. We first performed the reaction with 2 equiv of *tert*-BuLi, which did not yield the expected results. The substrate was still observed in the reaction mixture. Only the use of 4 equiv of base gave the desired findings. The treatment of compounds **1a**–**d** and **2a**–**d**, respectively, with *tert*-BuLi induced the carbanion formation followed by an addition–elimination reaction, affording the corresponding fluorinated **13a**–**d** (Scheme 4) and nonfluorinated **14a**–**d** (Scheme 5) unsaturated products. Also this time, for compounds **13a**–**d**, the formation of only *Z* isomers was observed (Scheme 4). The stereochemistry was determined by ^19^F{^1^H} NMR spectroscopy methods by the observed coupling constants *J* ≈ 6 Hz between vinylic fluorine atoms [41]. Due to the steric hindrance, these compounds did not serve as good Michael acceptors for the next step.

Only elimination products **14a**–**d** were obtained from trifluorinated amides **2a**–**d**, showing good yields (Scheme 5).

**Scheme 6:**
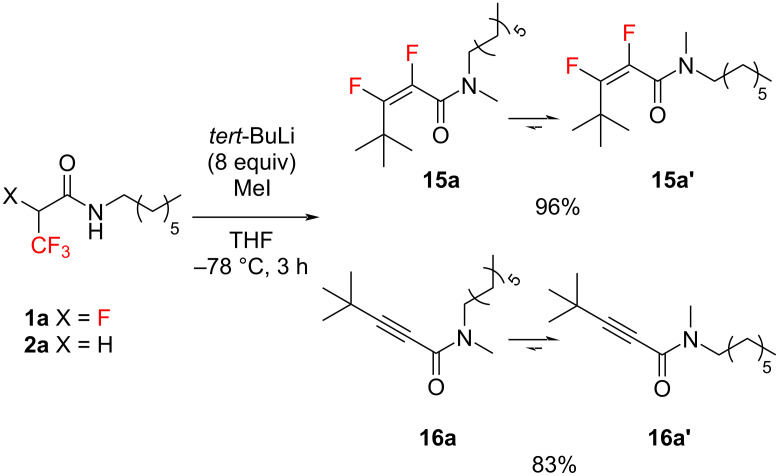
Formation of *N*-methylation products.

We also tried to perform a substitution reaction by treating compounds **1a** and **2a** with *tert*-BuLi, employing methyl iodide as the electrophile. However, similar to previous reactions, this did not yield substitution products at the *alpha* position, but to the addition–elimination reaction products. More importantly, the application of 8 equiv of *tert*-BuLi induced the formation of *N*-methylation products (Scheme 6).

Compounds **15a** (**15a’**) and **16a** (**16a’**) existed as two rotamers, in ratios of 1:1.15 and 1:1.76, respectively, with the predominant *cisoid* isomer. *Transoid* (*trans* **15a**(**16a**)) isomers contained a larger substituent at nitrogen located in the opposite direction to the carbonyl group, while *cisoid* (*cis* **15a’** (**16a’**)) isomers featured a smaller substituent at nitrogen located in the opposite direction to the carbonyl group. We determined the quantitative ratio as well as the *cis*/*trans* configuration of isomers by analyzing the differences in the chemical shift values in the ^1^H NMR spectra for NCH_3_ and NCH_2_- proton groups, based on our previous studies concerning fluorinated amides [46–47].

## Conclusion

In this study, we have established that tri- and tetrafluorinated amides, featuring a CF_3_ group at the α position, serve as effective motifs for designing stable *gem*-difluorovinyl and trifluorovinyl Michael acceptors. To our knowledge, this represents the inaugural instance of employing potent bases such as *n*-BuLi and *tert*-BuLi to fulfill dual roles as both base catalysts and Michael donors. The reactions exhibited remarkable stereoselectivity, a finding elucidated by DFT analysis. These results mark significant progress toward the synthesis of novel fluorinated building blocks. Our team is currently exploring the application of this methodology to amino acid substrates, aiming to contribute further to the burgeoning field of fluorinated peptidomimetics.

## Experimental

See Supporting Information File 1 for the Experimental section.

## Supporting Information

File 1 Detailed experimental procedures, DFT calculations, characterization data, and copies of ^1^H, ^13^C, ^19^F NMR and ^1^H−^13^C HSQC spectra.

## Data Availability

All data that supports the findings of this study is available in the published article and/or the supporting information of this article.
