# Supplementary material for: gem-Difluorovinyl and trifluorovinyl Michael acceptors in the synthesis of α,β-unsaturated fluorinated and nonfluorinated amides
Source: Beilstein J Org Chem. 2024 Nov 15;20:2946–53. doi: 10.3762/bjoc.20.247 (PMC11572014; doi:10.3762/bjoc.20.247)
Supplement: File 1 — Detailed experimental procedures, DFT calculations, characterization data, and copies of 1H, 13C, 19F NMR and 1H−13C HSQC spectra. [file Beilstein_J_Org_Chem-20-2946-s001.pdf]

BEILSTEIN JOURNAL OF ORGANIC CHEMISTRY

Supporting Information

for

gem-Difluorovinyl and trifluorovinyl Michael acceptors in the synthesis of α,β-unsaturated fluorinated and nonfluorinated amides

Monika Bilska-Markowska, Marcin Kaźmierczak, Wojciech Jankowski and Marcin Hoffmann

Beilstein J. Org. Chem. **2024,** *20,* 2946–2953. doi:10.3762/bjoc.20.247

Detailed experimental procedures, DFT calculations, characterization data, and copies of ^{1}H, ^{13}C, ^{19}F NMR and ^{1}H–^{13}C HSQC spectra

License and Terms: This is a supporting information file under the terms of the Creative Commons Attribution License (https://creativecommons.org/licenses/by/4.0). Please note that the reuse, redistribution and reproduction in particular requires that the author(s) and source are credited and that individual graphics may be subject to special legal provisions.
The license is subject to the *Beilstein Journal of Organic Chemistry* terms and conditions: (https://www.beilstein-journals.org/bjoc/terms)

Table of contents

1. Experimental section S1

1.1. General methods S1

1.2. Experimental procedures and compounds characterization S1

2. Copies of NMR spectra S13

3. DFT Calculations S67

3.1. Methods S67

3.2. Results S67

4. References S88

1. Experimental section

1.1. General methods

^{1}H NMR, ^{13}C NMR, ^{19}F NMR and 2D-NMR spectra were performed on Bruker ASCEND 400 (400 MHz) spectrometer. Chemical shifts of ^{1}H NMR were expressed in parts per million downfield from TMS as an internal standard (δ = 0) in $CDCl_3$. Chemical shifts of ^{13}C NMR were expressed in parts per million downfield and upfield from $CDCl_3$ as an internal standard (δ = 77.0). Chemical shifts of ^{19}F NMR were expressed in parts per million upfield from $CFCl_3$ as an internal standard (δ = 0) in $CDCl_3$. High-resolution mass spectra were recorded by electron spray (MS-ESI) technique using QToF Impact HD Bruker spectrometer. Reagent grade chemicals were used and solvents were dried by refluxing with sodium metal-benzophenone (THF) and distilled under an argon atmosphere. All moisture sensitive reactions were carried out under an argon atmosphere using oven-dried glassware. Reaction temperatures below 0 ^{o}C were obtained using a bath cooling (dry ice/*iso*-propanol). Thin-layer chromatography (TLC) was performed on Merck Kieselgel 60-F_{254} with EtOAc/hexane as developing systems. Visualization of the reactions products was achieved using UV light (254 nm) and a standard procedure (solution of potassium permanganate). Merck Kieselgel 60 (230–400 mesh) was used for column chromatography.

1.2. Experimental procedures and compounds characterization

1.2.1. Tri- and tetrafluorinated amides – starting materials

Starting compounds **1a–d** and **2a–d** for the preparation of designed products were prepared according to the synthetic pathway described earlier in our laboratory.[1]

1.2.2. General procedure for the synthesis of α,β- and α-fluorinated unsaturated amides with *n*-BuLi

To the mixture of corresponding amide in dry THF placed in a round-bottom flask and cooled to −78°C, *n*-BuLi (4 equiv, 2 M in cyclohexane) was added dropwise under an argon atmosphere. The solution was stirred at the −78 °C for 3 h. Next, the cooling bath was removed and the reaction mixture was left overnight at room temperature with stirring. Following this, the reaction mixture was carefully quenched by dropwise addition of saturated NH_4Cl aq. (10 mL), and then extracted with AcOEt (3 × 15 mL). The combined organic phases were dried (Na_2SO_4), the volatiles were removed using a rotary evaporator and the residue was subjected to careful column chromatography (hexane - 2% EtOAc/hexane) to yield the desired products.

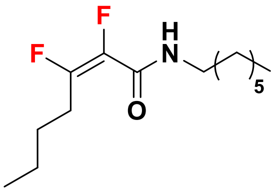

(*Z*)-2,3-Difluoro-*N*-heptylhept-2-enamide (9a)

The pure product was isolated as a white solid in 68% yield (45 mg). **^{1}H NMR** (400 MHz, $CDCl_3$) *δ* 6.22 (bs, 1H, N*H*), 3.32 (dt, *J* = 7.4, 6.1 Hz, 2H, NHC*H*$_2$), 2.87 (dtd, *J* = 26.8, 7.6, 2.8 Hz, 2H, C*H*$_2$CF), 1.62-1.52 (m, 2H, C*H*$_2$), 1.40 (dt, *J* = 15.0, 7.4 Hz, 2H, CH_3C*H*$_2$$CH_2$), 1.35-1.25 (m, 10H, 5xC*H*$_2$), 0.93 (t, *J* = 7.4 Hz, 3H, C*H*$_3$), 0.87 (t, *J* = 6.6 Hz, 3H, C*H*$_3$); **^{13}C NMR** (101 MHz, $CDCl_3$) *δ* 160.09 (dd, *J* = 24.2, 7.7 Hz, *C*O), 157.03 (dd, *J* = 268.2, 11.8 Hz, *C*F), 139.05 (dd, *J* = 254.3, 18.4 Hz, *C*FCO), 39.21 (NH*C*H$_2$), 31.71, 29.47, 28.91, 27.98 (d, *J* = 2.9 Hz, *C*H$_2$CH$_2$CF), 27.77 (dd, *J* = 19.8, 1.9 Hz, *C*H$_2$CF), 26.85, 22.57, 22.13, 14.04, 13.73; **^{19}F NMR** (376 MHz, $CDCl_3$) *δ* -112.58 (t, *J* = 26.9 Hz, 1F, *F*), -154.18 ÷ -158.21 (m, 1F, *F*); **^{19}F{/^{1}H} NMR** (376 MHz, $CDCl_3$) *δ* -112.58 (d, *J* = 2.0 Hz, 1F, *F*), -156.57 (d, *J* = 2.0 Hz, 1F, *F*); **HRMS** (ESI) m/z: calcd. for $C_{14}H_{26}F_2NO^+$ 262.1982 [M+H]$^+$; found 262.1974, calcd. for $C_{14}H_{25}F_2NONa^+$ 284.1801 [M+Na]$^+$; found 284.1792, calcd. for $C_{14}H_{25}F_2NOK^+$ 300.1541 [M+K]$^+$; found 300.2693.

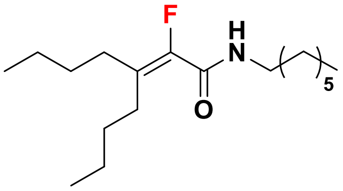

3-Butyl-2-fluoro-*N*-heptylhept-2-enamide (10a)

The pure product was isolated as a colorless oil in 13% yield (10 mg). **^{1}H NMR** (400 MHz, $CDCl_3$) *δ* 6.20 (bs, 1H, N*H*), 3.29 (q, *J* = 6.7 Hz, 2H, NHC*H*$_2$), 2.69-2.53 (m, 2H, C*H*$_2$), 2.17 (td, *J* = 7.7, 3.6 Hz, 2H, C*H*$_2$), 1.62-1.49 (m, 2H, C*H*$_2$), 1.49-1.37 (m, 4H, 2xC*H*$_2$), 1.37-1.22 (m, 12H, 6xC*H*$_2$), 0.97-0.82 (m, 9H, 3xC*H*$_3$); **^{13}C NMR** (101 MHz, $CDCl_3$) *δ* 161.12 (d, *J* = 31.9 Hz, *C*O), 140.05 (d, *J* = 249.4 Hz, *C*F), 133.95 (dd, *J* = 11.3 Hz, *C*=CF), 38.98 (NH*C*H$_2$), 31.74, 30.71 (d, *J* = 2.8 Hz), 29.79 (d, *J* = 1.8 Hz), 29.65 (d, *J* = 9.0 Hz), 29.55, 29.11 (d, *J* = 3.2 Hz), 28.96, 26.93, 22.91, 22.71, 22.59, 14.06, 13.99, 13.89; **^{19}F NMR** (376 MHz, $CDCl_3$) *δ* -130.19 (d, *J* = 4.4 Hz, 1F, *F*); **^{19}F{/^{1}H} NMR** (376 MHz, $CDCl_3$) *δ* -130.19 (s, 1F, *F*); **HRMS** (ESI) m/z: calcd. for $C_{18}H_{35}FNO^+$ 300.2702 [M+H]$^+$; found 300.2693, calcd. for $C_{18}H_{34}FNONa^+$ 322.2522 [M+Na]$^+$; found 322.2511.

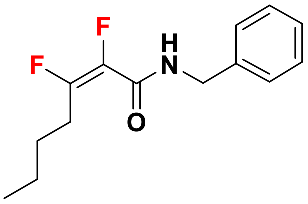

(*Z*)-*N*-Benzyl-2,3-difluorohept-2-enamide (9b)

The pure product was isolated as a white solid in 59% yield (39 mg). **^{1}H NMR** (400 MHz, $CDCl_3$) *δ* 7.43-7.17 (m, 5H, Ph), 6.54 (bs, 1H, N*H*), 4.51 (d, *J* = 5.8 Hz, 2H, NHC*H*$_2$), 2.89 (dtd, *J* = 26.7, 7.5, 2.8 Hz, 2H, C*H*$_2$CF), 1.65-1.56 (m, 2H, C*H*$_2$), 1.40 (h, *J* = 7.3 Hz, 2H, CH_3C*H*$_2$$CH_2$), 0.94 (t, *J* = 7.4 Hz, 3H, C*H*$_3$); **^{13}C NMR** (101 MHz, $CDCl_3$) *δ* 160.10 (dd, *J* = 24.4, 7.9 Hz, *C*O), 157.45 (dd, *J* = 269.1, 11.7 Hz, *C*F), 138.94 (dd, *J* = 254.1, 18.6 Hz, *C*FCO), 137.42, 128.85, 127.86, 127.81, 43.18 (NH*C*H_2), 27.97 (d, *J* = 2.8 Hz, *C*$H_2$$CH_2$CF), 27.82 (dd, *J* = 19.8, 2.0 Hz, *C*H_2CF), 22.15, 13.75; **^{19}F NMR** (376 MHz, $CDCl_3$) -111.40 (t, *J* = 26.7 Hz, 1F, *F*), -155.74 ÷ -157-79 (m, 1F, *F*); **^{19}F{/^{1}H} NMR** (376 MHz, $CDCl_3$) -111.40 (d, *J* = 2.0 Hz, 1F, *F*), -156.69 (d, *J* = 1.9 Hz, 1F, *F*); **HRMS** (ESI) m/z: calcd. for $C_{14}H_{18}F_2NO^+$ 254.1356 [M+H]$^+$; found 254.1353; calcd. for $C_{14}H_{17}F_2NONa^+$ 276.1175 [M+Na]$^+$; found 276.1172; calcd. for $C_{14}H_{17}F_2NOK^+$ 292.0915 [M+K]$^+$; found 292.2072.

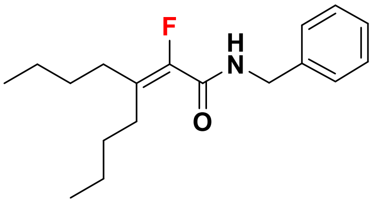

***N*-Benzyl-3-butyl-2-fluorohept-2-enamide (10b)**

The pure product was isolated as a colorless oil in 15% yield (11 mg). **^{1}H NMR** (400 MHz, $CDCl_3$) *δ* 7.39-7.24 (m, 5H, Ph), 6.51 (bs, 1H, N*H*), 4.50 (d, *J* = 5.7 Hz, 2H, NHC*H*$_2$), 2.69-2.60 (m, 2H, C*H*$_2$), 2.18 (td, *J* = 7.8, 3.6 Hz, 2H, C*H*$_2$), 1.52-1.24 (m, 8H, 4xC*H*$_2$), 0.92 (t, *J* = 7.1 Hz, 3H, C*H*$_3$), 0.90 (t, *J* = 7.1 Hz, 3H, C*H*$_3$); **^{13}C NMR** (101 MHz, $CDCl_3$) *δ* 161.03 (d, *J* = 32.3 Hz, *C*O), 145.86 (d, *J* = 248.9 Hz, *C*FCO), 137.97, 134.79 (d, *J* = 11.2 Hz, *C*=CF), 128.75, 127.87, 127.59, 43.00 (NH*C*H_2), 30.71 (d, *J* = 2.9 Hz), 29.78 (d, *J* = 2.0 Hz), 29.70 (d, *J* = 8.9 Hz), 29.15 (d, *J* = 3.2 Hz), 22.92, 22.71, 14.01, 13.89; **^{19}F NMR** (376 MHz, $CDCl_3$) *δ* -130.36 (d, *J* = 4.5 Hz, 1F, *F*); **^{19}F{/^{1}H} NMR** (376 MHz, $CDCl_3$) *δ* -130.36 (s, 1F, *F*); **HRMS** (ESI) m/z: calcd. for $C_{18}H_{27}FNO^+$ 292.2076 [M+H]$^+$; found 292.2072; calcd. for $C_{18}H_{26}FNONa^+$ 314.1896 [M+Na]$^+$; found 314.1892; calcd. for $C_{18}H_{26}FNOK^+$ 330.1635 [M+K]$^+$; found 330.0564.

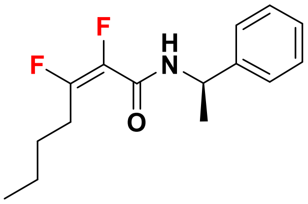

(*R,Z*)-2,3-Difluoro-*N*-(1-phenylethyl)hept-2-enamide (9c)

The pure product was isolated as a colorless oil in 61% yield (30 mg). **^{1}H NMR** (400 MHz, $CDCl_3$) δ 7.42-7.20 (m, 5H, Ph), 6.44 (bs, 1H, N*H*), 5.17 (p, *J* = 6.8 Hz, 1H, NHC*H*), 3.05-2.67 (m, 2H, C*H*$_2$CF), 1.59 (dd, 2H, *J* = 8.9, 6.0 Hz, C*H*$_2$), 1.54 (d, *J* = 6.9 Hz, 3H, C*H*$_3$CH), 1.38 (h, *J* = 7.3 Hz, 2H, C*H*$_2$), 0.92 (t, *J* = 7.3 Hz, 3H, C*H*$_3$); **^{13}C NMR** (101 MHz, $CDCl_3$) δ 159.30 (dd, *J* = 24.4, 7.8 Hz, *C*O), 157.34 (dd, *J* = 268.9, 11.7 Hz, *C*F), 142.55, 138.92 (dd, *J* = 254.1, 18.3 Hz, *C*FCO), 128.82, 127.64, 126.13, 48.70 (NHCH$_2$), 27.94 (d, *J* = 2.8 Hz), 27.80 (dd, *J* = 19.8, 2.1 Hz), 22.15, 21.87, 13.75; **^{19}F NMR** (376 MHz, $CDCl_3$) -112.13 (t, *J* = 26.5 Hz, 1F, *F*), -155.99 ÷ -159.11 (m, 1F, *F*); **^{19}F{/^{1}H} NMR** (376 MHz, $CDCl_3$) -112.13 (d, *J* = 2.0 Hz, 1F, *F*), -156.97 (d, *J* = 2.0 Hz, 1F, *F*); **HRMS** (ESI) m/z: calcd. for $C_{15}H_{20}F_2NO^+$ 268.1512 [M+H]$^+$; found 268.1503; calcd. for $C_{15}H_{19}F_2NONa^+$ 290.1332 [M+Na]$^+$; found 290.1323; calcd. for $C_{15}H_{19}F_2NOK^+$ 306.1071 [M+K]$^+$; found 306.1060.

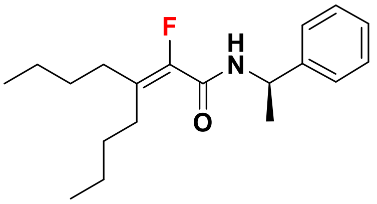

(*R*)-3-Butyl-2-fluoro-*N*-(1-phenylethyl)hept-2-enamide (10c)

The pure product was isolated as a colorless oil in 14% yield (8 mg). **^{1}H NMR** (400 MHz, $CDCl_3$) δ 7.38-7.24 (m, 5H, Ph), 6.43 (bs, 1H, N*H*), 5.25-5.06 (m, 1H, NHC*H*), 2.72-2.50 (m, 2H, C*H*$_2$CF), 2.17 (td, *J* = 7.8, 3.5 Hz, 2H, C*H*$_2$), 1.56 (s, 2H, C*H*$_2$), 1.52 (d, *J* = 6.9 Hz, 3H, C*H*$_3$CH), 1.49-1.26 (m, 6H, 3xC*H*$_2$), 0.91 (t, *J* = 7.2 Hz, 3H, C*H*$_3$), 0.90 (t, *J* = 7.1 Hz, 3H, C*H*$_3$); **^{13}C NMR** (101 MHz, $CDCl_3$) δ 160.23 (d, *J* = 32.0 Hz, *C*O), 145.88 (d, *J* = 248.6 Hz, *C*FCO), 143.07, 134.54 (d, *J* = 11.1 Hz, *C*=CF), 128.72, 127.41, 126.18, 48.25 (NHCH$_2$), 30.65 (d, *J* = 2.4 Hz), 29.79, 29.68 (d, *J* = 8.9 Hz), 29.10 (d, *J* = 3.1 Hz), 22.90, 22.73, 22.00, 14.00, 13.89; **^{19}F NMR** (376 MHz, $CDCl_3$) δ -130.56 (d, *J* = 4.2 Hz, 1F, *F*); **^{19}F{/^{1}H} NMR** (376 MHz, $CDCl_3$) δ -130.56 (s, 1F, *F*); **HRMS** (ESI) m/z: calcd. for $C_{19}H_{29}FNO^+$ 306.2233 [M+H]$^+$; found 306.2222; calcd. for $C_{19}H_{28}FNONa^+$ 328.2052 [M+Na]$^+$; found 328.2042; calcd. for $C_{19}H_{28}FNOK^+$ 344.1792 [M+K]$^+$; found 344.1781.

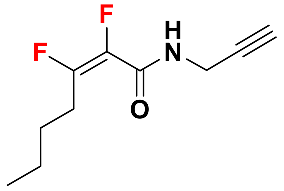

(*Z*)-2,3-Difluoro-*N*-(prop-2-yn-1-yl)hept-2-enamide (9d)

The pure product was isolated as a colorless oil in 48% yield (34 mg). **^{1}H NMR** (400 MHz, $CDCl_3$) δ 6.40 (bs, 1H, N*H*), 4.13 (dd, *J* = 5.2, 2.6 Hz, 1H, NHC*H*$_2$), 2.86 (dtd, *J* = 26.7, 7.6, 2.9 Hz, 2H, C*H*$_2$CF), 2.28 (t, *J* = 2.6 Hz, 1H, CC*H*), 1.65-1.53 (m, 2H, C*H*$_2$), 1.46-1.34 (m, 2H, C*H*$_2$), 1.39 (h, *J* = 7.3 Hz, 2H, C*H*$_2$), 0.93 (t, *J* = 7.3 Hz, 3H, C*H*$_3$); **^{13}C NMR** (101 MHz, $CDCl_3$) δ 159.89 (dd, *J* = 25.1, 8.1 Hz, *C*O), 157.77 (dd, *J* = 270.1, 11.4 Hz, *C*F), 138.65 (dd, *J* = 253.6, 18.9 Hz, *C*FCO), 78.65 (*C*CH), 72.12 (C*C*H), 28.89 (NHCH$_2$), 27.92 (d, *J* = 3.0 Hz), 27.77 (dd, *J* = 19.6, 2.1 Hz), 22.11, 13.72; **^{19}F NMR** (376 MHz, $CDCl_3$) -110.27 (t, *J* = 26.7 Hz, 1F, *F*), -156.54 ÷ -158.97 (m, 1F, *F*); **^{19}F{/^{1}H} NMR** (376 MHz, $CDCl_3$) -110.27 (d, *J* = 2.1 Hz, 1F, *F*), -

157.29 (d, J = 2.0 Hz, 1F, F); **HRMS** (ESI) m/z: calcd. for $C_{10}H_{14}F_2NO^+$ 202.1043 [M+H]$^+$; found 202.1046.

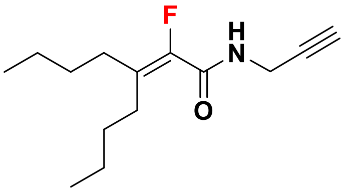

3-Butyl-2-fluoro-*N*-(prop-2-yn-1-yl)hept-2-enamide (10d)

The product was obtained in trace amounts.
^{19}F NMR (376 MHz, $CDCl_3$) δ -130.97 (d, J = 4.1 Hz, 1F, F).

1.2.3. General procedure for the synthesis of β-fluorinated and nonfluorinated unsaturated amides with *n*-BuLi

To the mixture of corresponding amide in dry THF placed in a round-bottom flask and cooled to −78 °C, *n*-BuLi (4 equiv, 2 M in cyclohexane) was added dropwise under an argon atmosphere. The solution was stirred at the −78 °C for 3 h. Next, the cooling bath was removed and the reaction mixture was left overnight at room temperature with stirring. Following this, the reaction mixture was carefully quenched by dropwise addition of saturated NH_4Cl aq (10 mL), and then extracted with AcOEt (3 × 15 mL). The combined organic phases were dried (Na_2SO_4), the volatiles were removed using a rotary evaporator and the residue was subjected to careful column chromatography (hexane - 10% EtOAc/hexane) to yield the desired products.

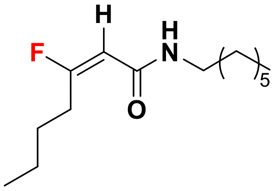

(*E*)-3-Fluoro-*N*-heptylhept-2-enamide (11a)

The pure product was isolated as a pale yellow oil in 21% yield (27 mg). **^{1}H NMR** (400 MHz, $CDCl_3$) δ 5.42 (d, 1H, J = 20.6 Hz, H_v), 5.33 (bs, 1H, NH), 3.27 (dt, J = 7.3, 5.9 Hz, 2H, NHCH_2), 2.86 (dt, J = 26.1, 7.6 Hz, 2H, CH_2CF), 1.63-1.47 (m, 2H, CH_2), 1.39 (h, J = 7.4 Hz, 2H, CH_3C$H_2$$CH_2$), 1.35-1.21 (m, 10H, 5xCH_2), 0.92 (t, J = 7.3 Hz, 3H, CH_3), 0.87 (t, J = 6.6 Hz, 3H, CH_3); **^{13}C NMR** (101 MHz, $CDCl_3$) δ 174.40 (d, J = 269.9 Hz, CF), 164.94 (d, J = 22.5 Hz, CO), 102.30 (d, J = 25.4 Hz, CH$_v$), 39.49 (NHCH$_2$), 31.75, 29.65, 29.17 (d, J = 22.9 Hz, CH$_2$CF), 28.96, 28.18, 26.92, 22.58, 22.28, 14.06, 13.79; **^{19}F NMR** (376 MHz, $CDCl_3$) δ -82.12 (dt, J = 26.1, 20.6 Hz, 1F, F); **^{19}F{/^{1}H} NMR** (376 MHz, $CDCl_3$) δ -82.12 (s, 1F, F); **HRMS** (ESI) m/z: calcd. for $C_{14}H_{27}FNO^+$ 244.2076 [M+H]$^+$; found 244.2072, calcd. for $C_{14}H_{26}FNONa^+$ 266.1896 [M+Na]$^+$; found 266.1892, calcd. for $C_{14}H_{26}FNOK^+$ 282.1635 [M+K]$^+$; found 282.1627.

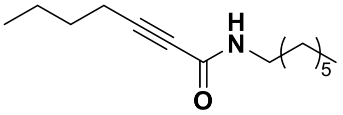

N-Heptylhept-2-ynamide (12a)

The pure product was isolated as a pale yellow oil in 54% yield (57 mg). **^{1}H NMR** (400 MHz, $CDCl_3$) δ 5.96 (bs, 1H, N*H*), 3.27 (td, *J* = 7.3, 6.0 Hz, 2H, NHC*H*$_2$), 2.29 (t, *J* = 7.1 Hz, 2H, C*H*$_2$C), 1.58-1.46 (m, 4H, 2xC*H*$_2$), 1.45-1.37 (m, 2H, C*H*$_2$), 1.35-1.25 (m, 8H, 4xC*H*$_2$), 0.91 (t, *J* = 7.3 Hz, 3H, C*H*$_3$), 0.88 (t, *J* = 7.3 Hz, 3H, C*H*$_3$); **^{13}C NMR** (101 MHz, $CDCl_3$) δ 153.62 (*C*O), 87.02 ($C_{sp}C_{sp}$), 75.63 ($C_{sp}C_{sp}$), 39.82 (NHCH$_2$), 31.70, 29.79, 29.32, 28.90, 26.81, 22.55, 21.93, 18.25, 14.02, 13.47; **HRMS** (ESI) m/z: calcd. for $C_{14}H_{26}NO^+$ 224.2014 [M+H]$^+$; found 224.2010; calcd. for $C_{14}H_{25}NONa^+$ 246.1833 [M+Na]$^+$; found 246.1828.

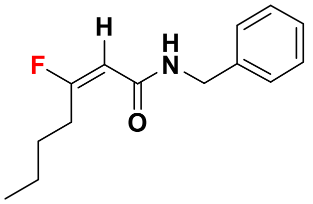

(*E*)-*N*-Benzyl-3-fluorohept-2-enamide (11b)

The pure product was isolated as a colorless oil in 28% yield (19 mg). **^{1}H NMR** (400 MHz, $CDCl_3$) δ 7.38-7.23 (m, 5H, Ph), 5.69 (bs, 1H, N*H*), 5.46 (d, *J* = 20.4 Hz, 1H, H_v), 4.46 (d, *J* = 5.7 Hz, 1H, NHC*H*$_2$), 2.95-2.81 (m, 2H, C*H*$_2$CF), 1.64-1.52 (m, 2H, CH$_2$), 1.46-1.34 (m, 2H, CH$_2$), 0.93 (t, *J* = 7.4 Hz, 3H, C*H*$_3$); **^{13}C NMR** (101 MHz, $CDCl_3$) δ 174.91 (d, *J* = 271.0 Hz, *C*F), 164.86 (d, *J* = 22.9 Hz, *C*O), 138.08, 128.72, 127.80, 127.56, 102.04 (d, *J* = 26.0 Hz, CH_v), 43.49 (NH*C*H$_2$), 29.22 (d, *J* = 22.4 Hz, *C*H$_2$CF), 28.12, 22.24, 13.76; **^{19}F NMR** (376 MHz, $CDCl_3$) -80.81 (td, *J* = 25.8, 20.2 Hz, 1F, *F*); **^{19}F{/^{1}H} NMR** (376 MHz, $CDCl_3$) -80.82 (s, 1F, *F*); **HRMS** (ESI) m/z: calcd. for $C_{14}H_{19}FNO^+$ 236.1450 [M+H]$^+$; found 236.1439; calcd. for $C_{14}H_{18}FNONa^+$ 258.1270 [M+Na]$^+$; found 258.1260.

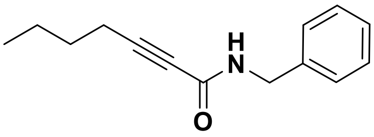

N-Benzylhept-2-ynamide (12b)

The pure product was isolated as a colorless oil in 60% yield (38 mg). **^{1}H NMR** (400 MHz, $CDCl_3$) δ 7.39-7.25 (m, 5H, Ph), 6.01 (bs, 1H, N*H*), 4.47 (d, *J* = 5.8 Hz, 1H, NHC*H*$_2$), 2.29 (t, *J* = 7.0 Hz, 2H, C*H*$_2$C), 1.59-1.47 (m, 2H, C*H*$_2$), 1.46-1.34 (m, 2H, CH$_2$), 0.91 (t, *J* = 7.3 Hz, 3H, C*H*$_3$); **^{13}C NMR** (101 MHz, $CDCl_3$) δ 153.45 (*C*O), 137.45, 128.80, 127.94, 127.75, 87.94 ($C_{sp}C_{sp}$), 75.37 ($C_{sp}C_{sp}$), 43.85 (NHCH$_2$), 29.76, 21.96, 18.30, 13.50; **HRMS** (ESI) m/z: calcd. for $C_{14}H_{18}NO^+$ 216.1388 [M+H]$^+$; found 216.1375; calcd. for $C_{14}H_{17}NONa^+$ 238.1207 [M+Na]$^+$; found 238.1196.

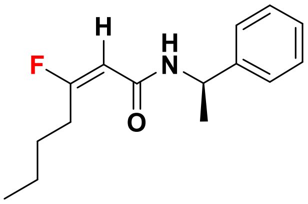

(R,E)-3-Fluoro-N-(1-phenylethyl)hept-2-enamide (11c)

The pure product was isolated as a colorless oil in 26% yield (16 mg). **^{1}H NMR** (400 MHz, $CDCl_3$) δ 7.39-7.22 (m, 5H, Ph), 5.59 (d, J = 8.1 Hz, 1H, NH), 5.43 (d, J = 20.5 Hz, 1H, H_v), 5.15 (p, J = 7.1 Hz, 1H, NHCH), 2.96-2.74 (m, 2H, CH_2CF), 1.62-1.51 (m, 2H, CH_2), 1.50 (d, J = 6.9 Hz, 3H, CH_3CH), 1.43-1.32 (m, 2H, CH_2), 0.91 (t, J = 7.3 Hz, 3H, CH_3); **^{13}C NMR** (101 MHz, $CDCl_3$) δ 174.77 (d, J = 270.7 Hz, CF), 164.10 (d, J = 22.7 Hz, CO), 143.13, 128.73, 127.43, 126.15, 102.24 (d, J = 25.8 Hz, CH_v), 48.73 (NHCH), 29.22 (d, J = 22.6 Hz, CH$_2$CF), 28.13, 22.28, 21.84, 13.79; **^{19}F NMR** (376 MHz, $CDCl_3$) -80.69 (td, J = 25.9, 20.3 Hz, 1F, F); **^{19}F{/^{1}H} NMR** (376 MHz, $CDCl_3$) -80.69 (s, 1F, F); **HRMS** (ESI) m/z: calcd. for $C_{15}H_{21}FNO^+$ 250.1607 [M+H]$^+$; found 250.1593; calcd. for $C_{15}H_{20}FNONa^+$ 272.1426 [M+Na]$^+$; found 272.1416.

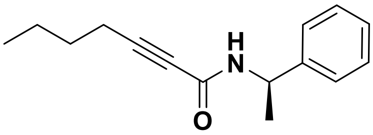

(R)-N-(1-Phenylethyl)hept-2-ynamide (12c)

The pure product was isolated as a colorless oil in 64% yield (36 mg). **^{1}H NMR** (400 MHz, $CDCl_3$) δ 7.37-7.25 (m, 5H, Ph), 5.98 (bs, 1H, NH), 5.22-5.09 (m, 1H, NHCH), 2.28 (t, J = 7.1 Hz, 2H, CH_2C), 1.59-1.47 (m, 2H, CH_2), 1.51 (d, J = 6.8 Hz, 3H, CH_3CH), 1.49-1.33 (m, 2H, CH_2), 0.91 (t, J = 7.3 Hz, 3H, CH_3); **^{13}C NMR** (101 MHz, $CDCl_3$) δ 152.67 (CO), 142.41, 128.75, 127.59, 126.26, 87.51 ($C_{sp}C_{sp}$), 75.58 ($C_{sp}C_{sp}$), 49.14 (NHCH), 29.77, 21.98, 21.49, 18.30, 13.51; **HRMS** (ESI) m/z: calcd. for $C_{15}H_{20}NO^+$ 230.1544 [M+H]$^+$; found 230.1529; calcd. for $C_{15}H_{19}NONa^+$ 252.1364 [M+Na]$^+$; found 252.1351 calcd. for $C_{15}H_{19}NOK^+$ 268.1103 [M+K]$^+$; found 268.1082.

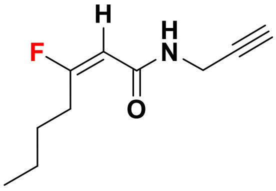

(E)-3-Fluoro-N-(prop-2-yn-1-yl)hept-2-enamide (11d)

The pure product was isolated as a colorless oil in 19% yield (11 mg). **^{1}H NMR** (400 MHz, $CDCl_3$) δ 5.51 (bs, 1H, NH), 5.44 (d, J = 20.1 Hz, 1H, H_v), 4.08 (dd, J = 5.3, 2.6 Hz, 2H, NHCH_2), 2.85 (dt, J = 26.1, 7.5 Hz, 2H, CH_2CF), 2.24 (t, J = 2.6 Hz, 1H, CH), 1.63-1.51 (m, 2H, CH_2), 1.44-1.35 (m, 2H, CH_2), 0.92 (t, J = 7.3 Hz, 3H, CH_3); **^{13}C NMR** (101 MHz, $CDCl_3$) δ 175.51 (d, J = 272.0 Hz, CF), 164.62 (d, J = 23.0 Hz, CO), 101.56 (d, J = 26.5 Hz, CH_v), 79.37 (CCH), 71.70 (CCH), 29.29 (d, J = 22.3 Hz, CH$_2$CF), 29.11 (NHCH$_2$), 28.11, 22.26, 13.77; **^{19}F NMR**

(376 MHz, $CDCl_3$) -79.05 (td, *J* = 26.2, 20.1 Hz, 1F, *F*); **^{19}F{^{1}H} NMR** (376 MHz, $CDCl_3$) -79.05 (s, 1F, *F*); **HRMS** (ESI) m/z: calcd. for $C_{10}H_{15}FNO^+$ 184.1137 $[M+H]^+$; found 184.1128; calcd. for $C_{10}H_{14}FNONa^+$ 206.0957 $[M+Na]^+$; found 206.0954.

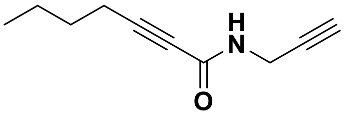

***N*-(Prop-2-yn-1-yl)hept-2-ynamide (12d)**

The pure product was isolated as a colorless oil in 51% yield (26 mg). **^{1}H NMR** (400 MHz, $CDCl_3$) δ 5.90 (bs, 1H, N*H*), 4.08 (dd, *J* = 5.4, 2.6 Hz, 2H, NHC*H*$_2$), 2.30 (t, *J* = 7.1 Hz, 2H, C*H*$_2$C), 2.26 (t, *J* = 2.6 Hz, 1H, CC*H*), 1.59-1.47 (m, 2H, C*H*$_2$), 1.59-1.50 (m, 2H, CH_2), 1.47-1.38 (m, 2H, C*H*$_2$), 0.92 (t, *J* = 7.3 Hz, 3H, C*H*$_3$); **^{13}C NMR** (101 MHz, $CDCl_3$) δ 153.05 (*C*O), 88.61 ($C_{sp}C_{sp}$), 78.66 (*C*CH), 74.88 ($C_{sp}C_{sp}$), 72.07 (C*C*H), 29.69, 29.43 (NH*C*H$_2$), 21.95, 18.29, 13.49; **HRMS** (ESI) m/z: calcd. for $C_{10}H_{14}NO^+$ 164.1075 $[M+H]^+$; found 164.1072; calcd. for $C_{10}H_{13}NONa^+$ 186.0894 $[M+Na]^+$; found 186.0893.

1.2.4. General procedure for the synthesis of *α, β*-fluorinated unsaturated amides with *tert*-BuLi

To the mixture of corresponding amide in dry THF placed in a round-bottom flask and cooled to −78°C, *tert*-BuLi (4 equiv, 1.7 M in pentane) was added dropwise under an argon atmosphere. The solution was stirred at the −78 °C for 3 h. Next, the cooling bath was removed and the reaction mixture was left overnight at room temperature with stirring. Following this, the reaction mixture was carefully quenched by dropwise addition of saturated NH_4Cl aq (10 mL), and then extracted with AcOEt (3 × 15 mL). The combined organic phases were dried (Na_2SO_4), the volatiles were removed using a rotary evaporator and the residue was subjected to careful column chromatography (hexane - 2% EtOAc/hexane) to yield the desired products.

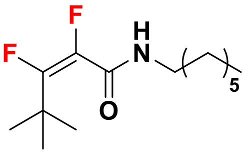

(*Z*)-2,3-Difluoro-*N*-heptyl-4,4-dimethylpent-2-enamide (13a)

The pure product was isolated as a colorless oil in 70% yield (37 mg). **^{1}H NMR** (400 MHz, $CDCl_3$) δ 6.21 (bs, 1H, N*H*), 3.32 (tdd, *J* = 7.0, 5.9, 1.0 Hz, 2H, NHC*H*$_2$), 1.54 (q, *J* = 7.2 Hz, 2H, C*H*$_2$), 1.34 (d, *J* = 2.7 Hz, 9H, C(C*H*$_3$)$_3$), 1.32-1.16 (m, 8H, 4xC*H*$_2$), 0.87 (t, *J* = 6.8 Hz, 3H, C*H*$_3$); **^{13}C NMR** (101 MHz, $CDCl_3$) δ 163.53 (dd, *J* = 272.1, 13.0 Hz, *C*F), 159.74 (dd, *J* = 26.2, 6.8 Hz, *C*O), 140.65 (dd, *J* = 253.1, 24.4 Hz, *C*FCO), 39.32 (NH*C*H$_2$), 34.76 (dd, *J* = 20.1, 1.7 Hz, *C*CF), 31.72, 29.43, 28.90, 27.27 (dd, *J* = 6.1, 2.0 Hz), 26.85, 22.56, 14.04; **^{19}F NMR** (376 MHz, $CDCl_3$) -112.32 ÷ -112.42 (m, 1F, *F*), -149.61 (t, *J* = 4.9 Hz, 1F, *F*); **^{19}F{^{1}H} NMR** (376 MHz, $CDCl_3$) -112.37 (d, *J* = 5.7 Hz, 1F, *F*), -149.61 (d, *J* = 6.1 Hz, 1F, *F*); **HRMS** (ESI) m/z:

calcd. for $C_{14}H_{26}F_2NO^+$ 262.1982 [M+H]$^+$; found 262.1973; calcd. for $C_{14}H_{25}F_2NONa^+$ 284.1801 [M+Na]$^+$; found 284.1797.

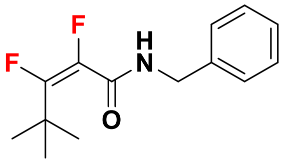

(*Z*)-*N*-Benzyl-2,3-difluoro-4,4-dimethylpent-2-enamide (13b)

The pure product was isolated as a colorless oil in 73% yield (48 mg). **^{1}H NMR** (400 MHz, $CDCl_3$) *δ* 7.38-7.25 (m, 5H, Ph), 6.53 (bs, 1H, N*H*), 4.51 (d, *J* = 5.8 Hz, 2H, NHC*H*$_2$), 1.35 (d, *J* = 2.8 Hz, 9H, C(C*H*$_3$)); **^{13}C NMR** (101 MHz, $CDCl_3$) *δ* 164.05 (dd, *J* = 273.0, 12.9 Hz, *C*F), 159.73 (dd, *J* = 26.5, 6.9 Hz, *C*O), 140.53 (dd, *J* = 252.8, 24.6 Hz, *C*FCO), 137.48, 128.84, 127.83, 127.77, 43.28 (NH*C*H$_2$), 34.85 (dd, *J* = 20.0, 1.6 Hz, *C*CF), 27.28 (dd, *J* = 6.1, 1.9 Hz); **^{19}F NMR** (376 MHz, $CDCl_3$) -110.84 ÷ 110.93 (m, 1F, *F*), -149.89 (t, *J* = 4.8 Hz, 1F, *F*); **^{19}F{/^{1}H} NMR** (376 MHz, $CDCl_3$) -110.89 (d, *J* = 5.7 Hz, 1F, *F*), -149.89 (d, *J* = 6.1 Hz, 1F, *F*); **HRMS** (ESI) m/z: calcd. for $C_{14}H_{18}F_2NO^+$ 254.1356 [M+H]$^+$; found 254.1351; calcd. for $C_{14}H_{17}F_2NONa^+$ 276.1175 [M+Na]$^+$; found 276.1175.

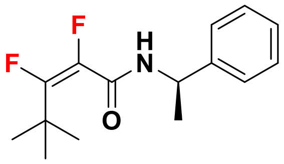

(*R,Z*)-2,3-Difluoro-4,4-dimethyl-*N*-(1-phenylethyl)pent-2-enamide (13c)

The pure product was isolated as a white solid in 65% yield (36 mg). **^{1}H NMR** (400 MHz, $CDCl_3$) *δ* 7.40-7.25 (m, 5H, Ph), 6.42 (bs, 1H, N*H*), 5.17 (ddd, *J* = 8.2, 6.8, 1.5 Hz, 2H, NHC*H*), 1.54 (d, *J* = 6.9 Hz, 3H, CHC*H*$_3$), 1.32 (d, *J* = 2.7 Hz, 9H, C(C*H*$_3$)); **^{13}C NMR** (101 MHz, $CDCl_3$) *δ* 163.85 (dd, *J* = 272.8, 12.9 Hz, *C*F), 158.93 (dd, *J* = 26.4, 6.8 Hz, *C*O), 142.55, 140.52 (dd, *J* = 252.8, 24.5 Hz, *C*FCO), 128.79, 127.59, 126.11, 48.75 (NH*C*H), 34.80 (dd, *J* = 20.1, 1.6 Hz, *C*CF), 27.26 (dd, *J* = 6.0, 1.9 Hz), 21.83; **^{19}F NMR** (376 MHz, $CDCl_3$) -109.91 ÷ -112.34 (m, 1F, *F*), -149.62 (t, *J* = 4.8 Hz, 1F, *F*); **^{19}F{/^{1}H} NMR** (376 MHz, $CDCl_3$) -111.42 (d, *J* = 5.7 Hz, 1F, *F*), -149.62 (d, *J* = 6.1 Hz, 1F, *F*); **HRMS** (ESI) m/z: calcd. for $C_{15}H_{19}F_2NONa^+$ 290.1332 [M+Na]$^+$; found 290.1316.

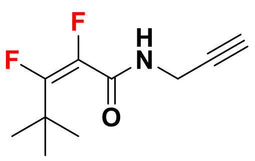

(*Z*)-2,3-Difluoro-4,4-dimethyl-*N*-(prop-2-yn-1-yl)pent-2-enamide (13d)

The pure product was isolated as a colorless oil in 59% yield (39 mg). **^{1}H NMR** (400 MHz, $CDCl_3$) *δ* 6.45 (bs, 1H, N*H*), 4.13 (ddd, *J* = 5.4, 2.6, 0.9 Hz, 2H, NHC*H*$_2$), 2.28 (t, *J* = 2.6 Hz,

1H, CH), 1.34 (d, *J* = 2.8 Hz, 9H, C(C*H*$_3$)); **^{13}C NMR** (101 MHz, CDCl$_3$) *δ* 164.47 (dd, *J* = 274.3, 12.7 Hz, *C*F), 159.45 (dd, *J* = 27.0, 7.2 Hz, *C*O), 140.21 (dd, *J* = 252.1, 25.0 Hz, *C*FCO), 78.59 (*C*CH), 72.06 (C*C*H), 34.85 (dd, *J* = 19.9, 1.7 Hz, NH*C*H$_2$), 28.98, 27.17 (dd, *J* = 6.2, 1.9 Hz); **^{19}F NMR** (376 MHz, CDCl$_3$) -109.93 ÷ 110.01 (m, 1F, *F*), -151.18 (dd, *J* = 6.1, 3.4 Hz, 1F, *F*); **^{19}F{/^{1}H} NMR** (376 MHz, CDCl$_3$) -109.97 (d, *J* = 5.9 Hz, 1F, *F*), -151.17 (d, *J* = 5.6 Hz, 1F, *F*); **HRMS** (ESI) m/z: calcd. for $C_{10}H_{14}F_2NO^+$ 202.1043 [M+H]$^+$; found 202.1043.

1.2.5. General procedure for the synthesis of nonfluorinated unsaturated amides with *tert*-BuLi

To the mixture of corresponding amide in dry THF placed in a round-bottom flask and cooled to −78°C, *tert*-BuLi (4 equiv, 1.7 M in pentane) was added dropwise under an argon atmosphere. The solution was stirred at the −78 °C for 3h. Next, the cooling bath was removed and the reaction mixture was left overnight at room temperature with stirring. Following this, the reaction mixture was carefully quenched by dropwise addition of saturated NH_4Cl aq (10 mL), and then extracted with AcOEt (3 × 15 mL). The combined organic phases were dried (Na_2SO_4), the volatiles were removed using a rotary evaporator and the residue was subjected to careful column chromatography (hexane - 10% EtOAc/hexane) to yield the desired products.

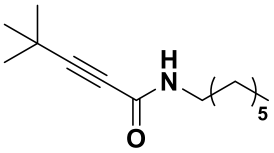

N-Heptyl-4,4-dimethylpent-2-ynamide (14a)

The pure product was isolated as a colorless oil in 70% yield (40 mg). **NMR** (400 MHz, CDCl$_3$) *δ* 5.75 (bs, 1H, N*H*), 3.27 (td, *J* = 7.4, 6.0 Hz, 2H, NHC*H*$_2$), 1.52 (h, *J* = 7.3 Hz, 2H, C*H*$_2$), 1.35-1.29 (m, 8H, 4xC*H*$_2$), 1.26 (s, 9H, C(C*H*$_3$)), 0.88 (t, *J* = 7.0 Hz, 3H, C*H*$_3$); **^{13}C NMR** (101 MHz, CDCl$_3$) *δ* 153.74 (*C*O), 94.19 ($C_{sp}C_{sp}$), 74.21 ($C_{sp}C_{sp}$), 39.87 (NH*C*H$_2$), 31.71, 30.22, 29.36, 28.92, 26.84, 22.58, 14.06; **HRMS** (ESI) m/z: calcd. for $C_{14}H_{26}NO^+$ 224.2014 [M+H]$^+$; found 224.2008; calcd. for $C_{14}H_{25}NONa^+$ 246.1833 [M+Na]$^+$; found 246.1828; calcd. for $C_{14}H_{25}NOK^+$ 262.1573 [M+K]$^+$; found 262.1566.

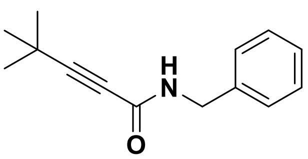

N-Benzyl-4,4-dimethylpent-2-ynamide (14b)

The pure product was isolated as a white solid in 79% yield (44 mg). **^{1}H NMR** (400 MHz, CDCl$_3$) *δ* 7.39-7.25 (m, 5H, Ph), 6.03 (bs, 1H, N*H*), 4.46 (d, *J* = 5.9 Hz, 2H, NHC*H*$_2$), 1.25 (s, 9H, C(C*H*$_3$)$_3$); **^{13}C NMR** (101 MHz, CDCl$_3$) *δ* 153.55 (*C*O), 137.44, 128.75, 127.98, 127.71, 94.98 ($C_{sp}C_{sp}$), 73.91 ($C_{sp}C_{sp}$), 43.83 (NH*C*H$_2$), 30.14, 27.36; **HRMS** (ESI) m/z: calcd. for $C_{14}H_{18}NO^+$

216.1388 [M+H]$^+$; found 216.1379; calcd. for $C_{14}H_{17}NONa^+$ 238.1207 [M+Na]$^+$; found 238.1213.

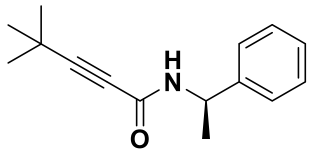

(*R*)-4,4-Dimethyl-N-(1-phenylethyl)pent-2-ynamide (14c)

The pure product was isolated as a white solid in 74% yield (42 mg). **^{1}H NMR** (400 MHz, $CDCl_3$) δ 7.36-7.26 (m, 5H, Ph), 6.04 (bs, 1H, N*H*), 5.23-5.08 (m, 1H, NHC*H*), 1.51 (d, *J* = 6.9 Hz, 3H, C*H*$_3$CH), 1.25 (s, 9H, C(C*H*$_3$)$_3$); **^{13}C NMR** (101 MHz, $CDCl_3$) δ 152.82 (*C*O), 142.44, 128.74, 127.58, 126.34, 94.56 ($\mathit{C}_{sp}C_{sp}$), 74.18 ($C_{sp}\mathit{C}_{sp}$), 49.13 (NH*C*H), 30.12, 27.38, 21.42; **HRMS** (ESI) m/z: calcd. for $C_{15}H_{20}NO^+$ 230.1544 [M+H]$^+$; found 230.1532; calcd. for $C_{15}H_{19}NONa^+$ 252.1364 [M+Na]$^+$; found 252.1355; calcd. for $C_{15}H_{19}NOK^+$ 268.1103 [M+K]$^+$; found 268.1088.

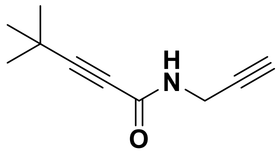

4,4-Dimethyl-*N*-(prop-2-yn-1-yl)pent-2-ynamide (14d)

The pure product was isolated as a white solid in 62% yield (31 mg). **^{1}H NMR** (400 MHz, $CDCl_3$) δ 5.94 (bs, 1H, N*H*), 4.08 (dd, *J* = 5.4, 2.6 Hz, 2H, NHC*H*$_2$), 2.26 (t, *J* = 2.6 Hz, 1H, C*H*), 1.26 (s, 9H, C(C*H*$_3$)$_3$); **^{13}C NMR** (101 MHz, $CDCl_3$) δ 153.21 (C*O*), 95.68 ($\mathit{C}_{sp}C_{sp}$), 78.70 (*C*CH), 73.49 ($C_{sp}\mathit{C}_{sp}$), 72.04 (C*C*H), 30.12, 29.42 (NH*C*H$_2$), 27.43; **HRMS** (ESI) m/z: calcd. for $C_{10}H_{14}NO^+$ 164.1075 [M+H]$^+$; found 164.1063; calcd. for $C_{10}H_{13}NONa^+$ 186.0894 [M+Na]$^+$; found 186.0893.

1.2.6. Synthesis of *N*-methylation products

To the mixture of corresponding amide in dry THF placed in a round-bottom flask and cooled to −78°C, *tert*-BuLi (8 equiv, 1.7 M in pentane) was added dropwise under an argon atmosphere. The solution was stirred at the −78 °C for 3 h. Further, methyl iodide (2 equiv) was dropped into the reaction mixture. Next, the cooling bath was removed and the reaction mixture was left overnight at room temperature with stirring. Following this, the reaction mixture was carefully quenched by dropwise addition of saturated NH_4Cl aq (10 mL), and then extracted with AcOEt (3 × 15 mL). The combined organic phases were dried (Na_2SO_4), the volatiles were removed using a rotary evaporator and the residue was subjected to careful column chromatography (hexane - 2% EtOAc/hexane) to yield the desired products.

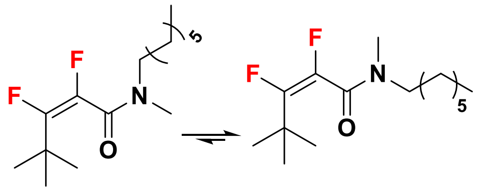

15a **15a'**

(Z)-2,3-Difluoro-N-heptyl-N,4,4-trimethylpent-2-enamide (15a (15a'))

The pure product was isolated as a colorless oil in 96% yield (40mg).

Minor - 15a: **^{1}H NMR** (400 MHz, $CDCl_3$) δ 3.33 (dt, J = 7.6, 1.9 Hz, 2H, NCH_3CH_2), 2.96 (d, J = 1.8 Hz, 3H, NCH_3), 1.68-1.49 (m, 2H, CH_2), 1.37-1.23 (m, 8H, 4xCH_2), 1.18 (s, 9H, $C(CH_3)$), 0.89 (t, J = 6.8 Hz, 3H, CH_3); **^{13}C NMR** (101 MHz, $CDCl_3$) δ 161.66 (dd, J = 26.9, 5.3 Hz, *C*O), 155.82 (dd, J = 258.9, 12.2 Hz, *C*F), 138.47 (dd, J = 252.6, 20.2 Hz, *C*FCO), 50.94 (NCH_2), 35.91, 33.59 (dd, J = 21.0, 3.5 Hz, *C*CF), 32.25, 31.64, 28.91, 28.21, 26.82 (dd, J = 5.9, 2.0 Hz), 26.47, 22.53, 14.03; **^{19}F NMR** (376 MHz, $CDCl_3$) δ -134.82 (d, J = 8.9 Hz, 1F, *F*), -142.85 (d, J = 9.0 Hz, 1F, *F*), **^{19}F{/^{1}H} NMR** (376 MHz, $CDCl_3$) δ -134.82 (d, J = 9.3 Hz, 1F, *F*), -142.85 (d, J = 9.0 Hz, 1F, *F*),

Major - 15a': **^{1}H NMR** (400 MHz, $CDCl_3$) δ 3.41-3.36 (m, 2H, NCH_3CH_2), 3.05 (d, J = 2.1 Hz, 3H, NCH_3), 1.68-1.49 (m, 2H, CH_2), 1.37-1.23 (m, 8H, 4xCH_2), 1.18 (s, 9H, $C(CH_3)$), 0.88 (t, J = 6.8 Hz, 3H, CH_3); **^{13}C NMR** (101 MHz, $CDCl_3$) δ 161.59 (dd, J = 26.9, 5.3 Hz, *C*O), 155.80 (dd, J = 259.3, 12.2 Hz, *C*F), 138.27 (dd, J = 252.3, 20.4 Hz, *C*FCO), 47.24 (NCH_2), 35.89, 33.58 (dd, J = 21.3, 3.4 Hz, *C*CF), 32.25, 31.72, 28.98, 28.21, 26.84 (d, J = 7.4, 1.8 Hz), 26.58, 22.54, 14.01; **^{19}F NMR** (376 MHz, $CDCl_3$) δ -135.24 (d, J = 8.7 Hz, 1F, *F*), -144.58 (d, J = 8.8 Hz, 1F, *F*); **^{19}F{/^{1}H} NMR** (376 MHz, $CDCl_3$) δ -135.24 (d, J = 8.8 Hz, 1F, *F*), -144.59 (d, J = 9.0 Hz, 1F, *F*);

HRMS (ESI) m/z: calcd. for $C_{15}H_{28}F_2NO^+$ 276.2138 $[M+H]^+$; found 276.2132, calcd. for $C_{15}H_{27}F_2NONa^+$ 298.1958 $[M+Na]^+$; found 298.1954, calcd. for $C_{15}H_{27}F_2NOK^+$ 314.1697 $[M+K]^+$; found 314.1690.

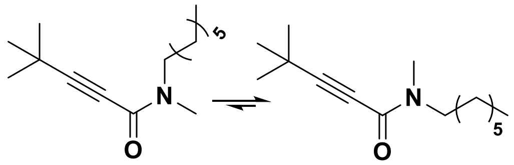

16a **16a'**

N-Heptyl-N,4,4-trimethylpent-2-ynamide (16a (16a'))

The pure product was isolated as a colorless oil in 83% yield (44 mg).

Minor - 16a: **^{1}H NMR** (400 MHz, $CDCl_3$) δ 3.37 (t, J = 7.2 Hz, 2H, $NHCH_2$), 3.14 (s, 3H, NCH_3), 1.63-1.49 (m, 4H, 2xCH_2), 1.34-1.27 (m, 6H, 3xCH_2), 1.26 (s, 9H, $C(CH_3)$), 0.93-0.83 (m, 3H,

CH_3); **^{13}C NMR** (101 MHz, $CDCl_3$) δ 153.72 (CO), 94.20 ($C_{sp}C_{sp}$), 74.21 ($C_{sp}C_{sp}$), 46.56 (NCH_2), 36.34, 31.75, 31.72, 30.23, 29.08, 28.93, 27.71, 26.96, 26.77, 22.59, 14.06;

Major - 16a’: ^{1}H NMR (400 MHz, $CDCl_3$) δ 3.52 (t, J = 7.2 Hz, 2H, $NHCH_2$), 2.93 (s, 3H, NCH_3), 1.63-1.49 (m, 4H, 2xCH_2), 1.34-1.27 (m, 6H, 3xCH_2), 1.28 (s, 9H, $C(CH_3)$), 0.93-0.83 (m, 3H, CH_3); **^{13}C NMR** (101 MHz, $CDCl_3$) δ 154.85 (CO), 99.39 ($C_{sp}C_{sp}$), 72.80 ($C_{sp}C_{sp}$), 51.27 (NCH_2), 39.87, 32.19, 31.78, 30.21, 29.37, 29.01, 28.23, 26.84, 26.61, 22.56, 14.06;

HRMS (ESI) m/z: calcd. for $C_{15}H_{28}NO^+$ 238.2170 $[M+H]^+$; found 238.2158; calcd. for $C_{15}H_{27}NONa^+$ 260.1990 $[M+Na]^+$; found 260.1981; calcd. for $C_{14}H_{25}NOK^+$ 276.1729 $[M+K]^+$; found 276.1715.

2. Copies of NMR spectra

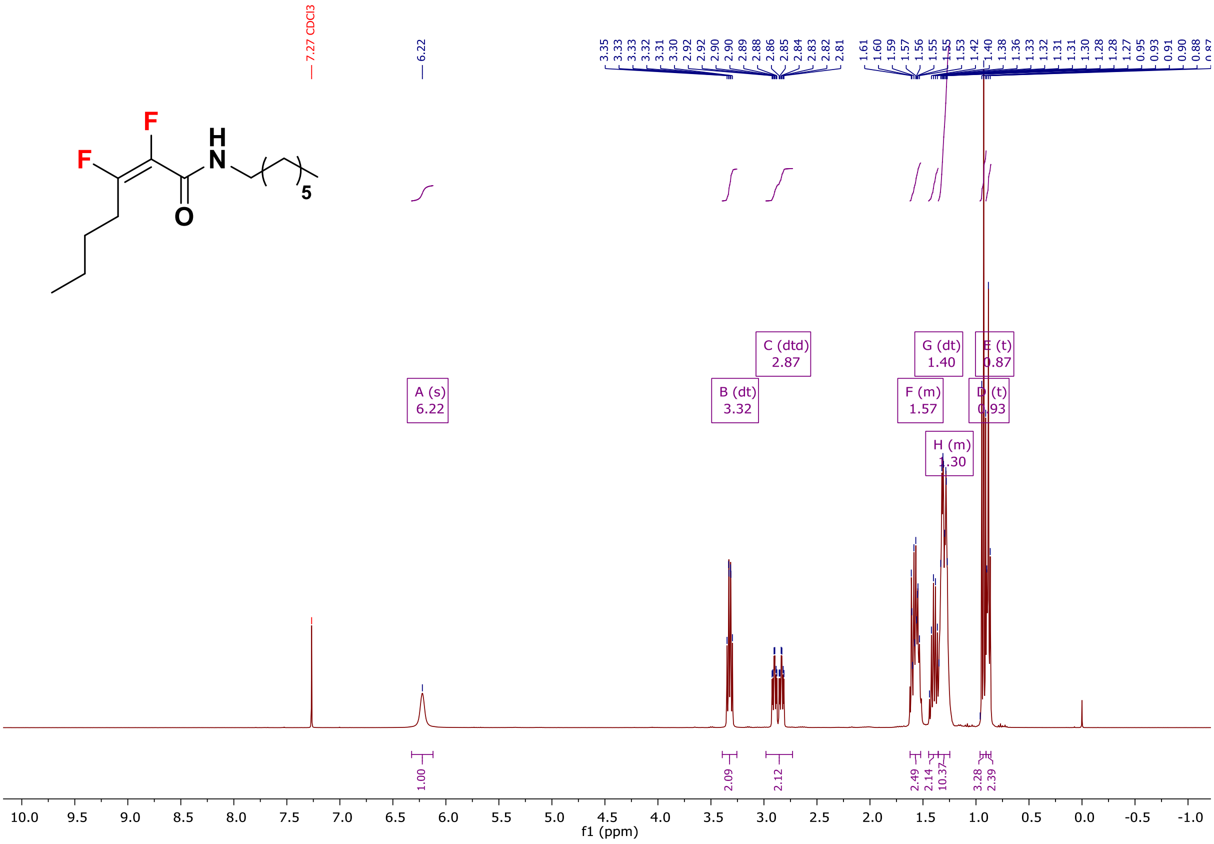

^{1}**H NMR** of (Z)-2,3-difluoro-N-heptylhept-2-enamide **9a**

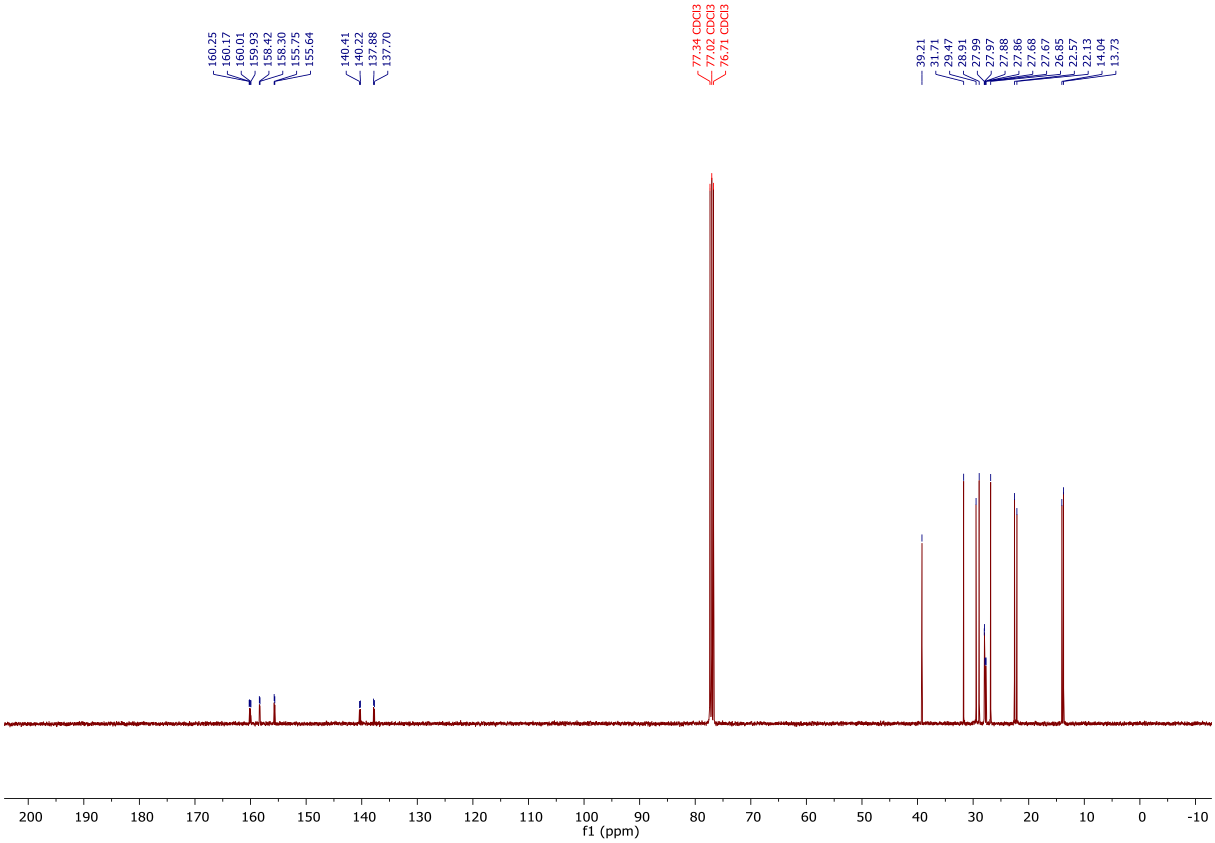

^{13}**C NMR** of (*Z*)-2,3-difluoro-*N*-heptylhept-2-enamide **9a**

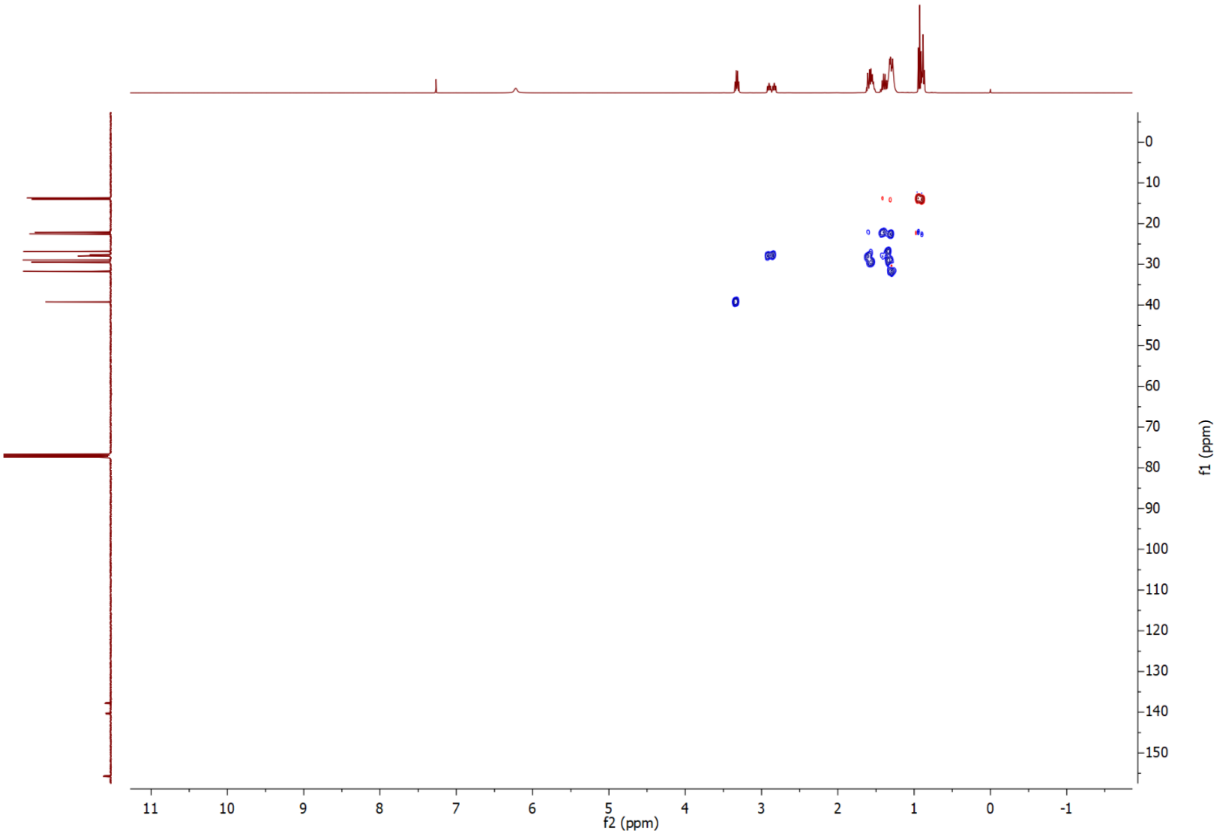

^{1}**H-**^{13}**C HSQC** of (*Z*)-2,3-difluoro-*N*-heptylhept-2-enamide **9a**

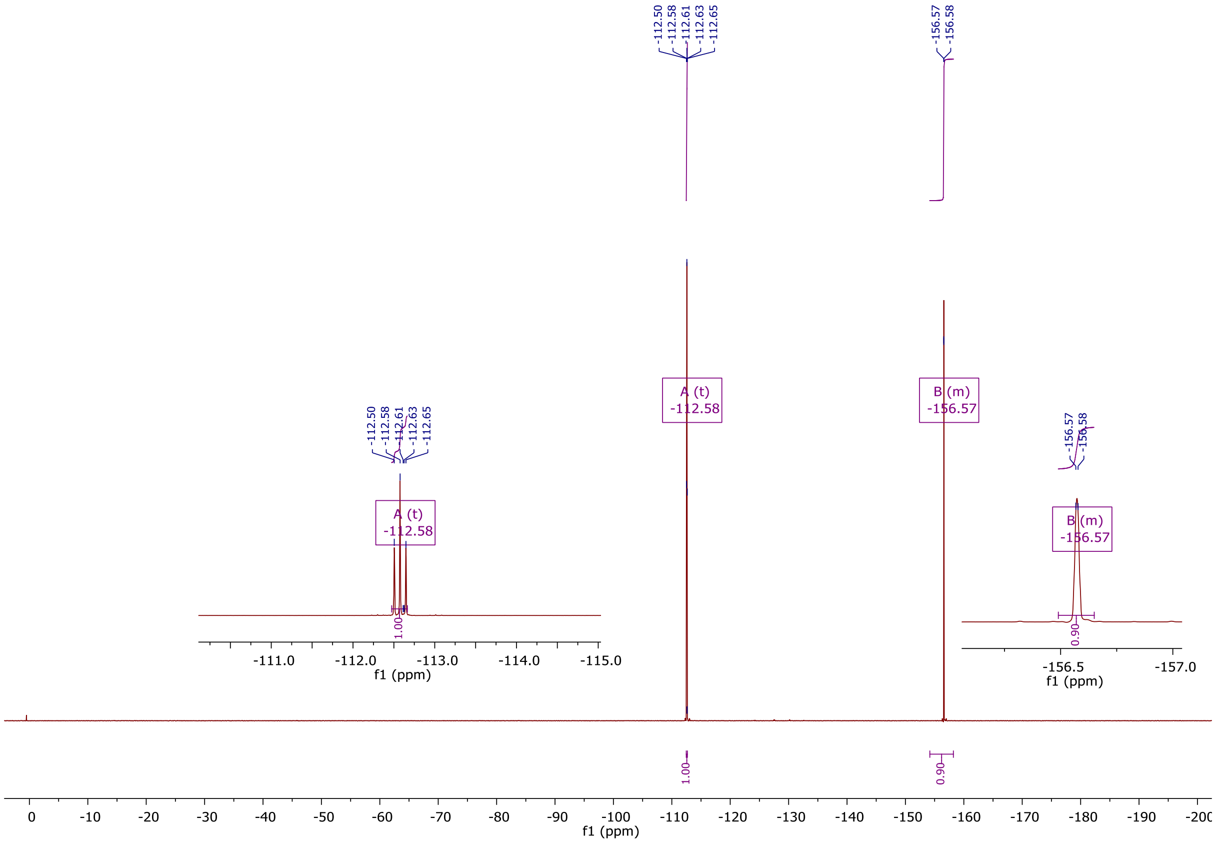

^{19}F NMR of (*Z*)-2,3-difluoro-*N*-heptylhept-2-enamide **9a**

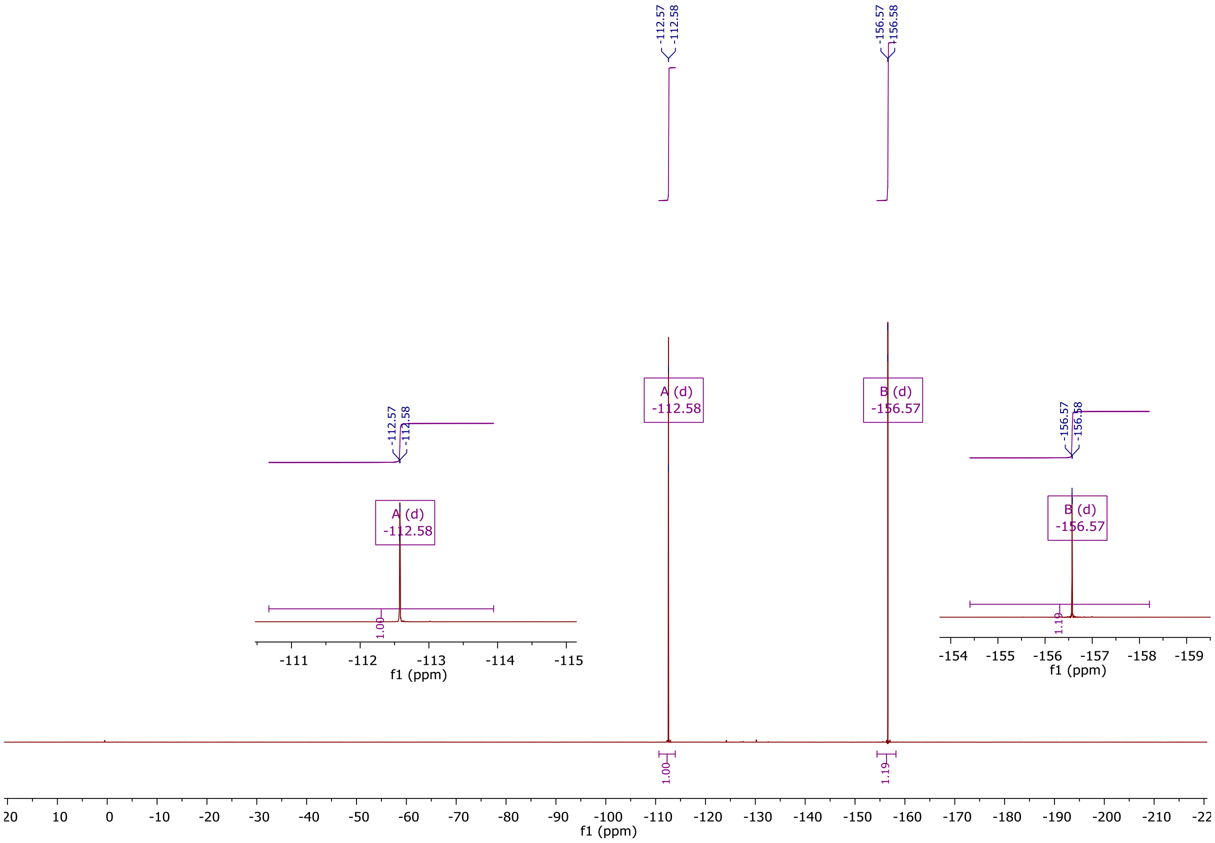

^{19}F{/^{1}H} NMR of (*Z*)-2,3-difluoro-*N*-heptylhept-2-enamide **9a**

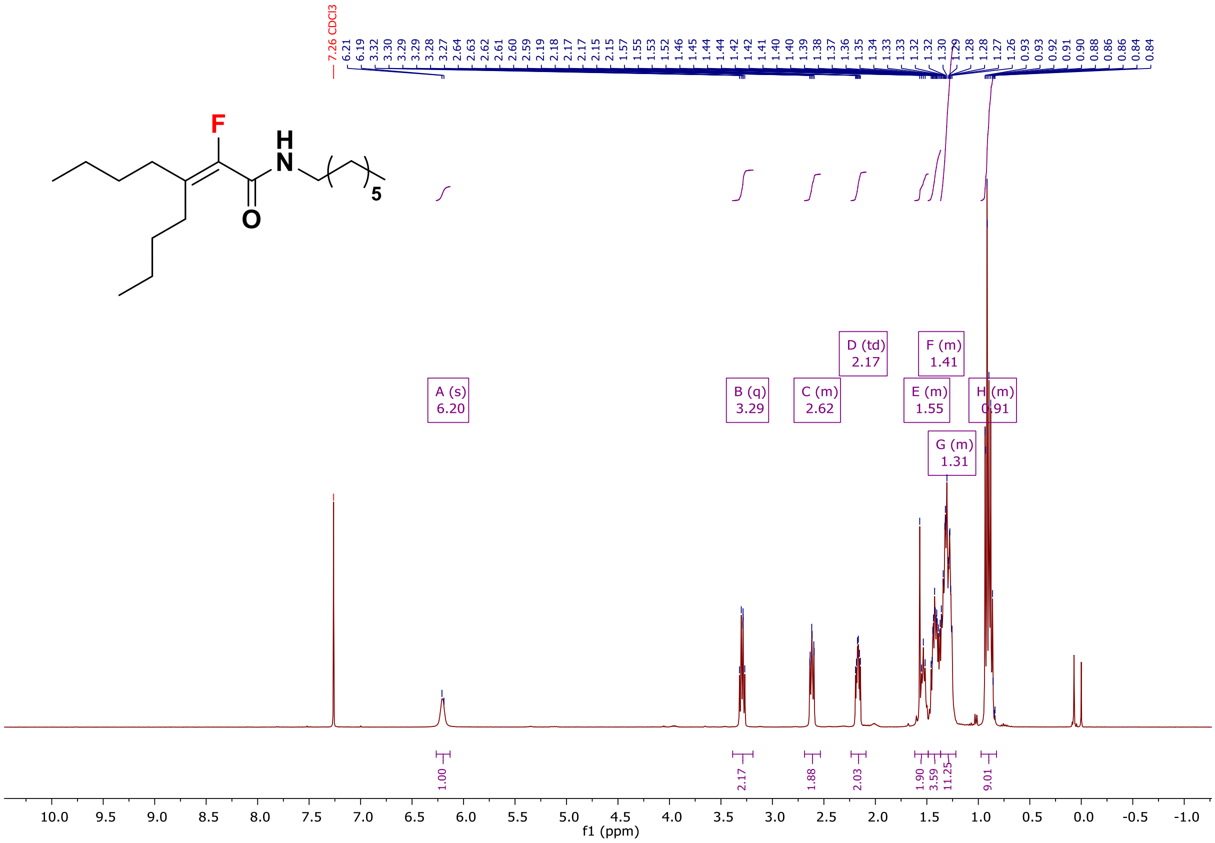

^{1}**H NMR** of 3-butyl-2-fluoro-*N*-heptylhept-2-enamide **10a**

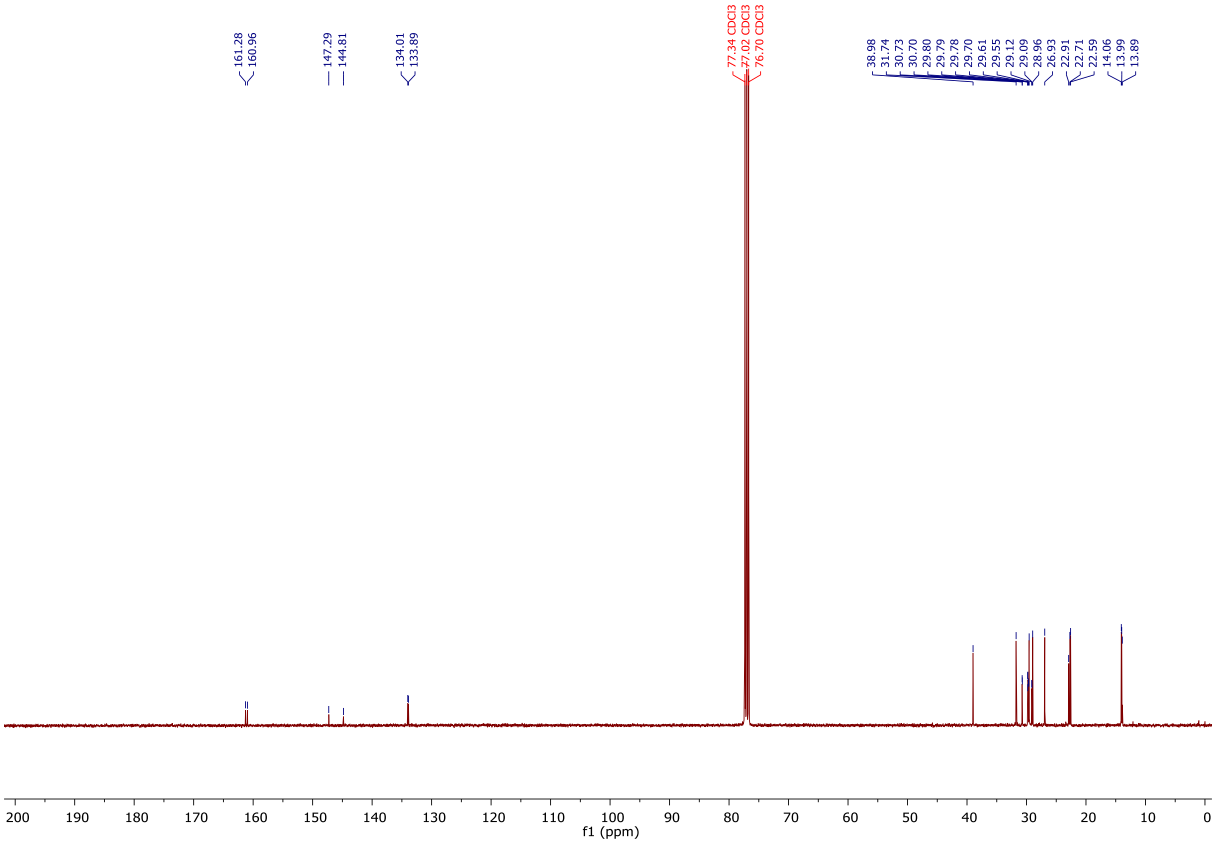

^{13}**C NMR** of 3-butyl-2-fluoro-*N*-heptylhept-2-enamide **10a**

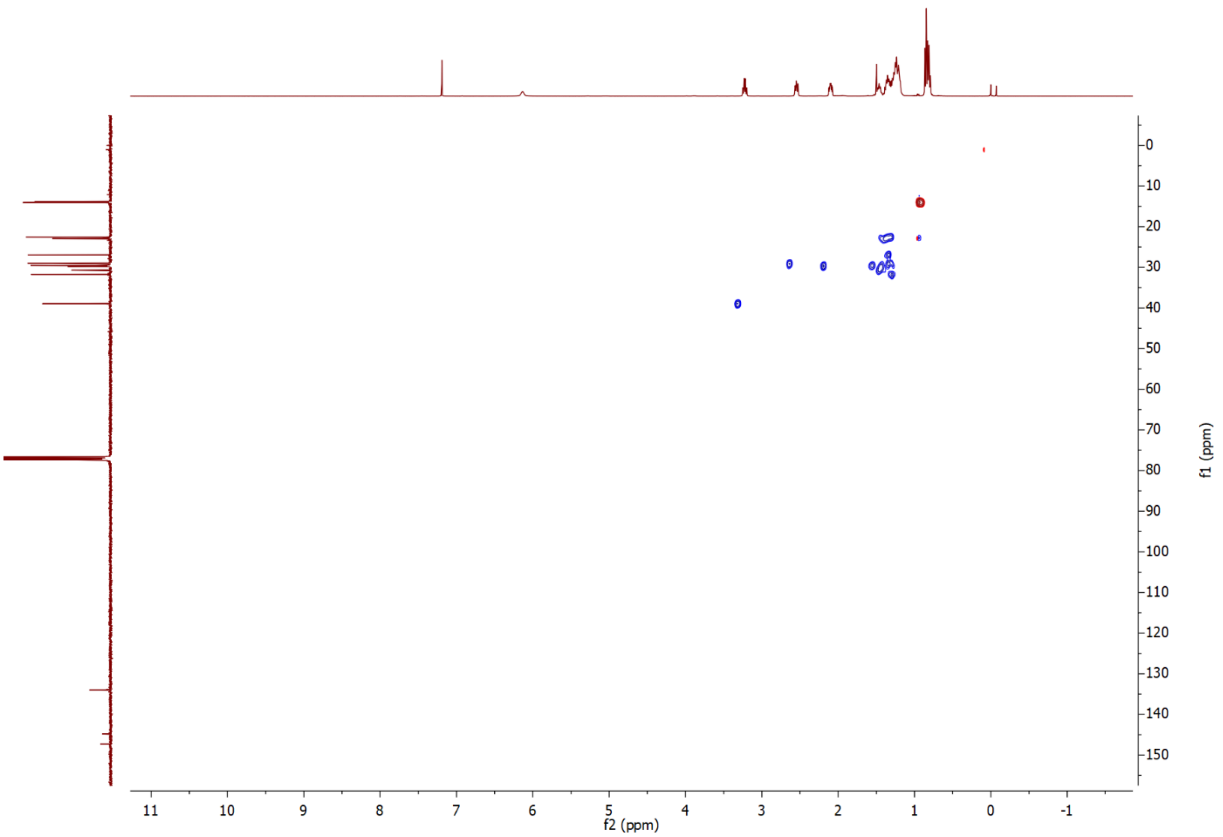

^{1}H-^{13}C **HSQC** of 3-butyl-2-fluoro-*N*-heptylhept-2-enamide **10a**

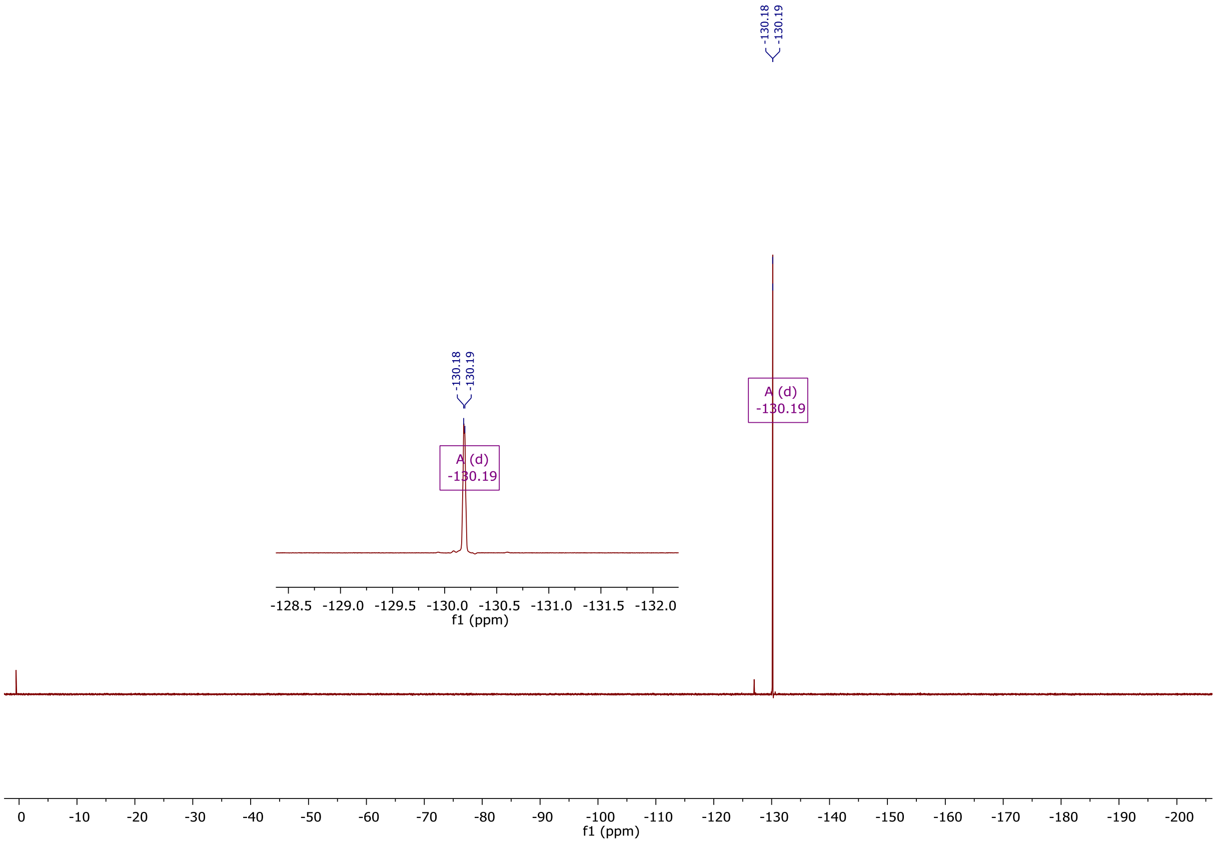

^{19}F **NMR** of 3-butyl-2-fluoro-*N*-heptylhept-2-enamide **10a**

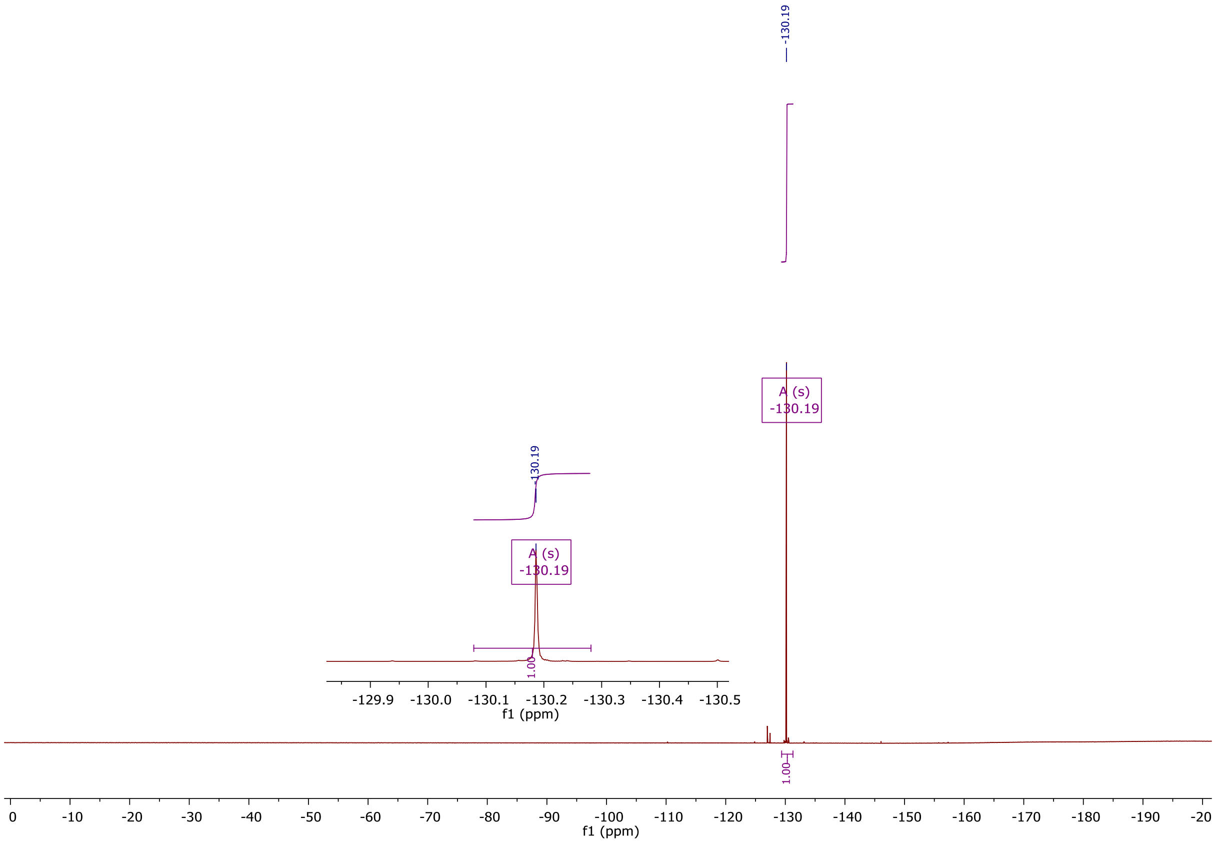

^{19}F{/^{1}H} NMR of 3-butyl-2-fluoro-*N*-heptylhept-2-enamide **10a**

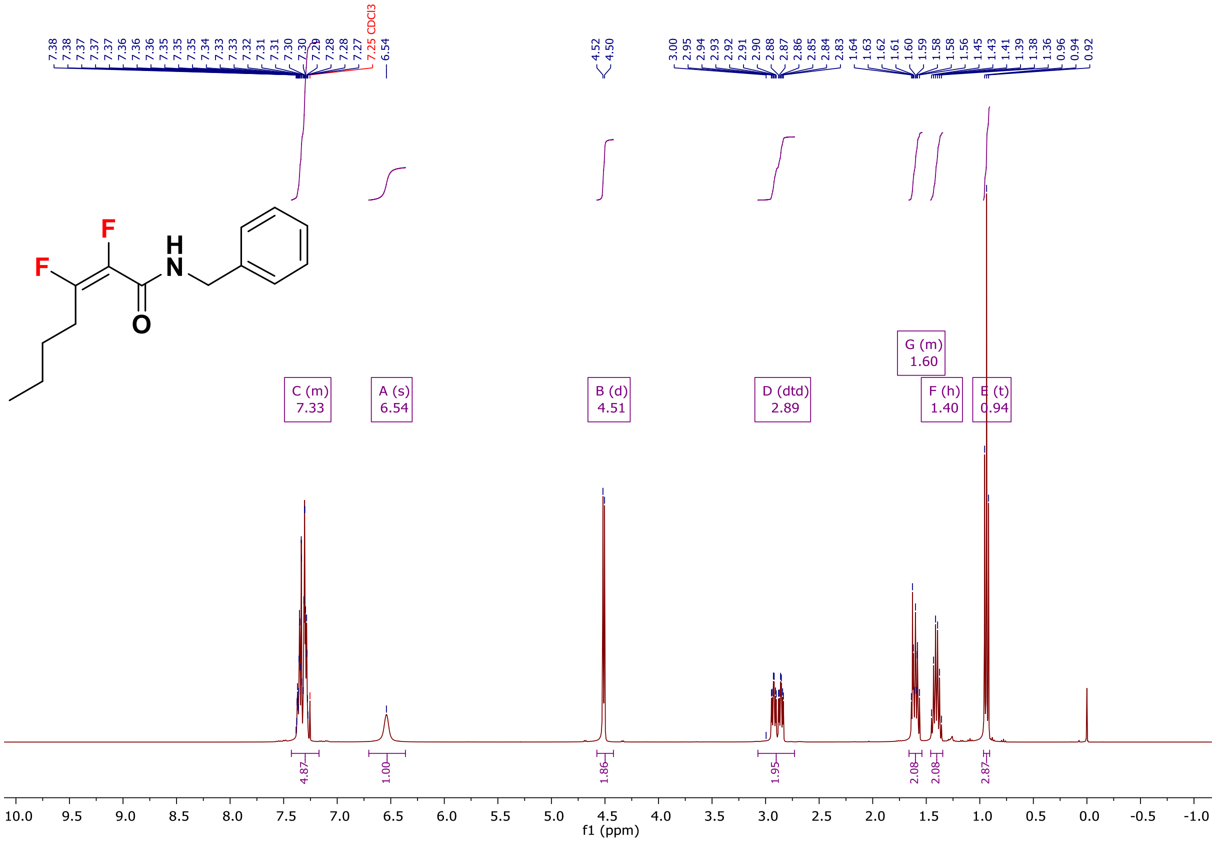

^{1}H NMR of (*Z*)-*N*-benzyl-2,3-difluorohept-2-enamide **9b**

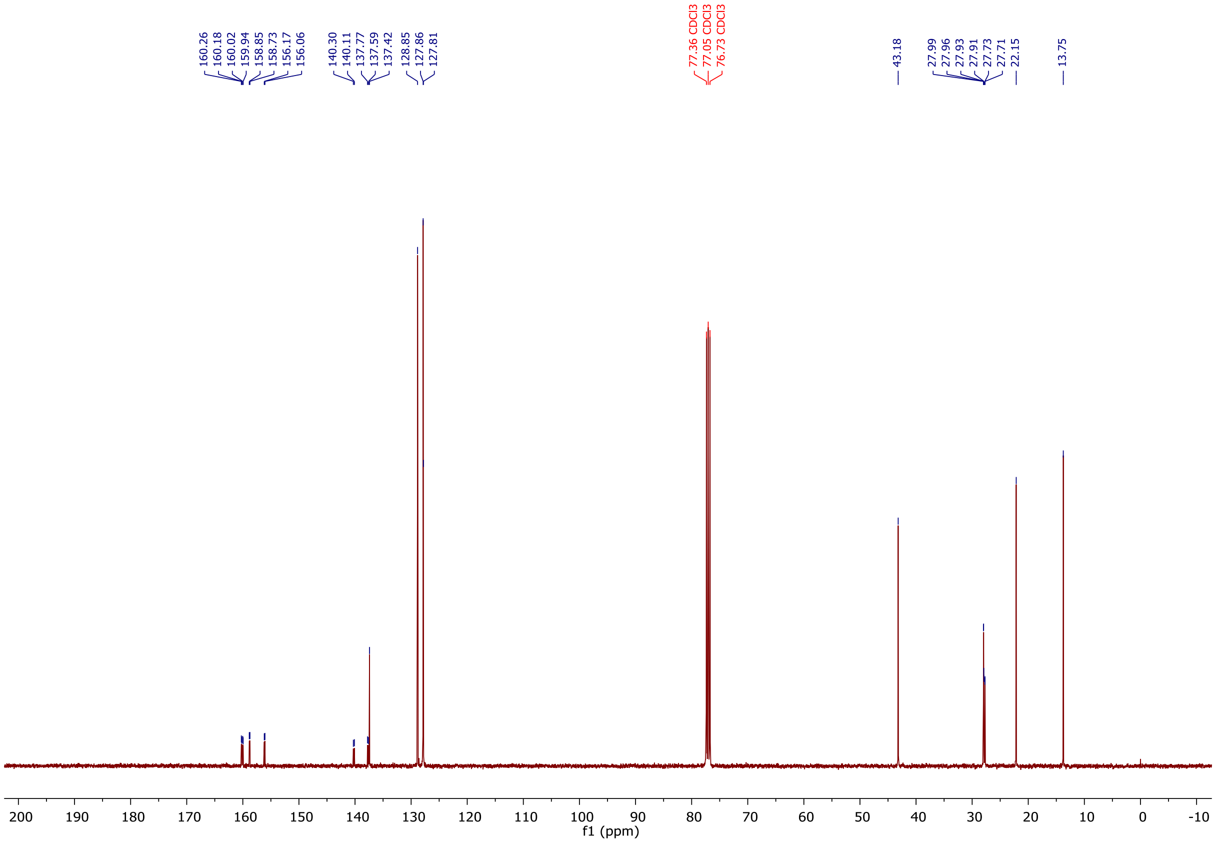

^{13}C NMR of (*Z*)-*N*-benzyl-2,3-difluorohept-2-enamide **9b**

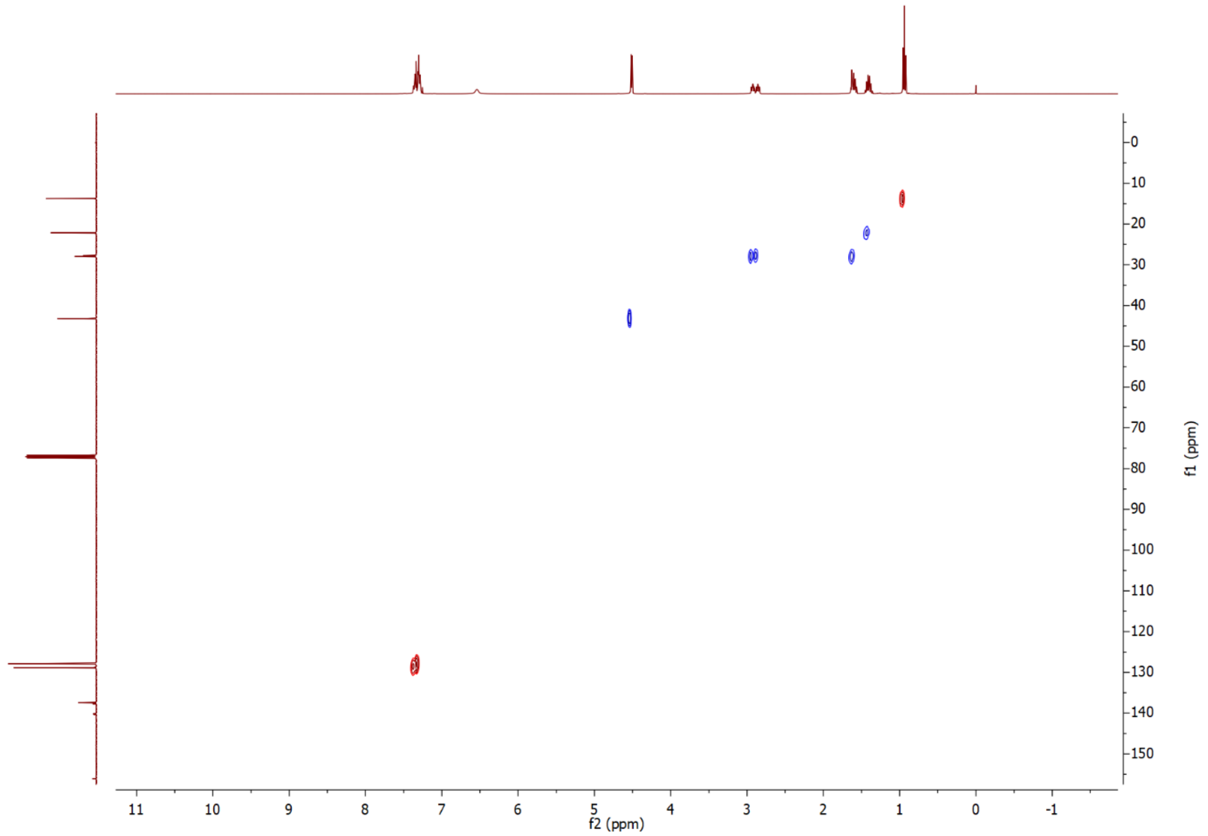

^{1}H-^{13}C HSQC of (*Z*)-*N*-benzyl-2,3-difluorohept-2-enamide **9b**

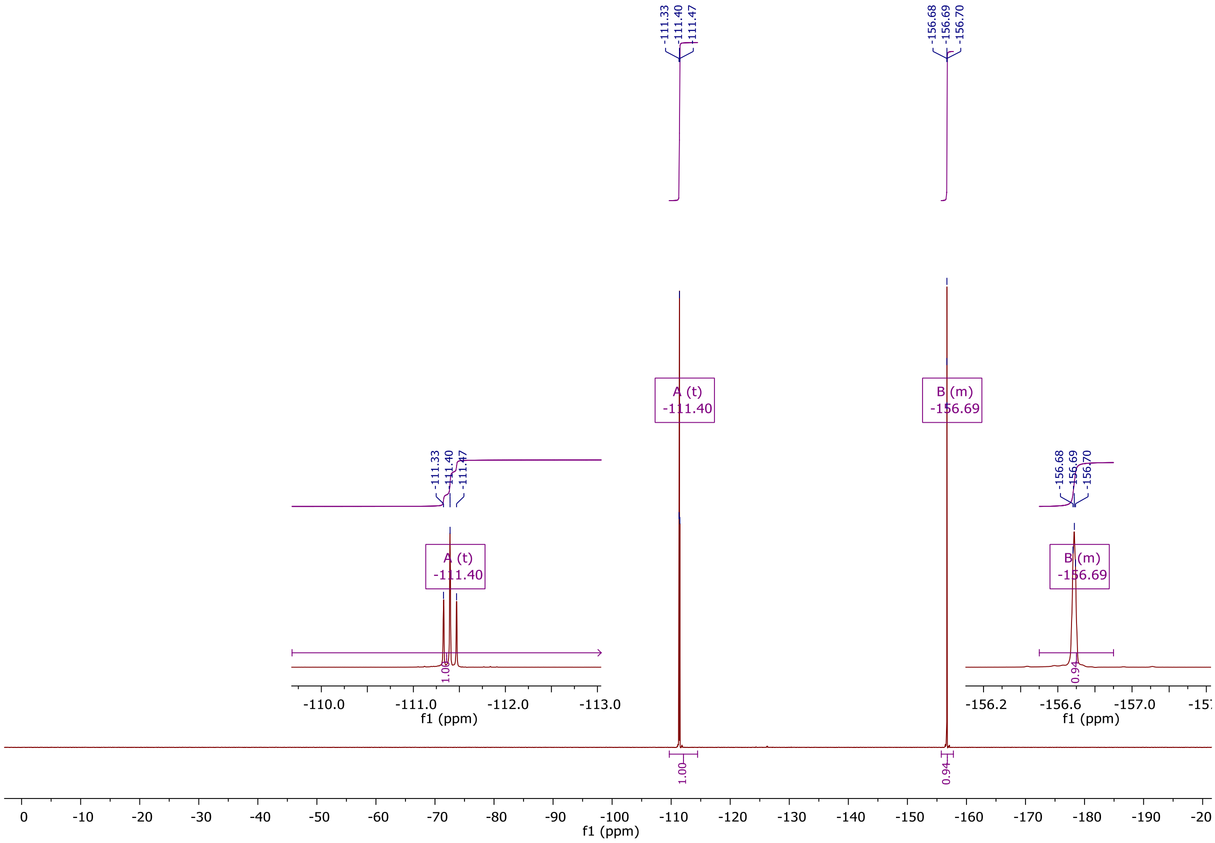

^{19}F NMR of (*Z*)-*N*-benzyl-2,3-difluorohept-2-enamide **9b**

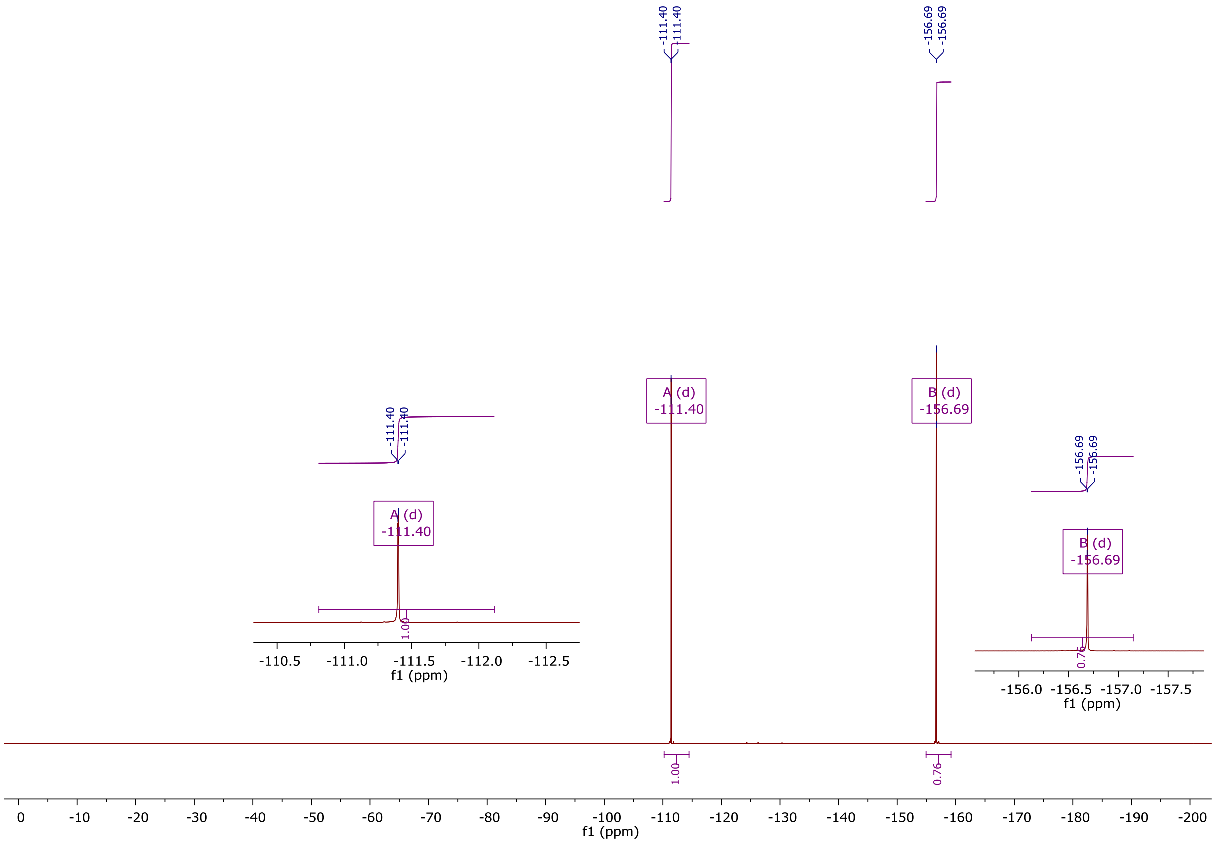

^{19}F{^{1}H} NMR of (*Z*)-*N*-benzyl-2,3-difluorohept-2-enamide **9b**

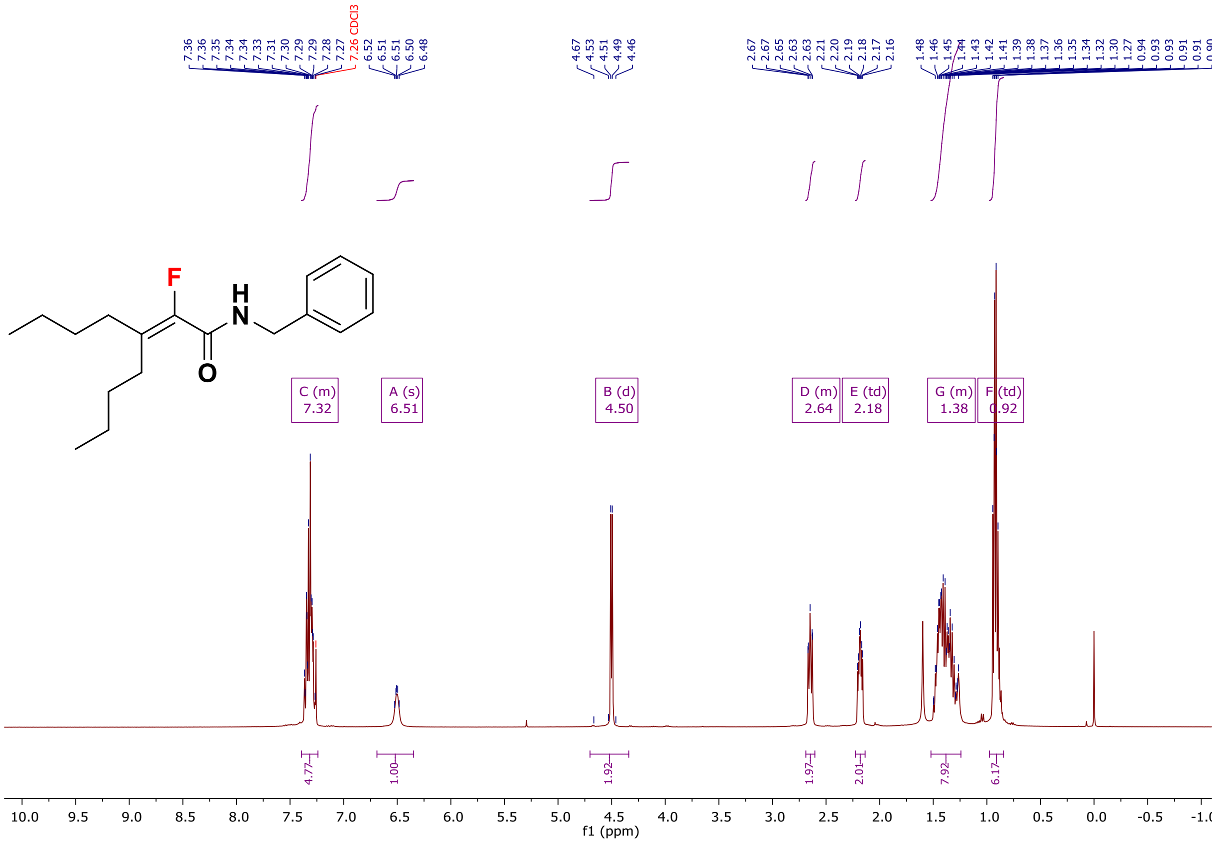

^{1}**H NMR** of *N*-benzyl-3-butyl-2-fluorohept-2-enamide **10b**

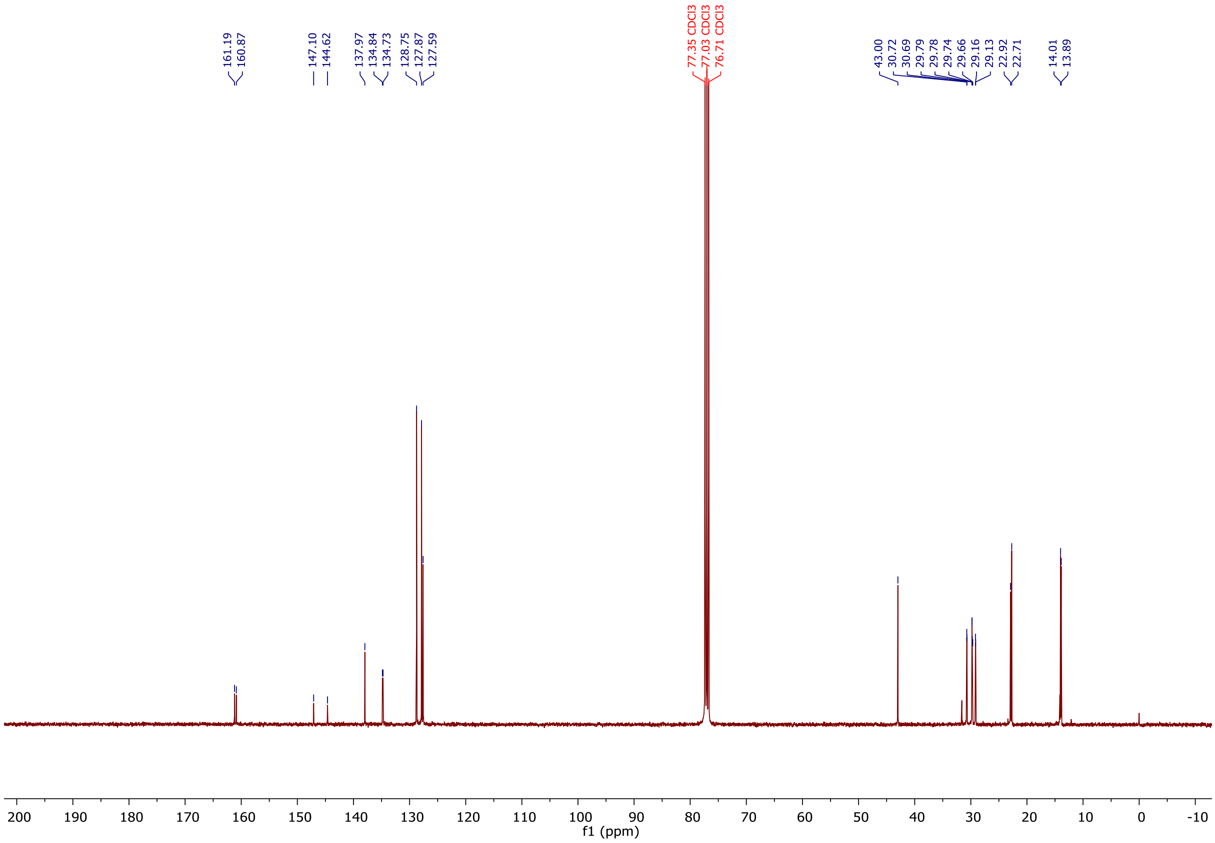

^{13}**C NMR** of *N*-benzyl-3-butyl-2-fluorohept-2-enamide **10b**

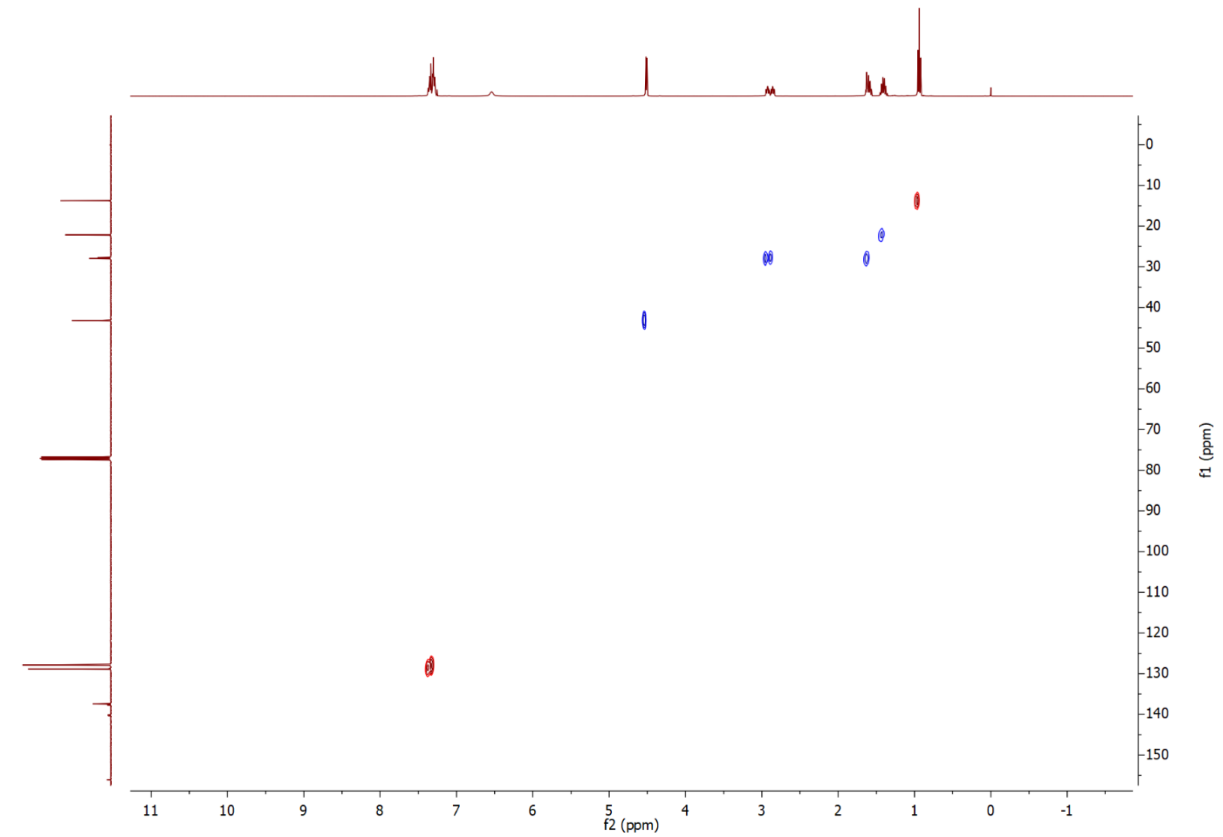

^{1}H-^{13}C **HSQC** of *N*-benzyl-3-butyl-2-fluorohept-2-enamide **10b**

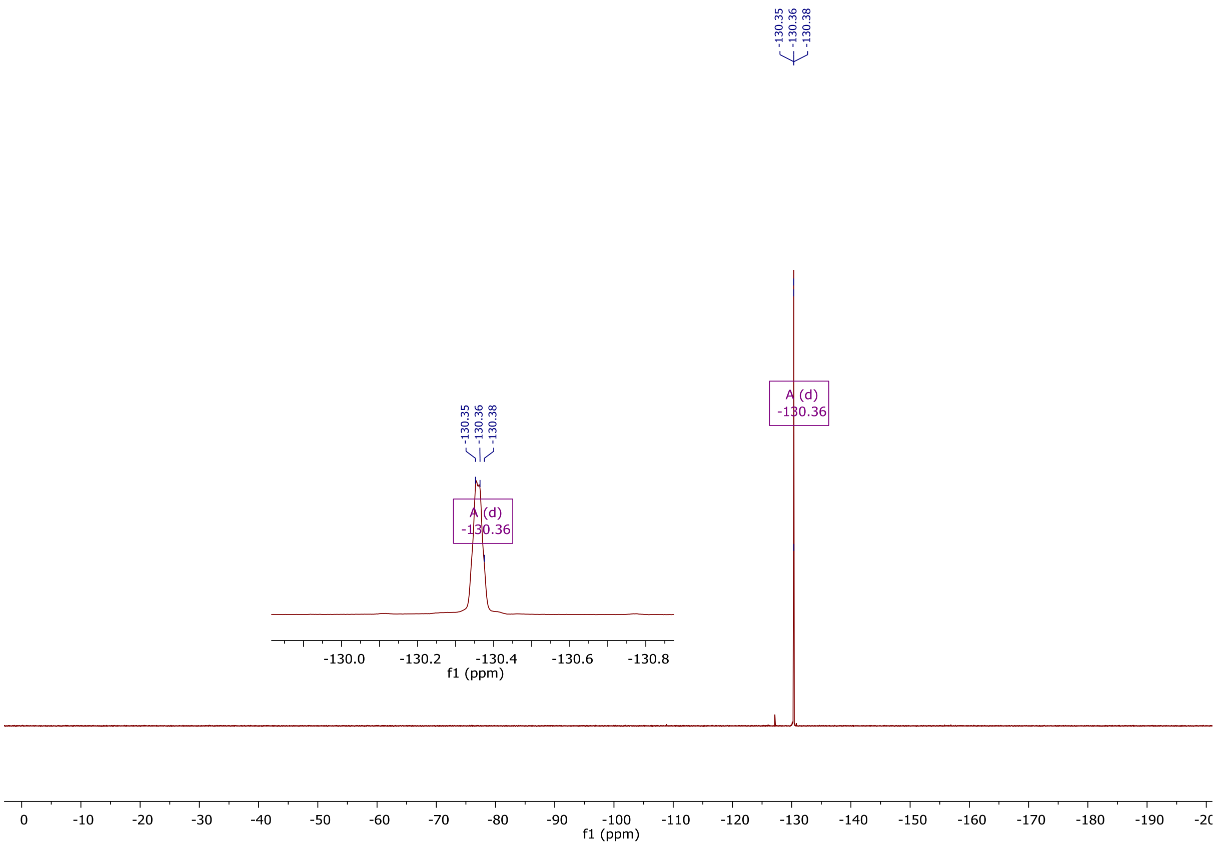

[19]**F NMR** of *N*-benzyl-3-butyl-2-fluorohept-2-enamide **10b**

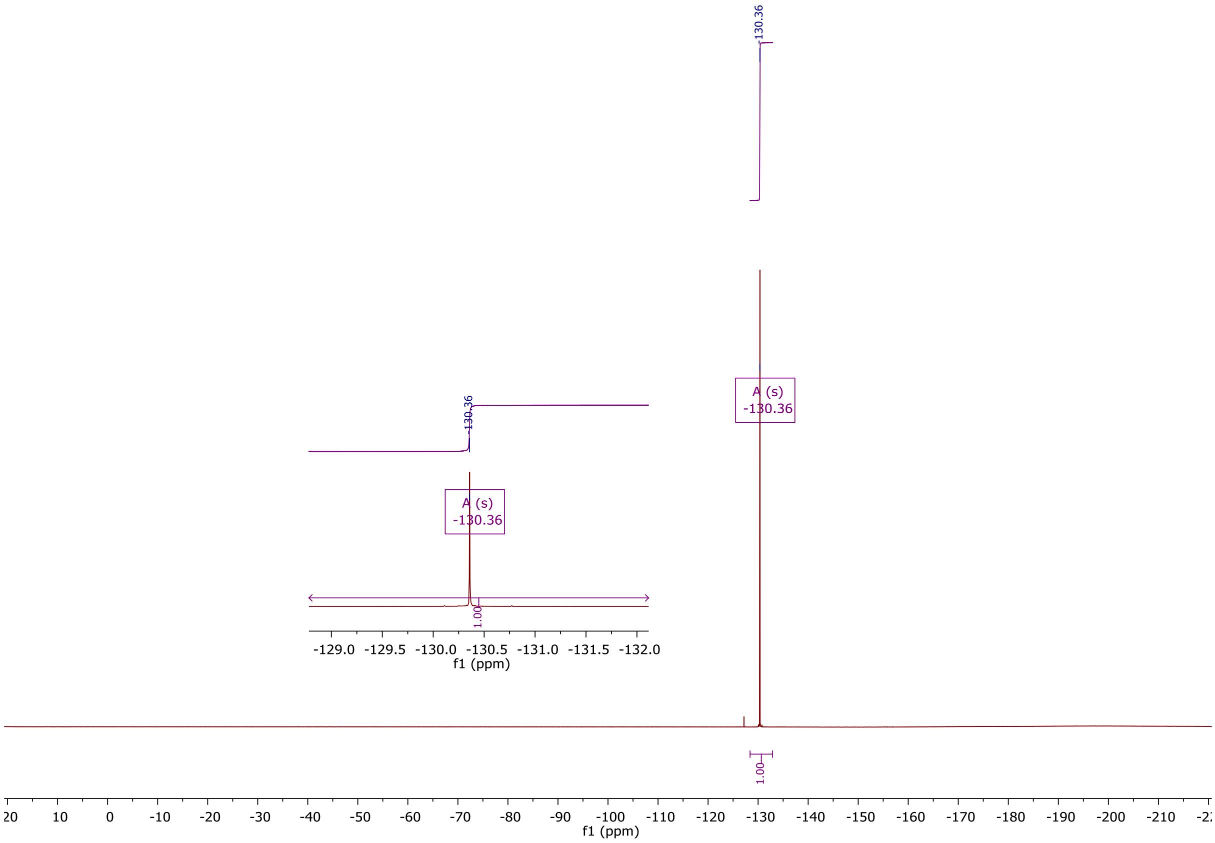

^{19}**F{/^{1}H} NMR** of *N*-benzyl-3-butyl-2-fluorohept-2-enamide **10b**

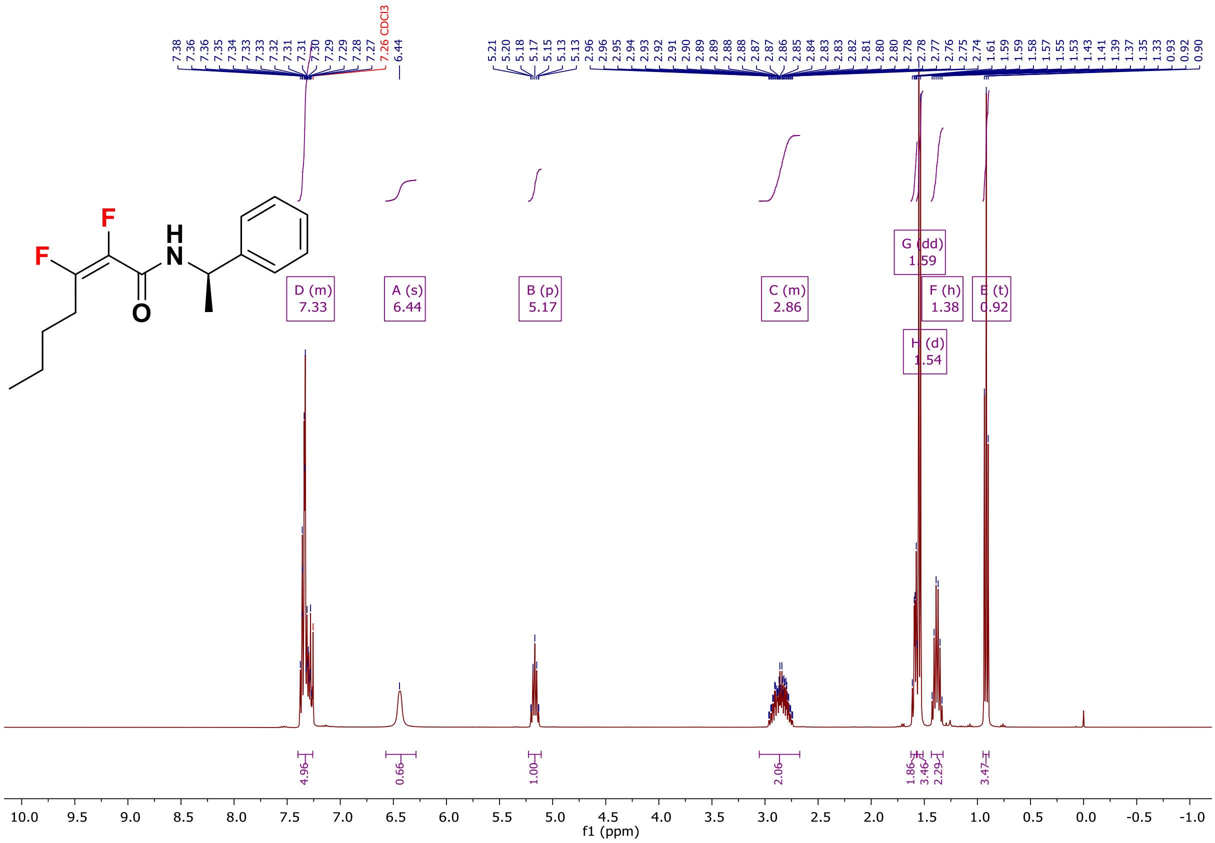

^{1}**H NMR** of (*R*,*Z*)-2,3-difluoro-*N*-(1-phenylethyl)hept-2-enamide **9c**

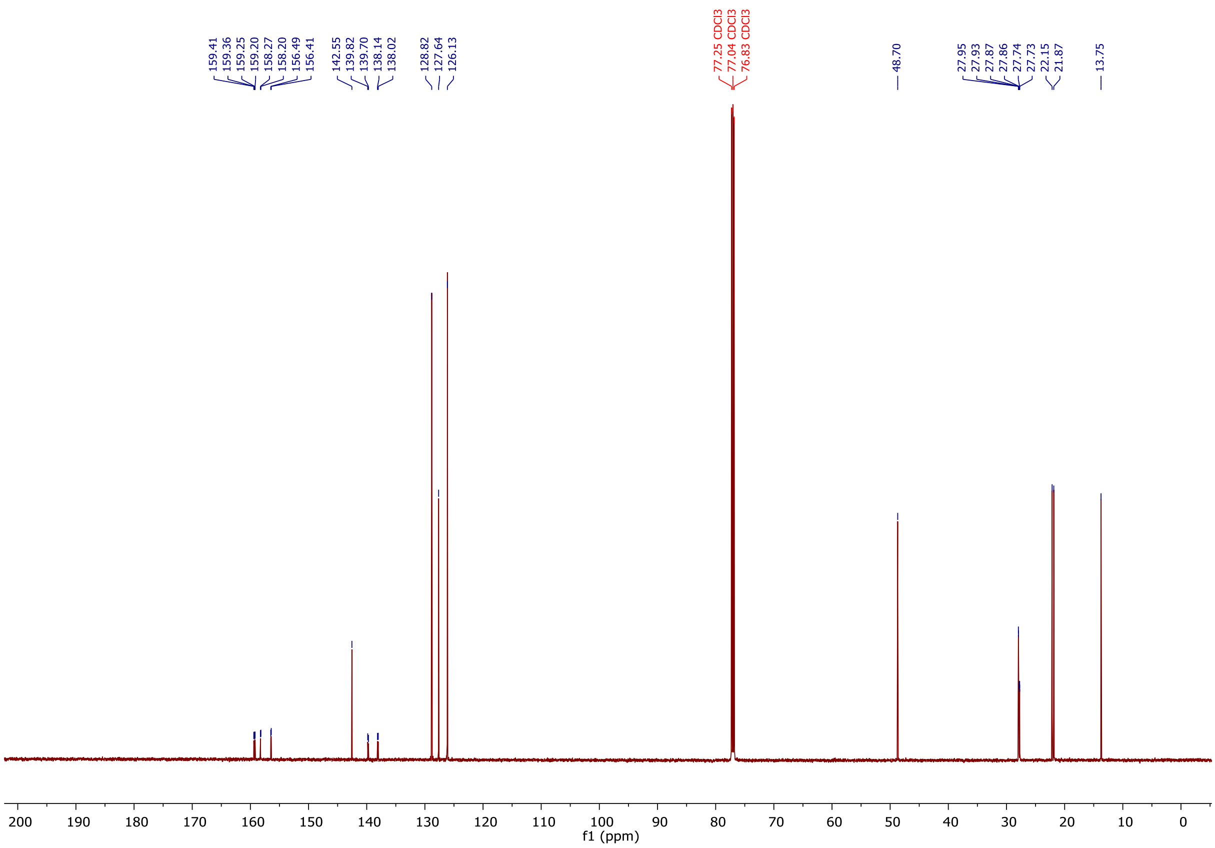

^{13}**C NMR** of (*R*,*Z*)-2,3-difluoro-*N*-(1-phenylethyl)hept-2-enamide **9c**

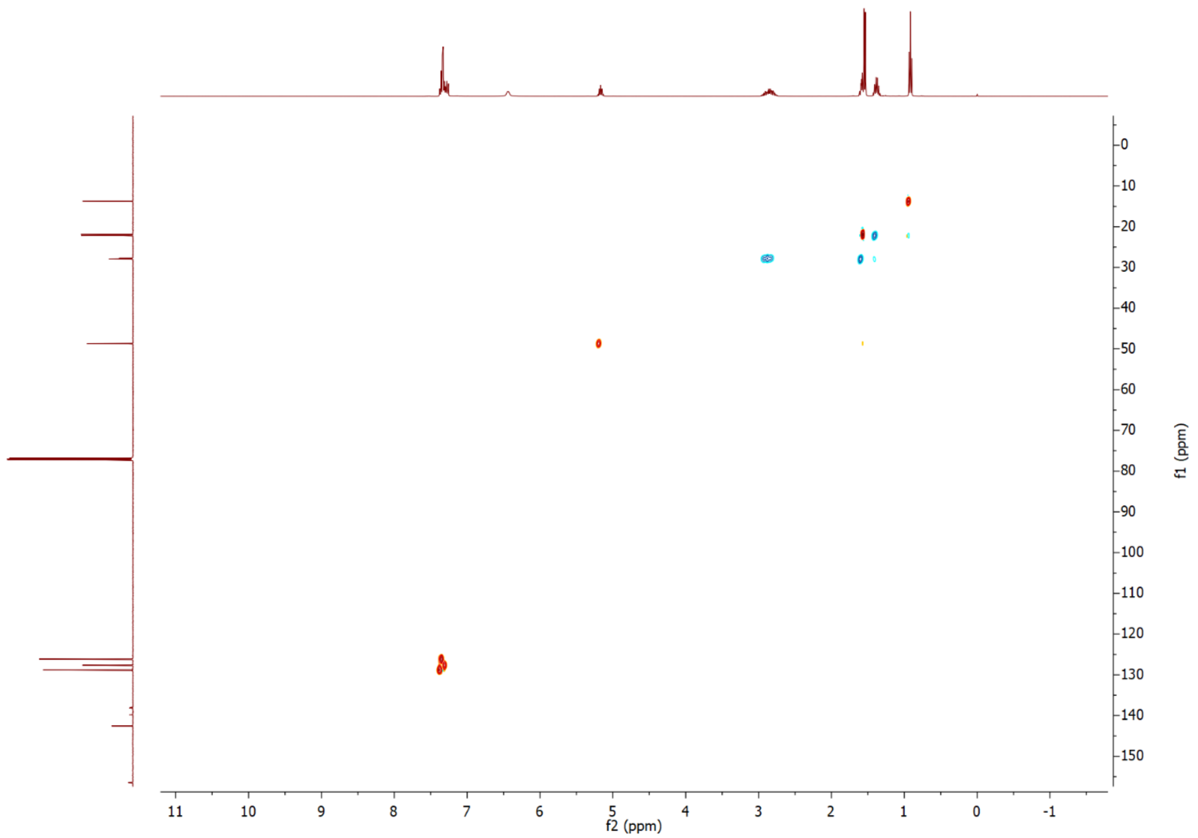

^{1}**H-**^{13}**C HSQC** of (*R*,*Z*)-2,3-difluoro-*N*-(1-phenylethyl)hept-2-enamide **9c**

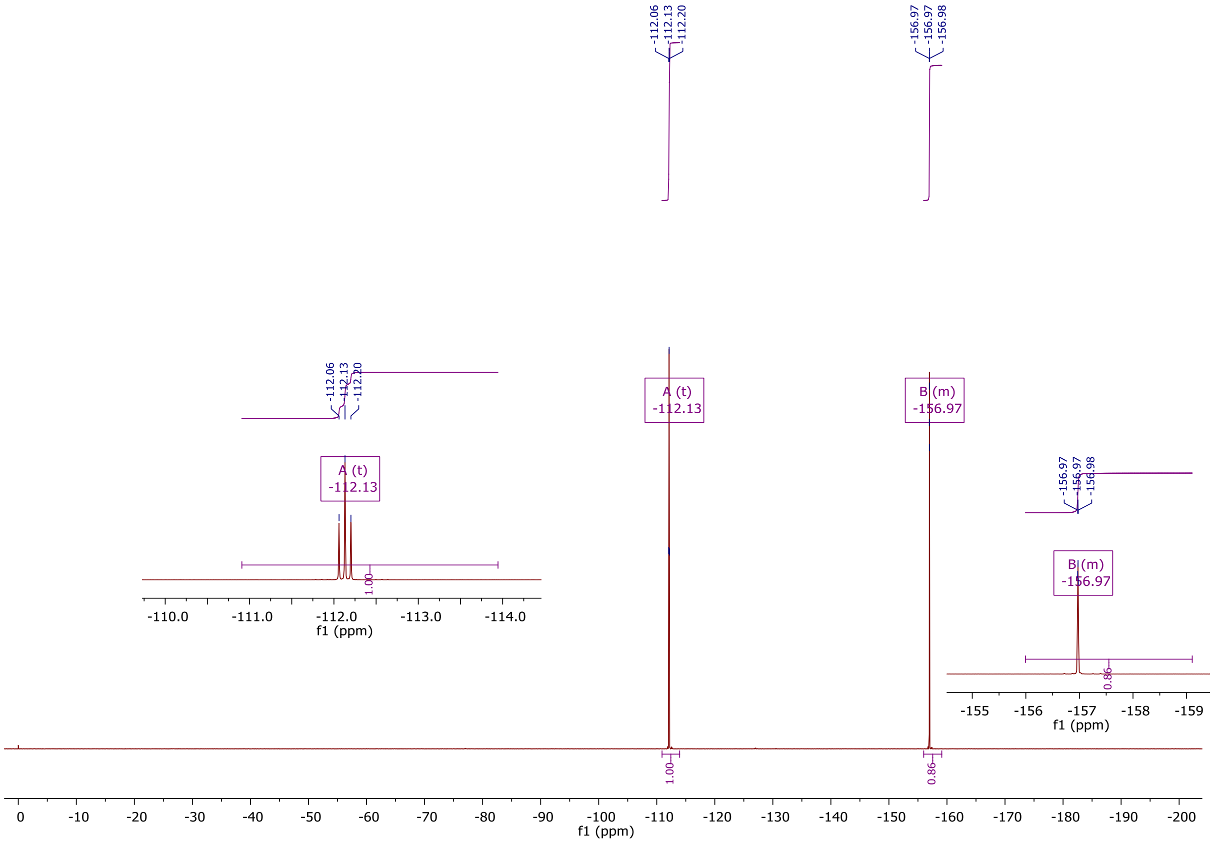

^{19}F NMR of (*R*,*Z*)-2,3-difluoro-*N*-(1-phenylethyl)hept-2-enamide **9c**

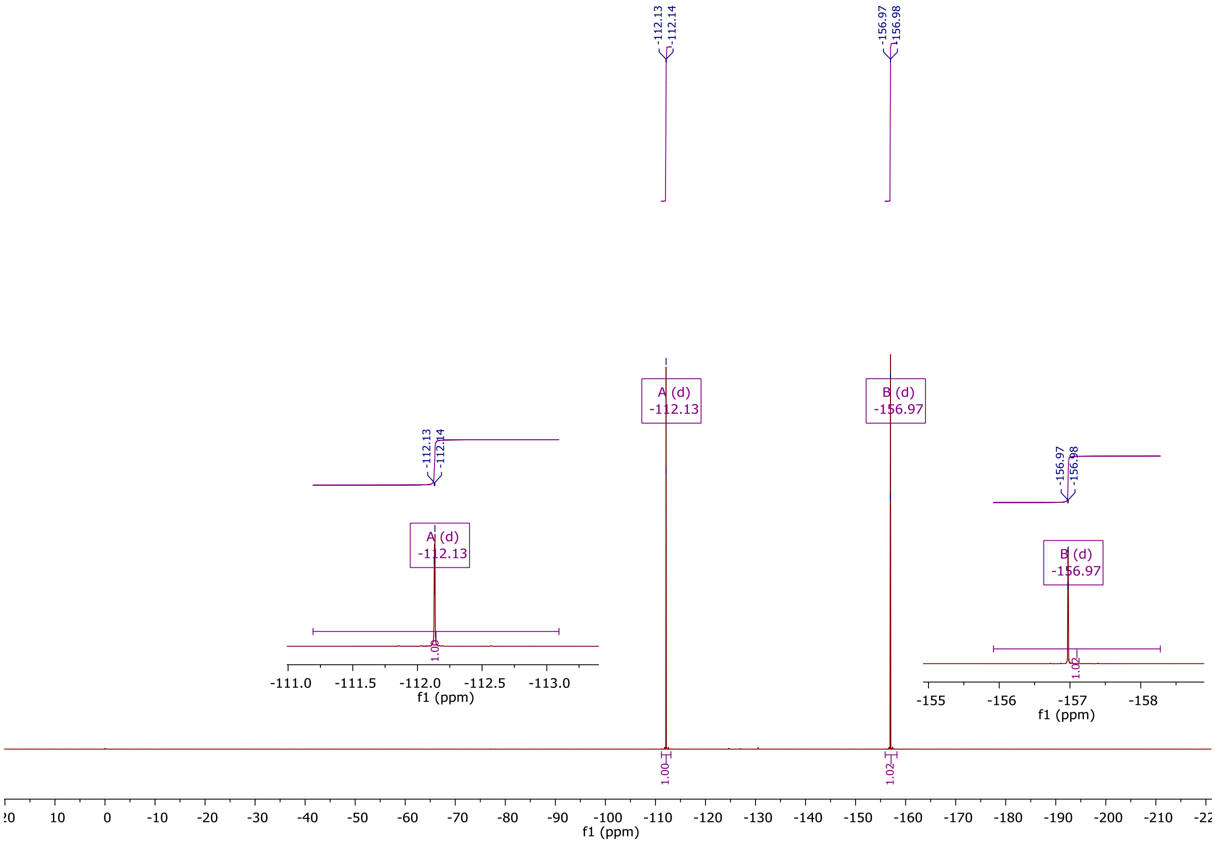

^{19}F{*I*^{1}H} NMR of (*R*,*Z*)-2,3-difluoro-*N*-(1-phenylethyl)hept-2-enamide **9c**

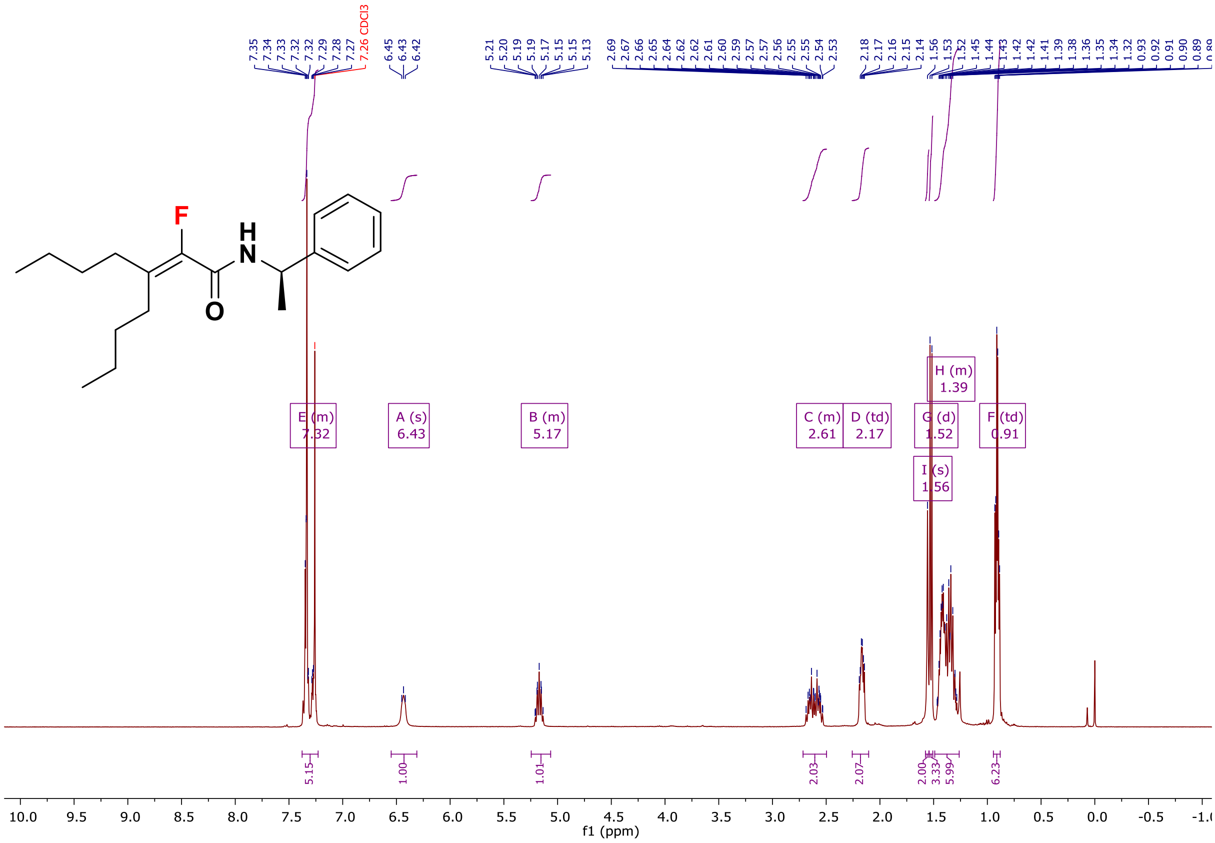

^{1}**H NMR** of (*R*)-3-butyl-2-fluoro-*N*-(1-phenylethyl)hept-2-enamide **10c**

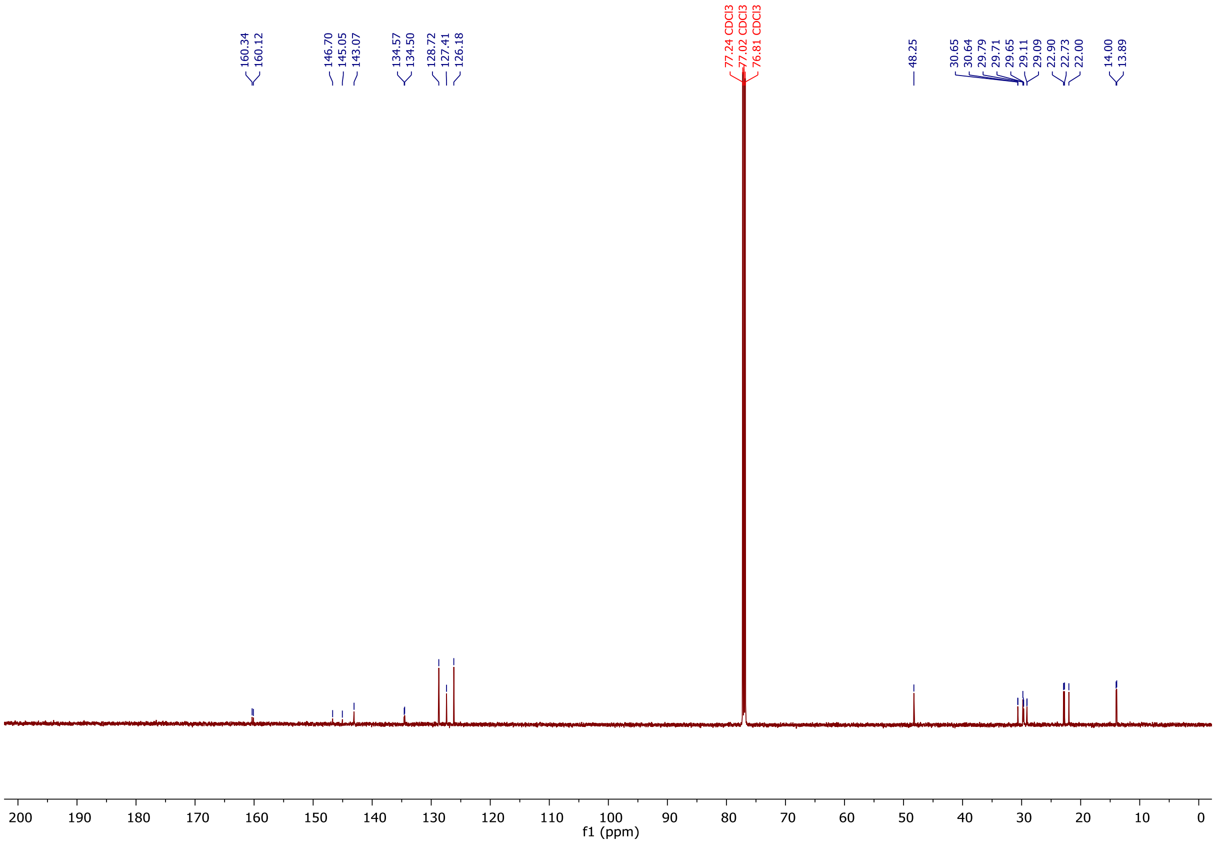

^{13}**C NMR** of (*R*)-3-butyl-2-fluoro-*N*-(1-phenylethyl)hept-2-enamide **10c**

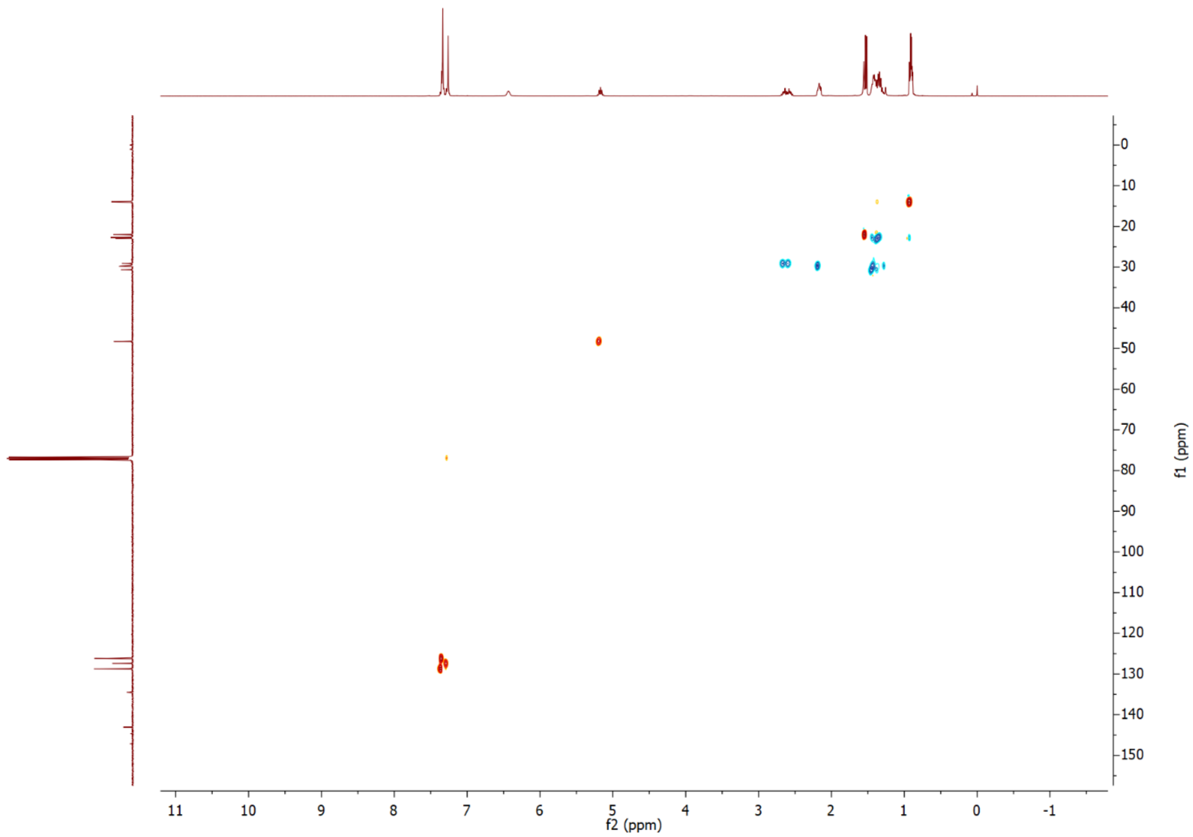

^{1}H-^{13}C HSQC of (*R*)-3-butyl-2-fluoro-*N*-(1-phenylethyl)hept-2-enamide **10c**

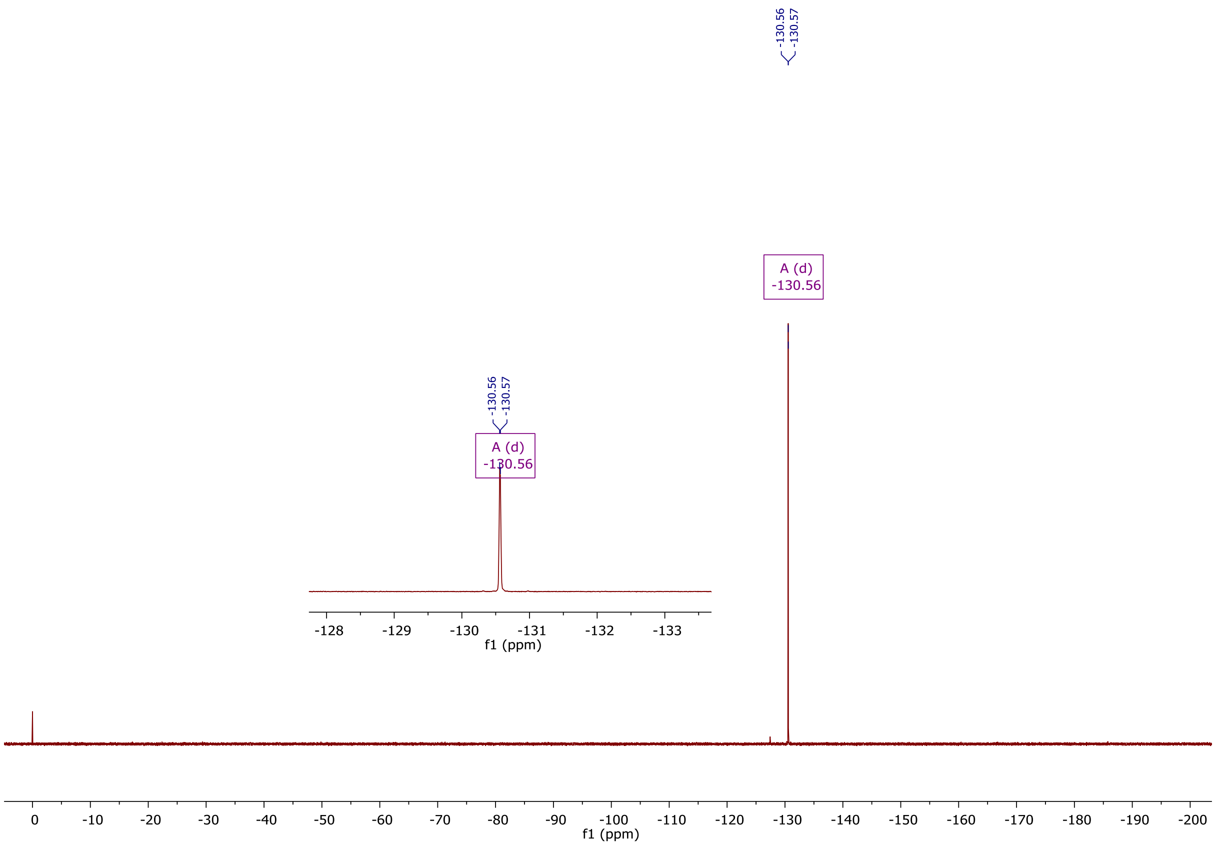

^{19}F NMR of (*R*)-3-butyl-2-fluoro-*N*-(1-phenylethyl)hept-2-enamide **10c**

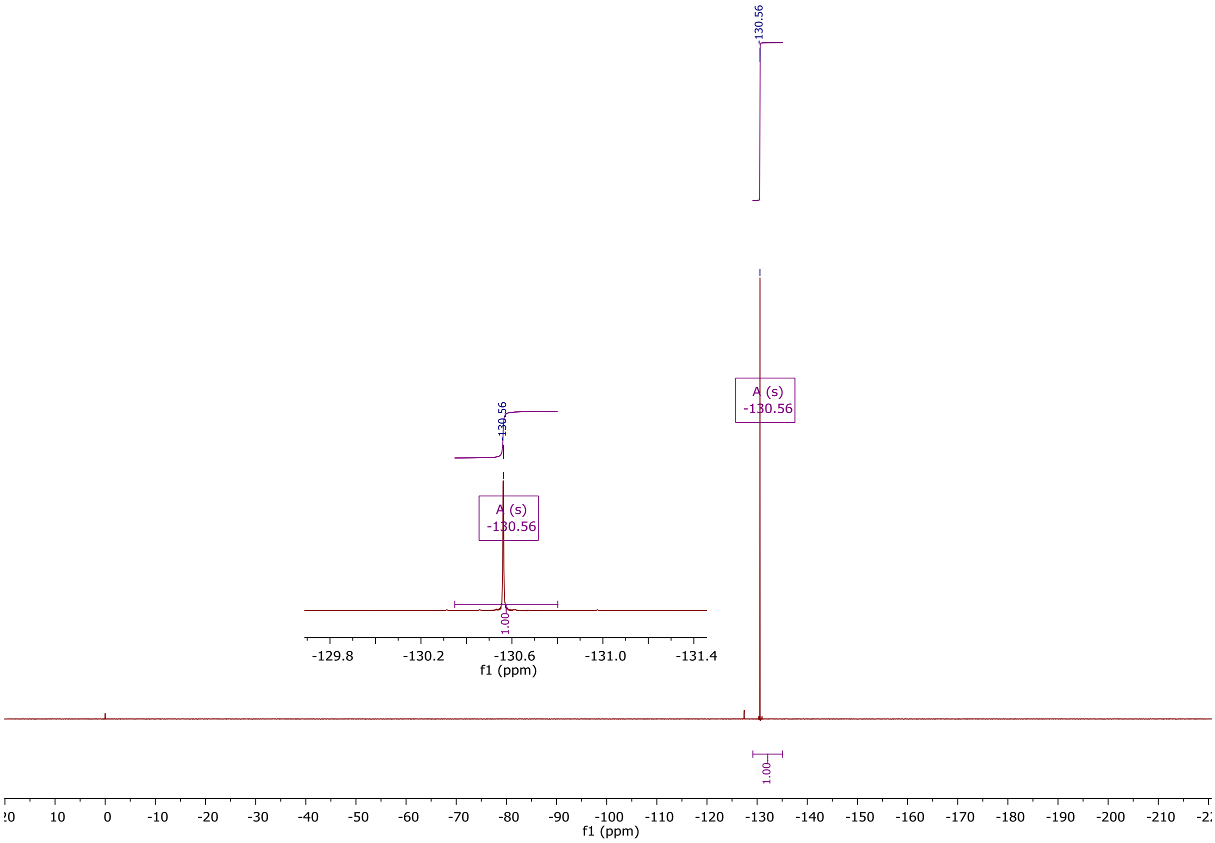

^{19}**F{^{1}H} NMR** of (*R*)-3-butyl-2-fluoro-*N*-(1-phenylethyl)hept-2-enamide **10c**

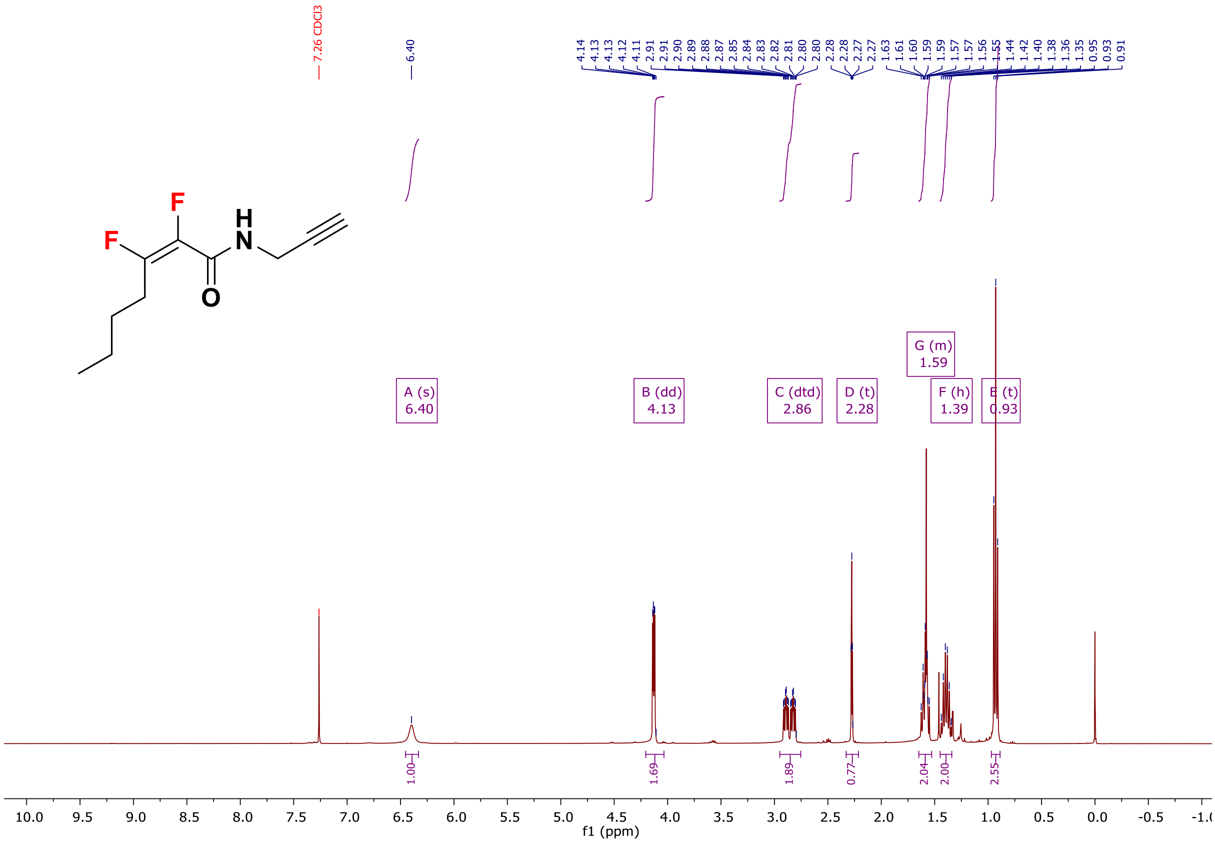

^{1}**H NMR** of (*Z*)-2,3-difluoro-*N*-(prop-2-yn-1-yl)hept-2-enamide **9d**

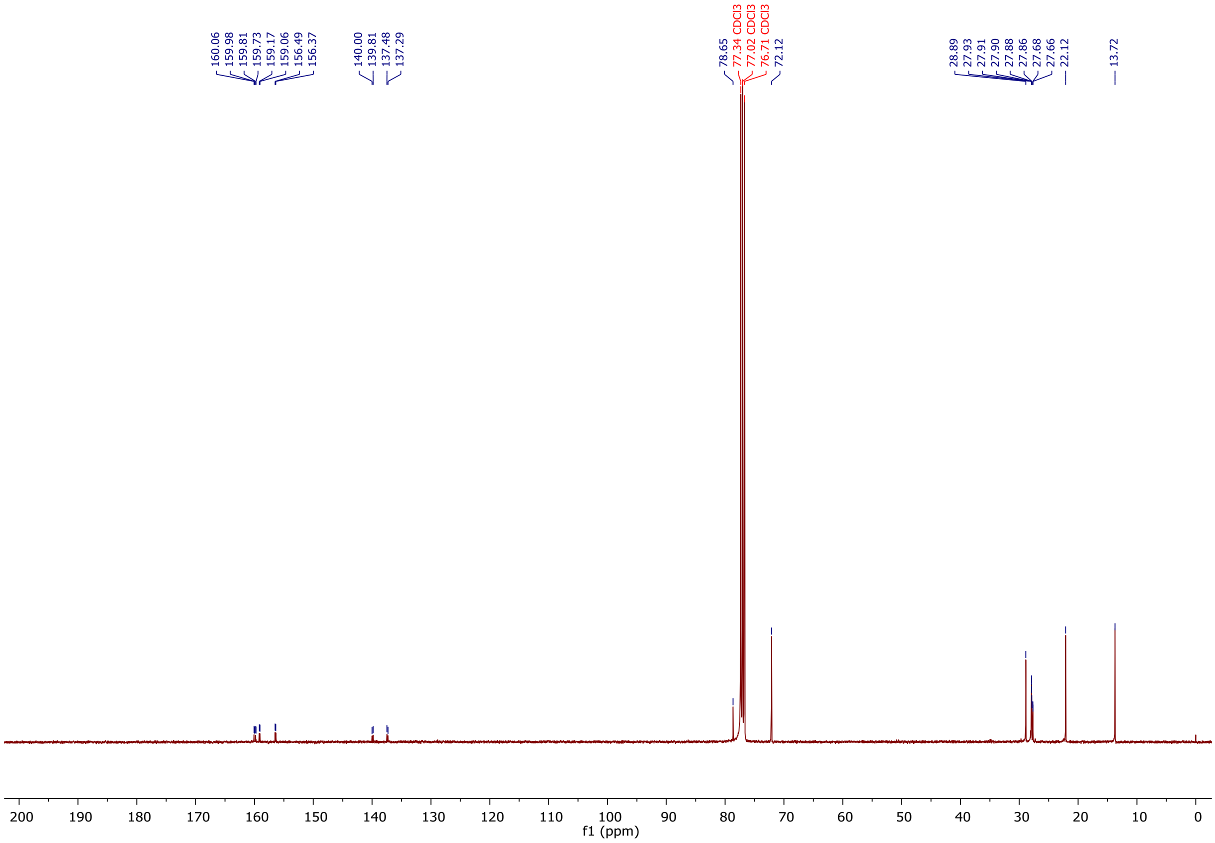

^{13}C NMR of (*Z*)-2,3-difluoro-*N*-(prop-2-yn-1-yl)hept-2-enamide **9d**

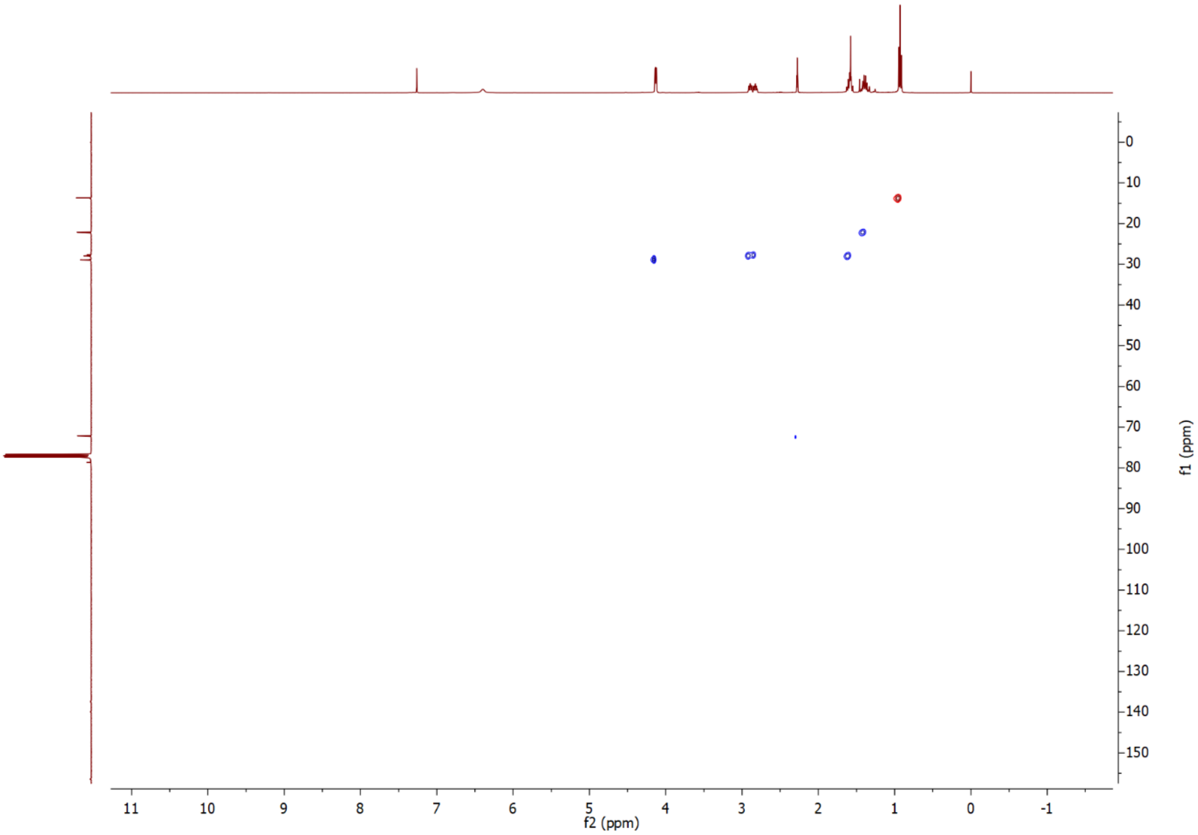

^{1}H-^{13}C HSQC of (*Z*)-2,3-difluoro-*N*-(prop-2-yn-1-yl)hept-2-enamide **9d**

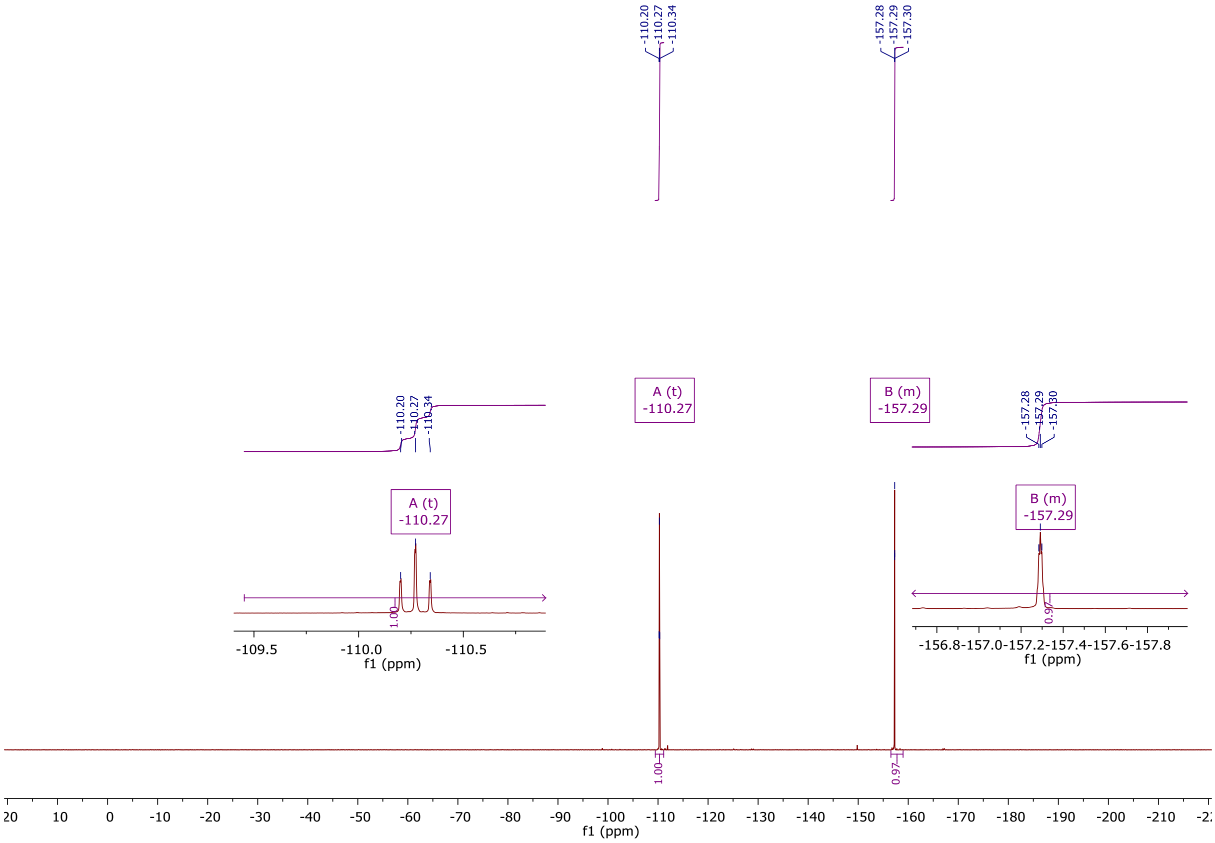

^{19}F NMR of (*Z*)-2,3-difluoro-*N*-(prop-2-yn-1-yl)hept-2-enamide **9d**

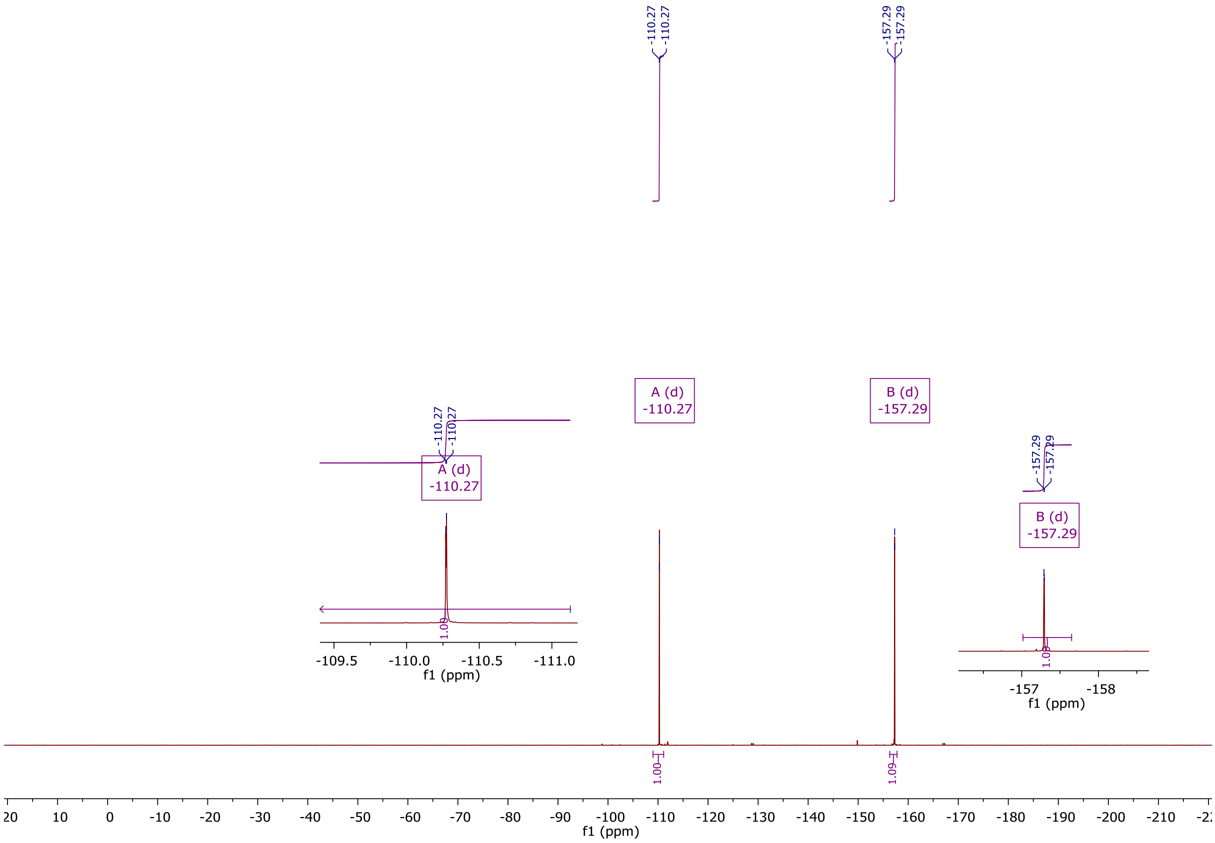

^{19}F{/^{1}H} NMR of (*Z*)-2,3-difluoro-*N*-(prop-2-yn-1-yl)hept-2-enamide **9d**

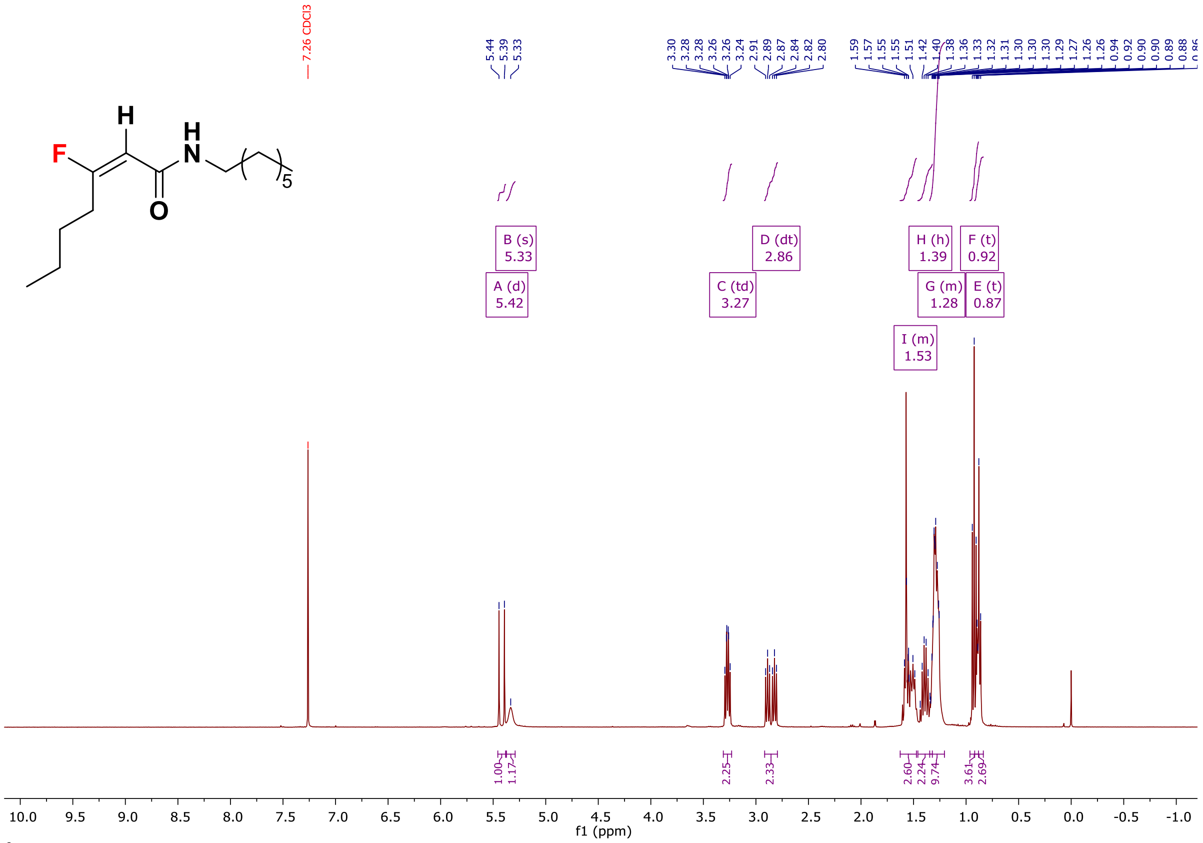

^{1}**H NMR** of (*E*)-3-fluoro-*N*-heptylhept-2-enamide **11a**

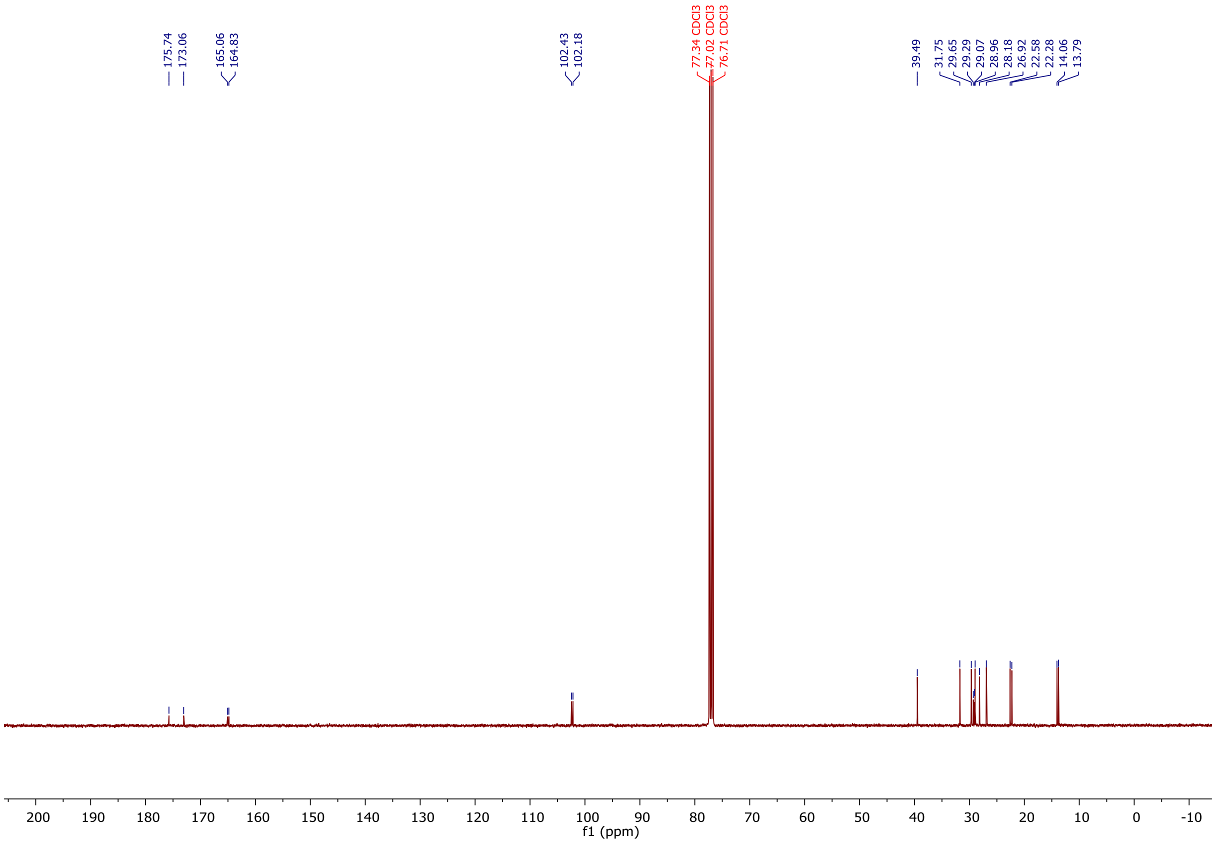

^{13}**C NMR** of (*E*)-3-fluoro-*N*-heptylhept-2-enamide **11a**

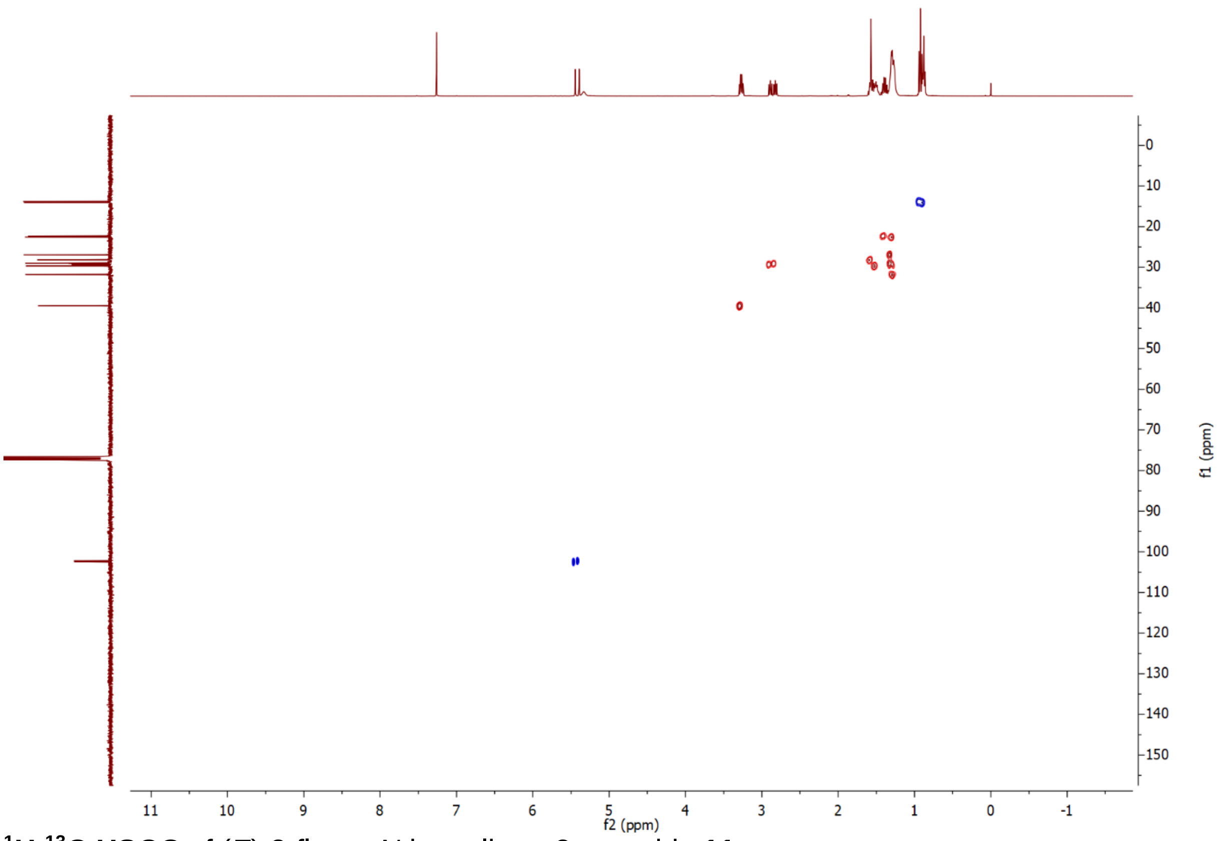

^{1}H-^{13}C HSQC of (*E*)-3-fluoro-*N*-heptylhept-2-enamide **11a**

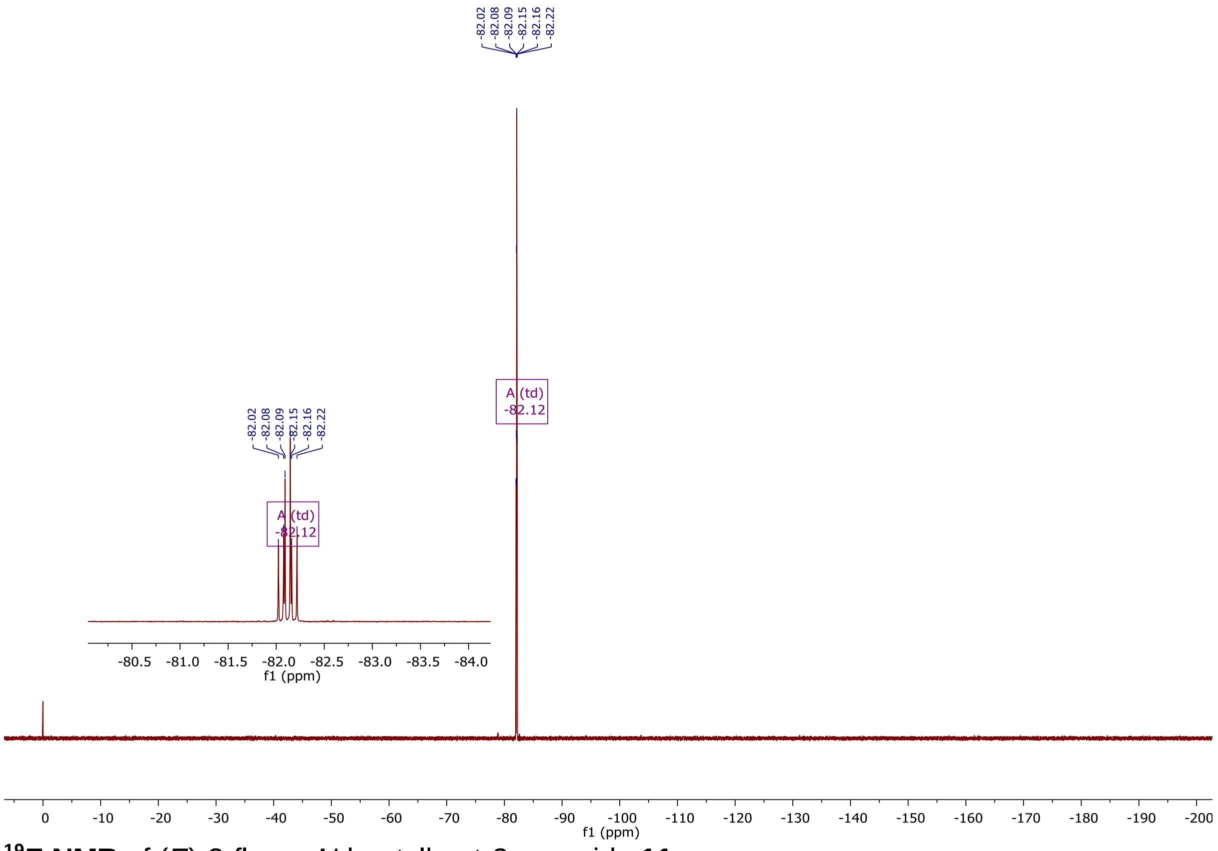

^{19}F NMR of (*E*)-3-fluoro-*N*-heptylhept-2-enamide **11a**

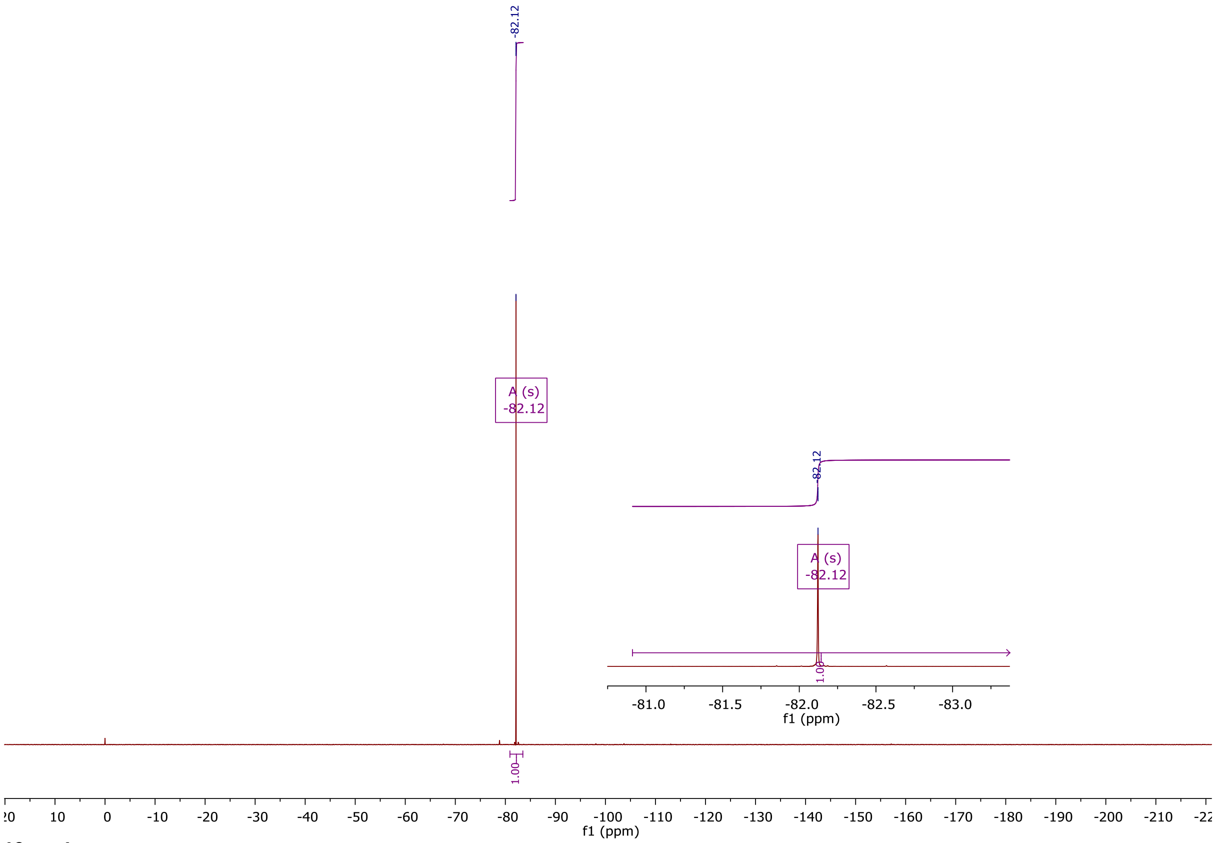

^{19}F{/^{1}H} NMR of (*E*)-3-fluoro-*N*-heptylhept-2-enamide **11a**

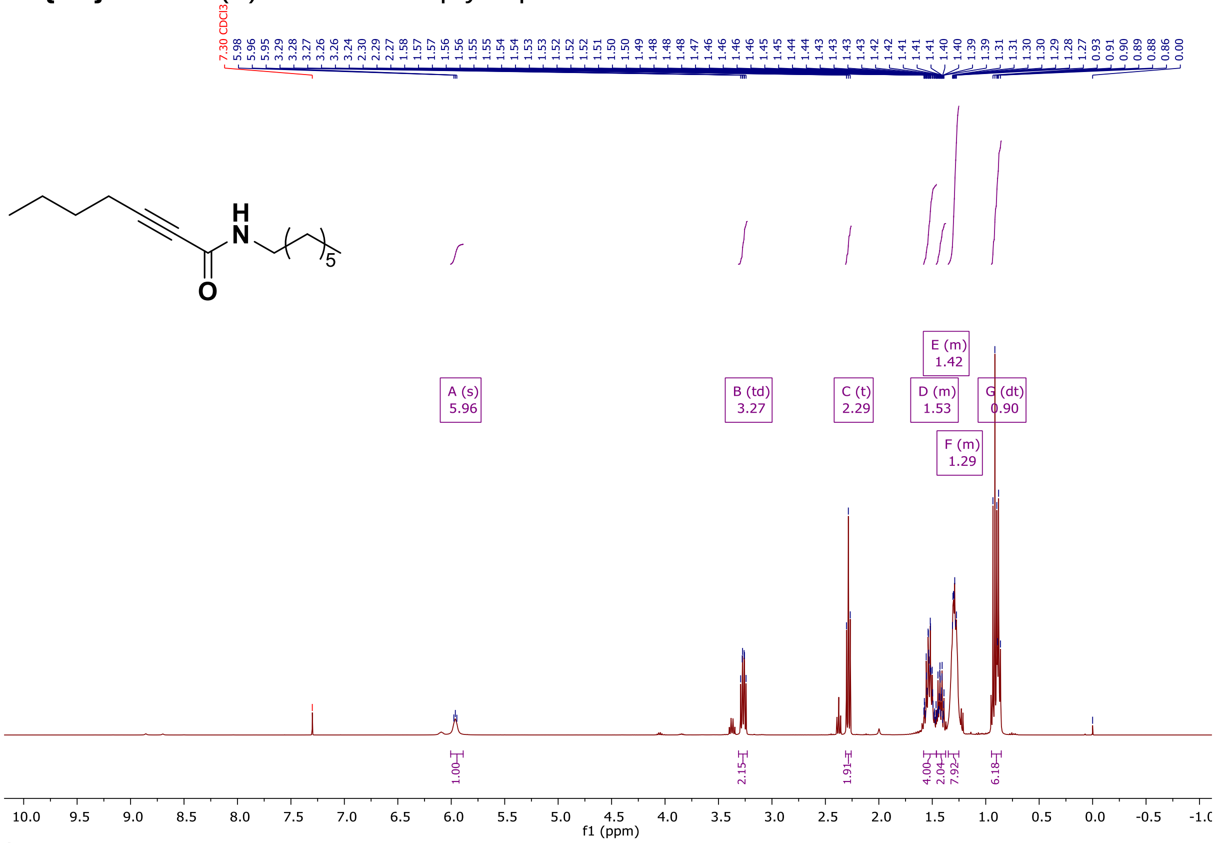

^{1}H NMR of *N*-heptylhept-2-ynamide **12a**

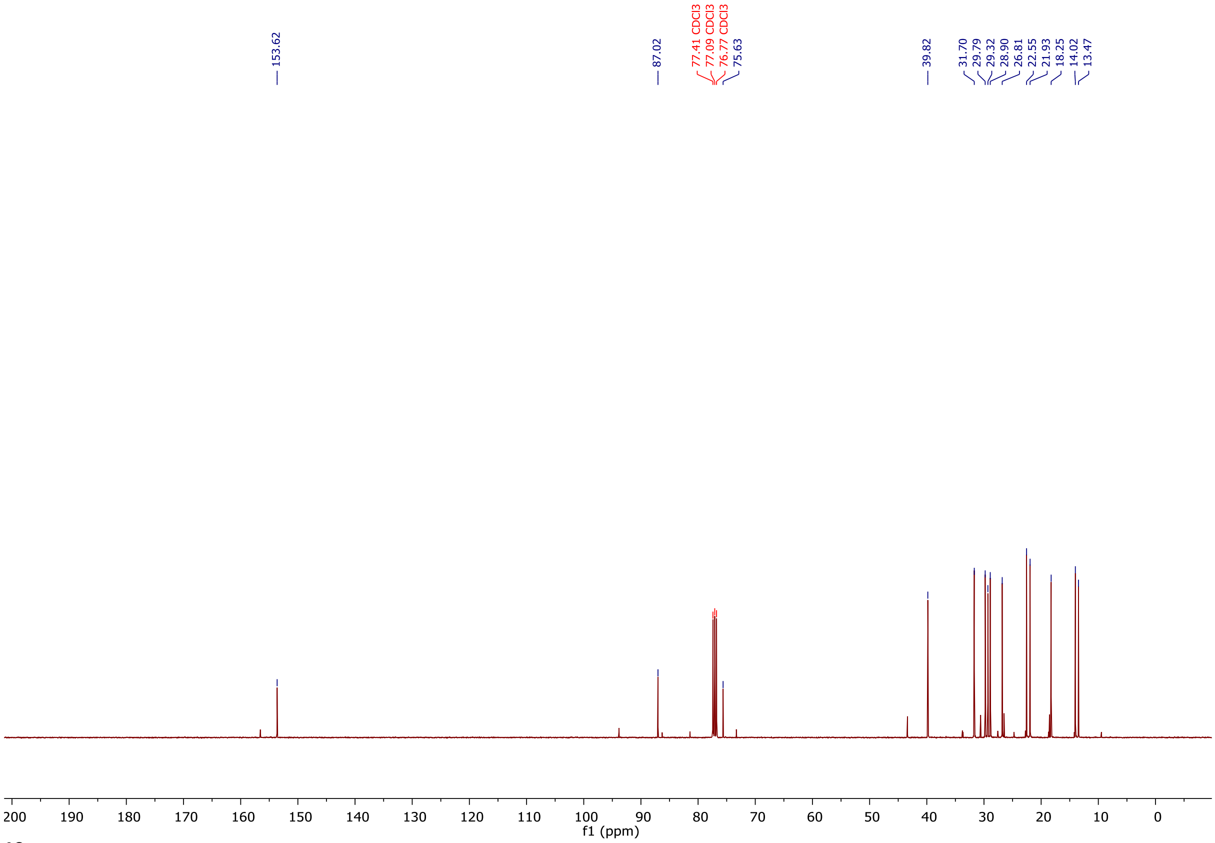

^{13}**C NMR** of *N*-heptylhept-2-ynamide **12a**

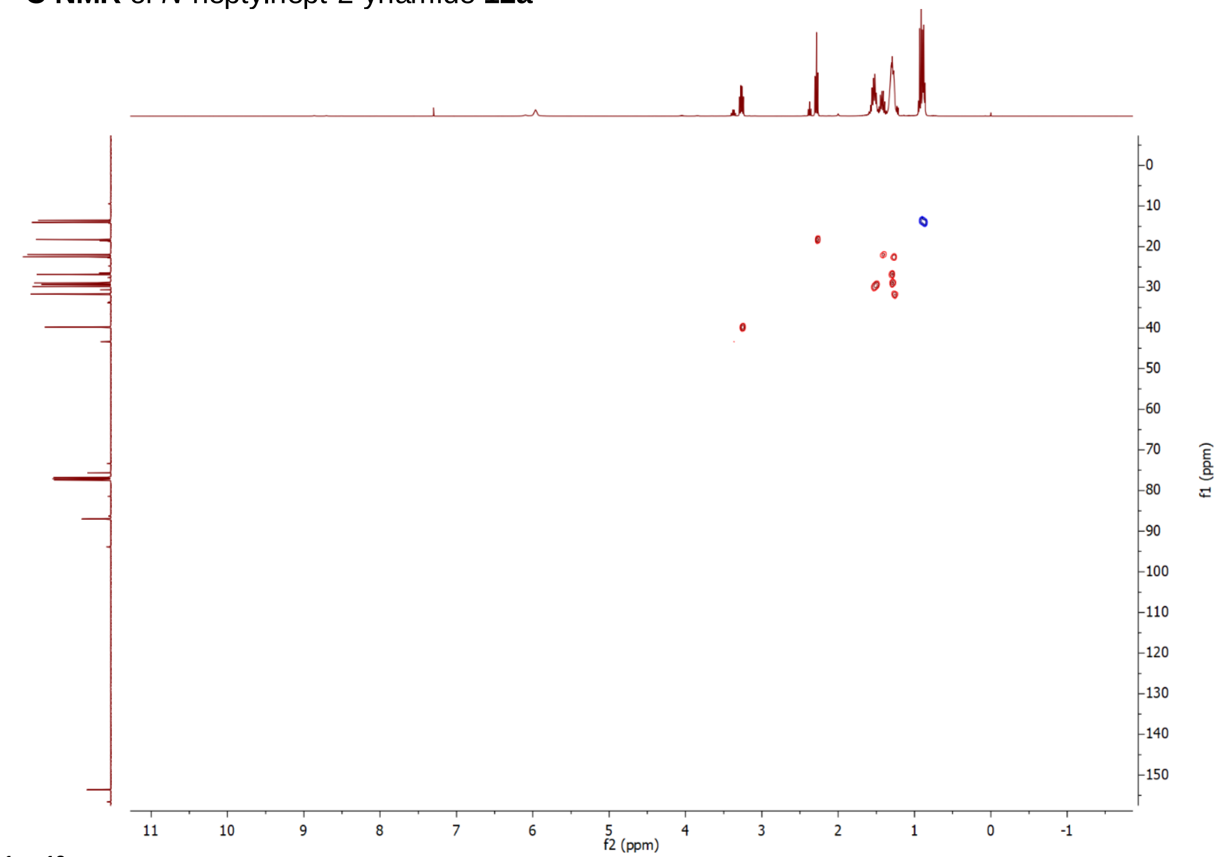

^{1}**H-**^{13}**C HSQC** of *N*-heptylhept-2-ynamide **12a**

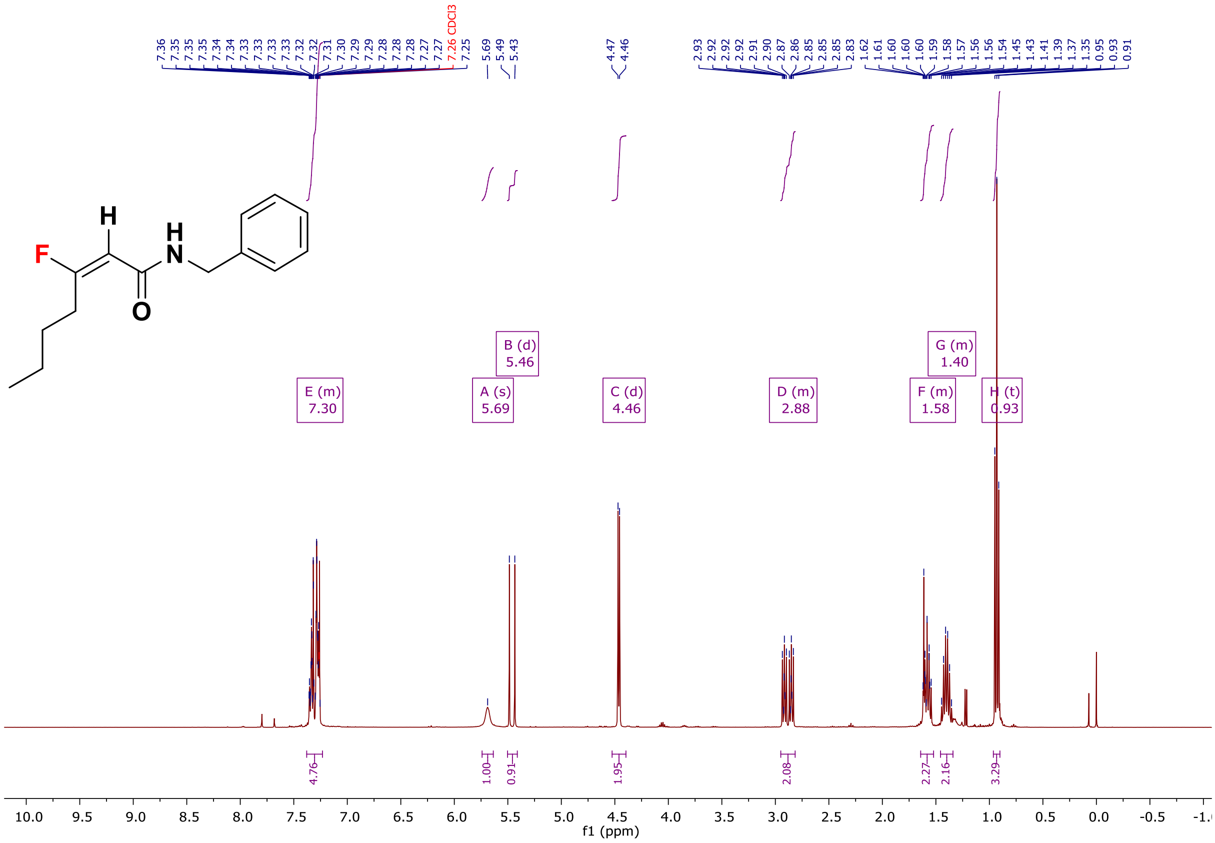

^{1}H NMR of (*E*)-*N*-benzyl-3-fluorohept-2-enamide **11b**

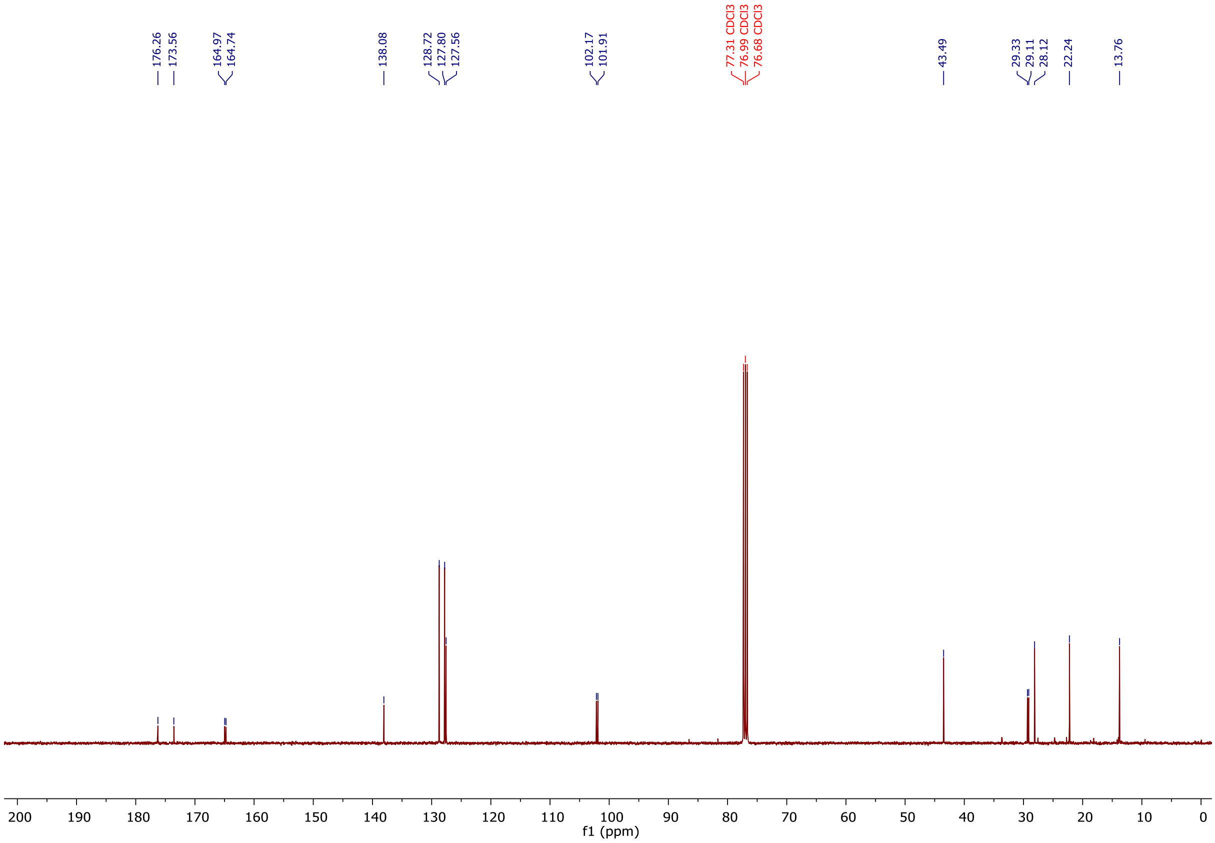

^{13}C NMR of (*E*)-*N*-benzyl-3-fluorohept-2-enamide **11b**

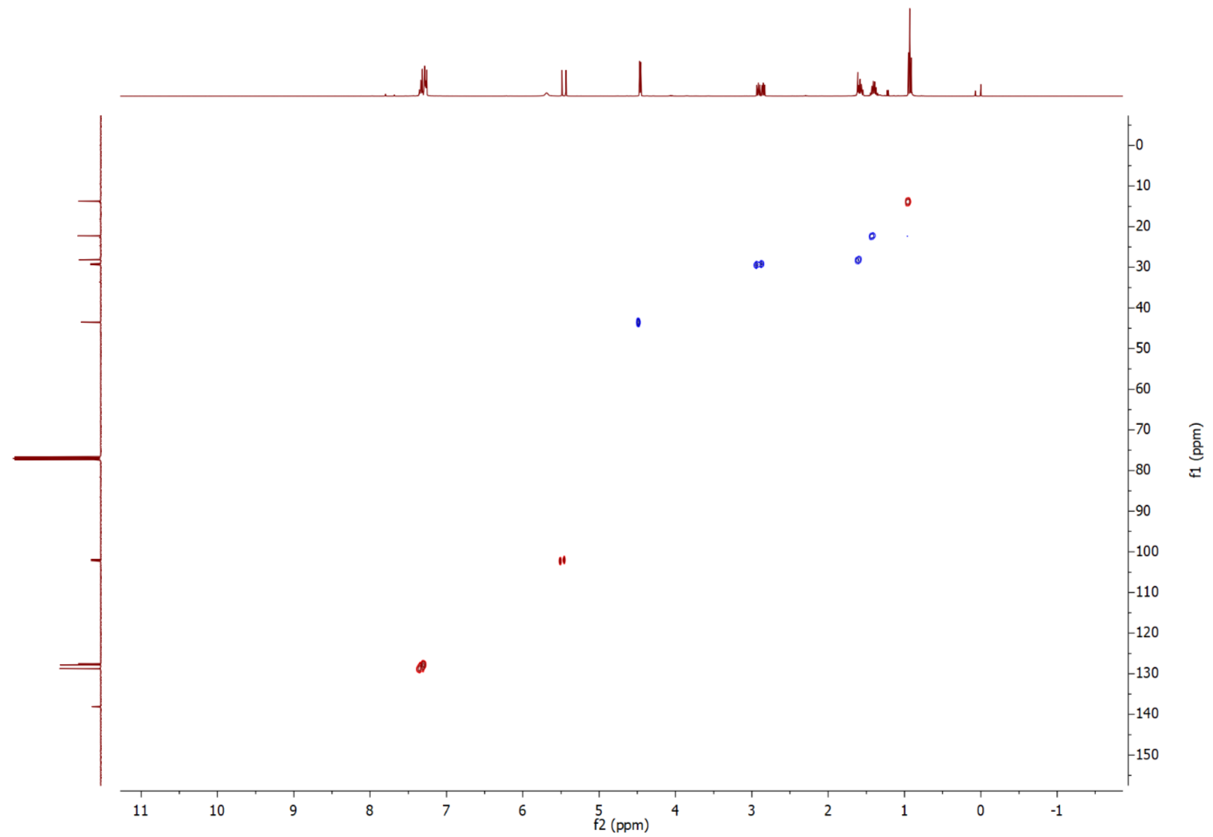

^{1}H-^{13}C HSQC of (*E*)-*N*-benzyl-3-fluorohept-2-enamide **11b**

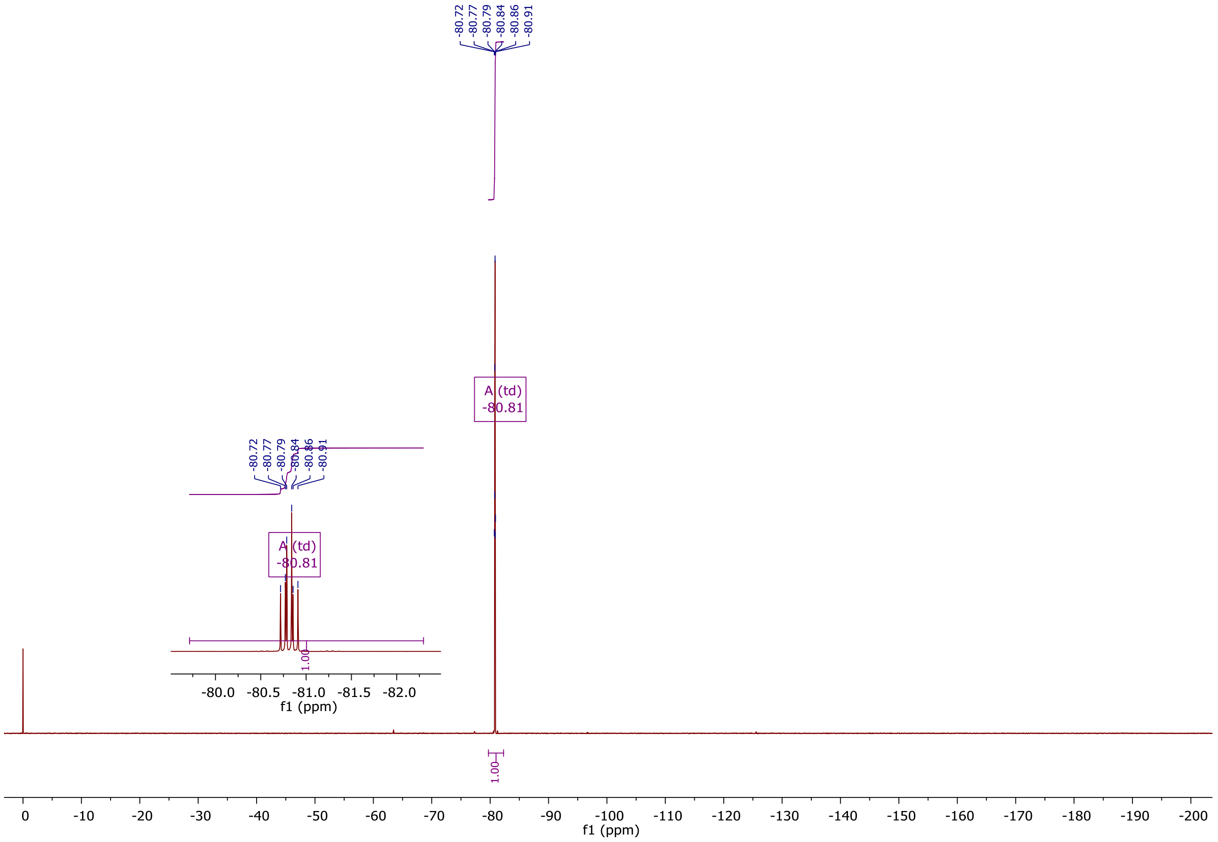

^{19}F NMR of (*E*)-*N*-benzyl-3-fluorohept-2-enamide **11b**

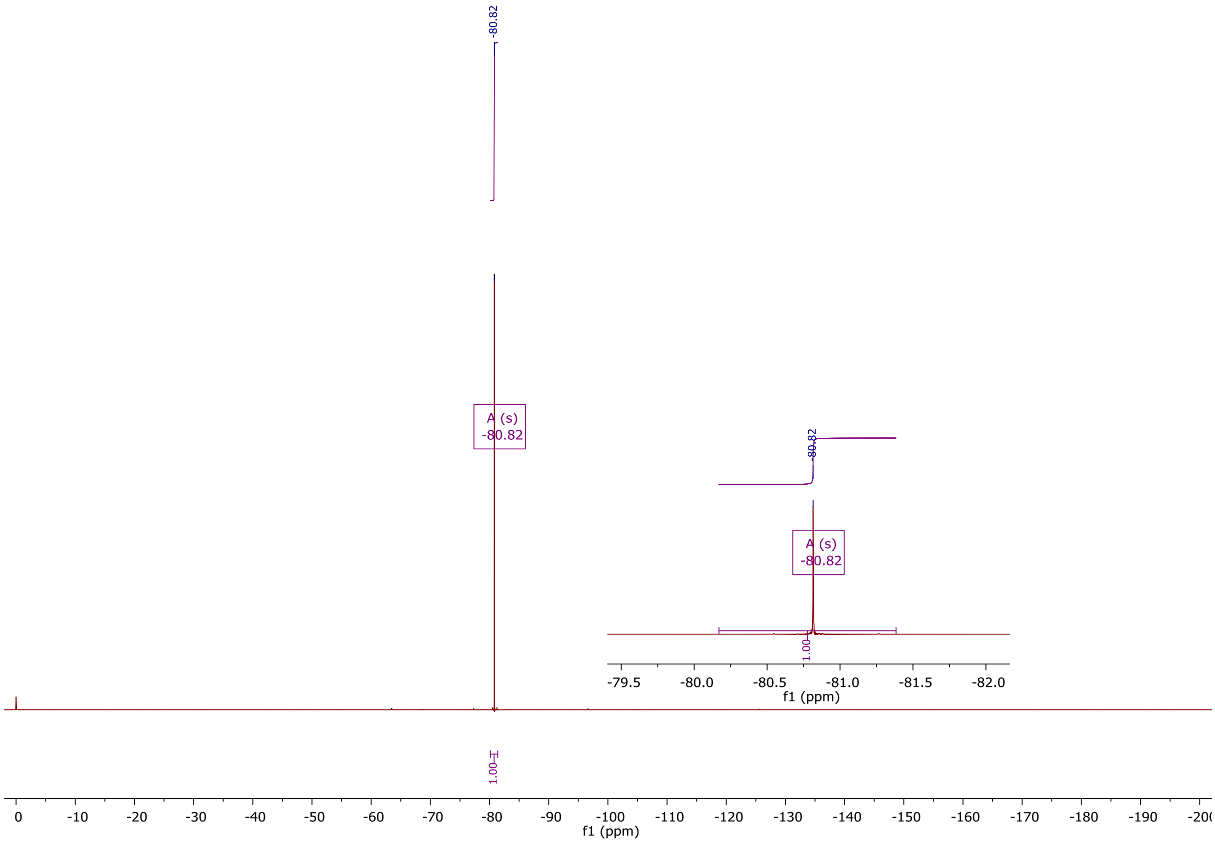

^{19}F{^{1}H} NMR of (*E*)-*N*-benzyl-3-fluorohept-2-enamide **11b**

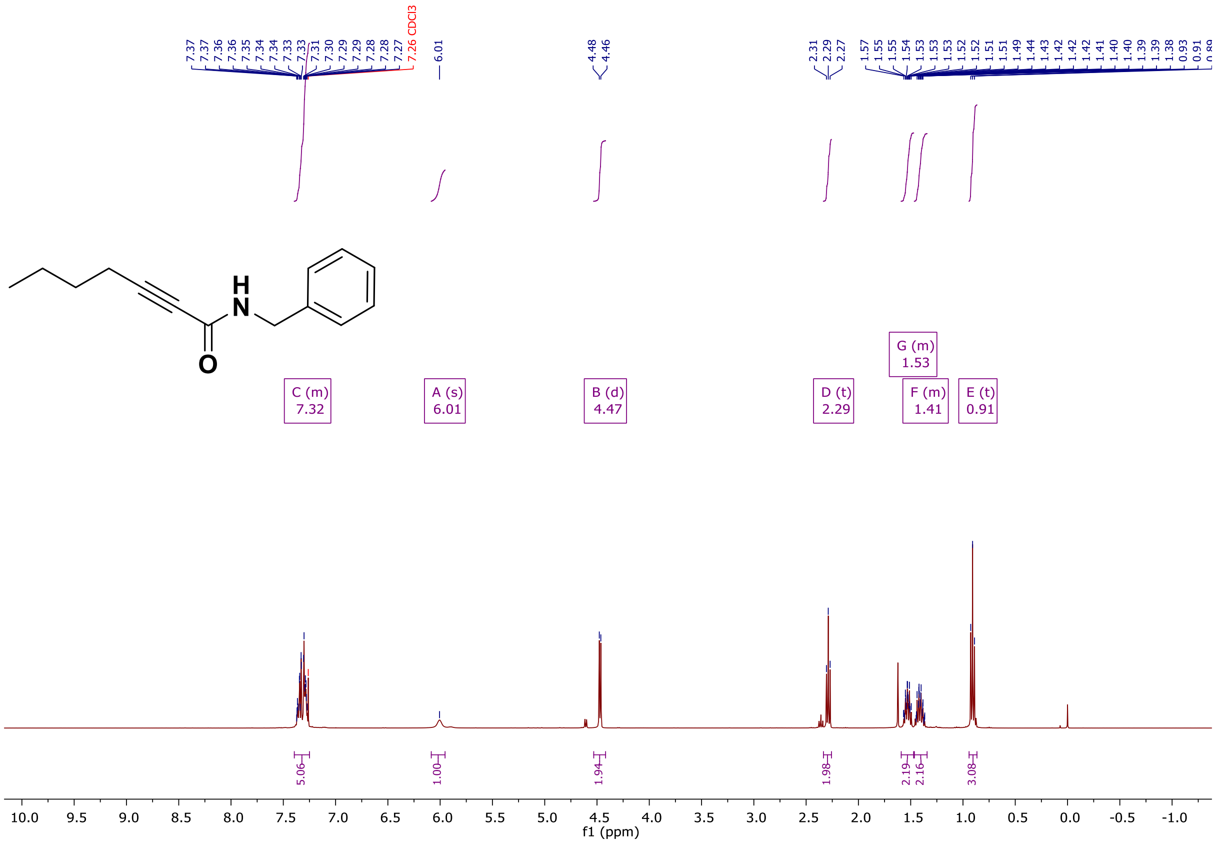

^{1}H NMR of *N*-benzylhept-2-ynamide **12b**

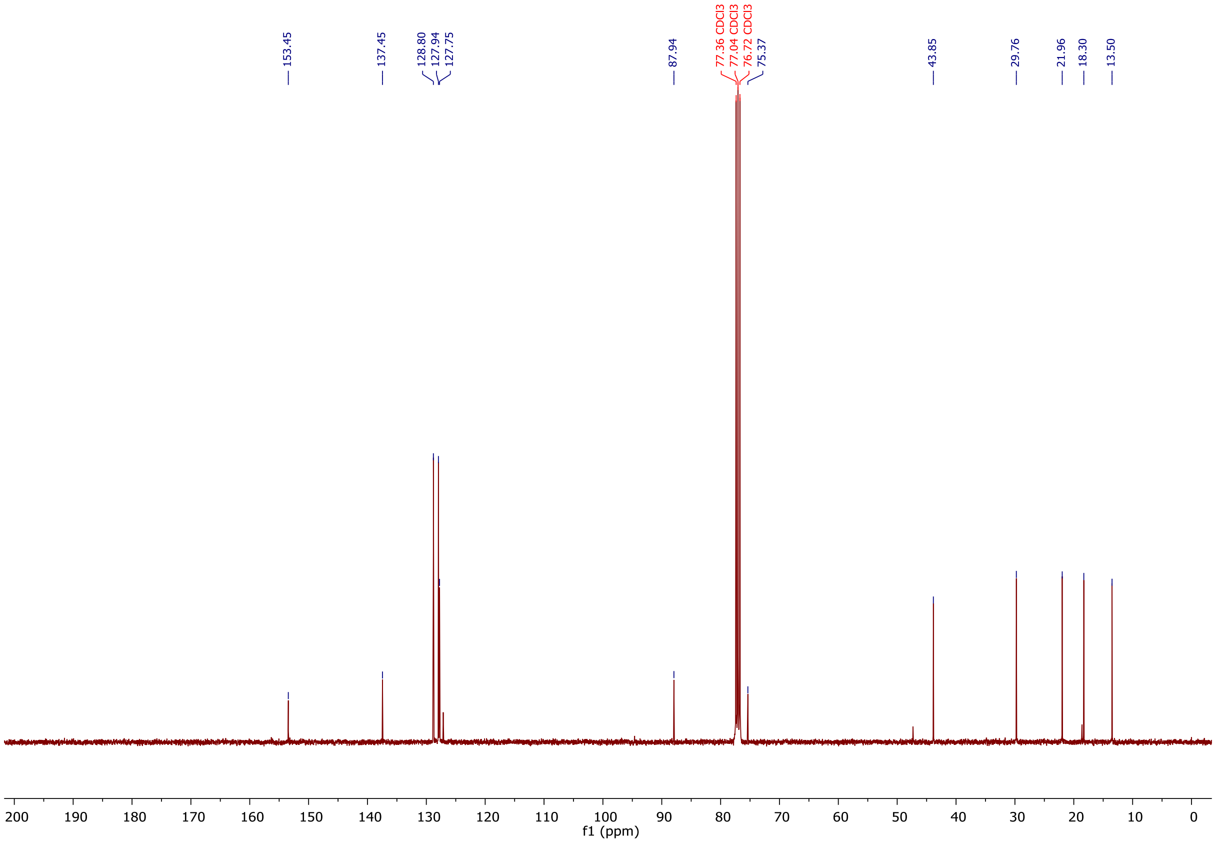

^{13}C NMR of *N*-benzylhept-2-ynamide **12b**

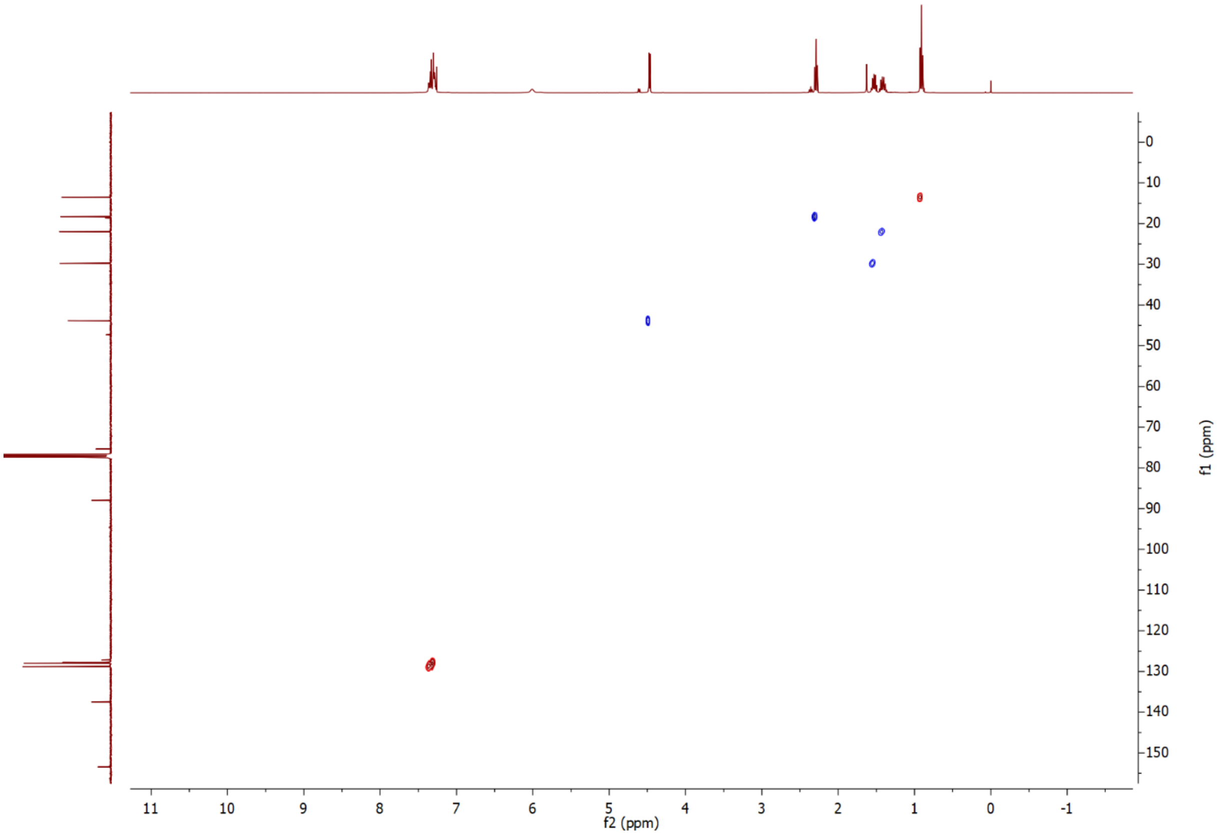

^{1}H-^{13}C HSQC of *N*-benzylhept-2-ynamide **12b**

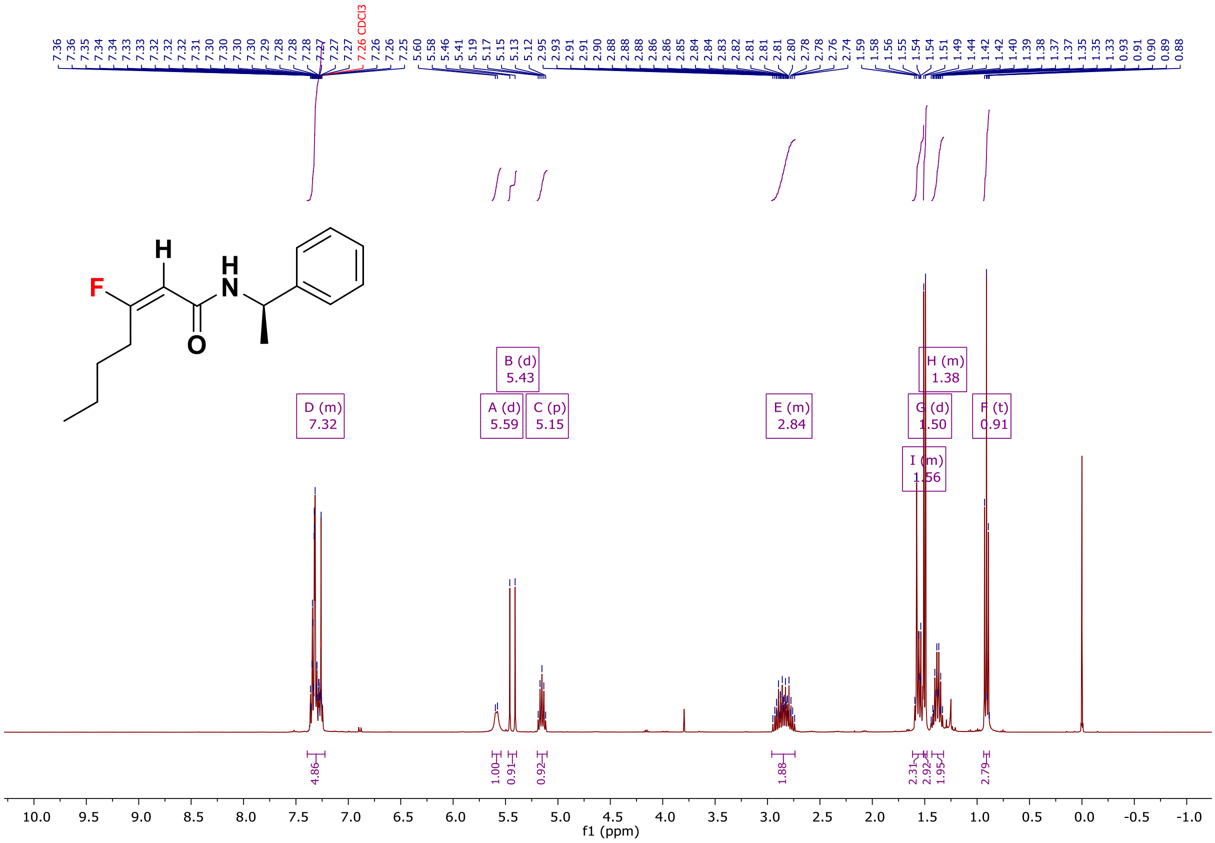

^{1}**H NMR** of (*R*,*E*)-3-fluoro-*N*-(1-phenylethyl)hept-2-enamide **11c**

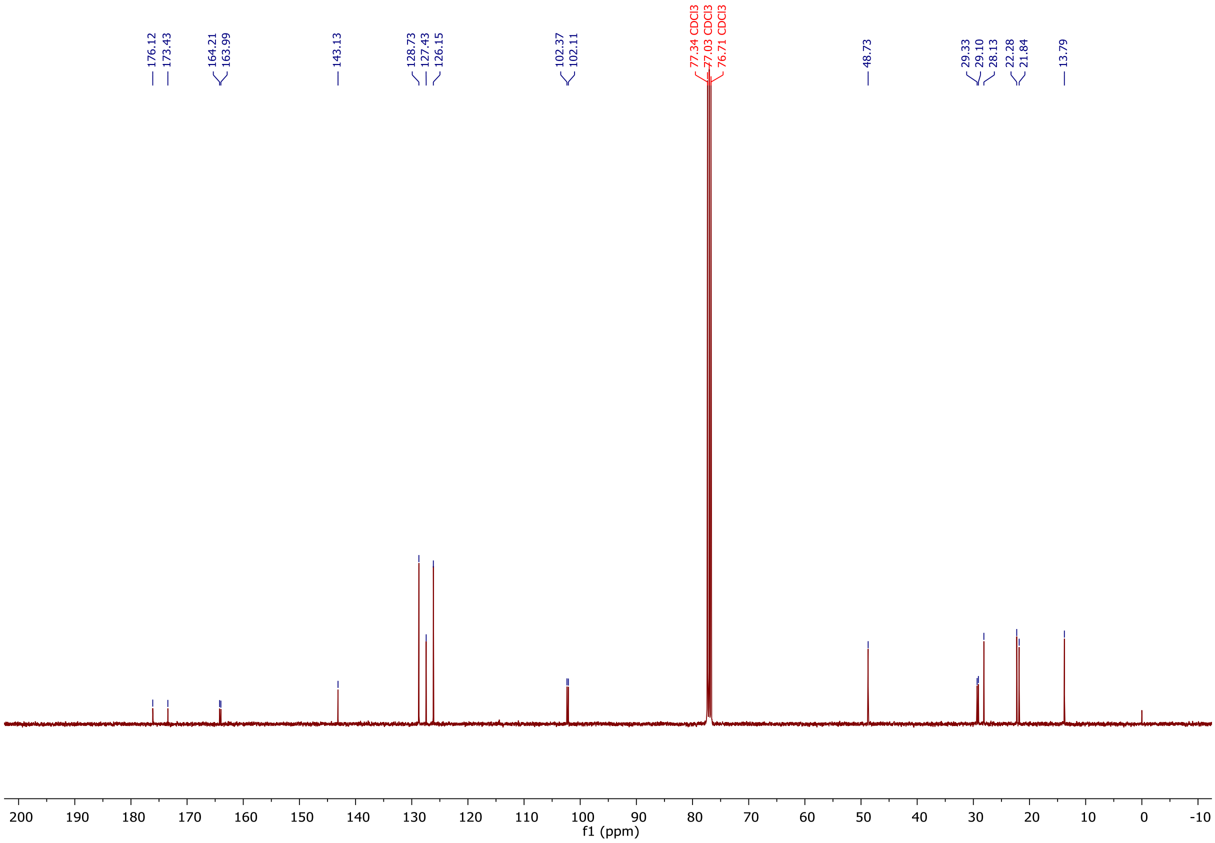

^{13}**C NMR** of (*R*,*E*)-3-fluoro-*N*-(1-phenylethyl)hept-2-enamide **11c**

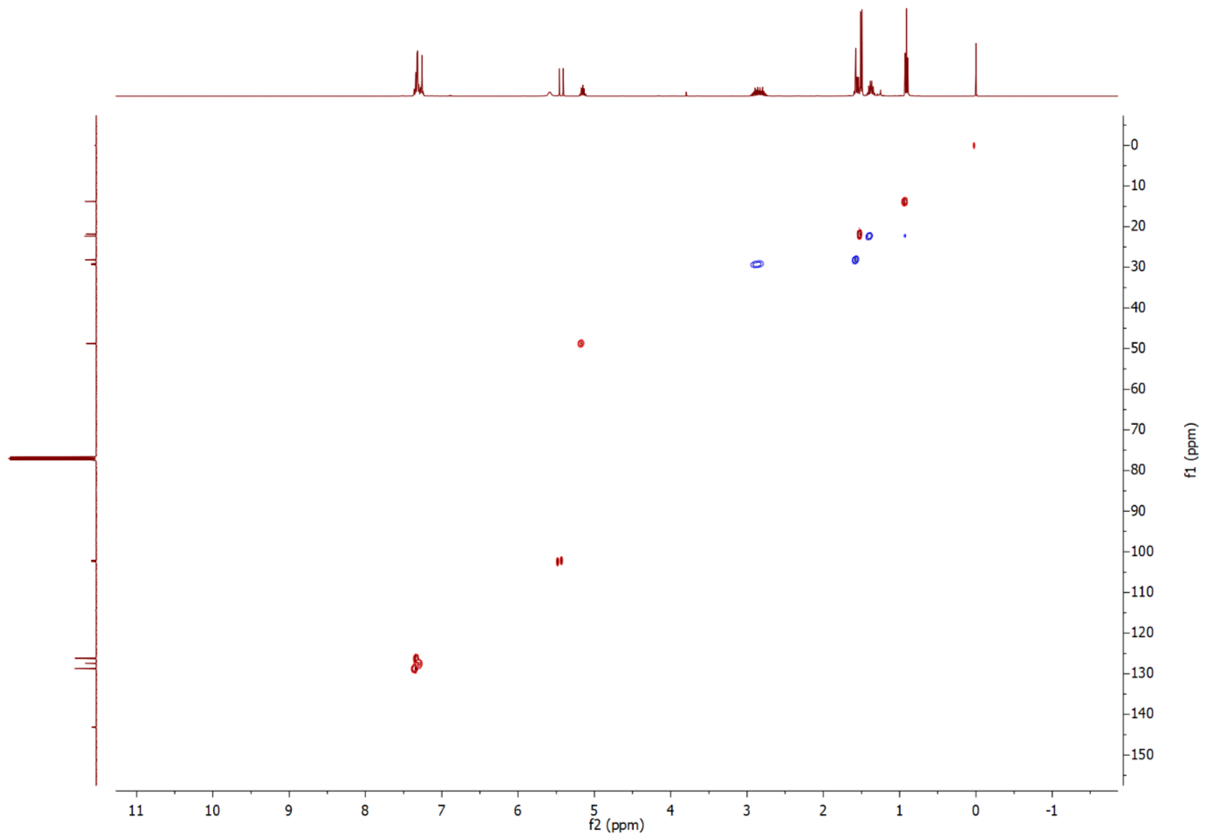

^{1}H-^{13}C HSQC of (*R*,*E*)-3-fluoro-*N*-(1-phenylethyl)hept-2-enamide **11c**

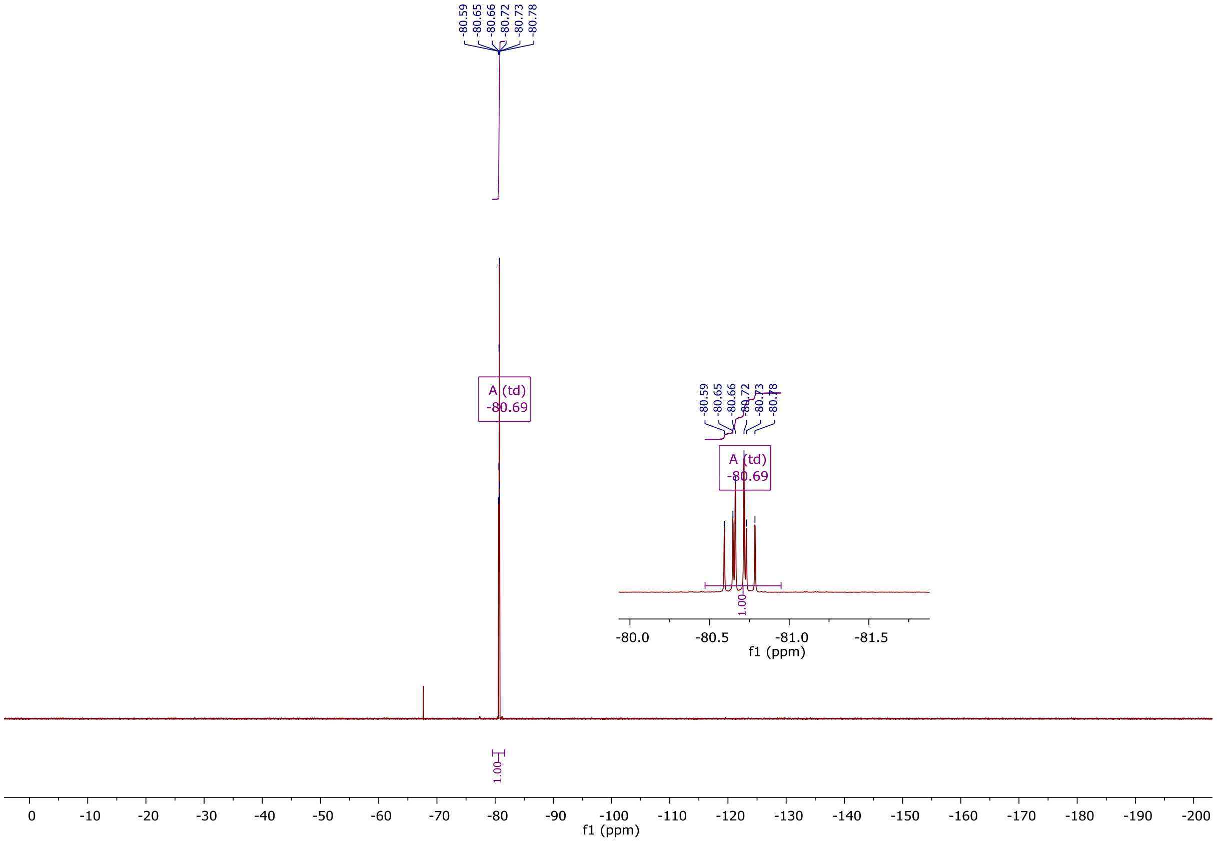

^{19}F NMR of (*R*,*E*)-3-fluoro-*N*-(1-phenylethyl)hept-2-enamide **11c**

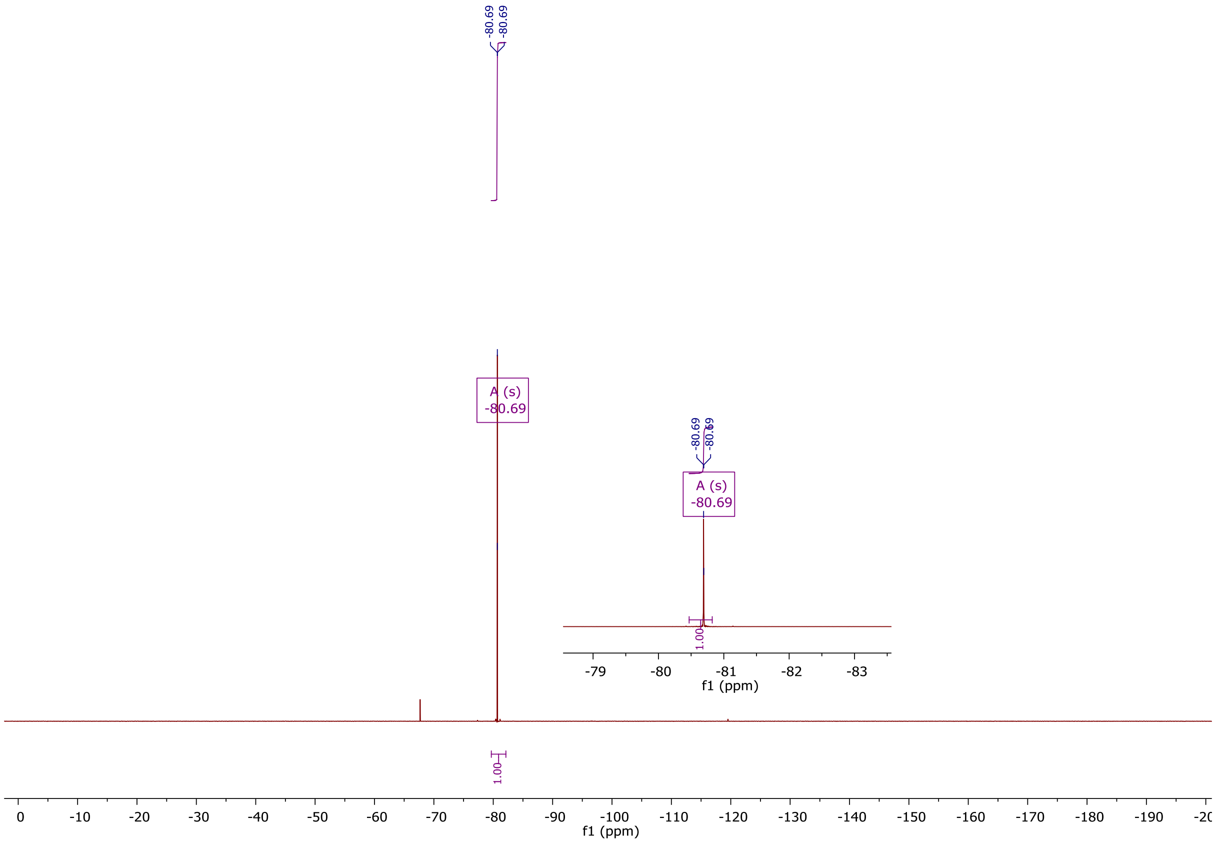

^{19}F{/^{1}H} NMR of (*R*,*E*)-3-fluoro-*N*-(1-phenylethyl)hept-2-enamide **11c**

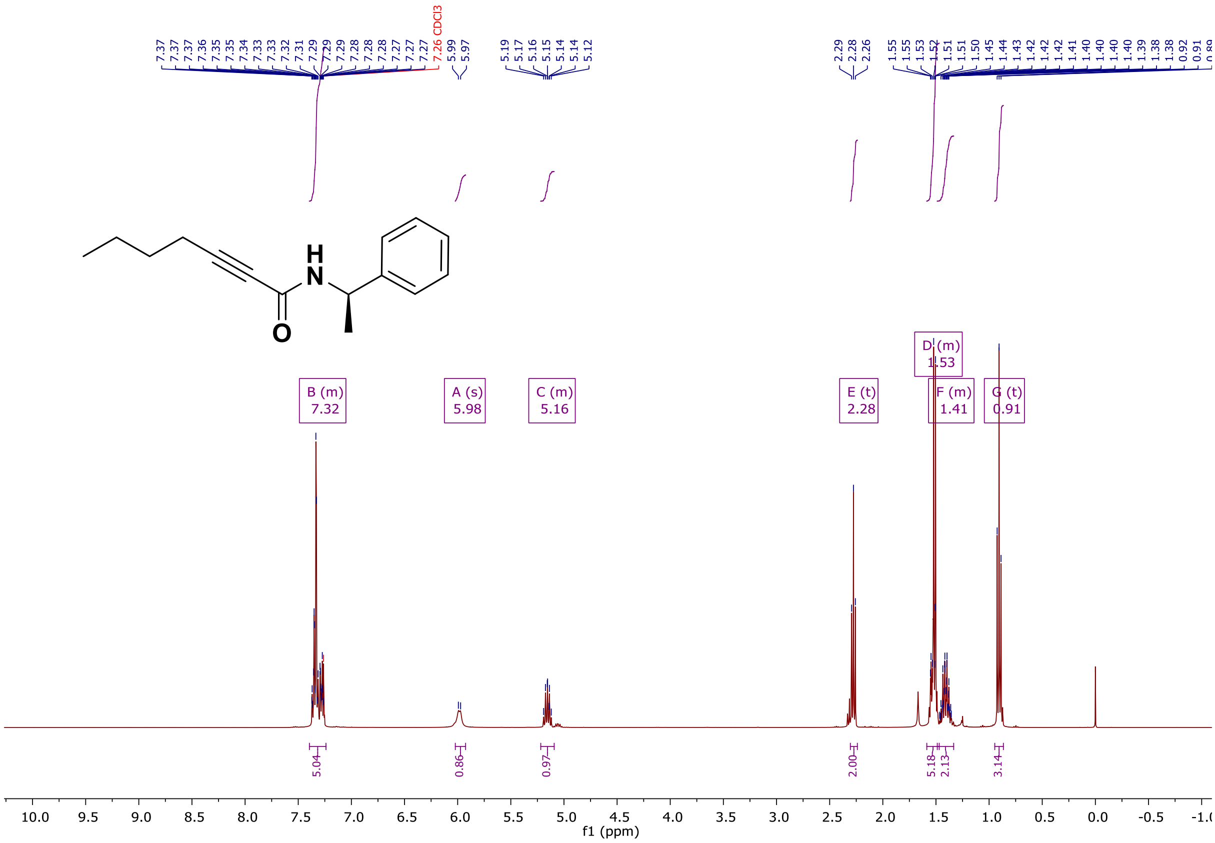

^{1}H NMR of (*R*)-*N*-(1-phenylethyl)hept-2-ynamide **12c**

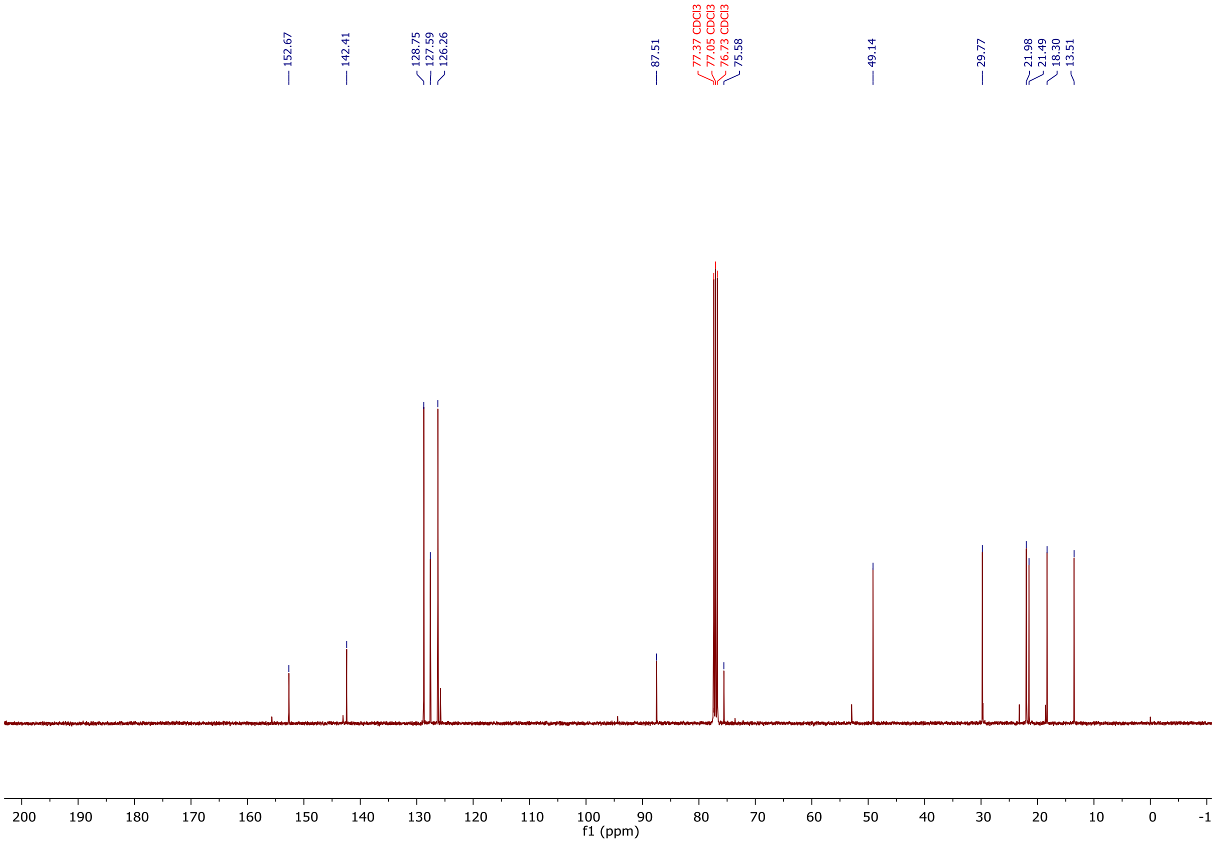

^{13}C NMR of (*R*)-*N*-(1-phenylethyl)hept-2-ynamide **12c**

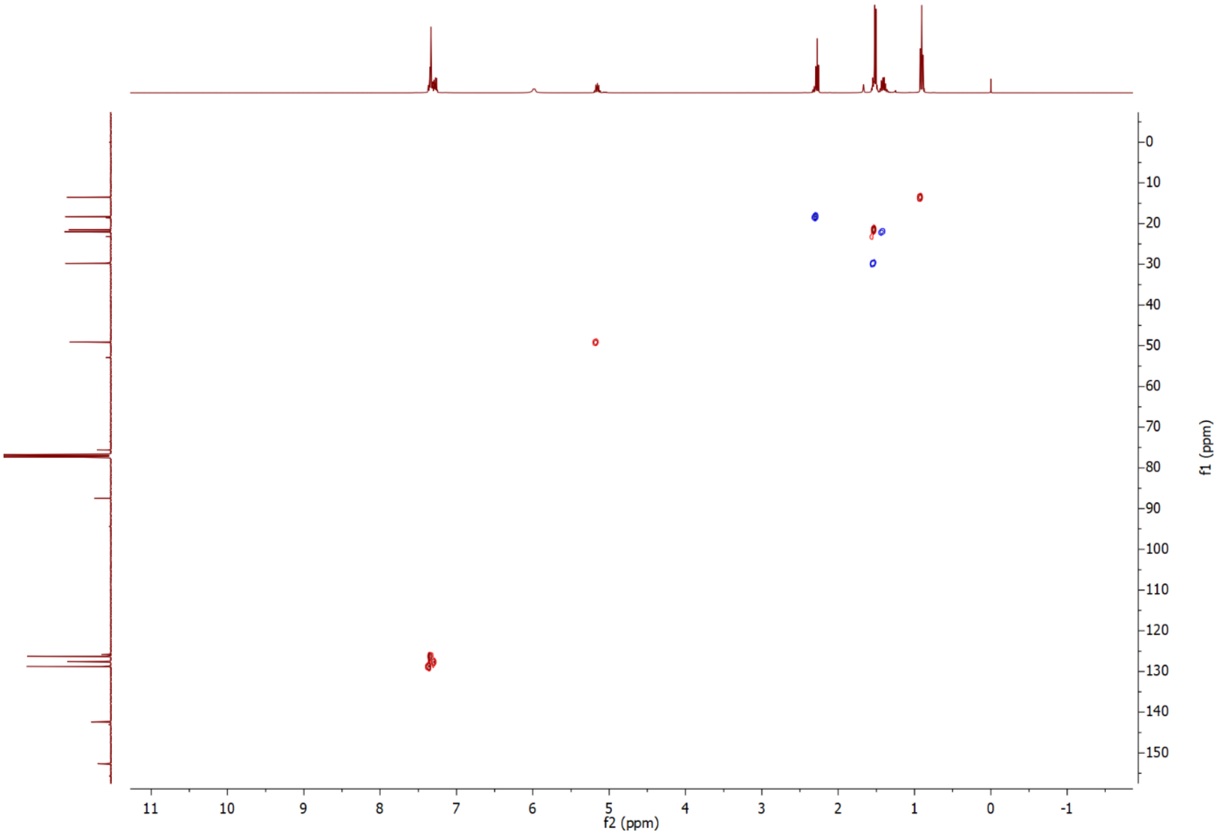

^{1}H-^{13}C HSQC of (*R*)-*N*-(1-phenylethyl)hept-2-ynamide **12c**

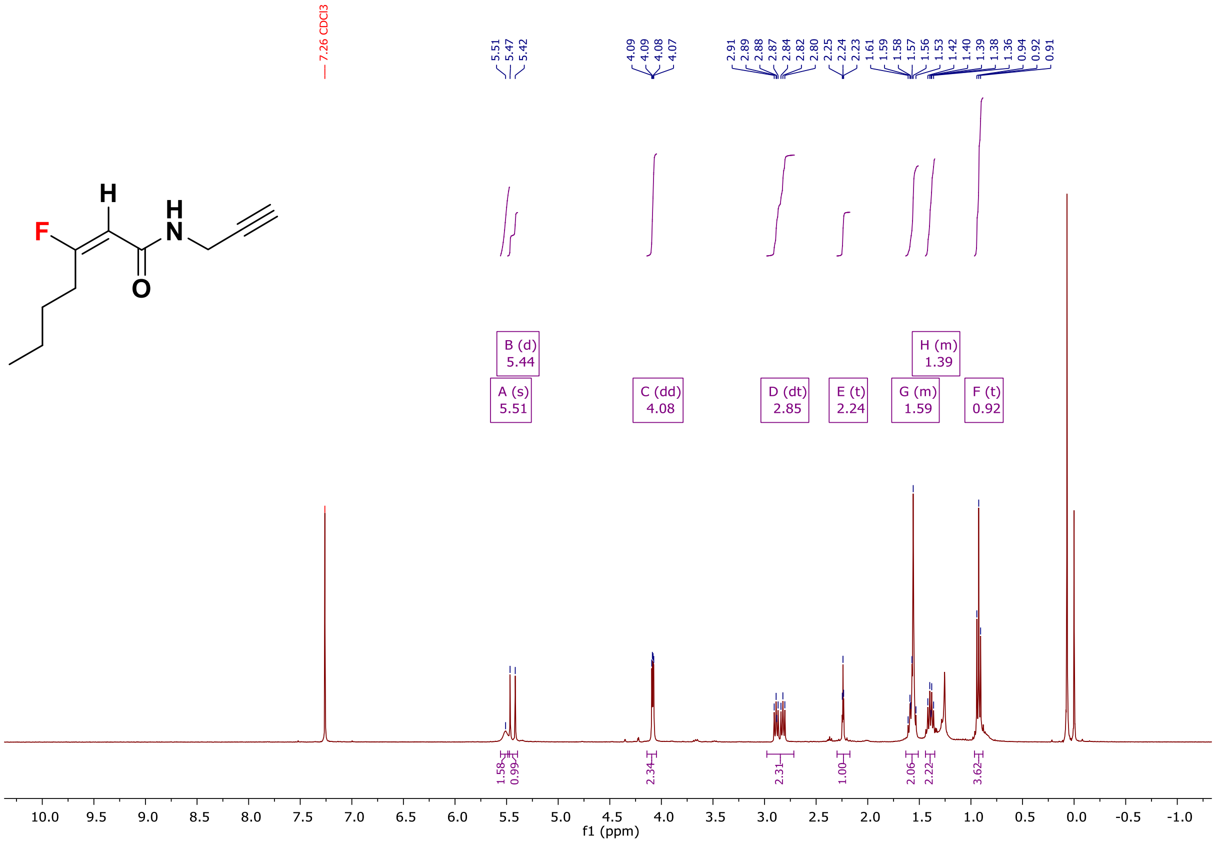

^{1}**H NMR** of (*E*)-3-fluoro-*N*-(prop-2-yn-1-yl)hept-2-enamide **11d**

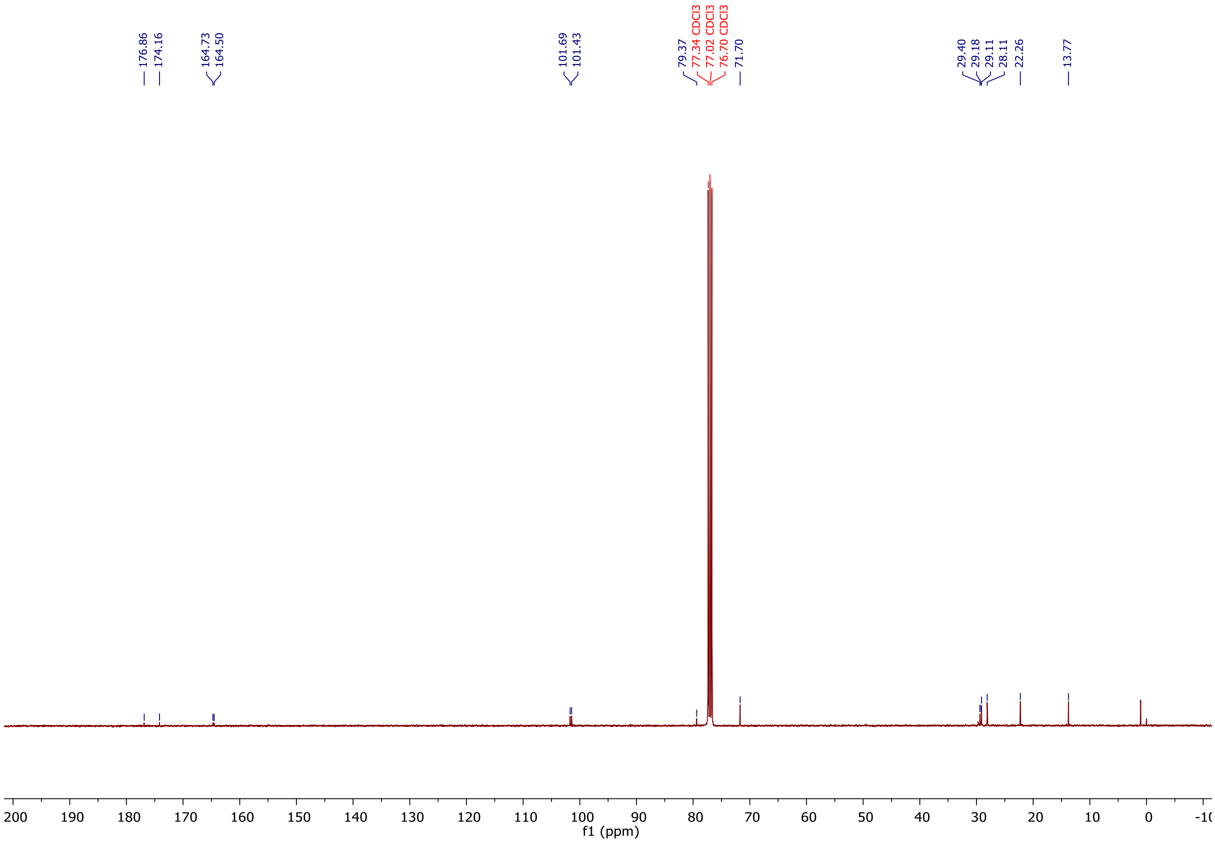

^{13}**C NMR** of (*E*)-3-fluoro-*N*-(prop-2-yn-1-yl)hept-2-enamide **11d**

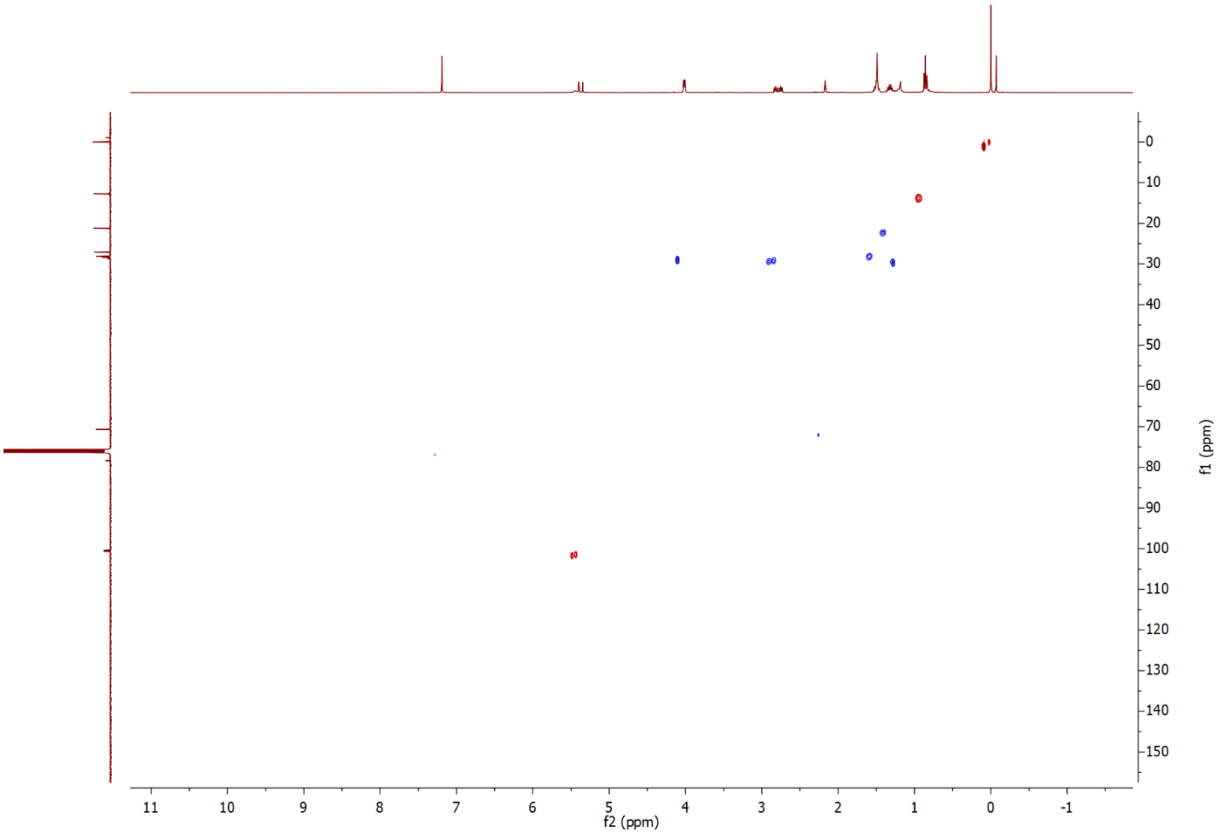

^{1}H-^{13}C HSQC of (*E*)-3-fluoro-*N*-(prop-2-yn-1-yl)hept-2-enamide **11d**

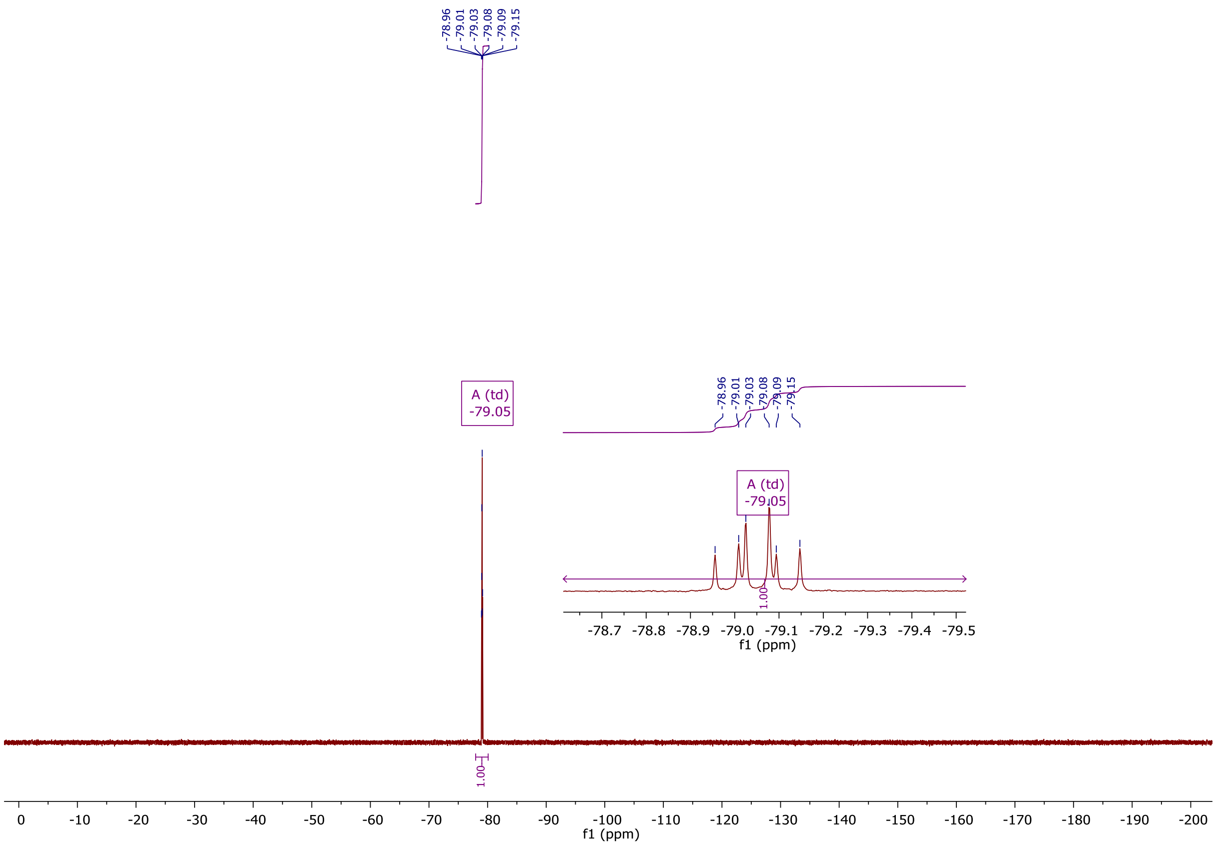

^{19}F NMR of (*E*)-3-fluoro-*N*-(prop-2-yn-1-yl)hept-2-enamide **11d**

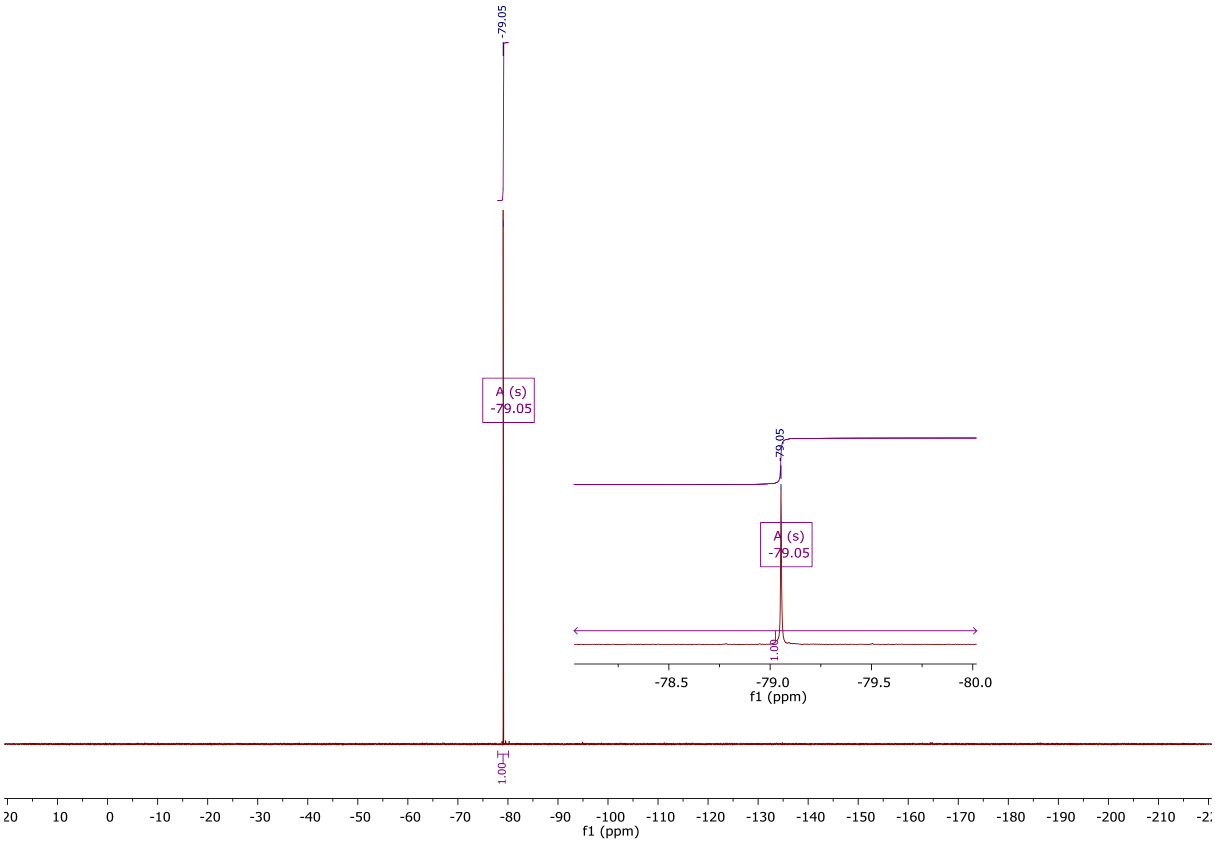

^{19}F{/^{1}H} NMR of (*E*)-3-fluoro-*N*-(prop-2-yn-1-yl)hept-2-enamide **11d**

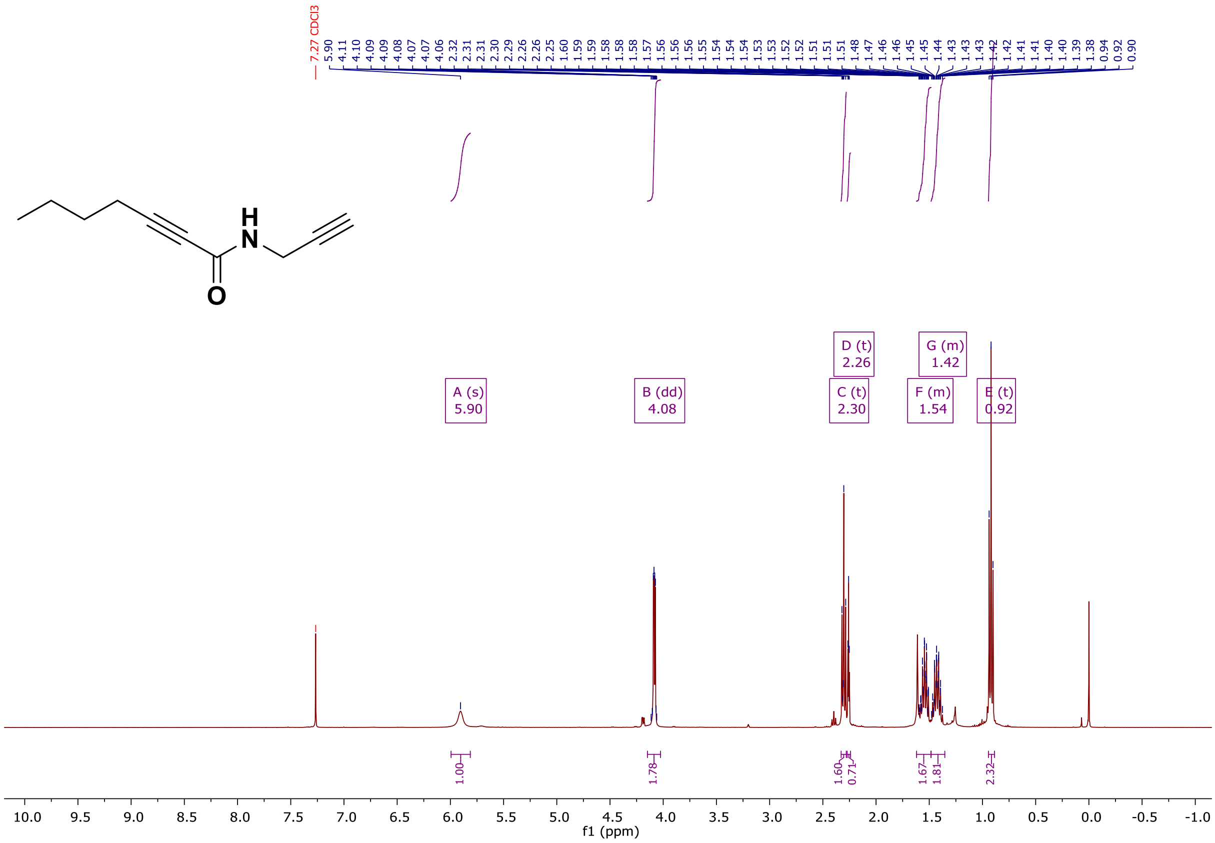

^{1}H NMR of *N*-(prop-2-yn-1-yl)hept-2-ynamide **12d**

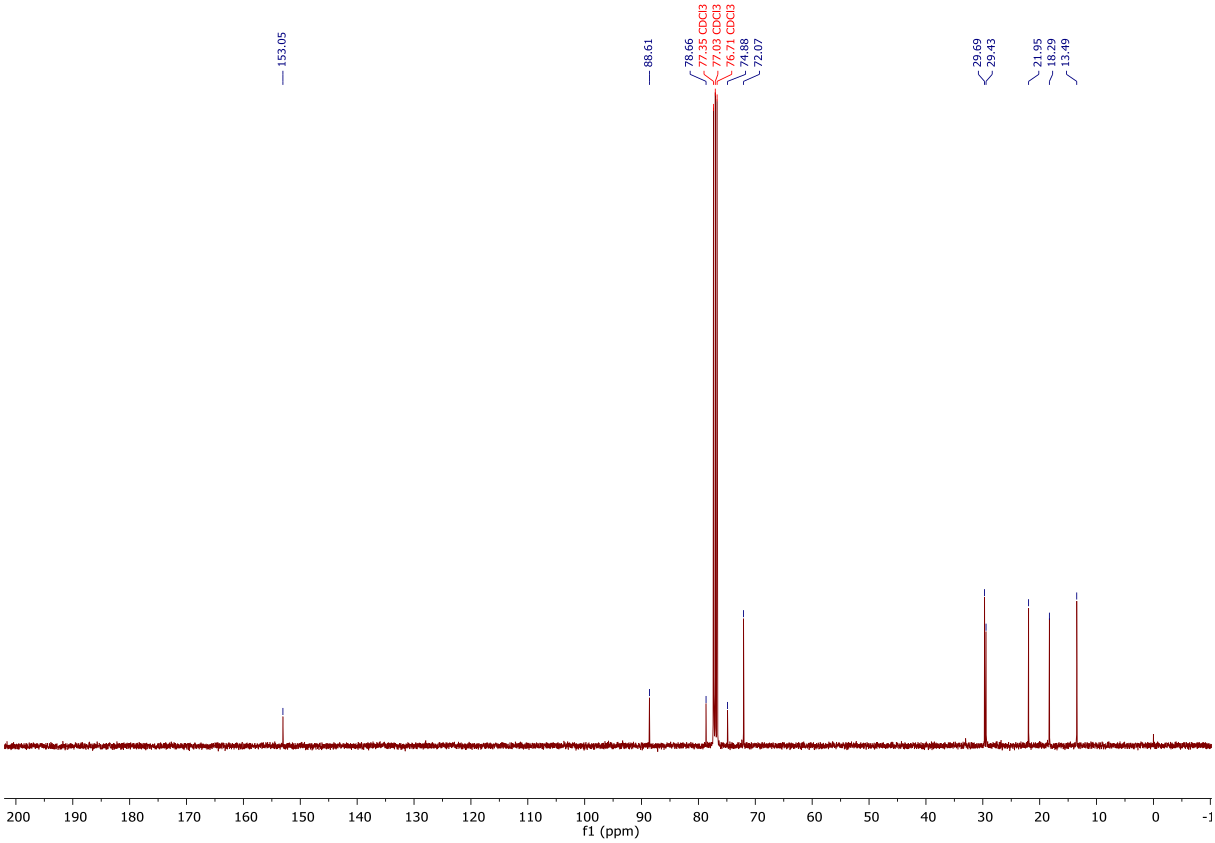

^{13}C NMR of *N*-(prop-2-yn-1-yl)hept-2-ynamide **12d**

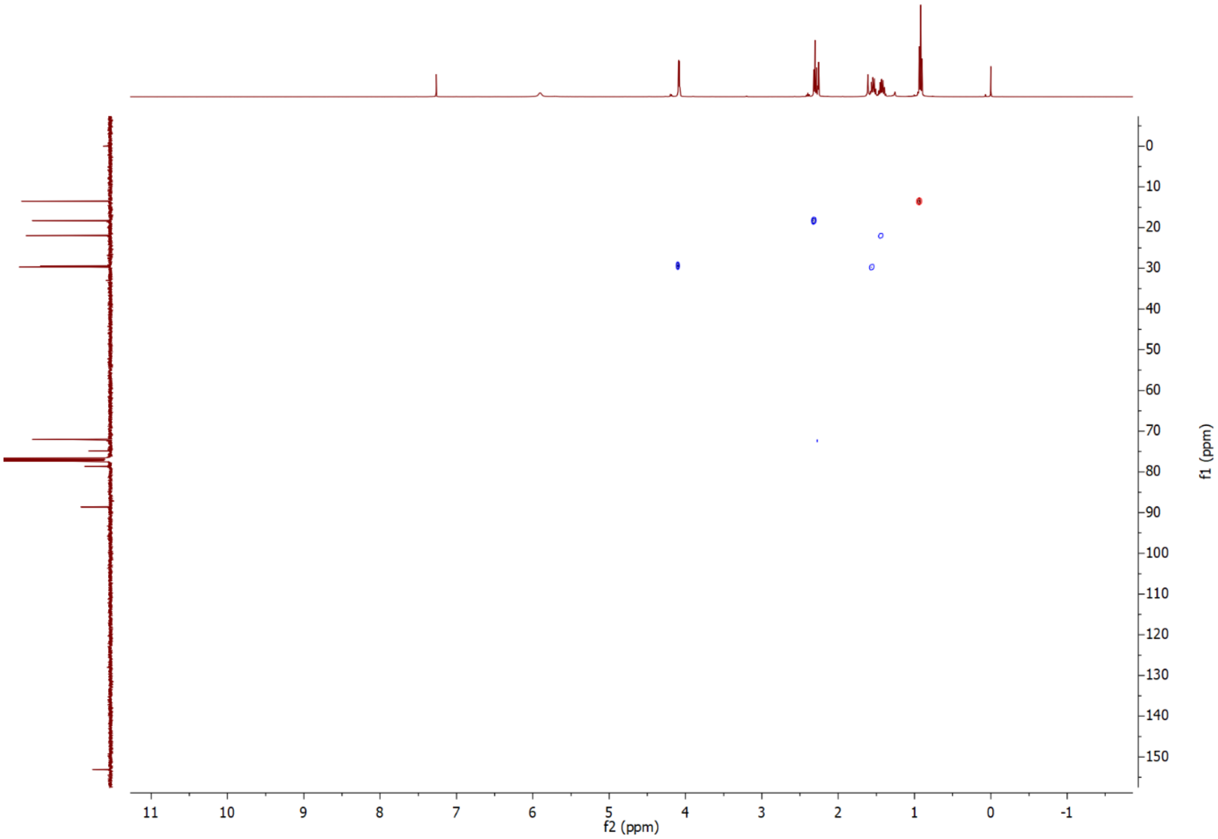

^{1}H-^{13}C HSQC of *N*-(prop-2-yn-1-yl)hept-2-ynamide **12d**

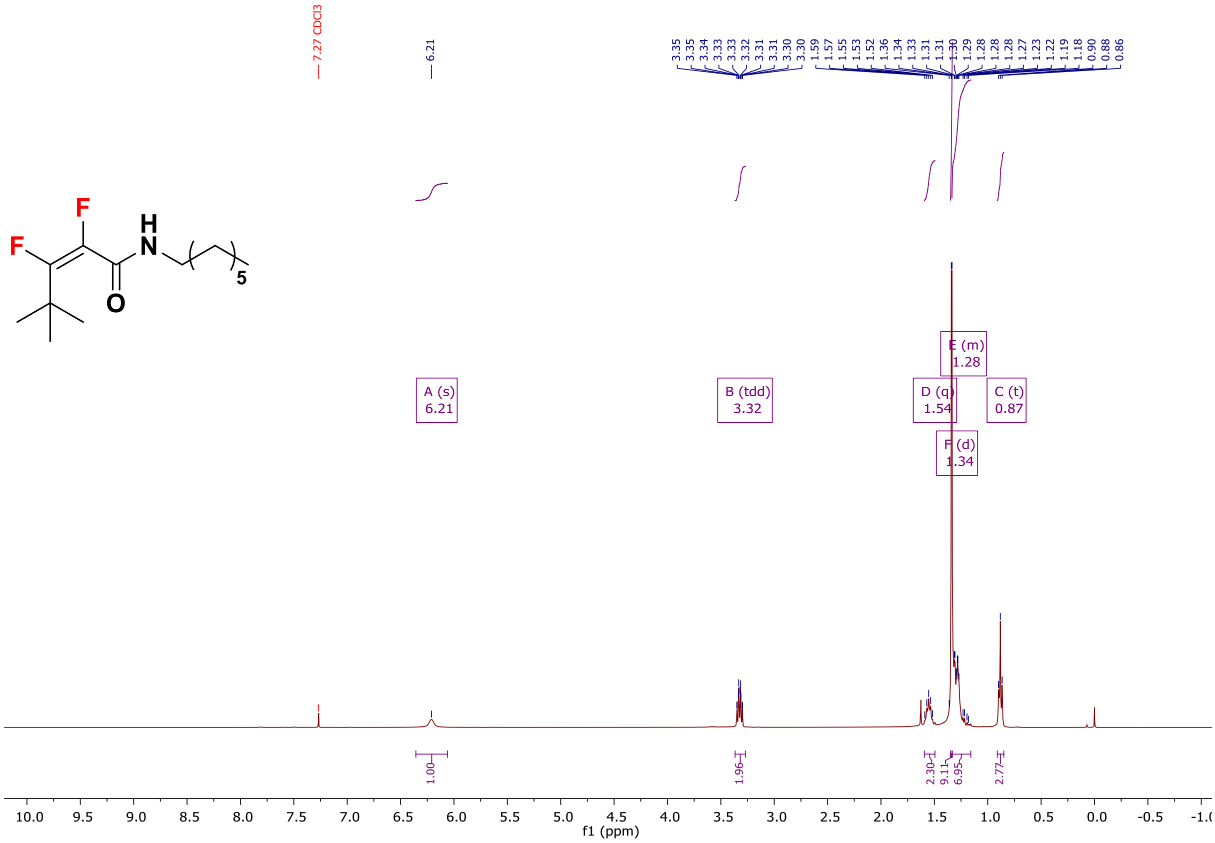

^{1}H NMR of (*Z*)-2,3-difluoro-*N*-heptyl-4,4-dimethylpent-2-enamide **13a**

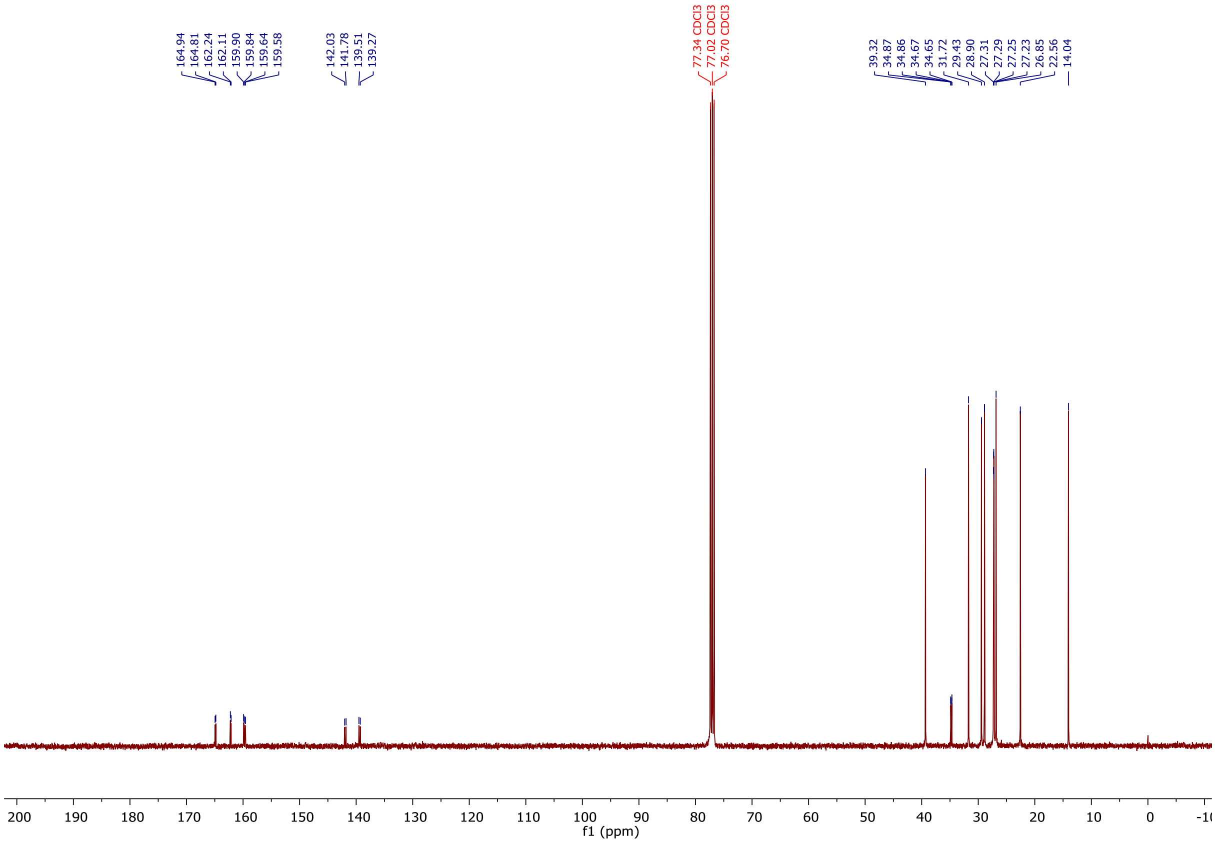

^{13}C NMR of (*Z*)-2,3-difluoro-*N*-heptyl-4,4-dimethylpent-2-enamide **13a**

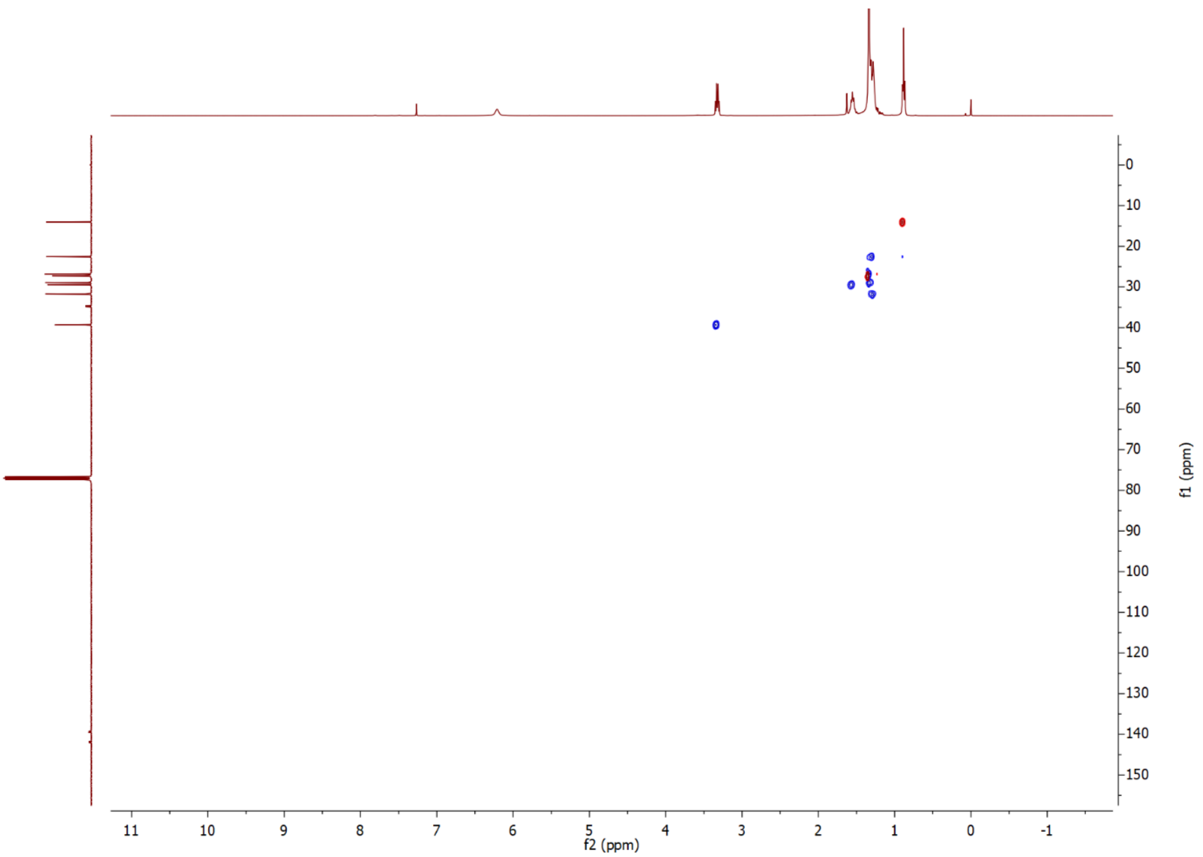

^{1}H-^{13}C HSQC of (*Z*)-2,3-difluoro-*N*-heptyl-4,4-dimethylpent-2-enamide **13a**

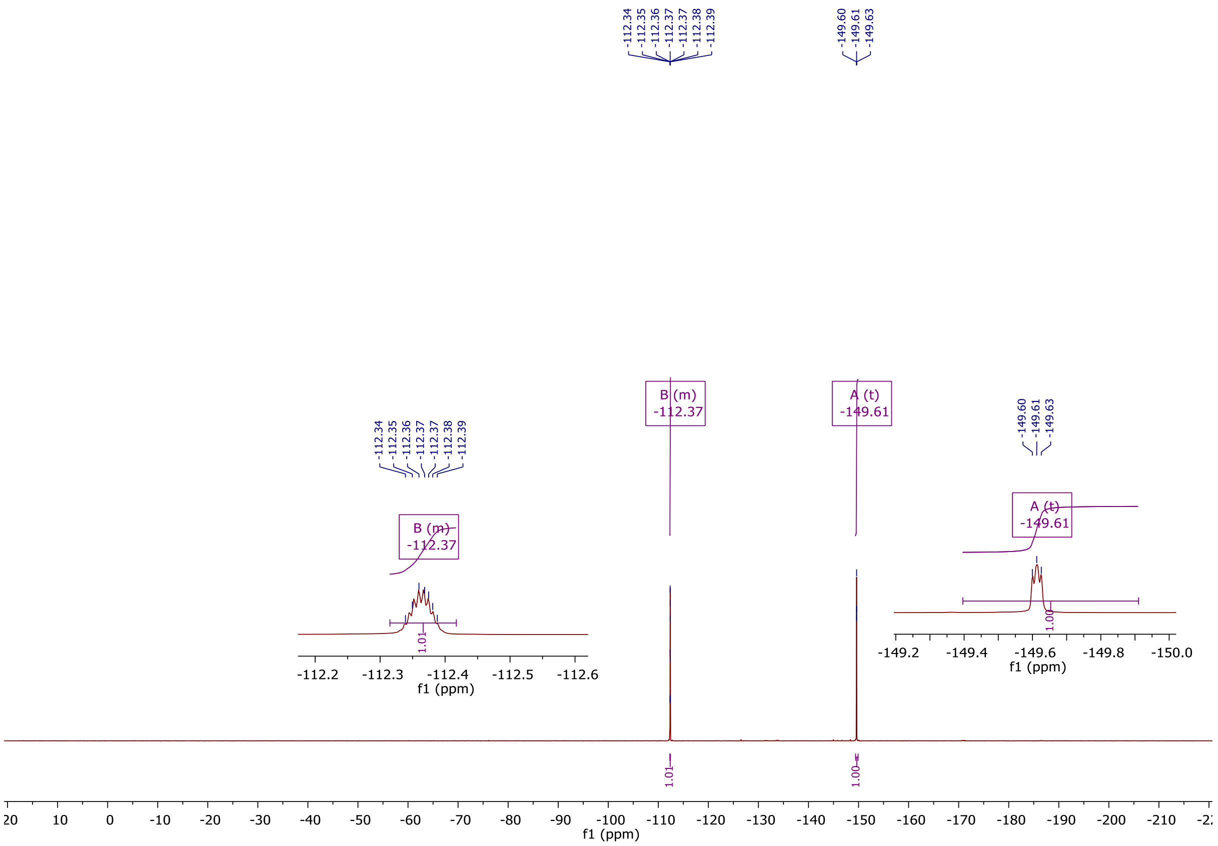

^{19}F NMR of (*Z*)-2,3-difluoro-*N*-heptyl-4,4-dimethylpent-2-enamide **13a**

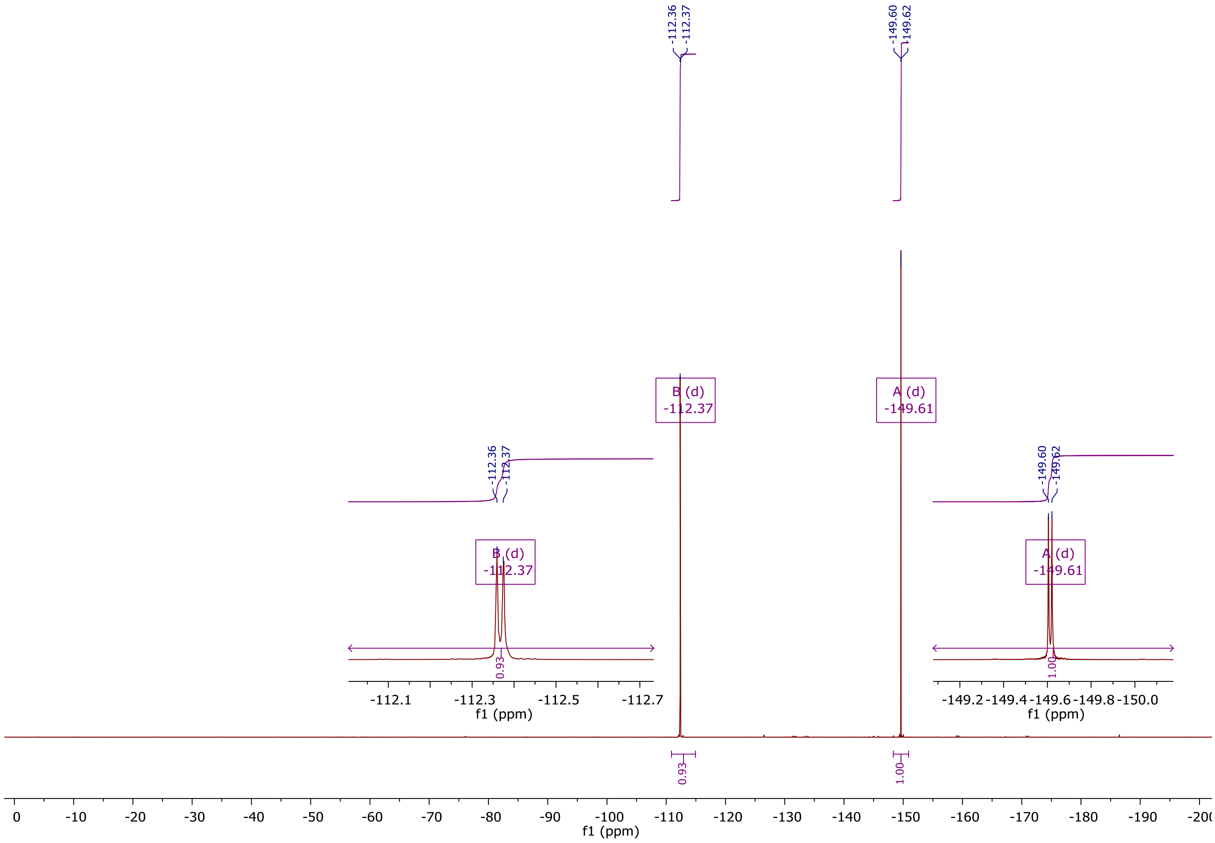

^{19}**F{/^{1}H} NMR** of (*Z*)-2,3-difluoro-*N*-heptyl-4,4-dimethylpent-2-enamide **13a**

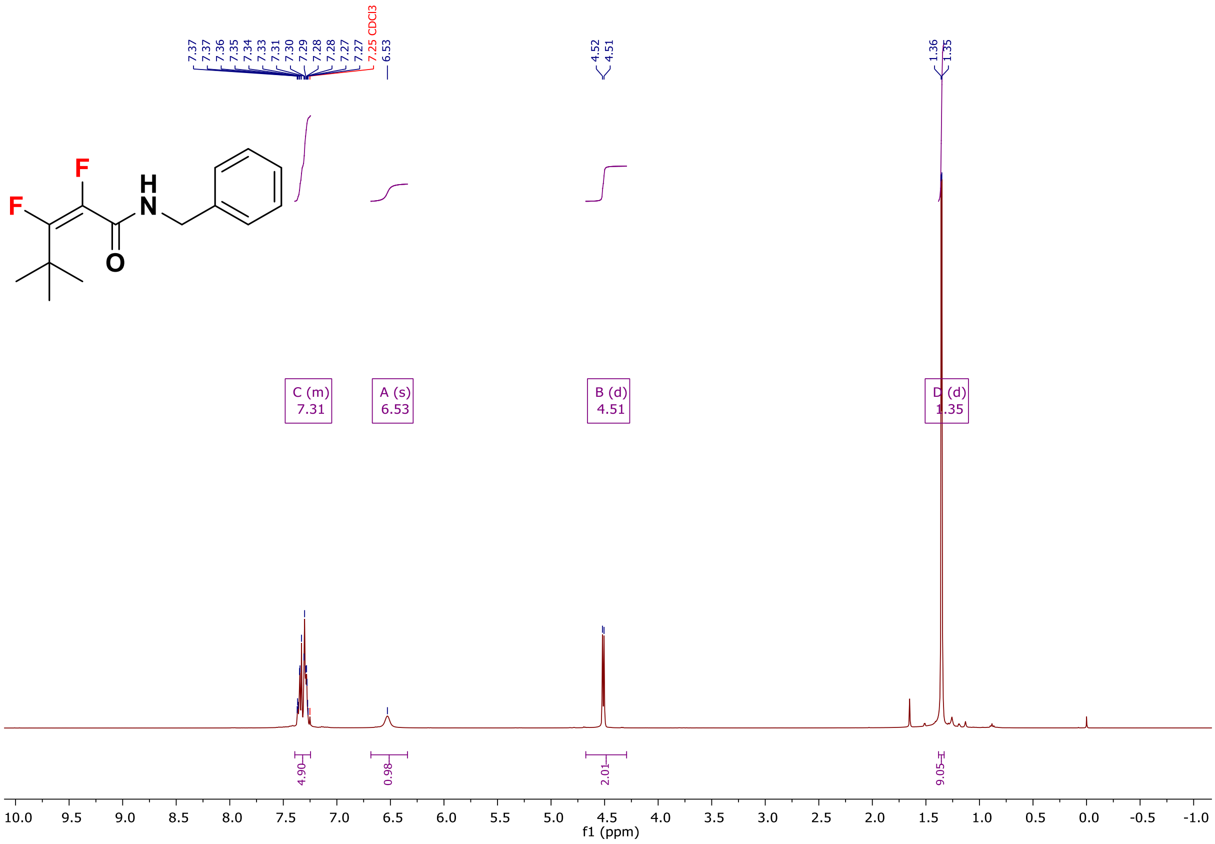

^{1}**H NMR** of (*Z*)-*N*-benzyl-2,3-difluoro-4,4-dimethylpent-2-enamide **13b**

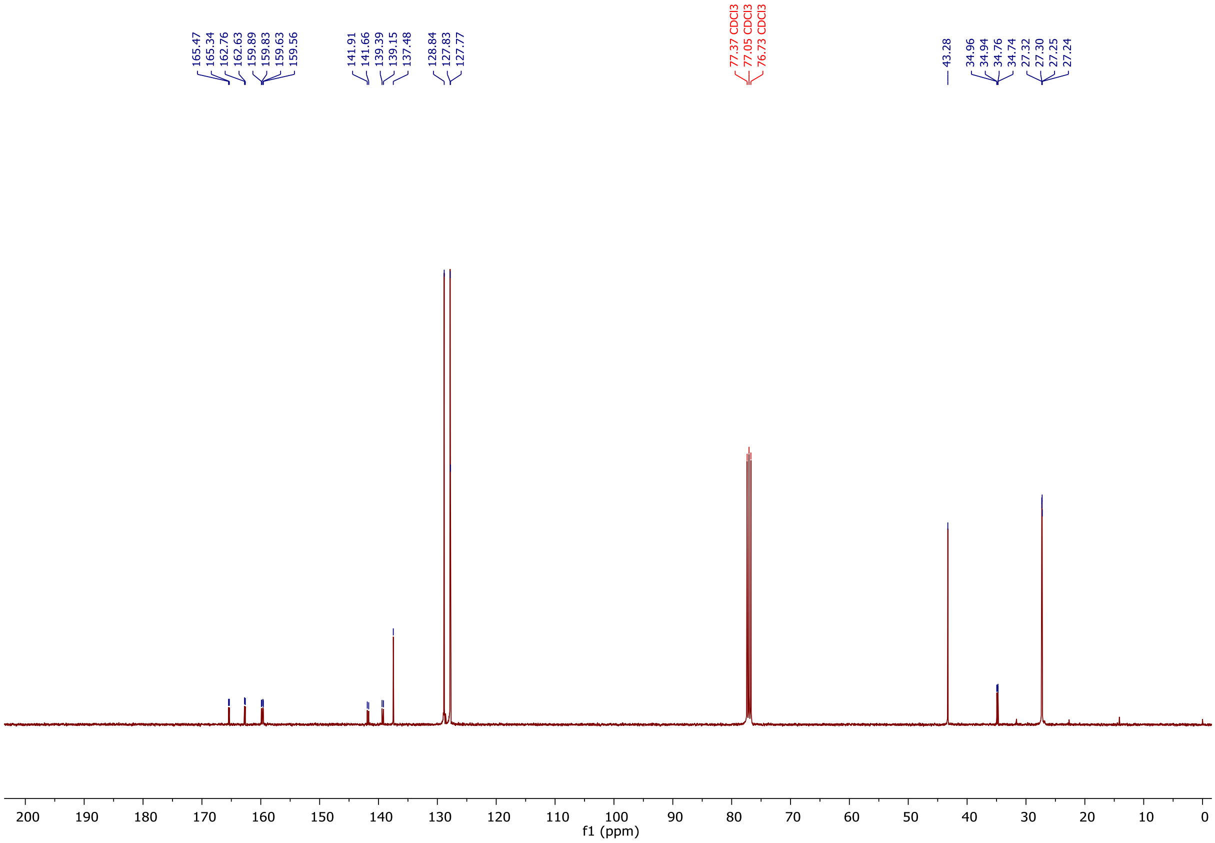

^{13}**C NMR** of (*Z*)-*N*-benzyl-2,3-difluoro-4,4-dimethylpent-2-enamide **13b**

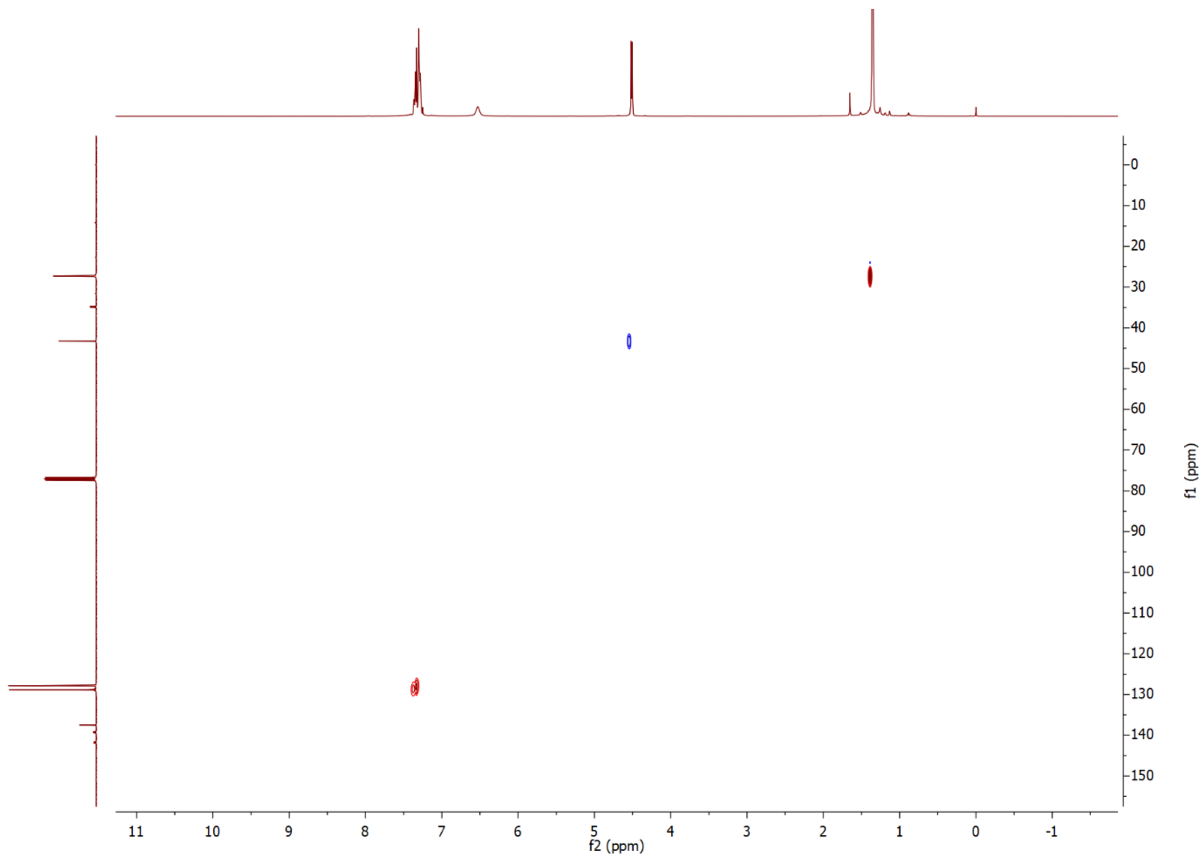

^{1}**H-**^{13}**C HSQC** of (*Z*)-*N*-benzyl-2,3-difluoro-4,4-dimethylpent-2-enamide **13b**

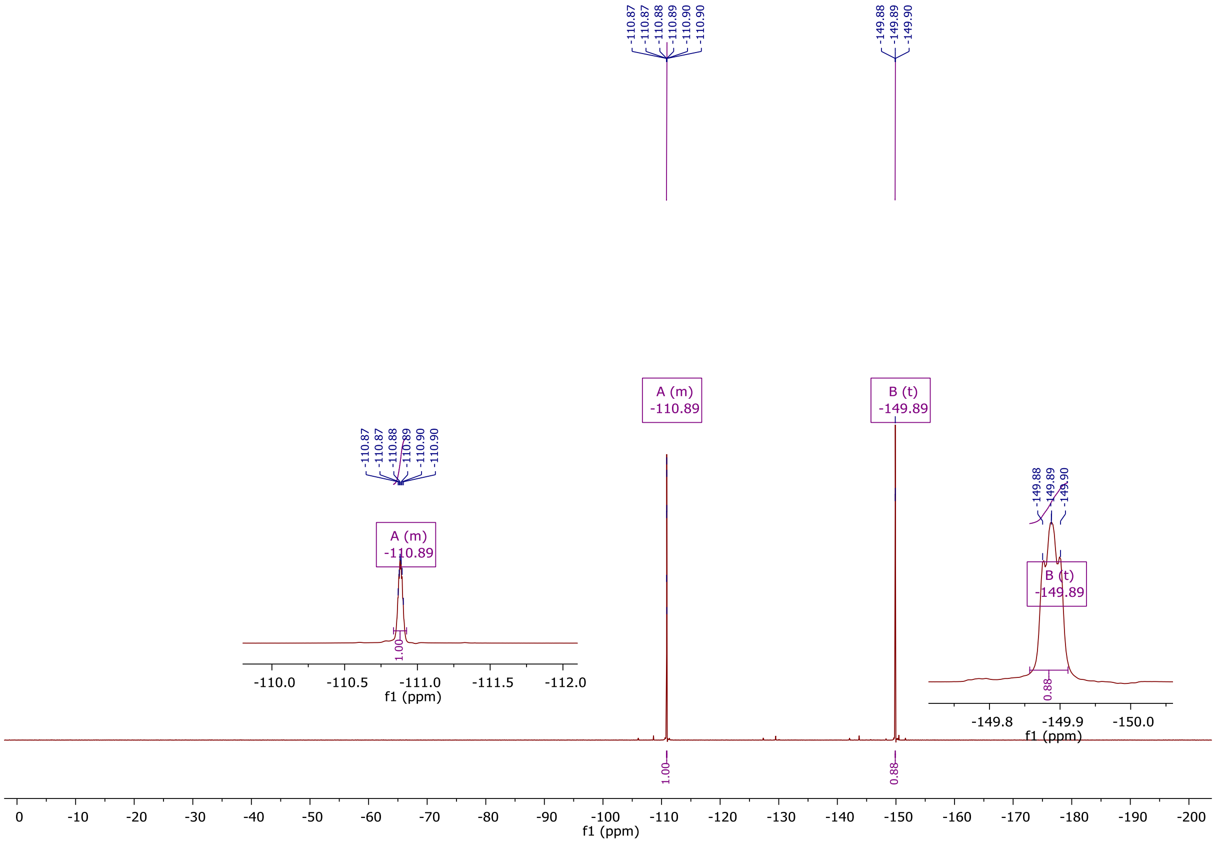

^{19}F NMR of (*Z*)-*N*-benzyl-2,3-difluoro-4,4-dimethylpent-2-enamide **13b**

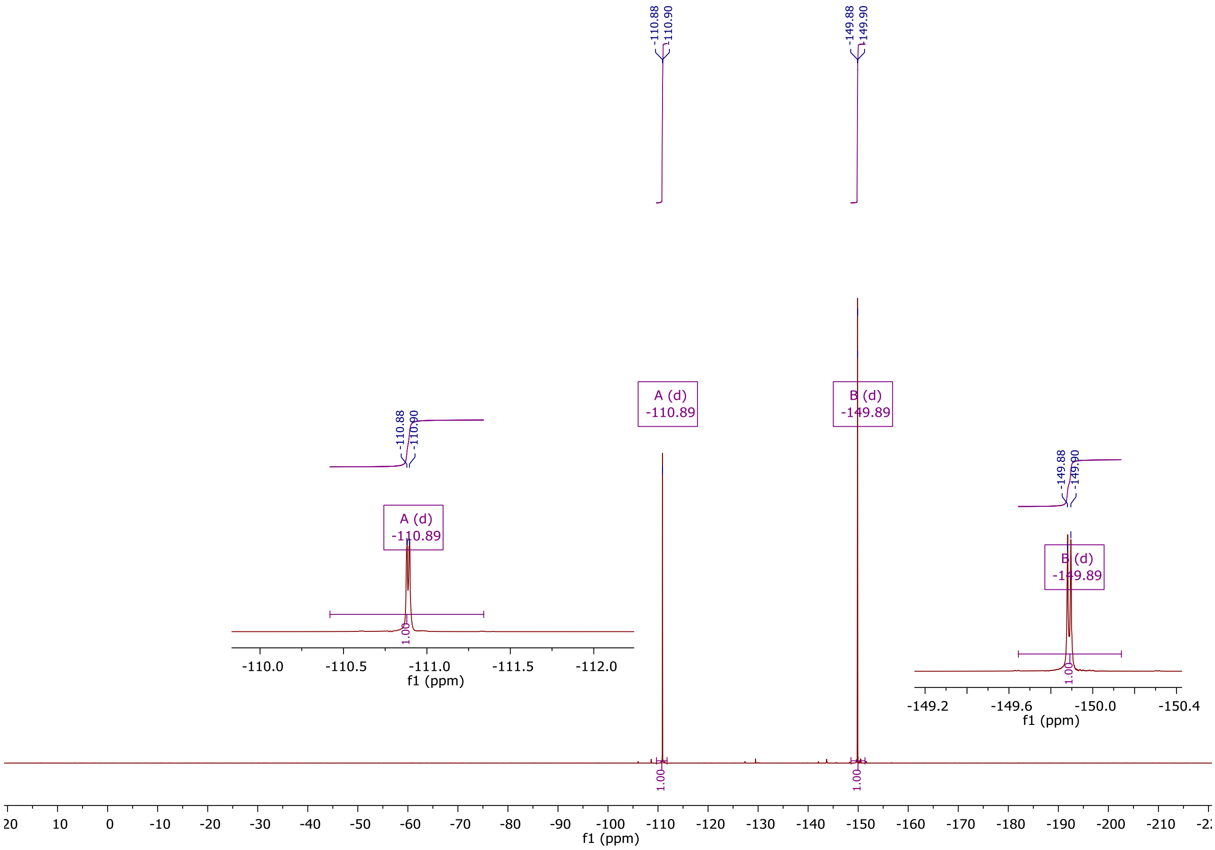

^{19}F{/^{1}H} NMR of (*Z*)-*N*-benzyl-2,3-difluoro-4,4-dimethylpent-2-enamide **13b**

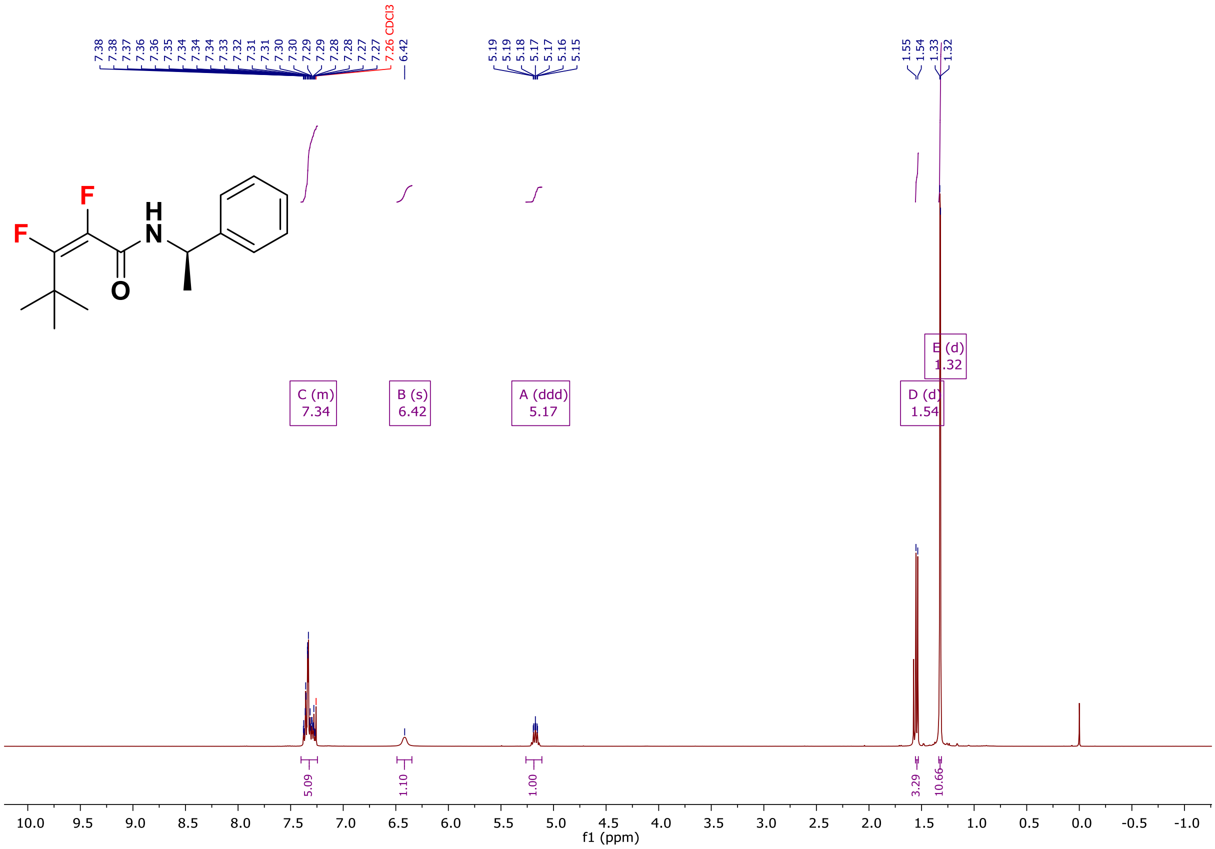

[1]**H NMR** of (*R*,*Z*)-2,3-difluoro-4,4-dimethyl-*N*-(1-phenylethyl)pent-2-enamide **13c**

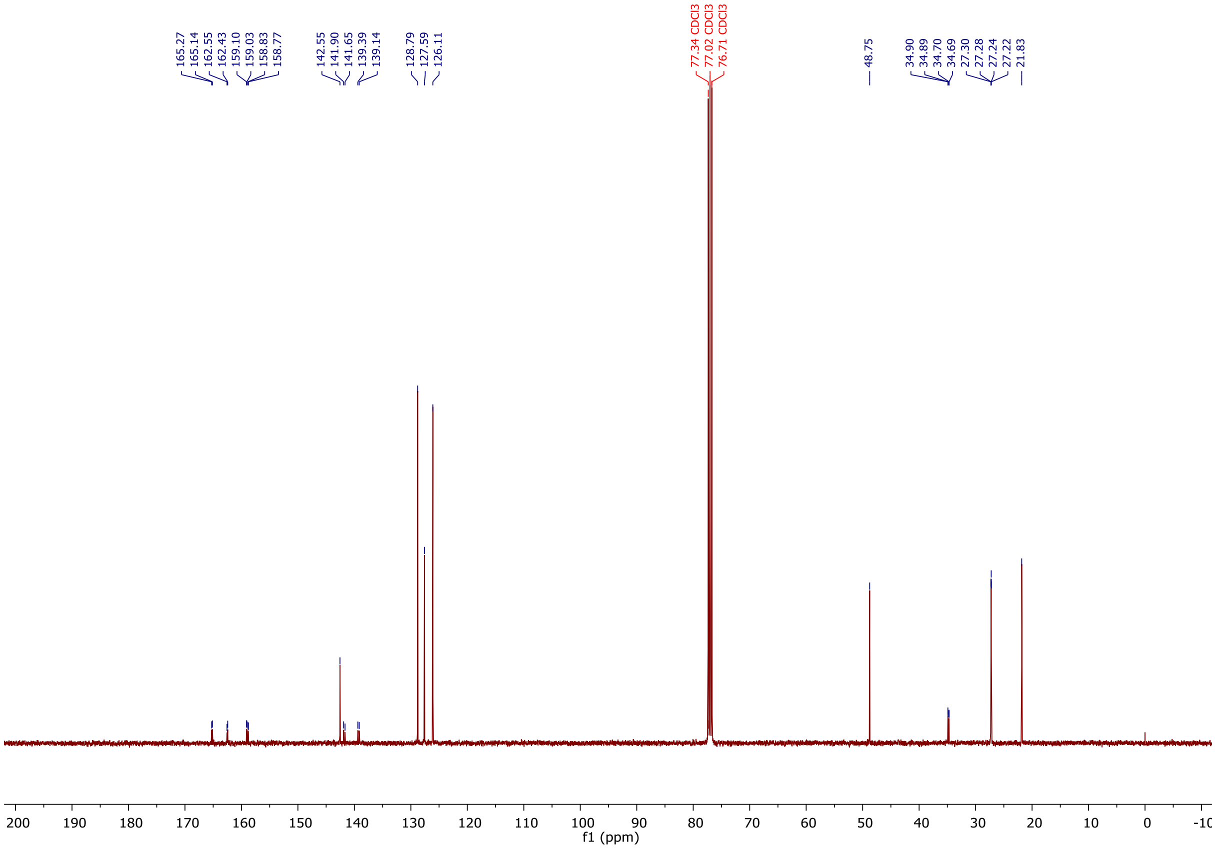

[13]**C NMR** of (*R*,*Z*)-2,3-difluoro-4,4-dimethyl-*N*-(1-phenylethyl)pent-2-enamide **13c**

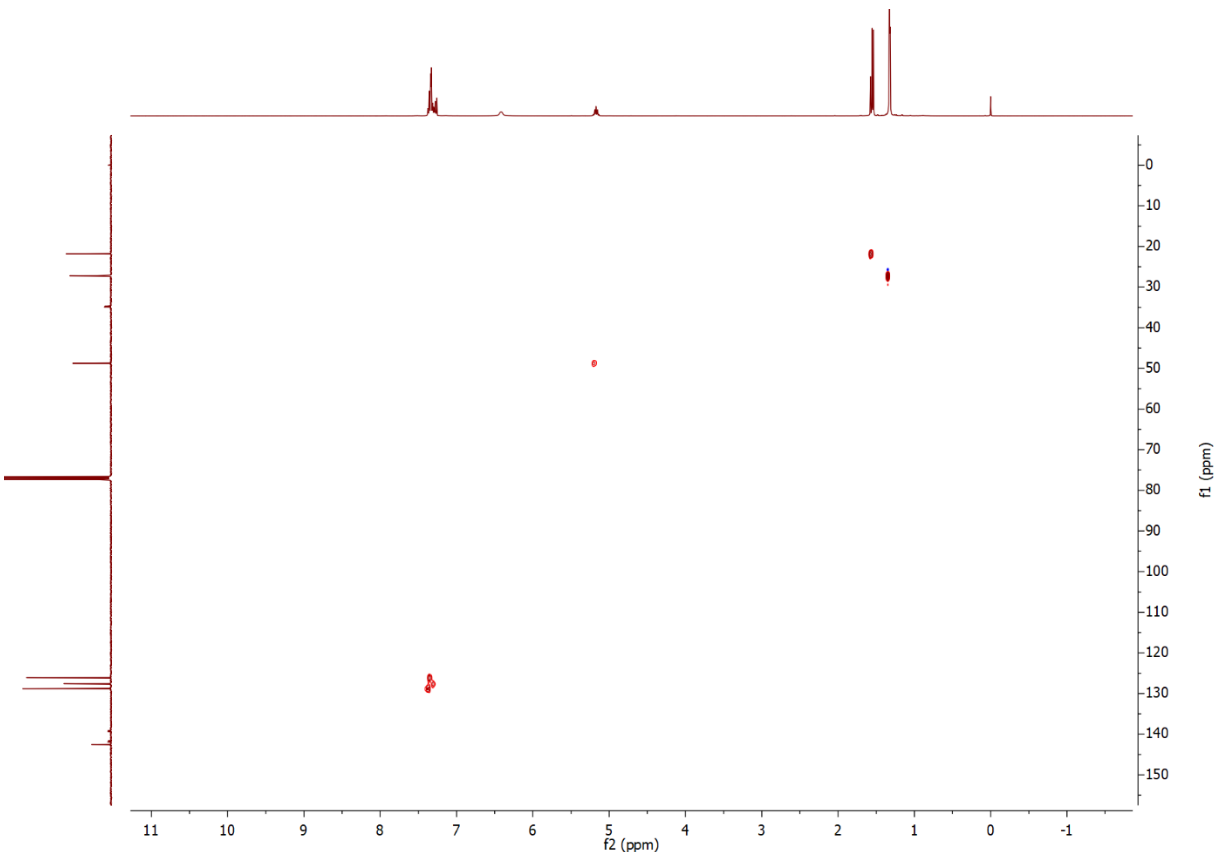

^{1}**H-**^{13}**C HSQC** of (*R*,*Z*)-2,3-difluoro-4,4-dimethyl-*N*-(1-phenylethyl)pent-2-enamide **13c**

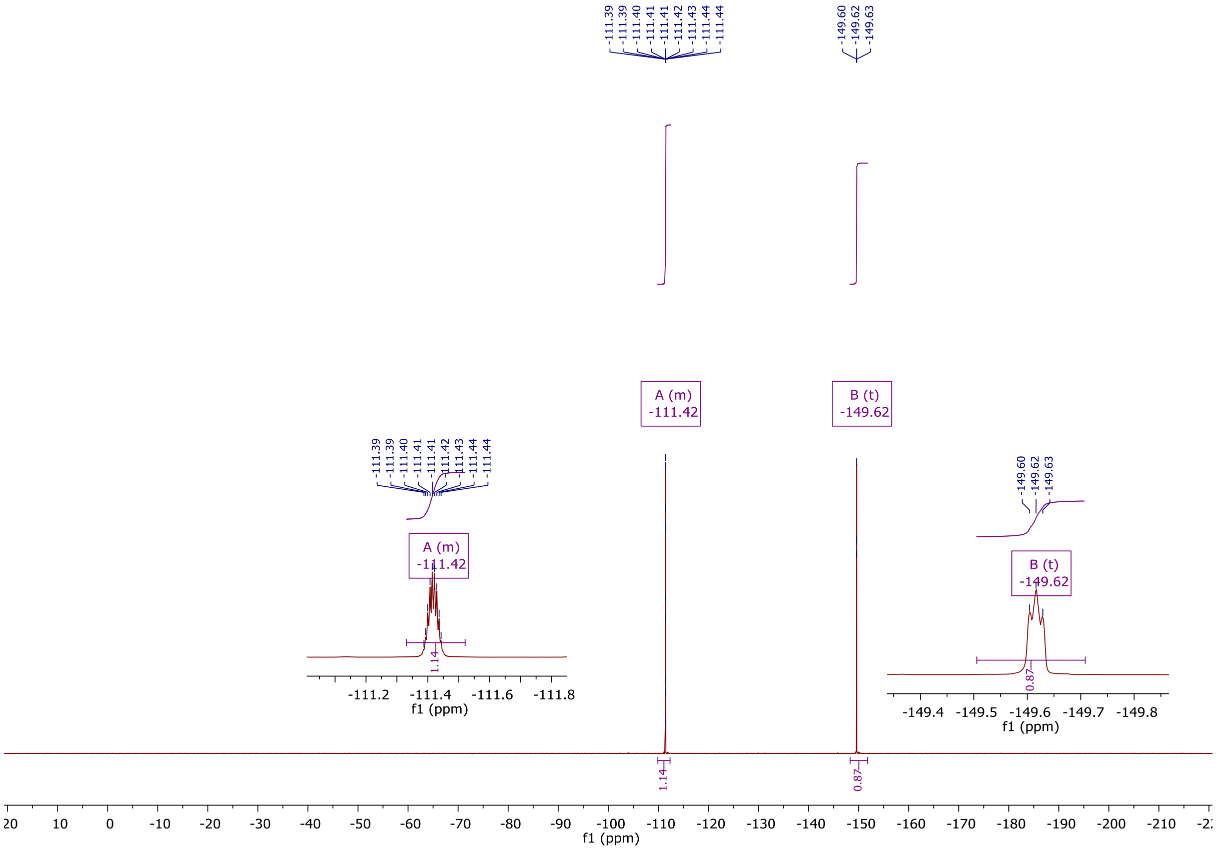

^{19}**F NMR** of (*R*,*Z*)-2,3-difluoro-4,4-dimethyl-*N*-(1-phenylethyl)pent-2-enamide **13c**

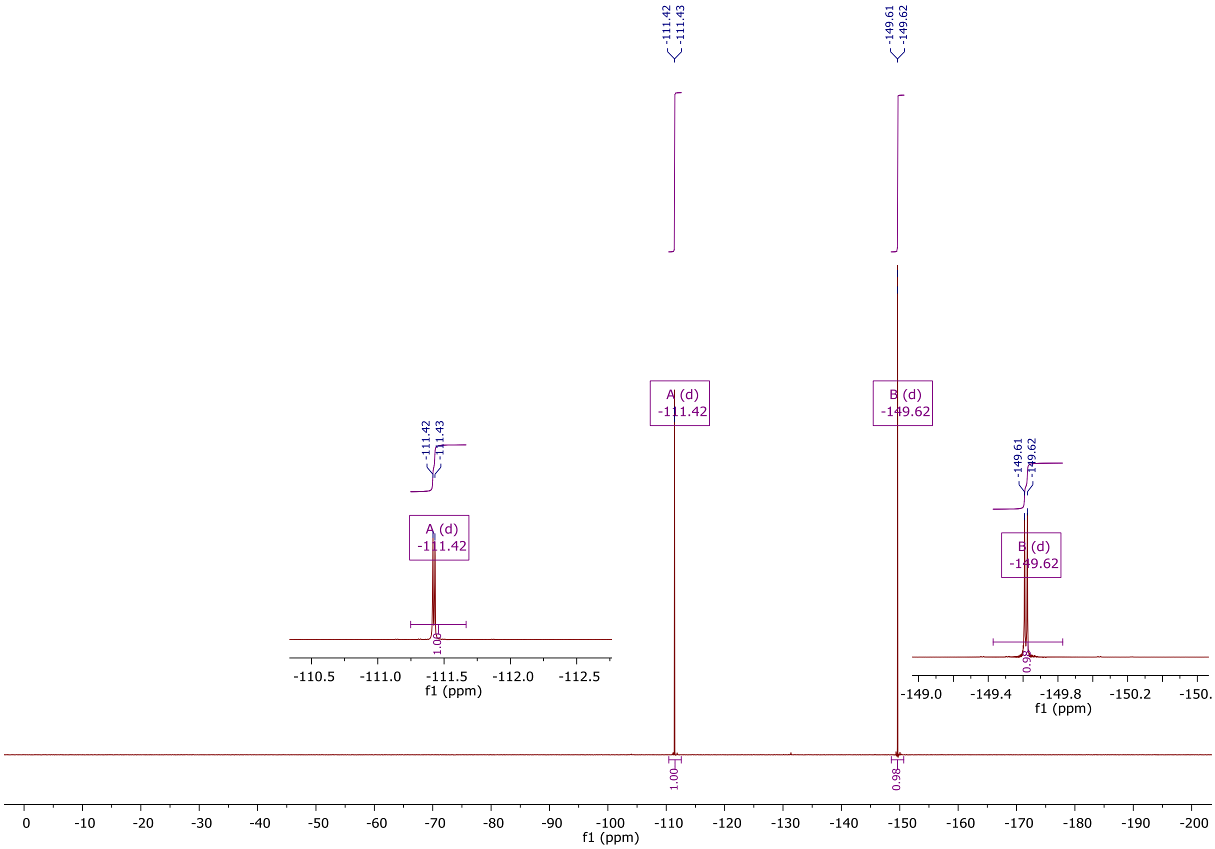

^{19}**F{/^{1}H} NMR** of (*R*,*Z*)-2,3-difluoro-4,4-dimethyl-*N*-(1-phenylethyl)pent-2-enamide **13c**

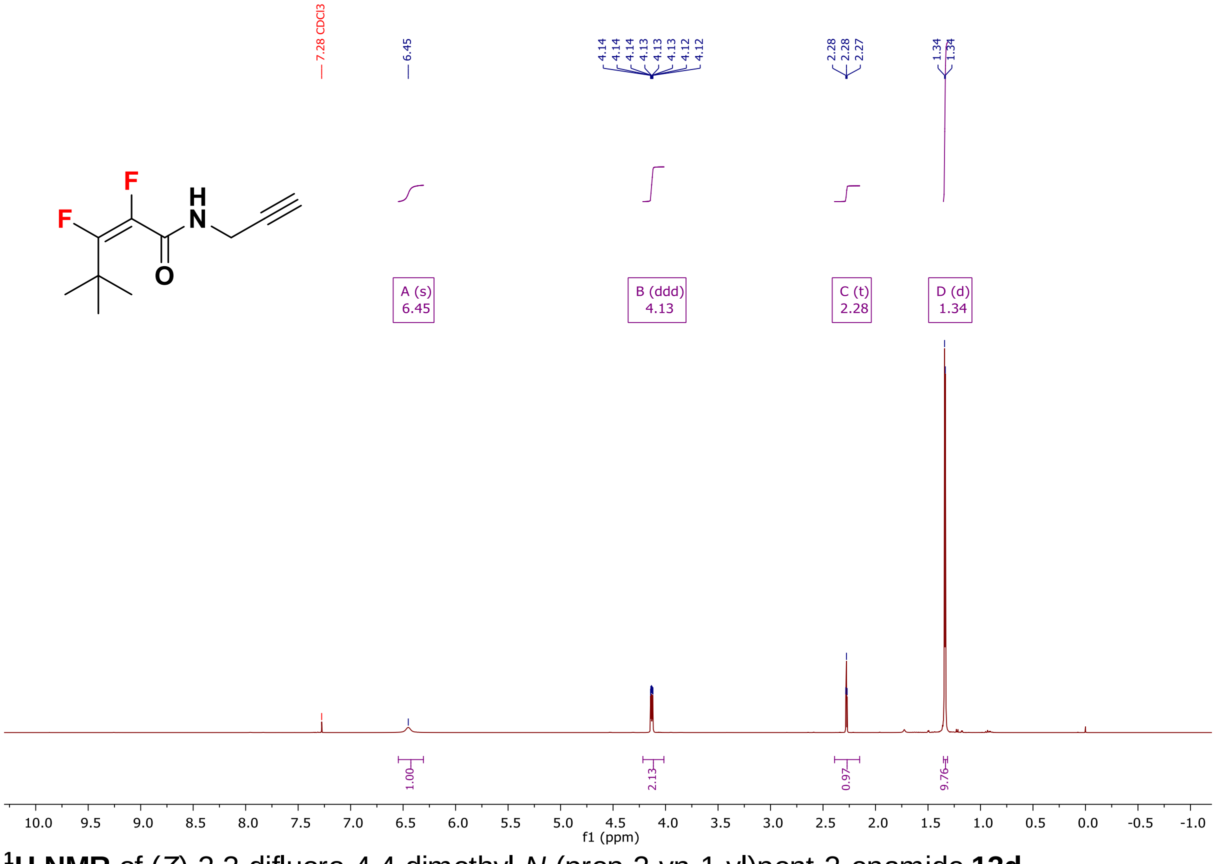

^{1}**H NMR** of (*Z*)-2,3-difluoro-4,4-dimethyl-*N*-(prop-2-yn-1-yl)pent-2-enamide **13d**

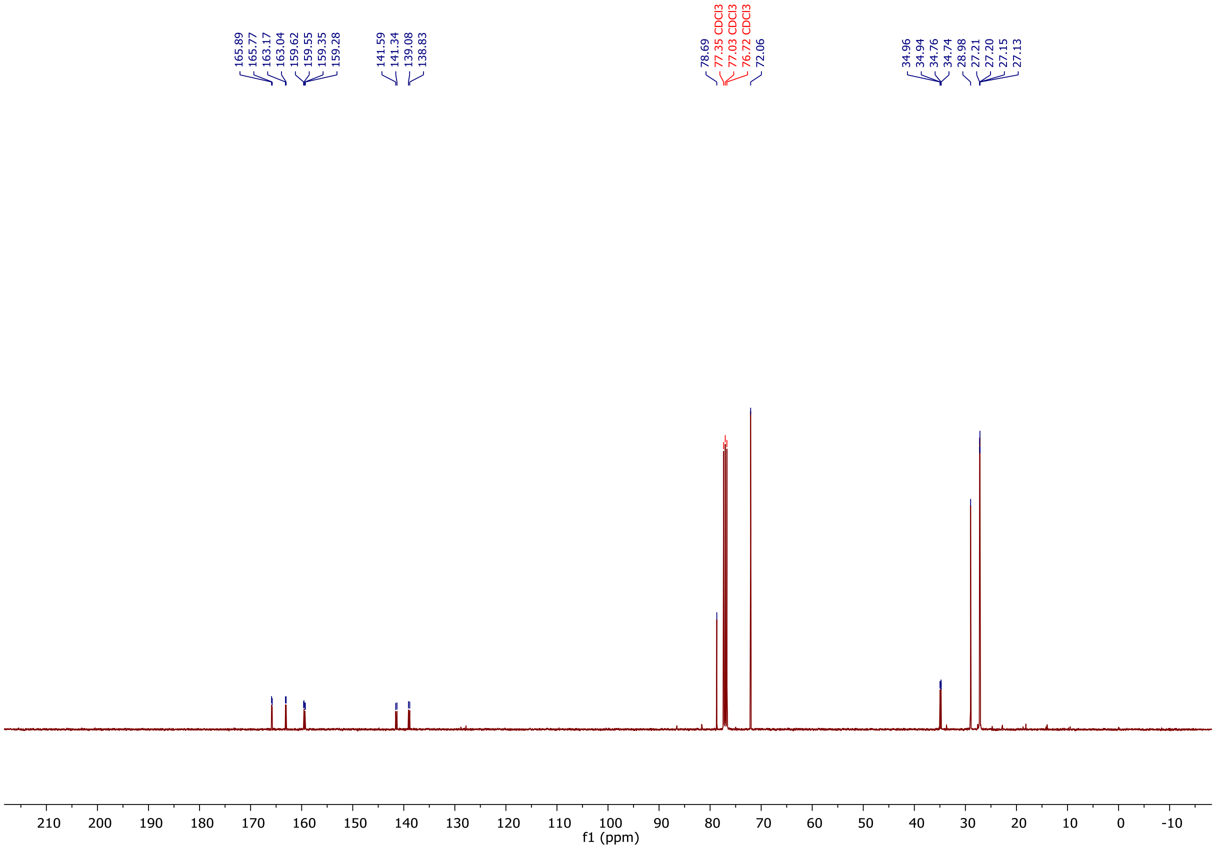

^{13}C NMR of (*Z*)-2,3-difluoro-4,4-dimethyl-*N*-(prop-2-yn-1-yl)pent-2-enamide **13d**

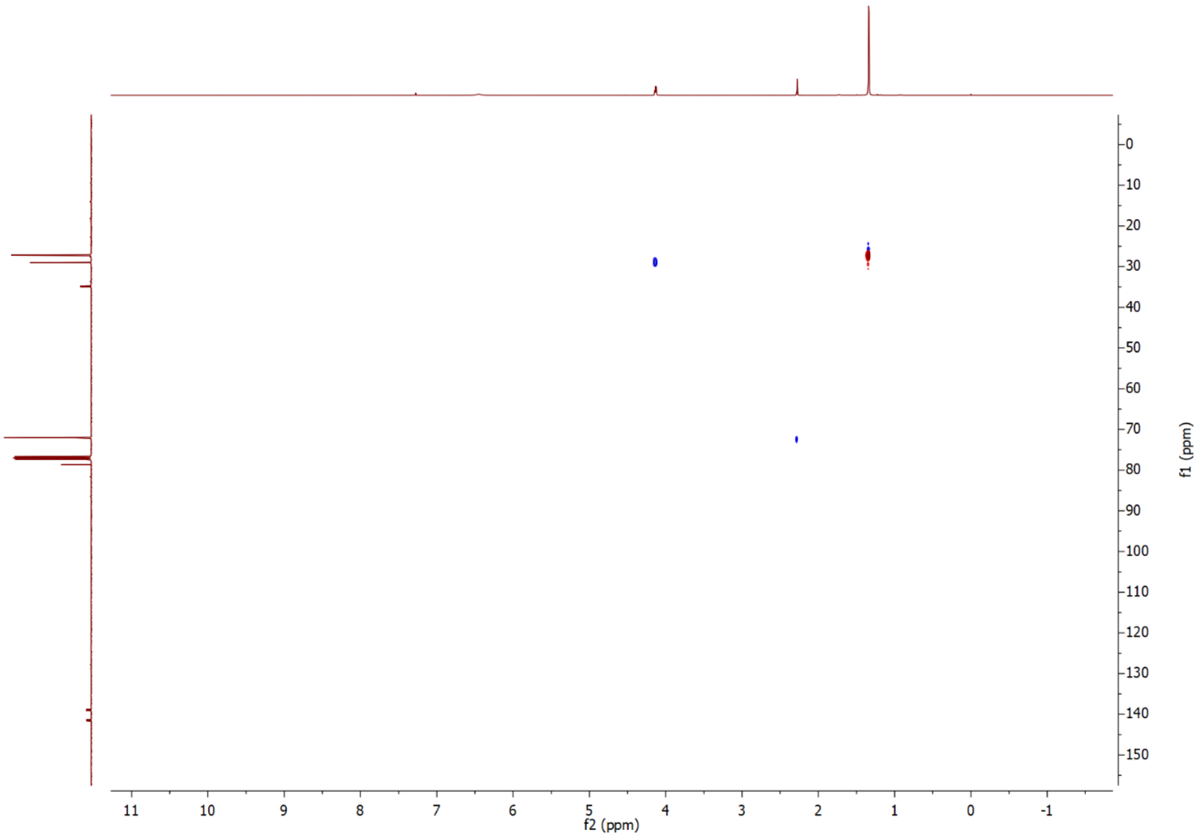

^{1}H-^{13}C HSQC of (*Z*)-2,3-difluoro-4,4-dimethyl-*N*-(prop-2-yn-1-yl)pent-2-enamide **13d**

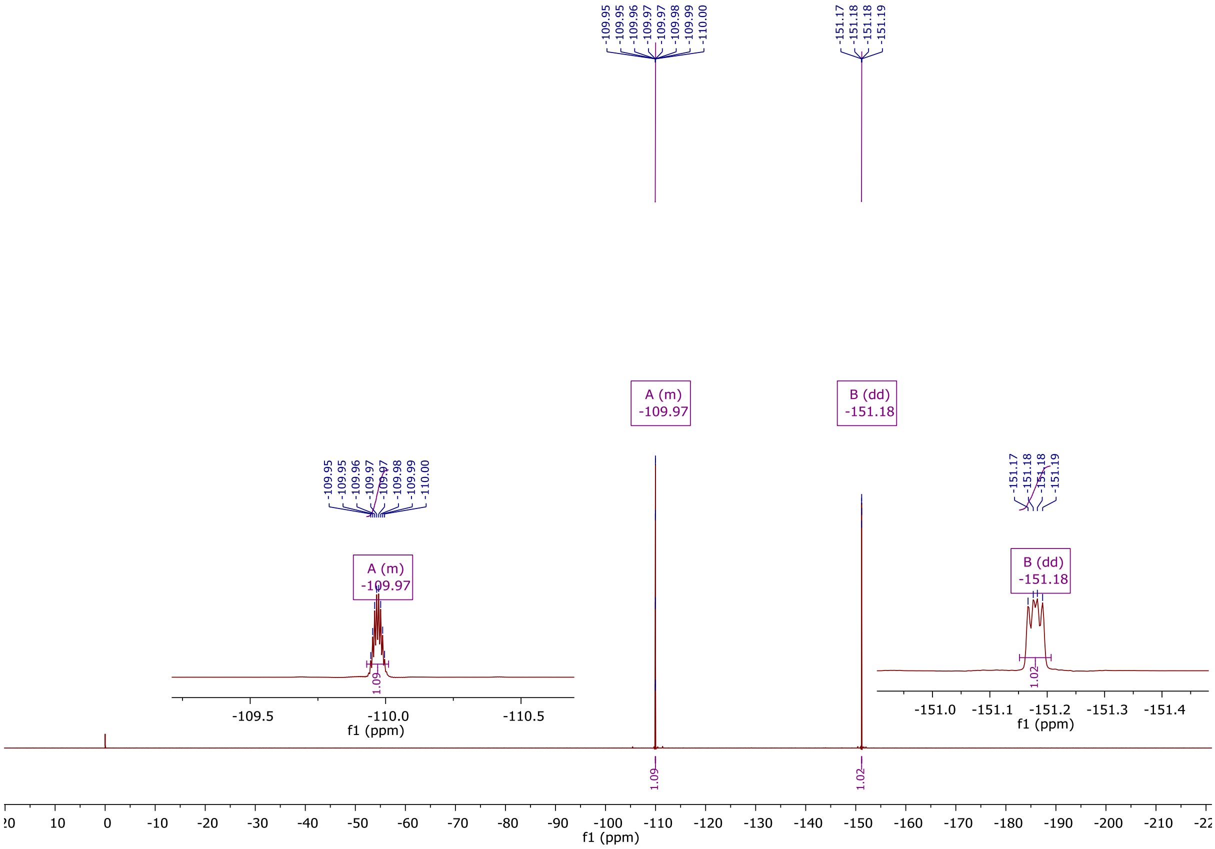

^{19}F NMR of (*Z*)-2,3-difluoro-4,4-dimethyl-*N*-(prop-2-yn-1-yl)pent-2-enamide **13d**

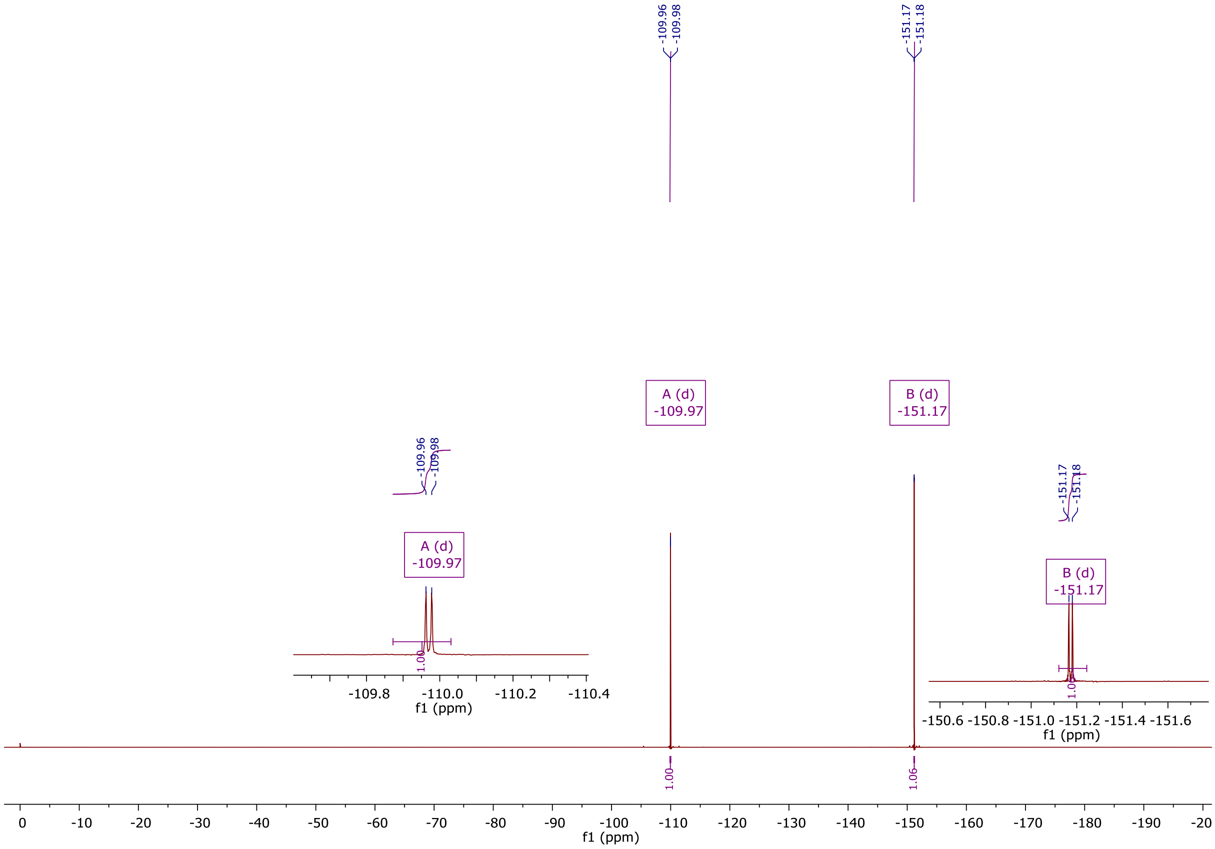

^{19}F{/^{1}H} NMR of (*Z*)-2,3-difluoro-4,4-dimethyl-*N*-(prop-2-yn-1-yl)pent-2-enamide **13d**

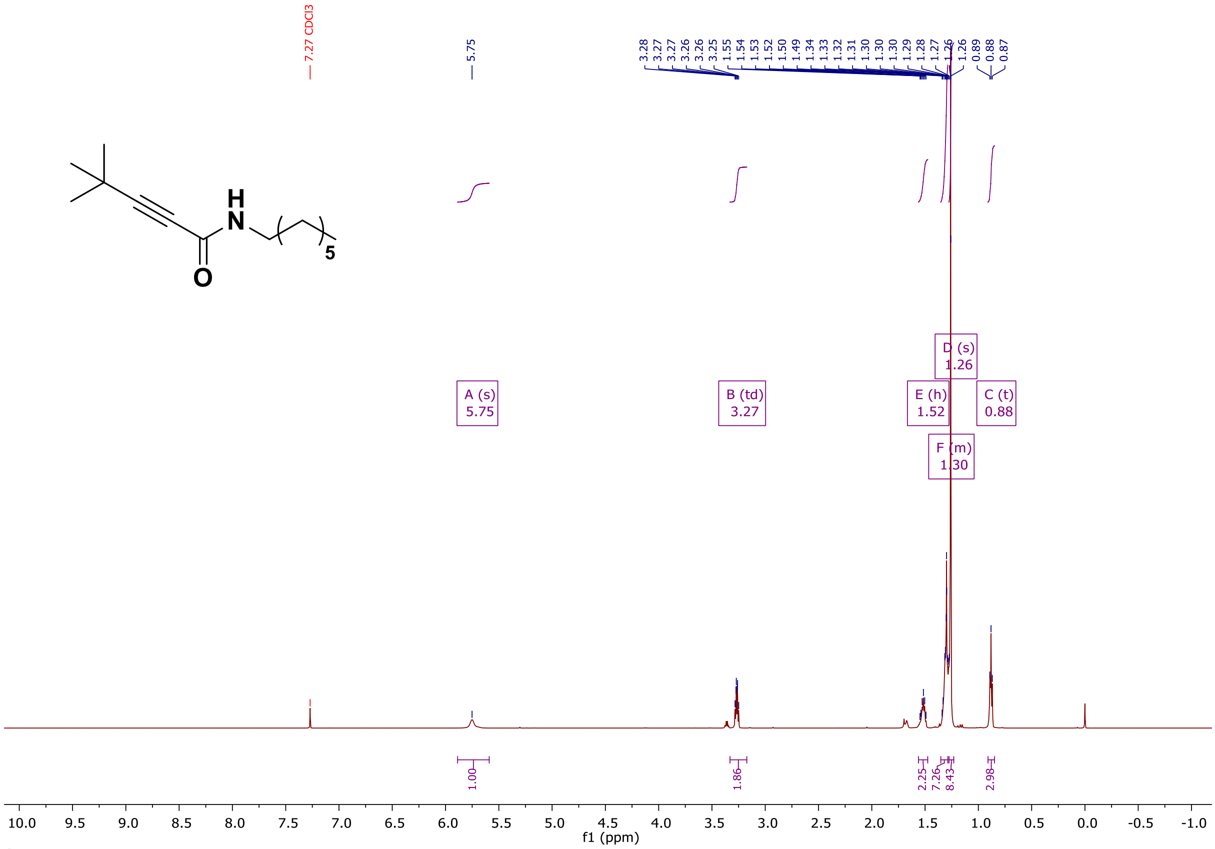

^{1}H NMR of *N*-heptyl-4,4-dimethylpent-2-ynamide **14a**

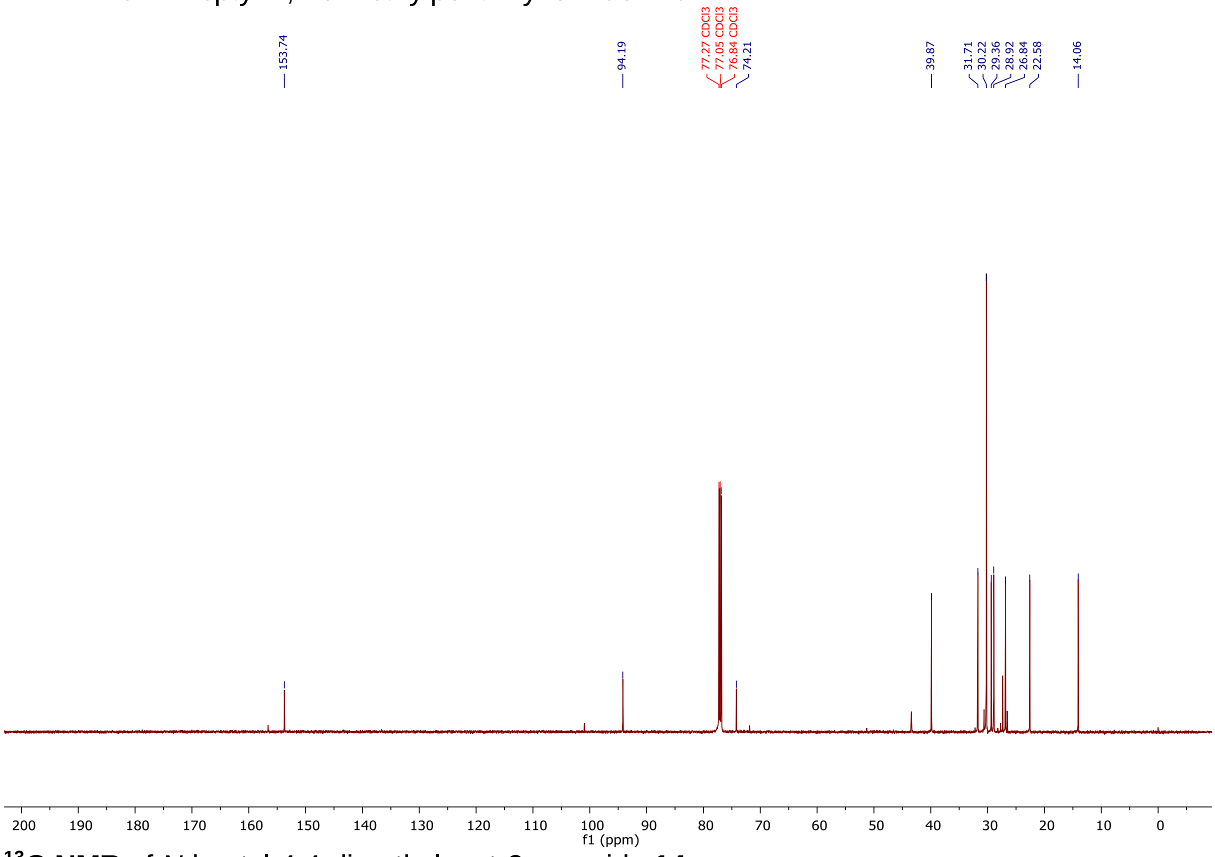

^{13}C NMR of *N*-heptyl-4,4-dimethylpent-2-ynamide **14a**

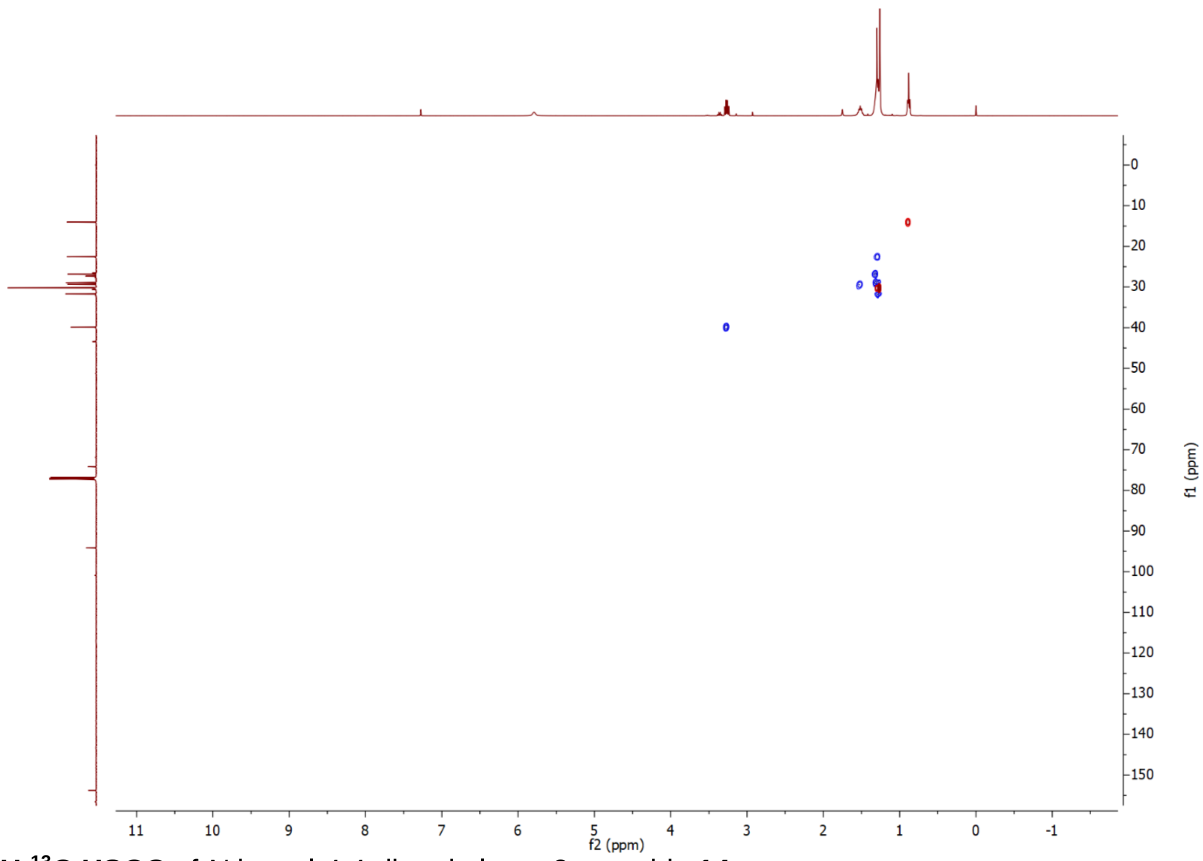

^{1}**H-**^{13}**C HSQC** of *N*-heptyl-4,4-dimethylpent-2-ynamide **14a**

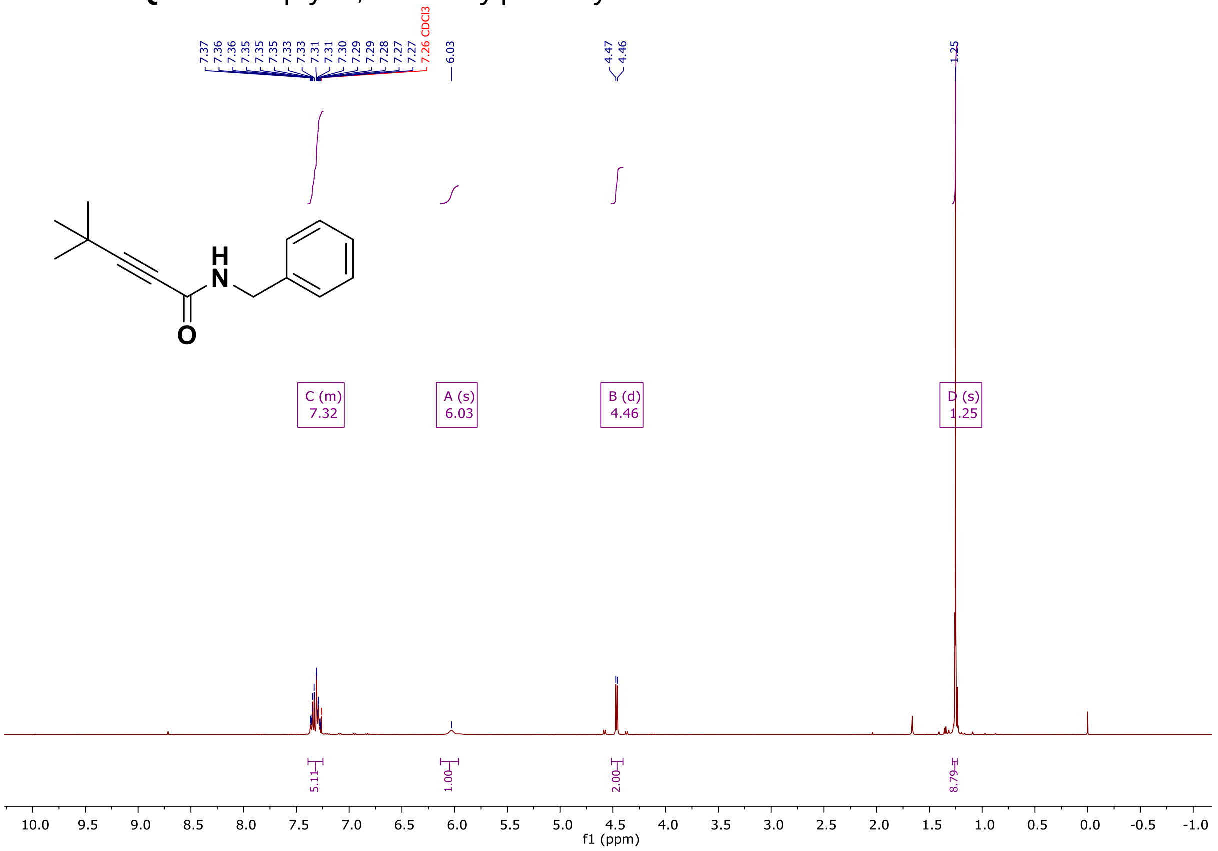

^{1}**H NMR** of *N*-benzyl-4,4-dimethylpent-2-ynamide **14b**

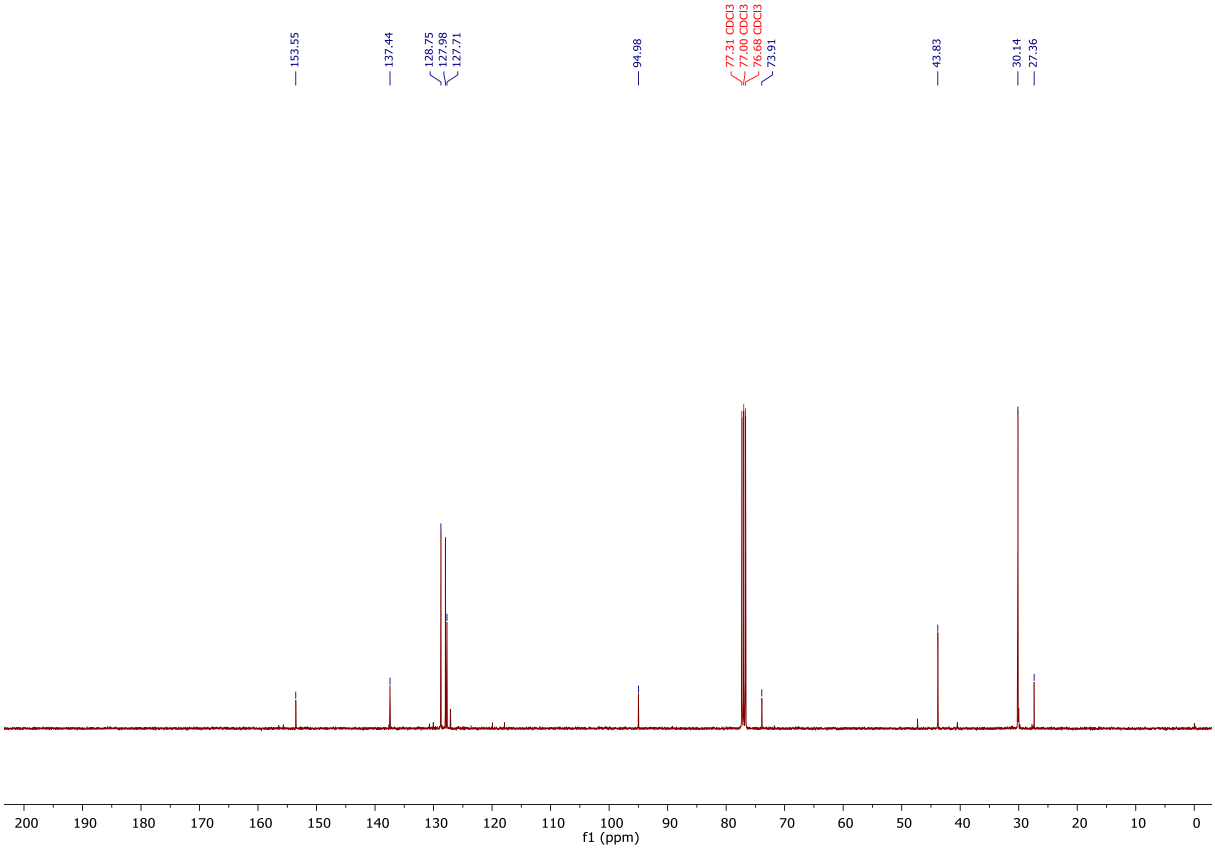

^{13}C NMR of *N*-benzyl-4,4-dimethylpent-2-ynamide **14b**

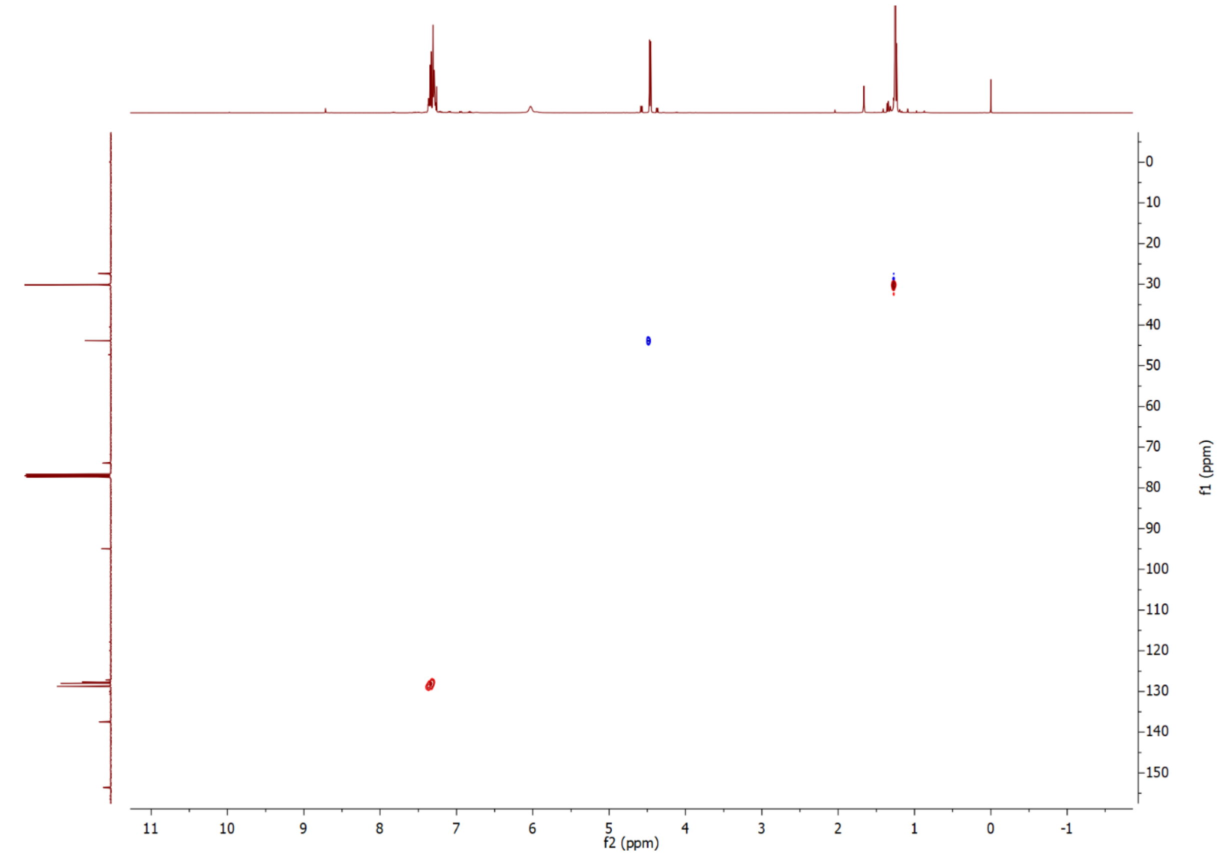

^{1}H-^{13}C HSQC of *N*-benzyl-4,4-dimethylpent-2-ynamide **14b**

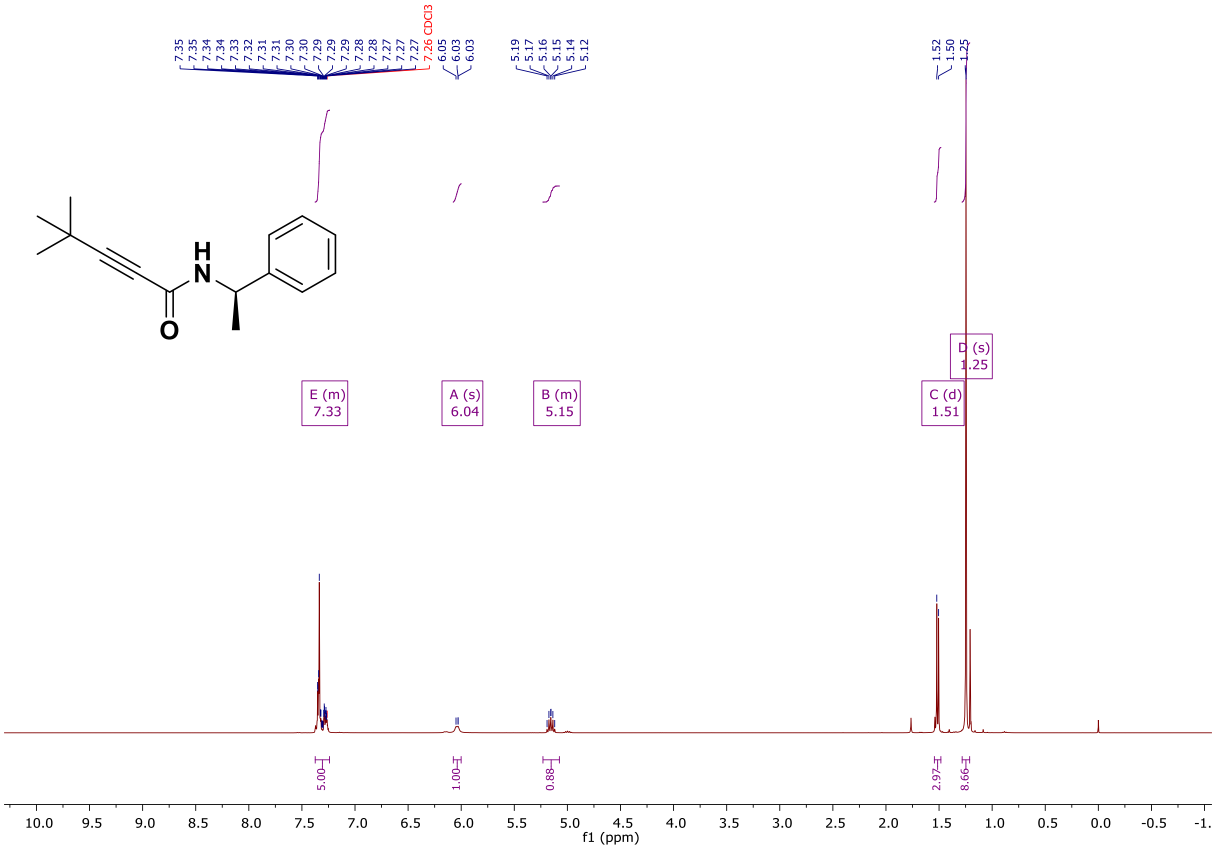

¹H NMR of (*R*)-4,4-dimethyl-N-(1-phenylethyl)pent-2-ynamide **14c**

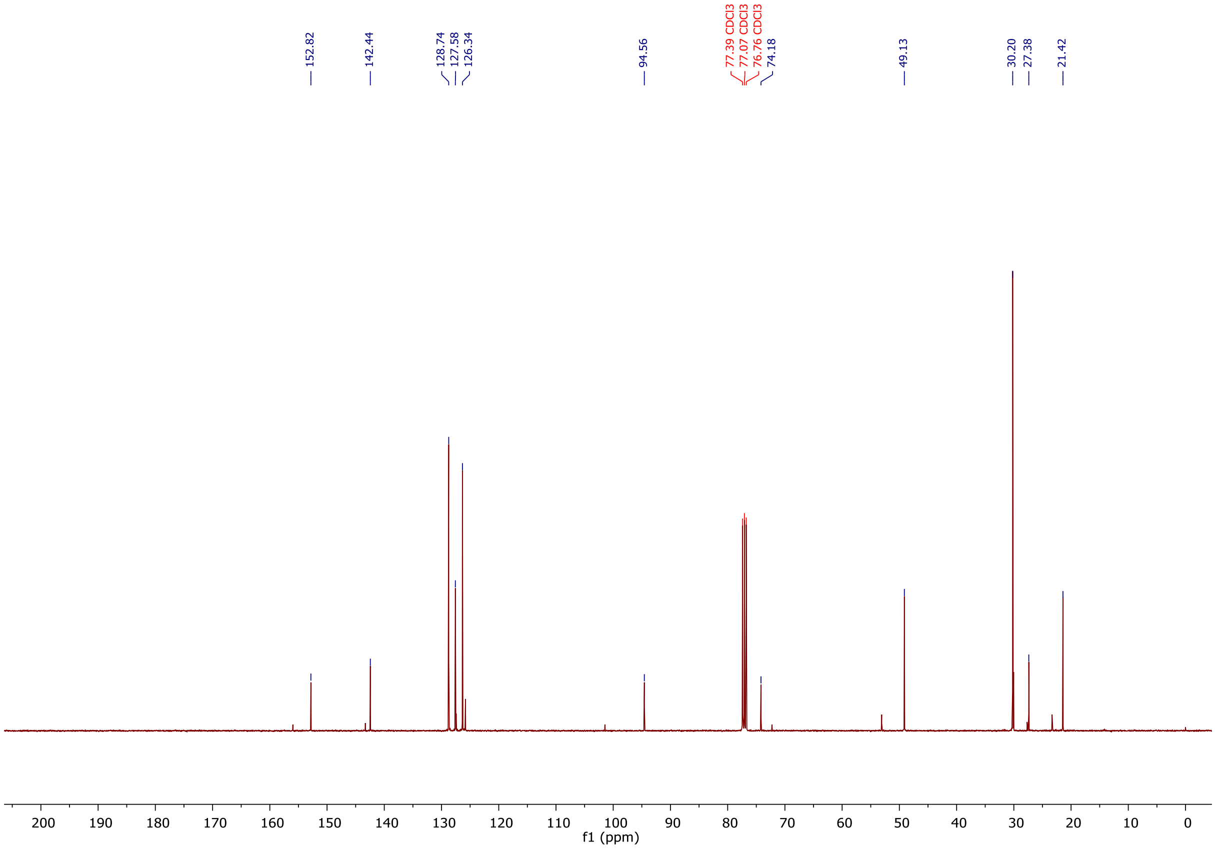

¹³C NMR of (*R*)-4,4-dimethyl-N-(1-phenylethyl)pent-2-ynamide **14c**

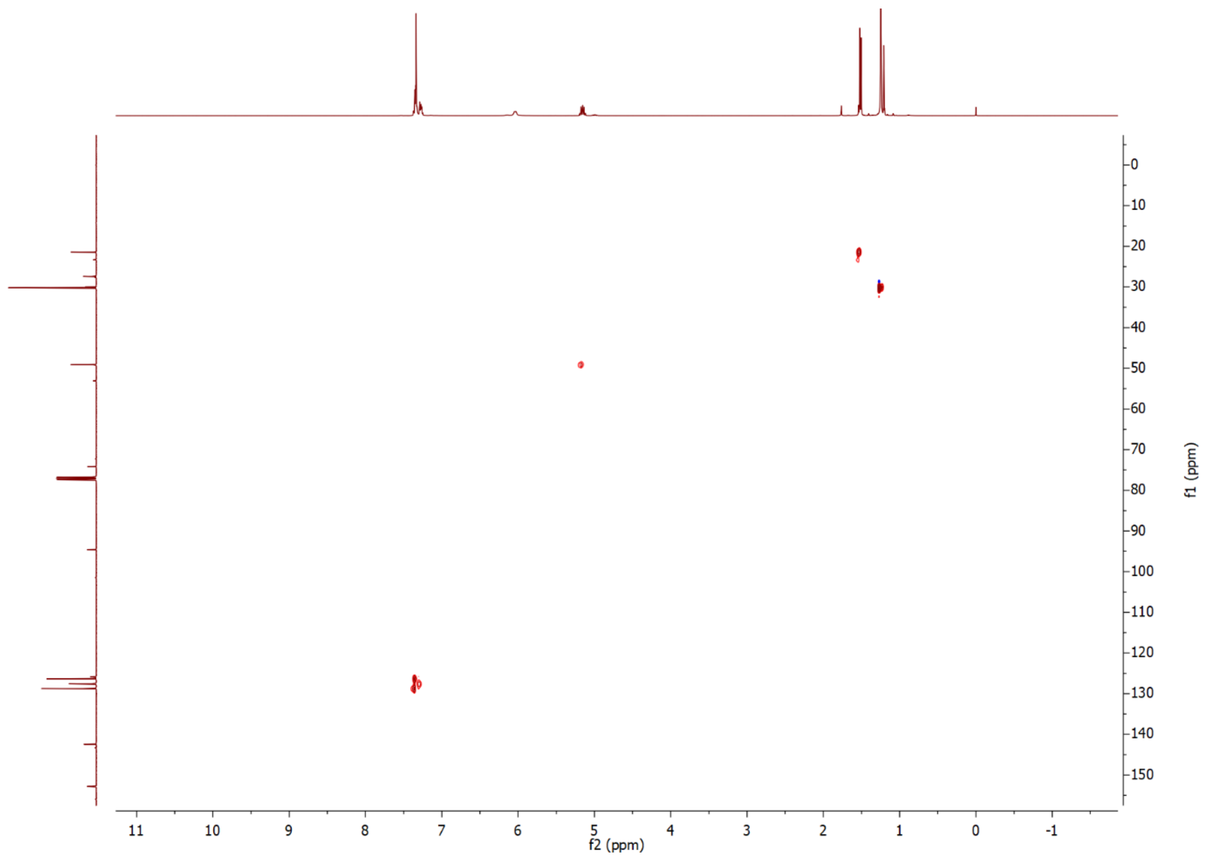

^{1}H-^{13}C HSQC of (*R*)-4,4-dimethyl-N-(1-phenylethyl)pent-2-ynamide **14c**

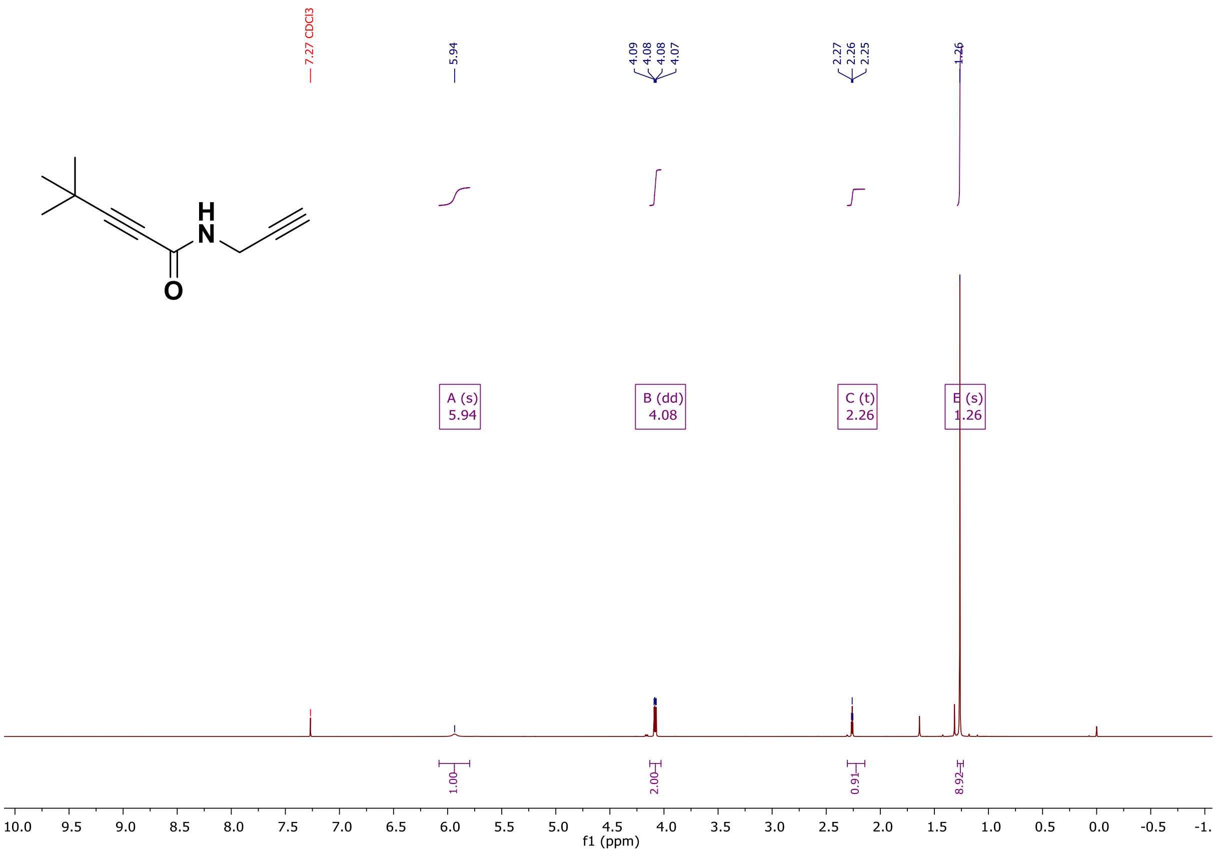

^{1}H NMR of 4,4-dimethyl-*N*-(prop-2-yn-1-yl)pent-2-ynamide **14d**

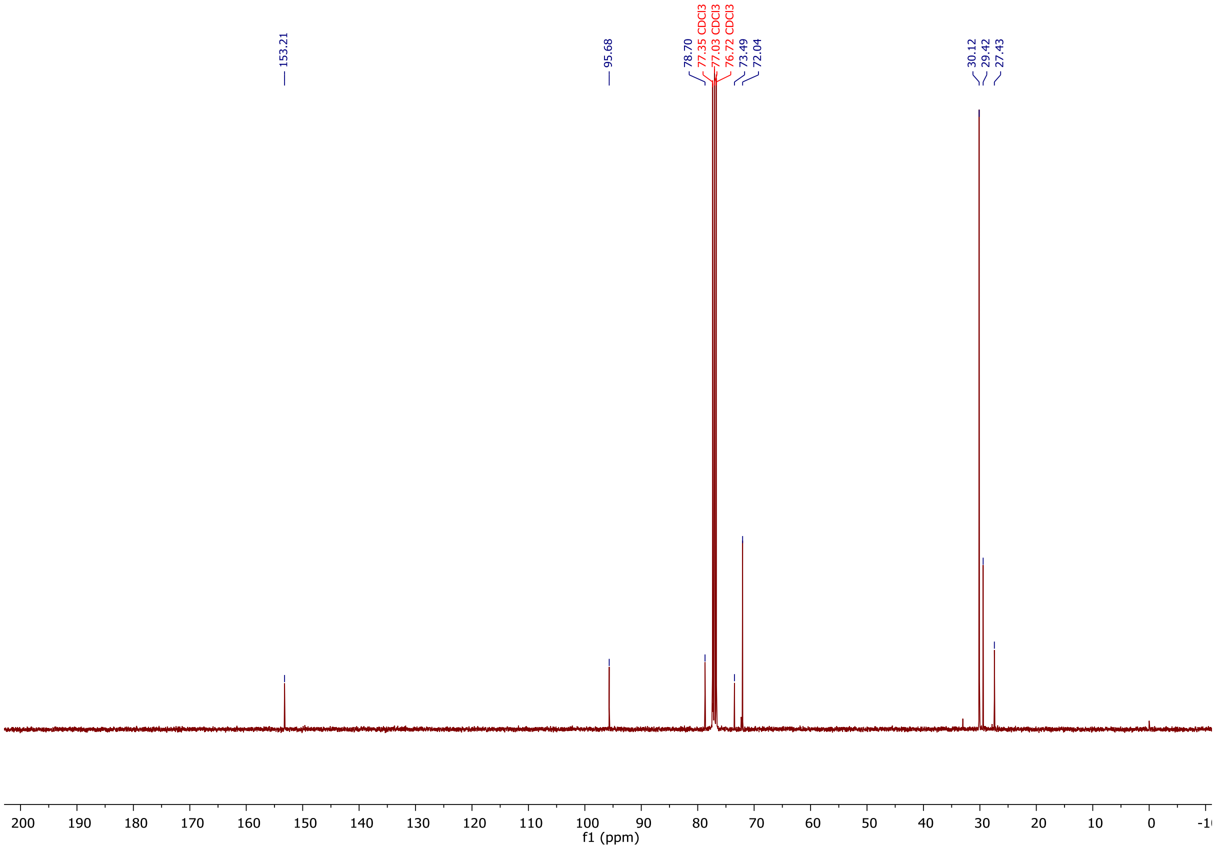

^{13}**C NMR** of 4,4-dimethyl-*N*-(prop-2-yn-1-yl)pent-2-ynamide **14d**

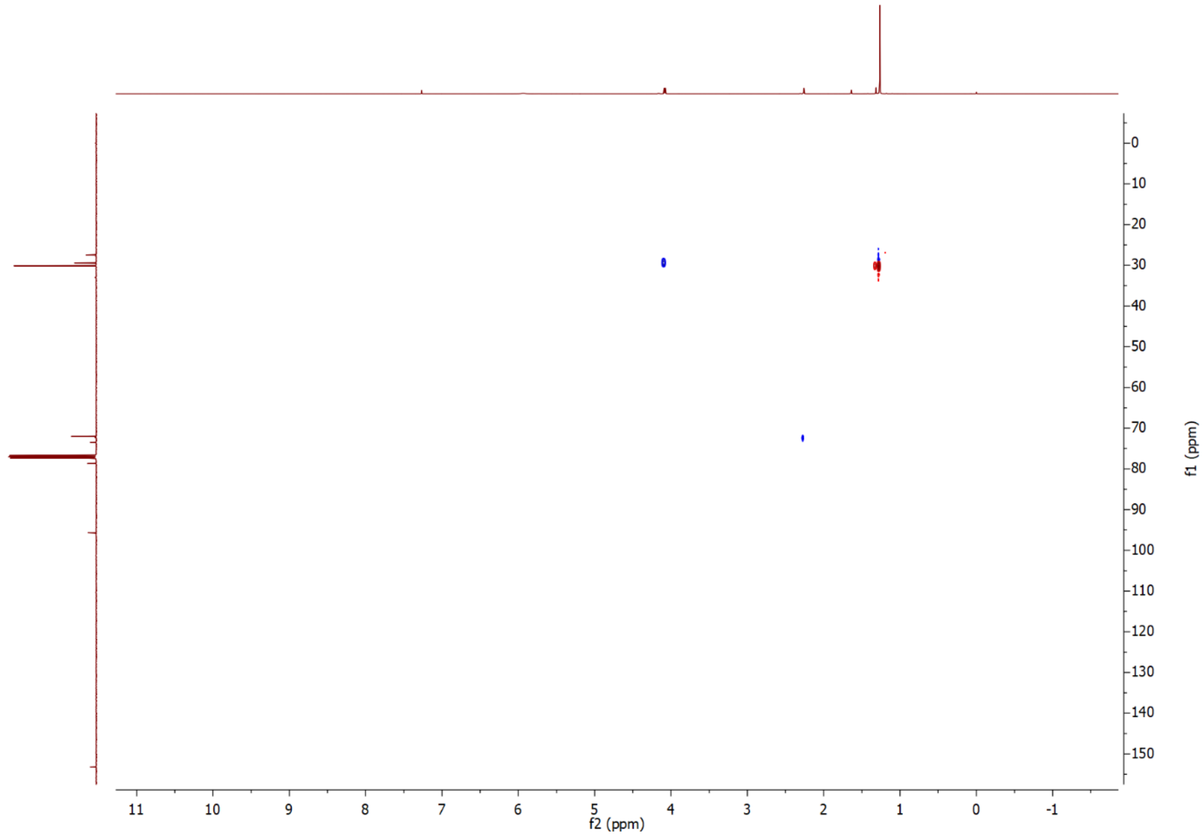

^{1}**H-**^{13}**C HSQC** of 4,4-dimethyl-*N*-(prop-2-yn-1-yl)pent-2-ynamide **14d**

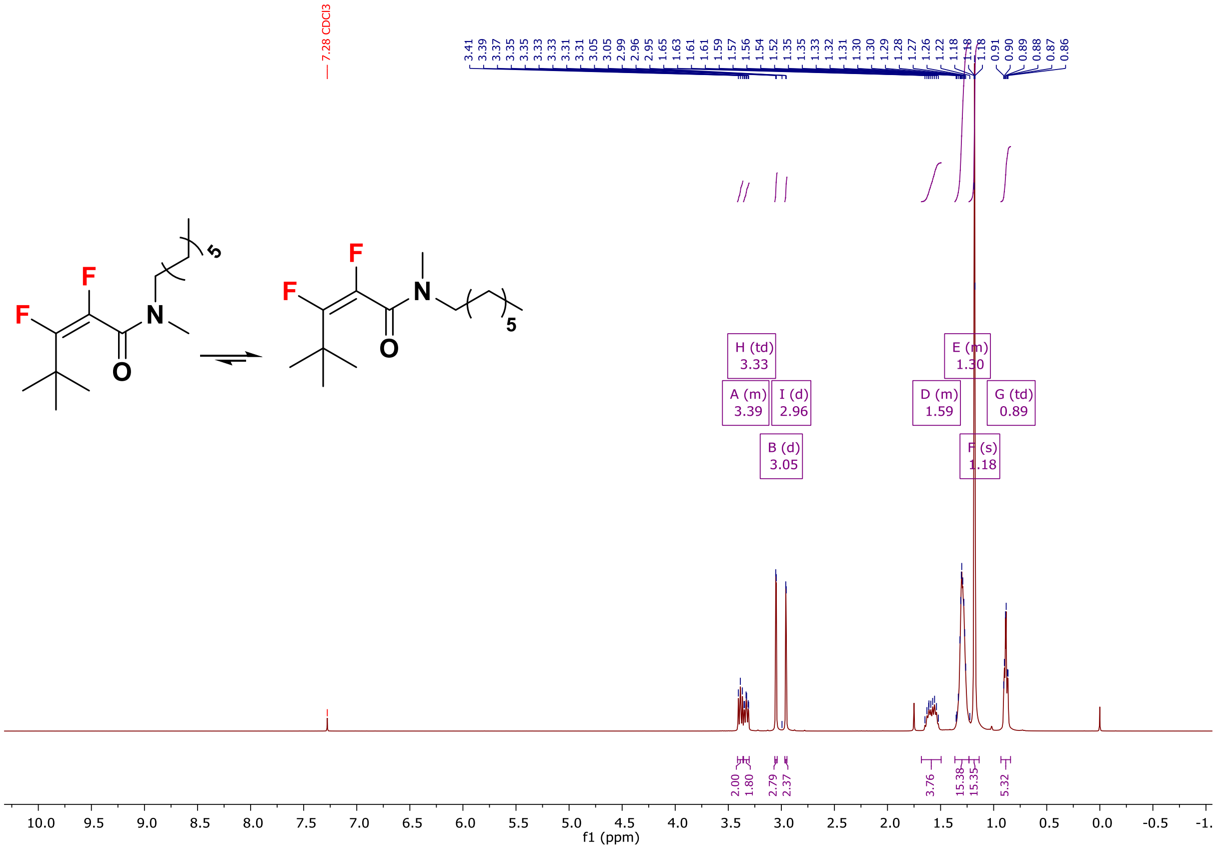

^{1}H NMR of (*Z*)-2,3-difluoro-*N*-heptyl-*N*,4,4-trimethylpent-2-enamide **15a (15a')**

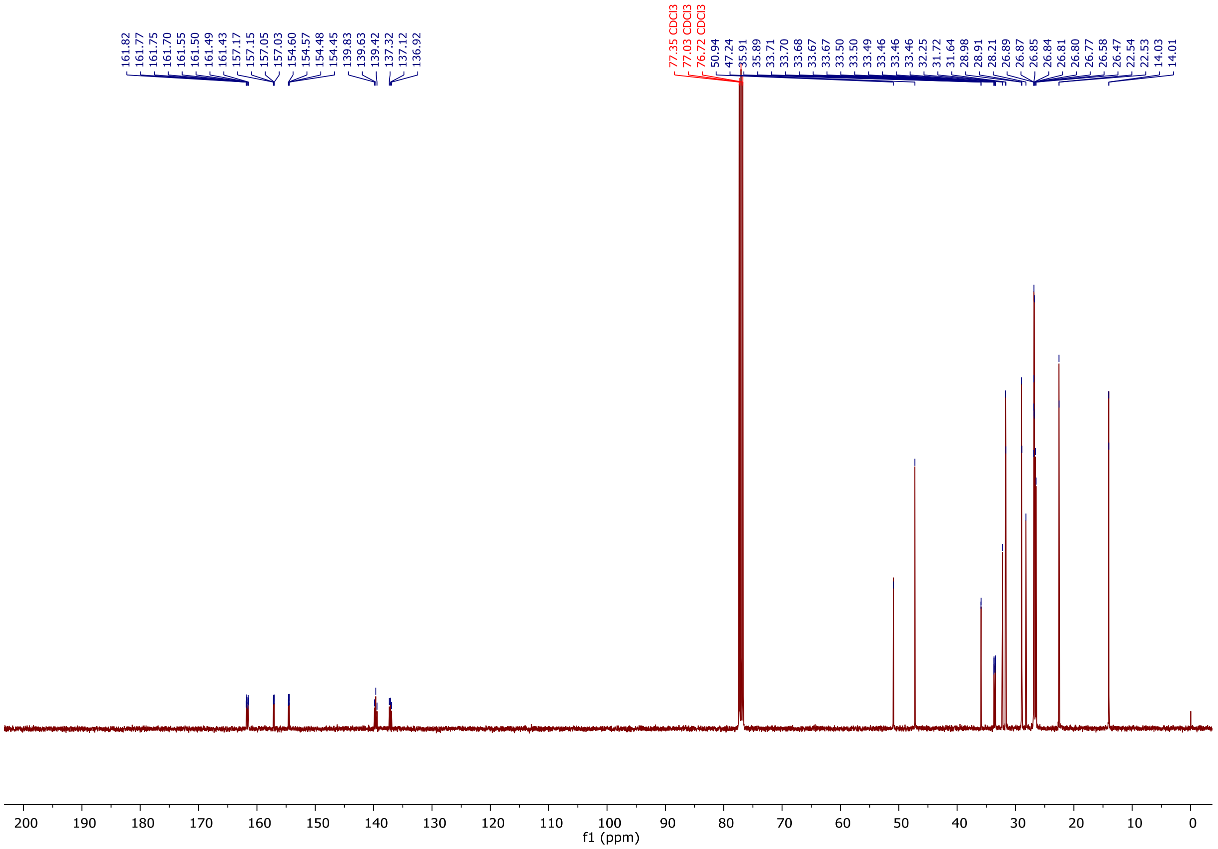

^{13}C NMR of (*Z*)-2,3-difluoro-*N*-heptyl-*N*,4,4-trimethylpent-2-enamide **15a (15a')**

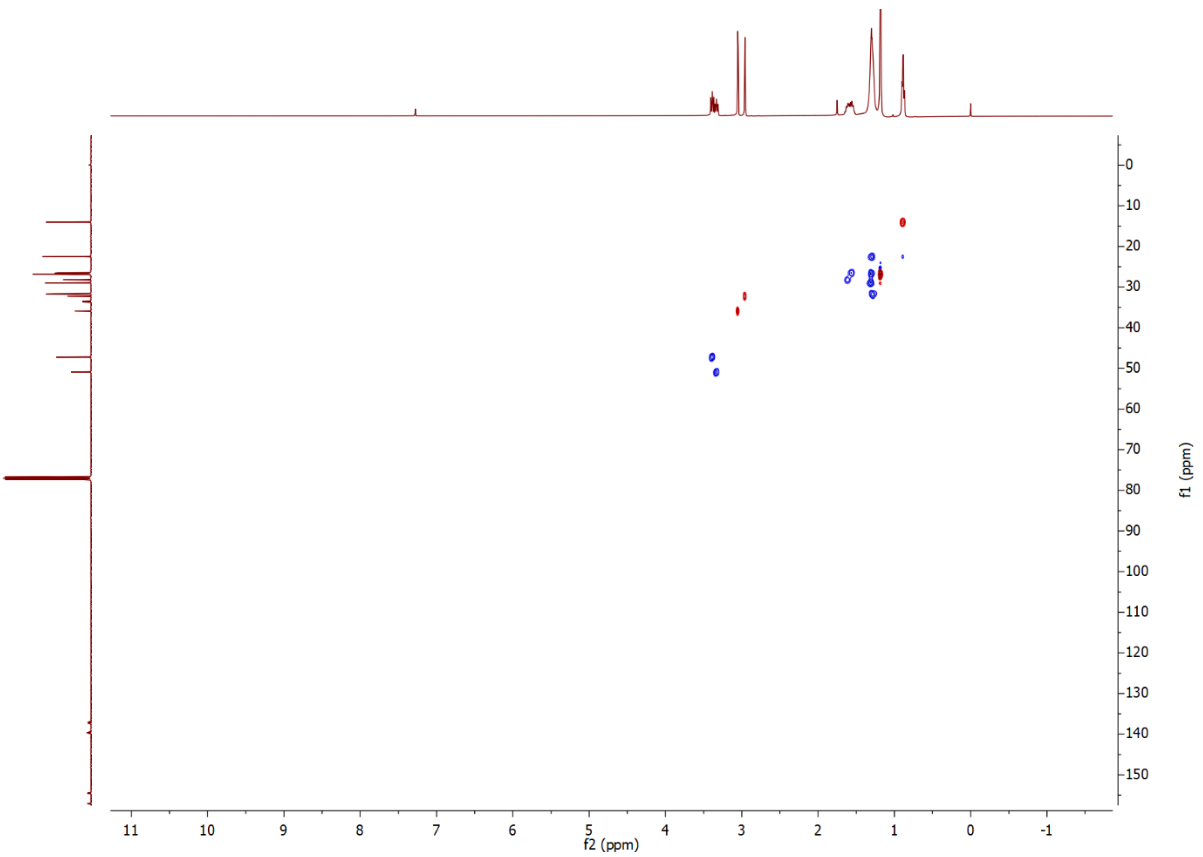

^{1}H-^{13}C HSQC of (*Z*)-2,3-difluoro-*N*-heptyl-*N*,4,4-trimethylpent-2-enamide **15a (15a')**

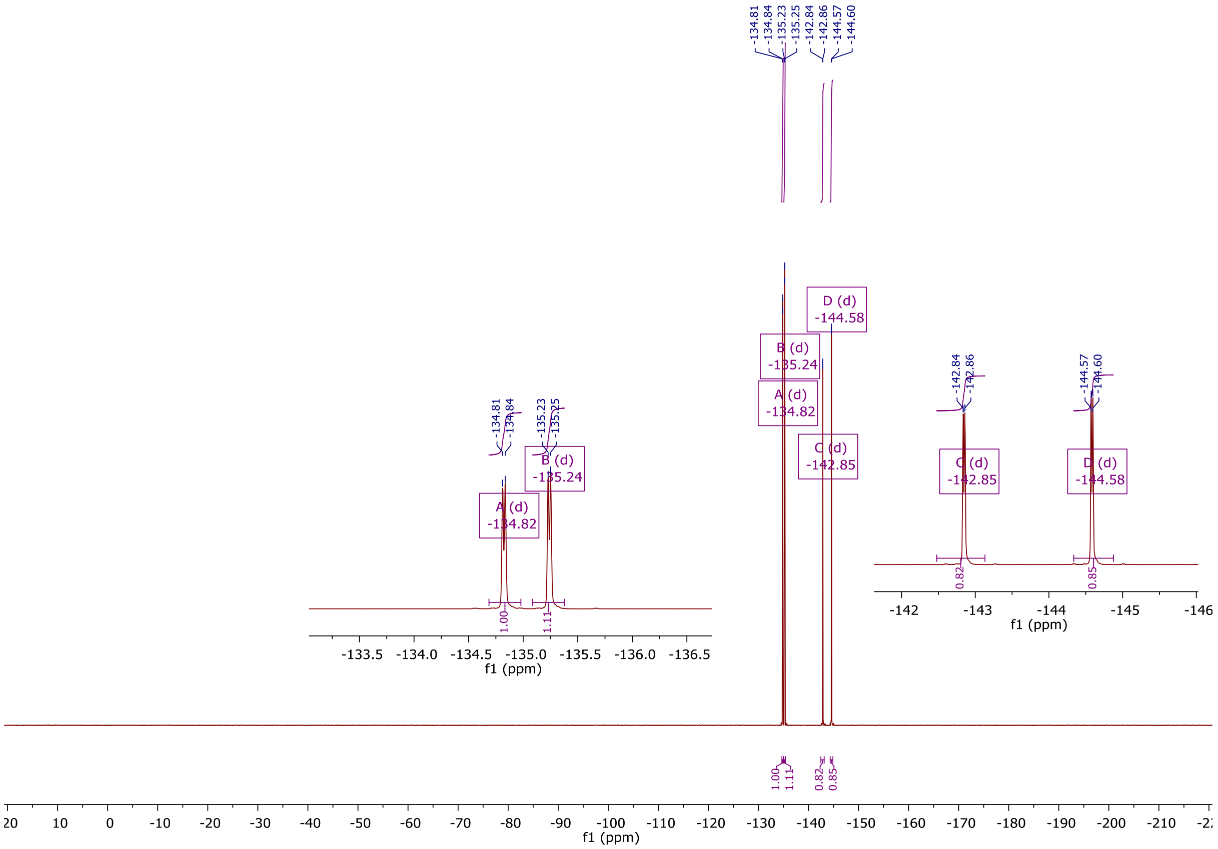

^{19}F NMR of (*Z*)-2,3-difluoro-*N*-heptyl-*N*,4,4-trimethylpent-2-enamide **15a (15a')**

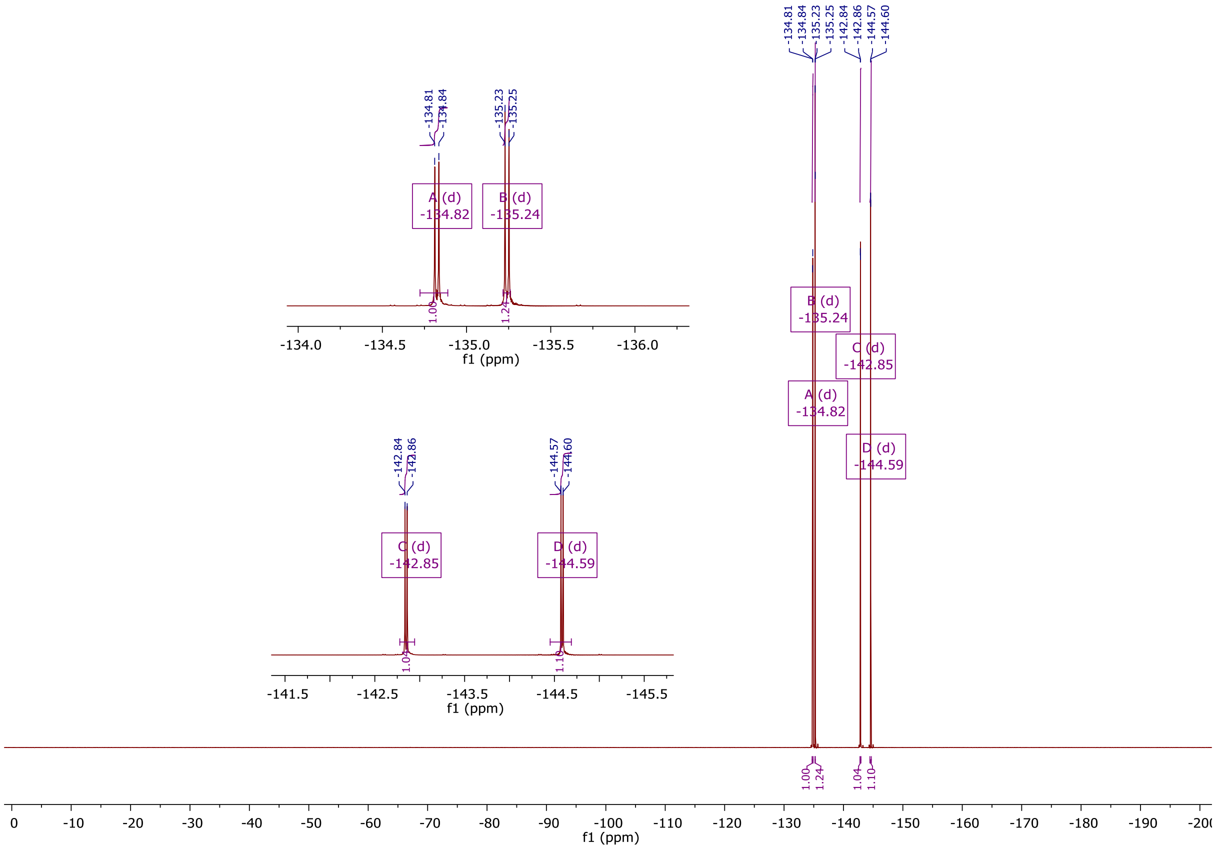

^{19}F{^{1}H} NMR of (*Z*)-2,3-difluoro-*N*-heptyl-*N*,4,4-trimethylpent-2-enamide **15a (15a')**

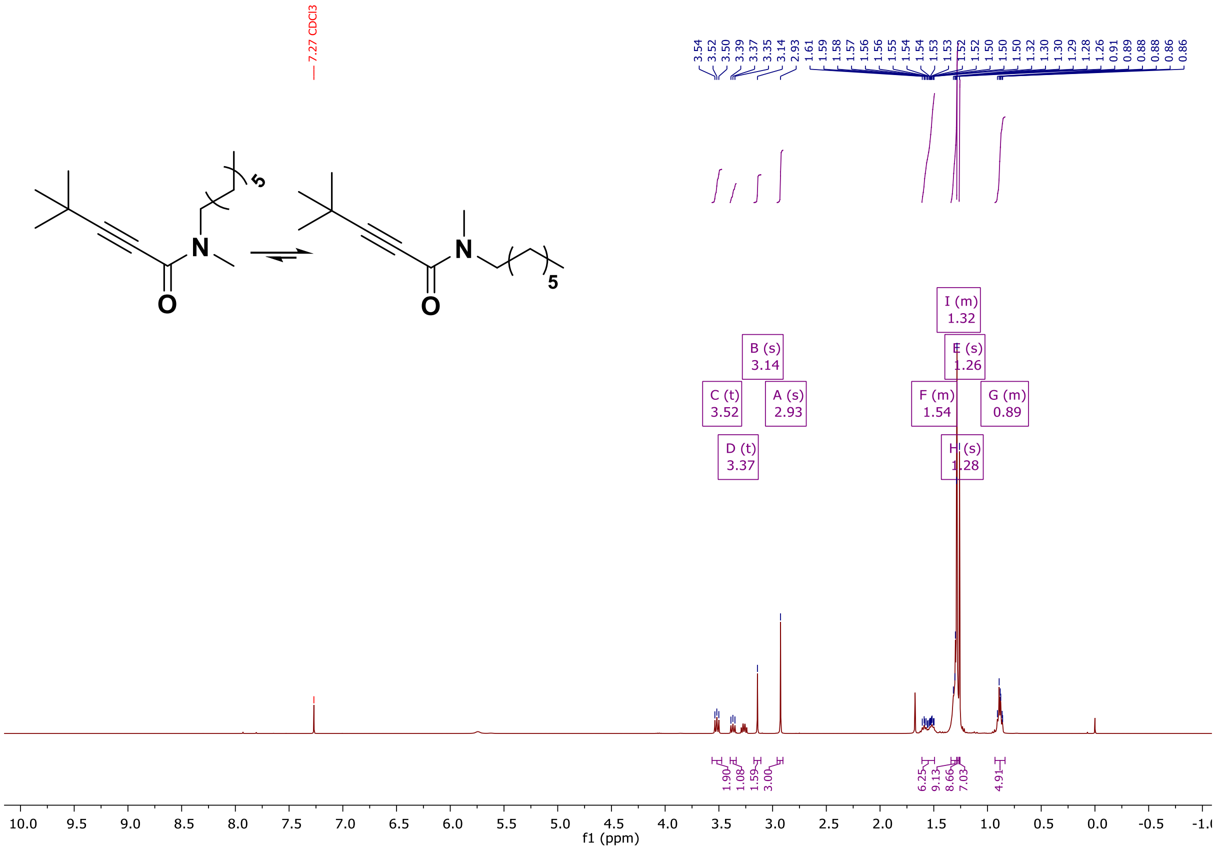

^{1}H NMR of *N*-heptyl-*N*,4,4-trimethylpent-2-ynamide **16a (16a')**

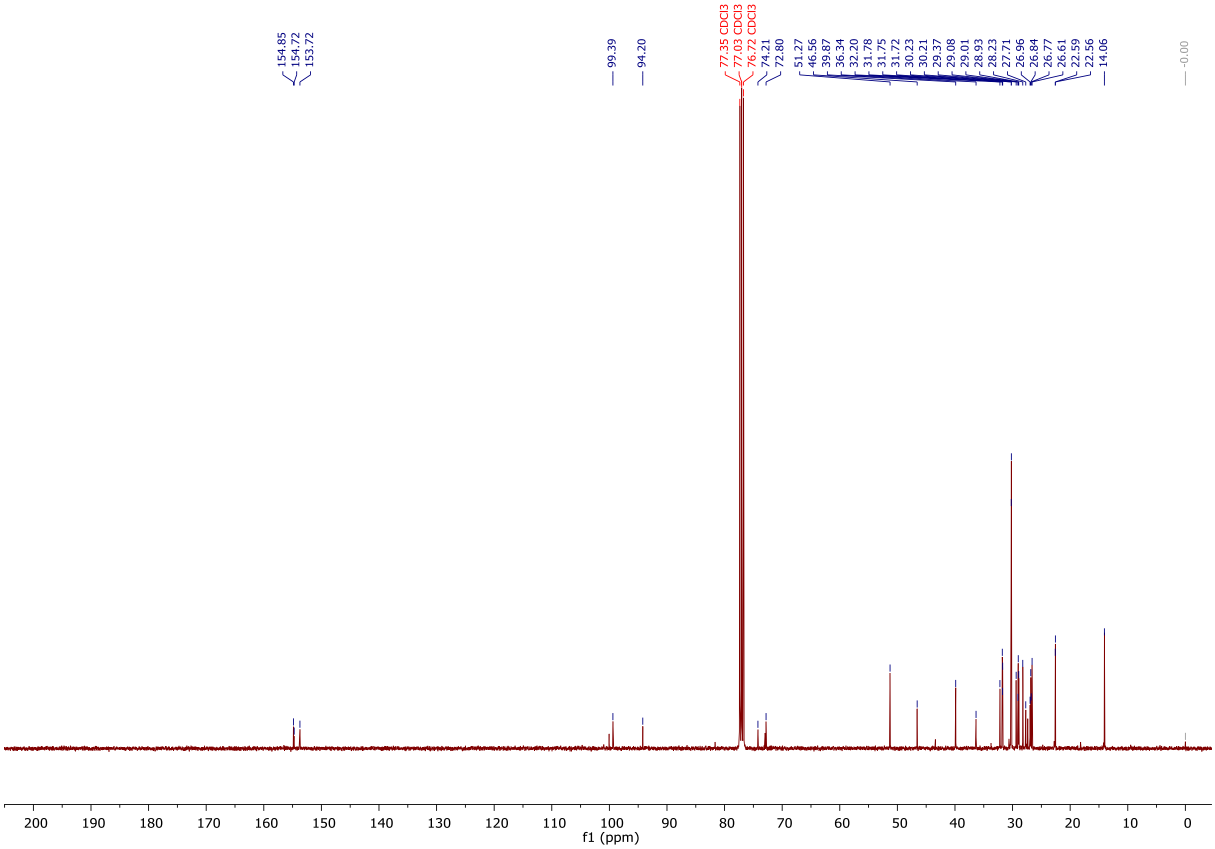

^{13}**C NMR** of *N*-heptyl-*N*,4,4-trimethylpent-2-ynamide **16a (16a')**

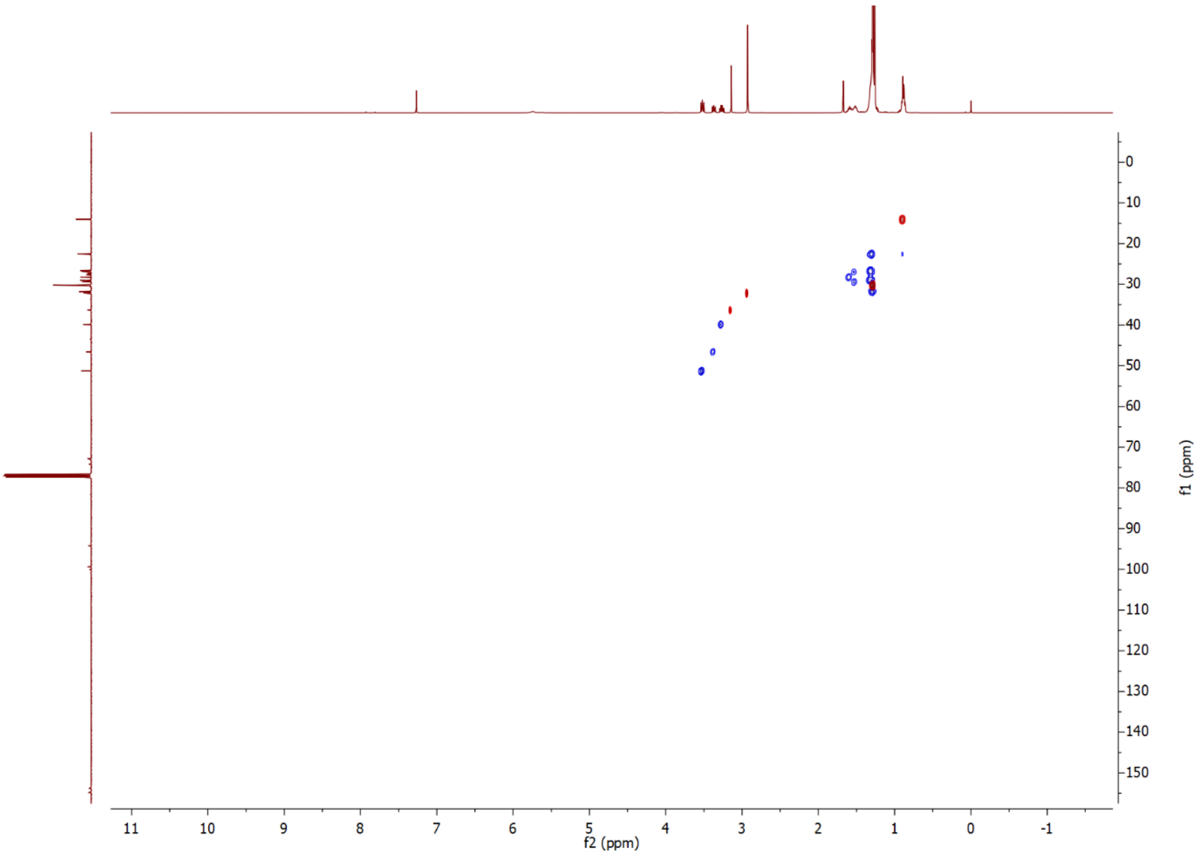

^{1}**H-**^{13}**C HSQC** of *N*-heptyl-*N*,4,4-trimethylpent-2-ynamide **16a (16a')**

3. DFT Calculations

3.1. Methods

Initial structures of substrates and experimentally obtained products were optimized within DFT framework at ωB97XD/6-31+G(d) level of theory[2–8]. To find possible reaction pathways we conducted relaxed potential energy scans while controlling 1 or 2 interatomic distances. QST3[9] (synchronous transit-guided quasi-Newton approach) was used to determine the geometry of transition state. Pseudo IRC[10] calculation were conducted to confirm or generate potential energy minima that are connected by a given TS. For all stationary points identified throughout the research, force constants and the resulting vibrational modes (freq calculations[11]) were computed. All calculations were performed with the GAUSSIAN 16[12].

3.2. Results

3.2.1. Reaction pathway of 2,3,3,3-tetrafluoro-*N*-heptylpropanamide (**1a**) with *n*-BuLi

Performed calculations allowed us to obtain optimized structures of substrates, transition state and products of reaction of 2,3,3,3-tetrafluoro-*N*-heptylpropanamide (**1a**) with *n*-BuLi. Gained energies are gathered in Figure S1. As shown in Figure S1, in order to obtain transition state with lithium connected to oxygen atom of 2,3,3,3-tetrafluoro-*N*-heptylpropanamide (**1a**), a barrier of 0.1 kcal/mol has to overcome. Formation of the *gem*-difluorinated Michael acceptor seems to be reversible as the reverse reaction would require 0.2 kcal/mol.

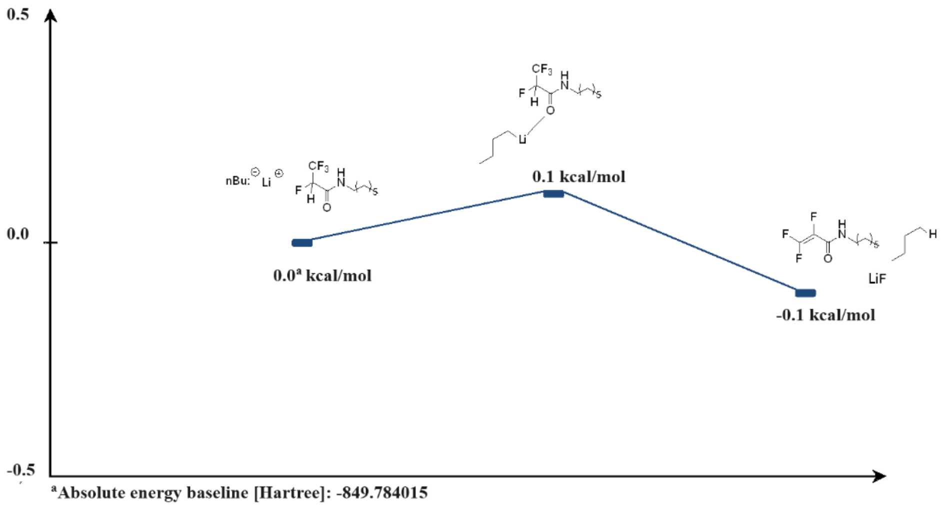

Figure S1. DFT Gibbs free energy calculation of possible reaction pathway of 2,3,3-trifluoro-*N*-heptylacrylamide formation.

Optimized structures of substrates, transition state and products are depicted in Figure S2 (coordinates of those structures are included in Table S1).

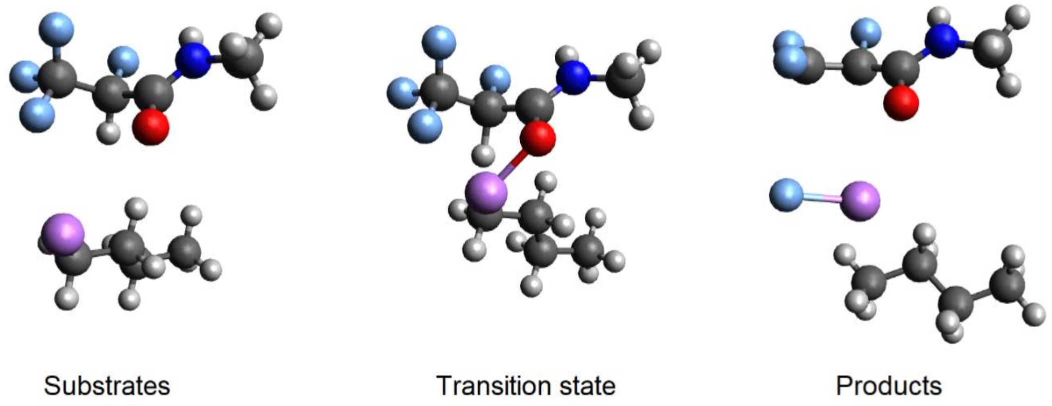

Figure S2. Optimized structures of substrates, transition state and products of 2,3,3,3-tetrafluoro-*N*-heptylpropanamide **1a** reaction with n-BuLi.

In second step, 2,3,3-trifluoro-*N*-heptylacrylamide reacts with *n*-BuLi yielding (*Z*)-2,3-difluoro-*N*-heptylhept-2-enamide (**9a**). Energies for this reaction are gathered in Figure S3. As shown in Figure S3, the formation of transition state requires overcoming an energy barrier of 17.9 kcal/mol. Creating final product seems to be irreversible because reverse reaction would require 93.6 kcal/mol.

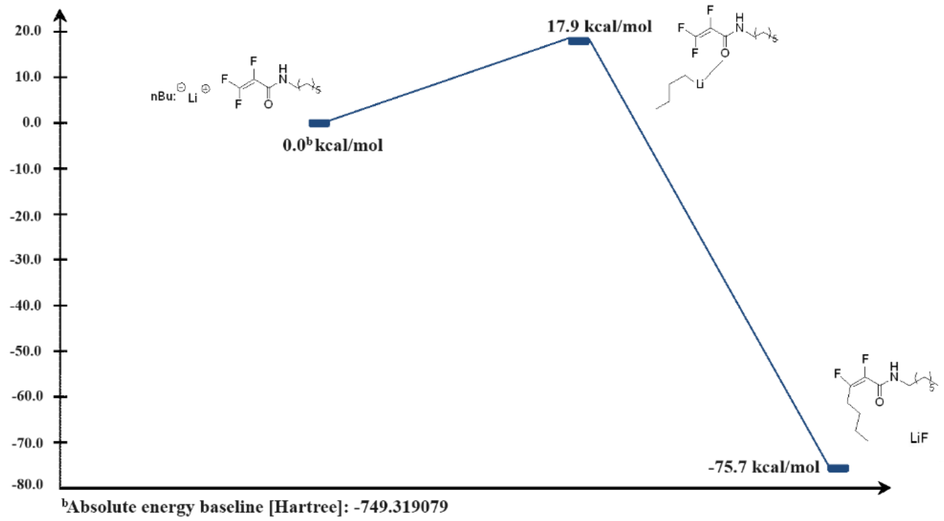

Figure S3. DFT Gibbs free energy calculation of possible reaction pathway of (*Z*)-2,3-difluoro-*N*-heptylhept-2-enamide (**9a**) formation.

Optimized structures of substrates, transition state and products are presented in Figure S4 (coordinates of those structures are included in Table S2).

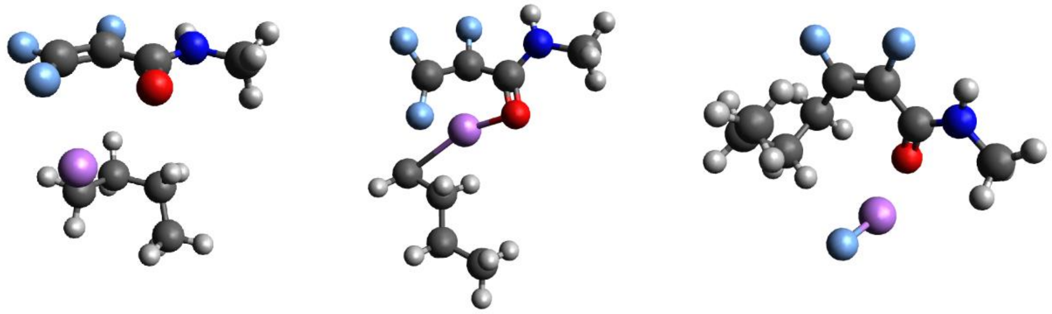

Figure S4. Optimized structures of substrates, transition state and products of 2,3,3-trifluoro-*N*-heptylacrylamide reaction with *n*-BuLi.

In third step, (*Z*)-2,3-difluoro-*N*-heptylhept-2-enamide **(**9a**)** reacts with *n*-BuLi forming 3-butyl-2-fluoro-*N*-heptylhept-2-enamide (**10a**). Energies for this reaction are gathered in Figure S5. As shown in Figure S5, the formation of transition state requires overcoming an energy barrier of 7.4 kcal/mol. Creating final product seems to be irreversible because reverse reaction would require 90.7 kcal/mol.

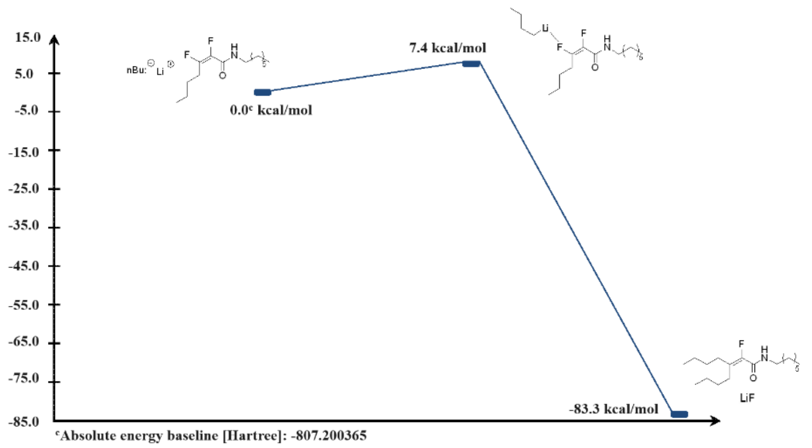

Figure S5. DFT Gibbs free energy calculation of possible reaction pathway of 3-butyl-2-fluoro-*N*-heptylhept-2-enamide (**10a**) formation.

Optimized structures of substrates, transition state and products are depicted in Figure S6 (coordinates of those structures are included in Table 3).

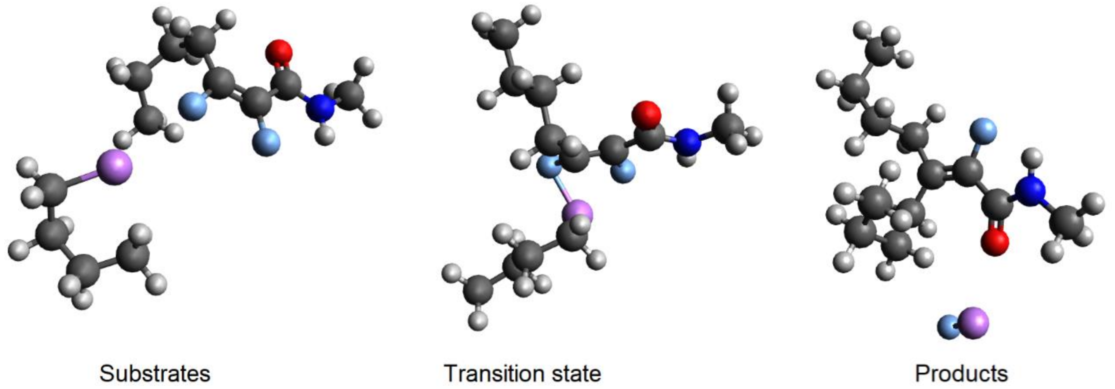

Figure S6. Optimized structures of substrates, transition state and products of (*Z*)-2,3-difluoro-*N*-heptylhept-2-enamide (**9a**) reaction with n-BuLi.

3.2.2. Reaction pathway of 3,3,3-trifluoro-*N*-heptylpropanamide (**2a**) with n-BuLi

Performed calculations allowed us to obtain optimized structures of substrates, transition state and products. Obtained energies are gathered in Figure S7. As shown in Figure S7, in order to obtain transition state with lithium connected to oxygen atom of 3,3,3-trifluoro-*N*-heptylpropanamide (**2a**) a barrier of 0.1 kcal/mol has to overcome. The formation of *gem*-difluorinated Michael acceptor seems to be reversible as the reverse reaction would require 0.2 kcal/mol.

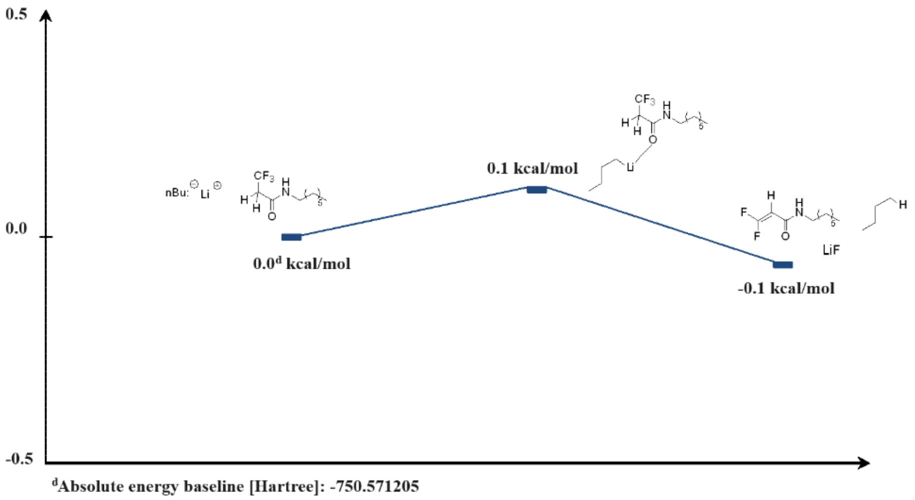

Figure S7. DFT Gibbs free energy calculation of possible reaction pathway of 3,3-difluoro-*N*-heptylacrylamide formation.

Optimized structures of substrates, transition state and products are depicted in Figure S8 (coordinates of those structures are included in Table 4).

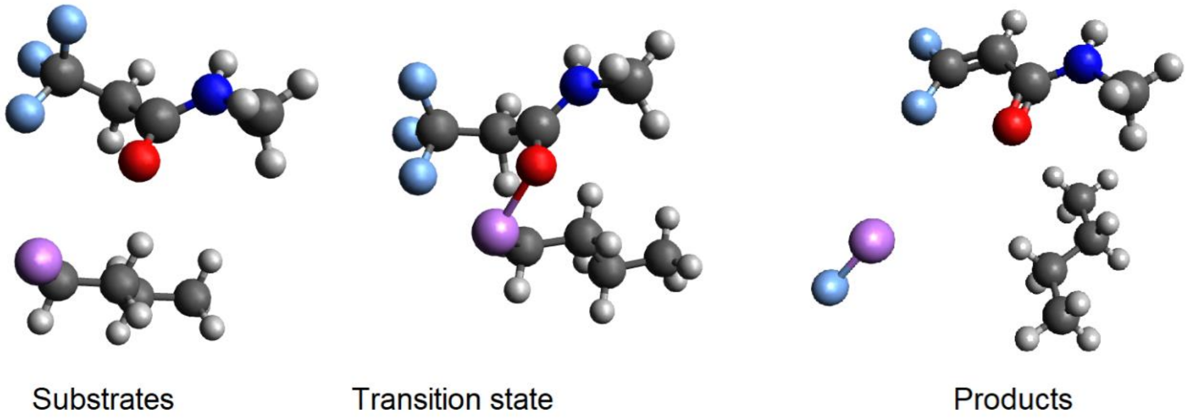

Figure S8. Optimized structures of substrates, transition state and products of 3,3,3-trifluoro-*N*-heptylpropanamide (**2a**) reaction with n-BuLi.

In second step, 3,3-difluoro-*N*-heptylacrylamide reacts with *n*-BuLi yielding (*E*)-3-fluoro-*N*-heptylhept-2-enamide (**11a**). Energies for this reaction are presented in Figure S9. As shown in Figure S9 the formation of transition state requires overcoming an energy barrier of 22.9 kcal/mol. Creating final product seems to be irreversible because reverse reaction would require 99.4 kcal/mol.

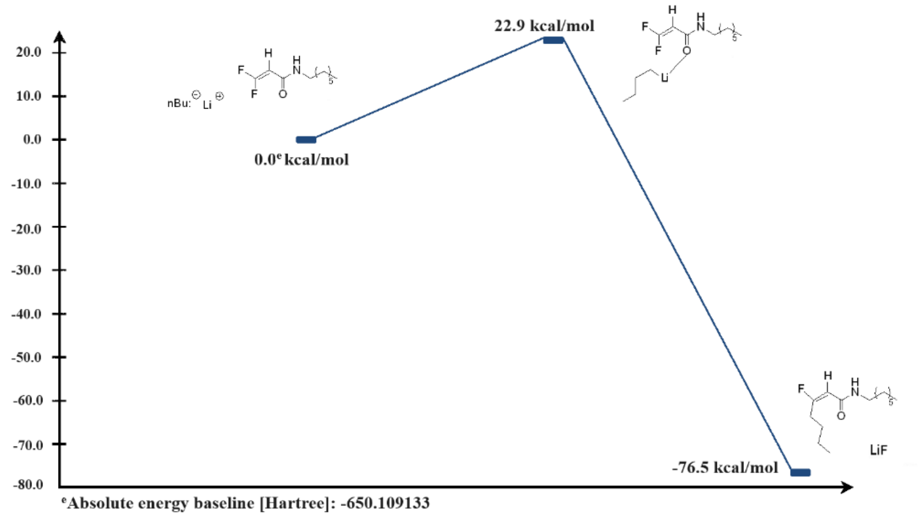

Figure S9. DFT Gibbs free energy calculation of possible reaction pathway of (*E*)-3-fluoro-*N*-heptylhept-2-enamide (**11a**) formation.

Optimized structures of substrates, transition state and products are depicted in Figure S10 (coordinates of those structures are included in Table 5).

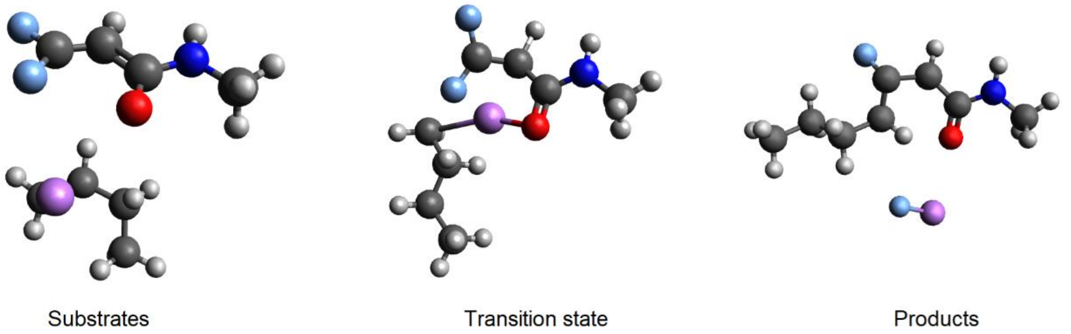

Figure S10. Optimized structures of substrates, transition state and products of reaction of 3,3-difluoro-*N*-heptylacrylamide with n-BuLi.

In third step, (*E*)-3-fluoro-*N*-heptylhept-2-enamide (**11a**) reacts with n-BuLi yielding *N*-heptylhept-2-ynamide (**12a**). Energies for this reaction are gathered in Figure S11. As shown in Figure S11 the formation of transition state requires overcoming an energy barrier of 37.0 kcal/mol. Creating final product seems to be irreversible because reverse reaction would require 73.5 kcal/mol.

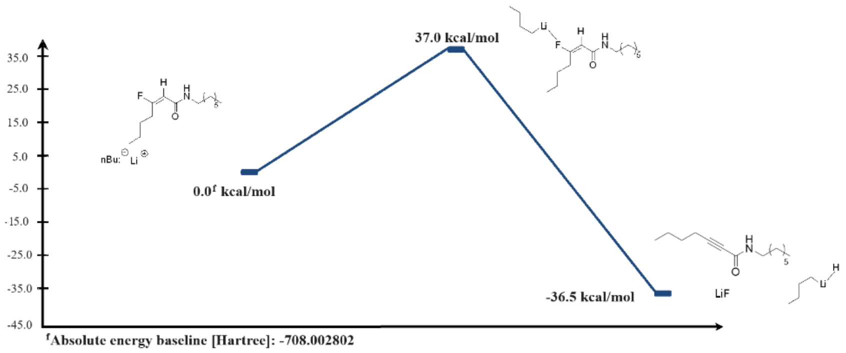

Figure S11. DFT Gibbs free energy calculation of possible reaction pathway of *N*-heptylhept-2-ynamide (**12a**) formation.

Optimized structures of substrates, transition state and products are depicted in Figure S12 (coordinates of those structures are included in Table 6).

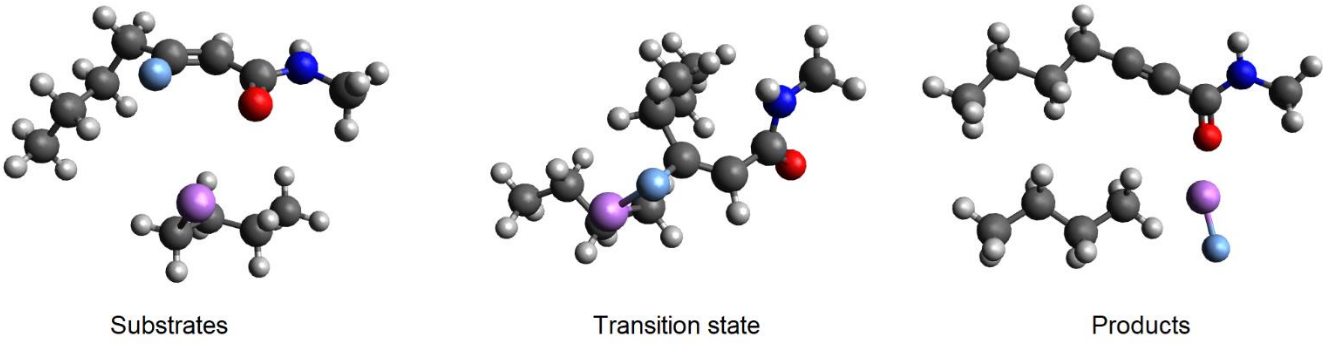

Figure S12. Optimized structures of substrates, transition state and products of reaction of (*E*)-3-fluoro-*N*-heptylhept-2-enamide (**11a**) with n-BuLi.

3.2.3. Reaction pathway of 2,3,3,3-tetrafluoro-*N*-heptylpropanamide **1a** with *tert*-BuLi

Performed calculations allowed us to obtain optimized structures of substrates, transition state and products. Energies are gathered in Figure S13. As shown in Figure S13 in order to obtain transition state with lithium connected to oxygen atom of 2,3,3,3-tetrafluoro-*N*-heptylpropanamide (**1a**) a barrier of 0.4 kcal/mol has to overcome. The formation of final product seems to be irreversible as the reverse reaction would require 34.3 kcal/mol.

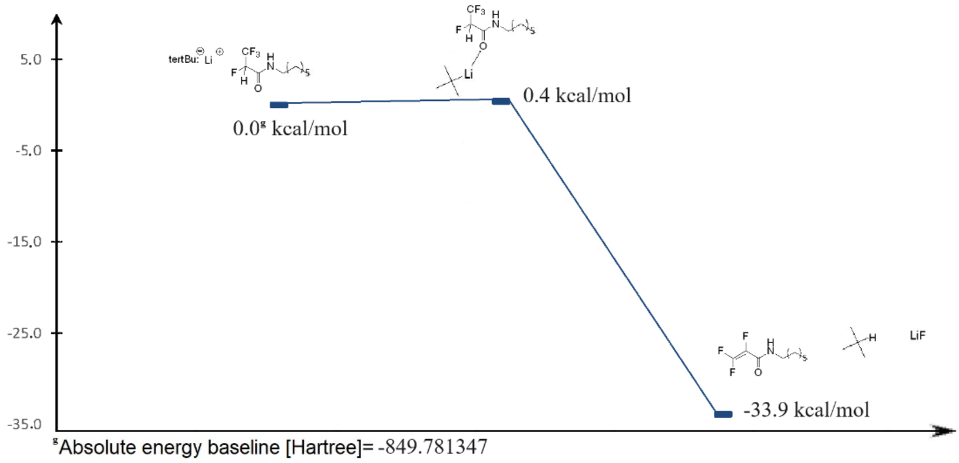

Figure S13. DFT Gibbs free energy calculation of possible reaction pathway of 2,3,3-trifluoro-*N*-heptylacrylamide formation.

Optimized structures of substrates, transition state and products are depicted in Figure S14 (coordinates of those structures are included in Table 7).

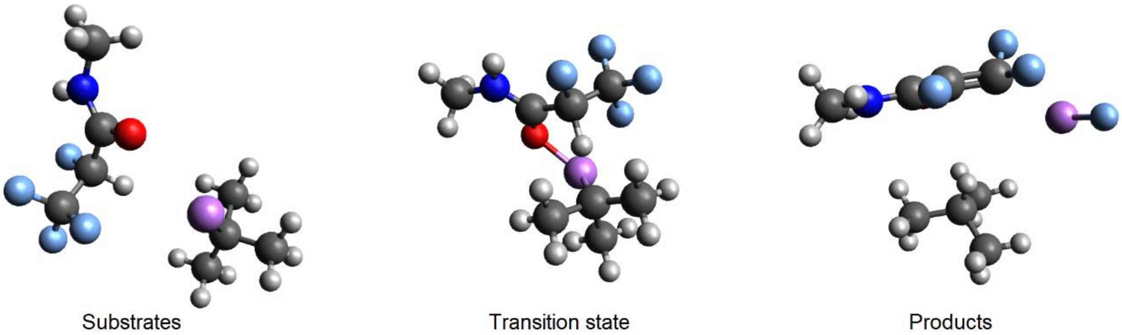

Figure S14. Optimized structures of substrates, transition state and products of 2,3,3,3-tetrafluoro-*N*-heptylpropanamide (**1a**) reaction with *tert*-BuLi.

In second step, the *gem*-difluorinated Michael acceptor reacts with *tert*-BuLi yielding (*Z*)-2,3-difluoro-*N*-heptyl-4,4-dimethylpent-2-enamide (**13a**). Obtained energies for this reaction are gathered in Figure S15. As shown in Figure S15 obtaining transition state requires overcoming an energy barrier of 2.3 kcal/mol. Creating final product seems to be irreversible because reverse reaction would require 62.3 kcal/mol.

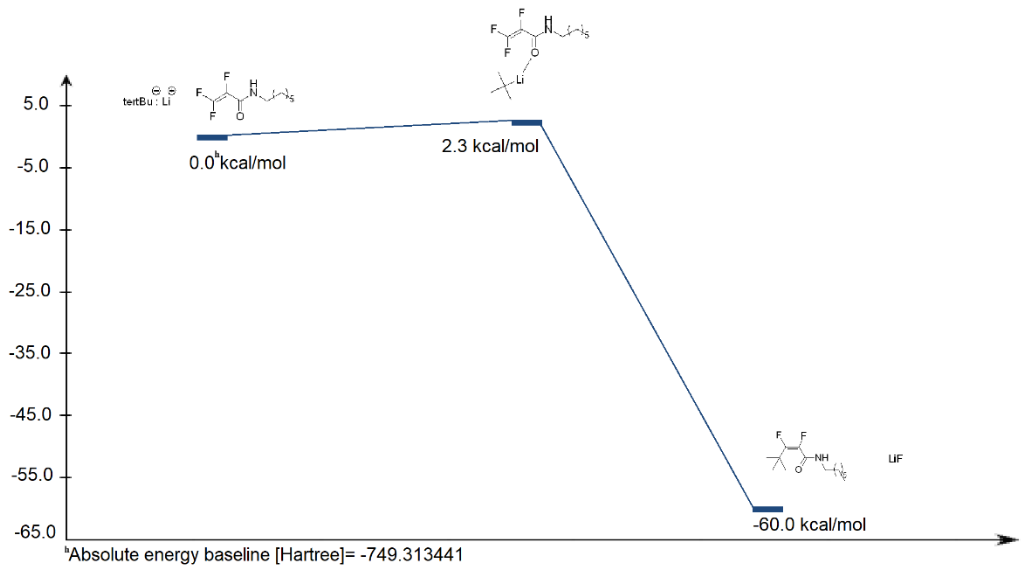

Figure S15. DFT Gibbs free energy calculation of possible reaction pathway of (*Z*)-2,3-difluoro-*N*-heptyl-4,4-dimethylpent-2-enamide (**13a**) formation.

Optimized structures of substrates, transition state and products are depicted in Figure S16 (coordinates of those structures are included in Table 8).

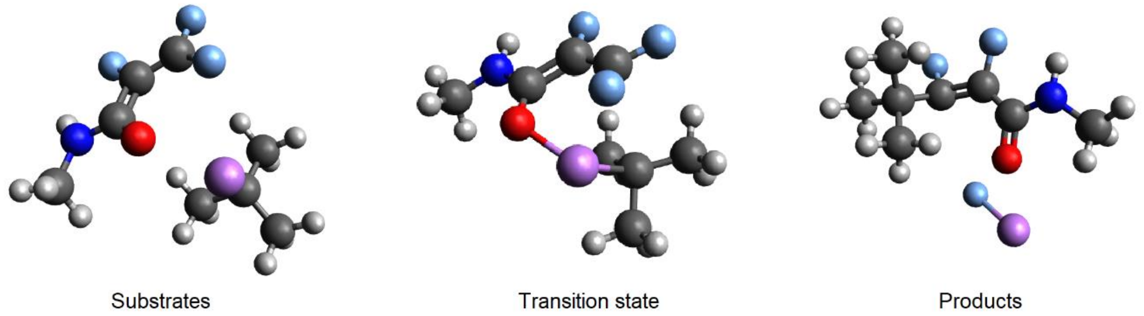

Figure S16. Optimized structures of substrates, transition state and products of 2,3,3-trifluoro-*N*-heptylacrylamide reaction with *tert*-BuLi.

3.2.4. Reaction pathway of 3,3,3-trifluoro-*N*-heptylpropanamide (**2a**) with *tert*-BuLi

Performed calculations allowed us to obtain optimized structures of reaction substrates, transition state and products. Obtained energies are gathered in Figure S17. As shown in Figure S17 in order to obtain transition state with lithium connected to oxygen atom of 3,3,3-trifluoro-*N*-heptylpropanamide (**2a**) a barrier of 0.1 kcal/mol has to overcome. The creation of final product may be reversible as the reverse reaction would require 0.2 kcal/mol.

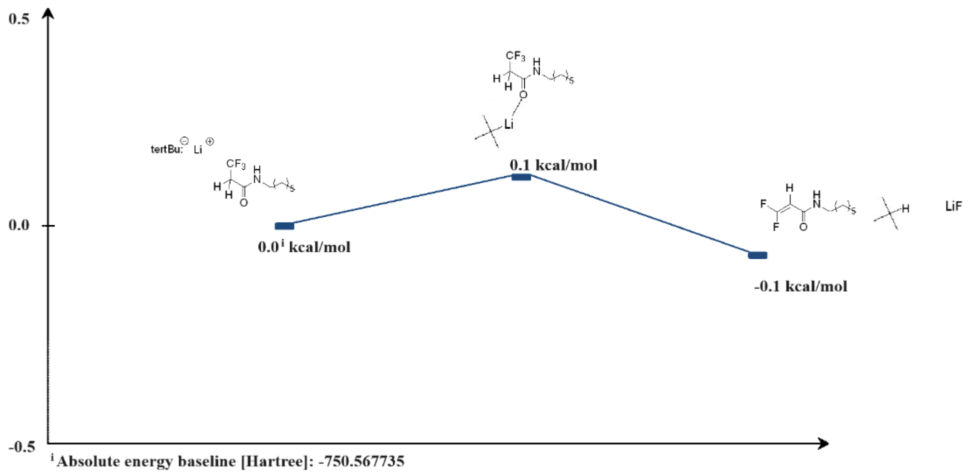

Figure S17. DFT Gibbs free energy calculation of possible reaction pathway of 3,3-difluoro-*N*-heptylacrylamide formation.

Obtained optimized structures of substrates, transition state and products are depicted in Figure S18 (coordinates of those structures are included in Table 9).

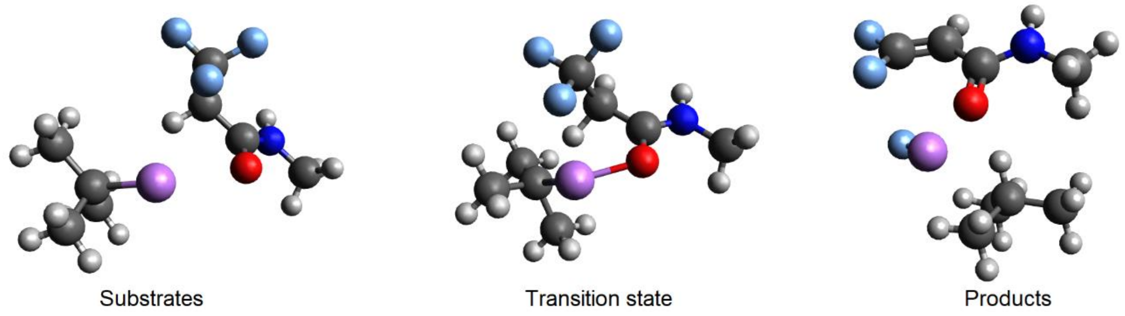

Figure S18. Optimized structures of substrates, transition state and products of 3,3,3-trifluoro-*N*-heptylpropanamide (**2a**) reaction with *tert*-BuLi.

According to the DFT calculation in second step, 3,3-difluoro-*N*-heptylacrylamide reacts with *tert*-BuLi creating (*E*)-3-fluoro-*N*-heptyl-4,4-dimethylpent-2-enamide. Energies for this reaction are gathered in Figure S19. As shown in Figure S19 the creation of transition state requires overcoming an energy barrier of 2.5 kcal/mol. Forming final product seems to be irreversible because reverse reaction would require 79.1 kcal/mol. However, in the performed experiment (*E*)-3-fluoro-*N*-heptyl-4,4-dimethylpent-2-enamide was not isolated. The reaction continued with the

formation of the elimination product *N*-heptyl-4,4-dimethylpent-2-ynamide (**14a**, Figure S21).

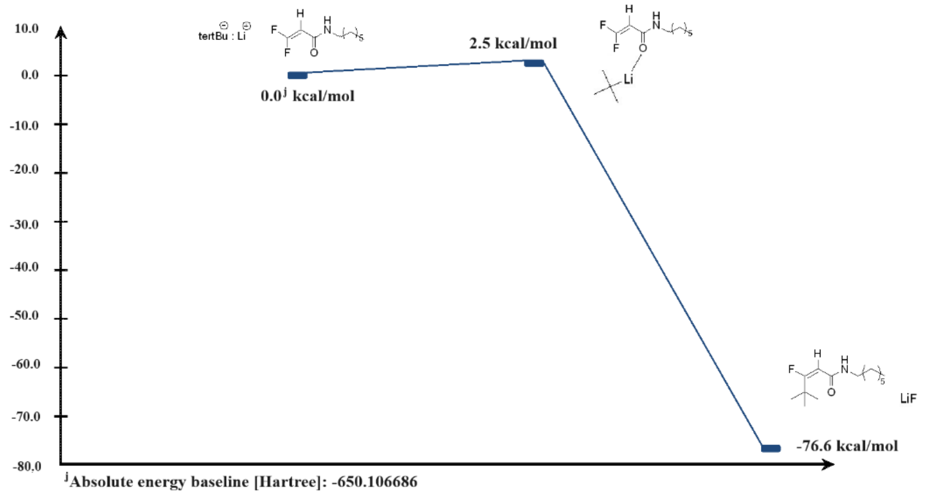

Figure S19. DFT Gibbs free energy calculation of possible reaction pathway of (*E*)-3-fluoro-*N*-heptyl-4,4-dimethylpent-2-enamide formation.

Optimized structures of substrates, transition state and products of 3,3-difluoro-*N*-heptylacrylamide reaction with *tert*-BuLi are depicted in Figure S20 (coordinates of those structures are included in Table 10).

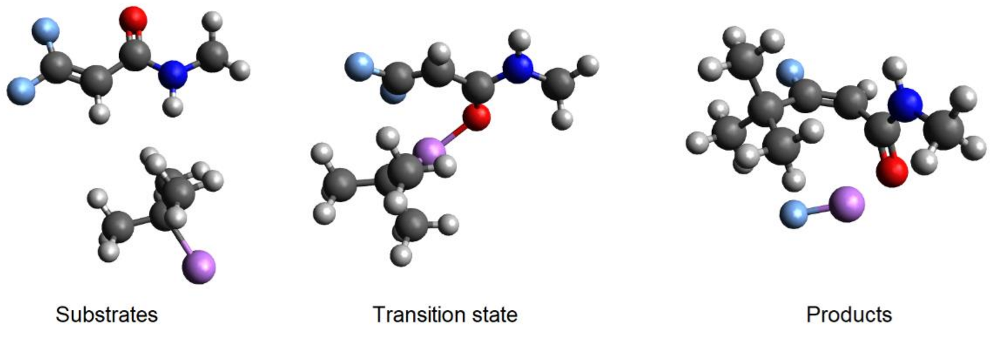

Figure S20. Optimized structures of substrates, transition state and products of 3,3-difluoro-*N*-heptylacrylamide reaction with *tert*-BuLi.

In third step, (*E*)-3-fluoro-*N*-heptyl-4,4-dimethylpent-2-enamide reacts with *tert*-BuLi creating *N*-heptyl-4,4-dimethylpent-2-ynamide (**14a**). Energies for this reaction are gathered in Figure S21. As shown in Figure S21 the formation of transition state

requires overcoming an energy barrier of 3.8 kcal/mol. Creating final product seems to be irreversible because reverse reaction would require 61.7 kcal/mol.

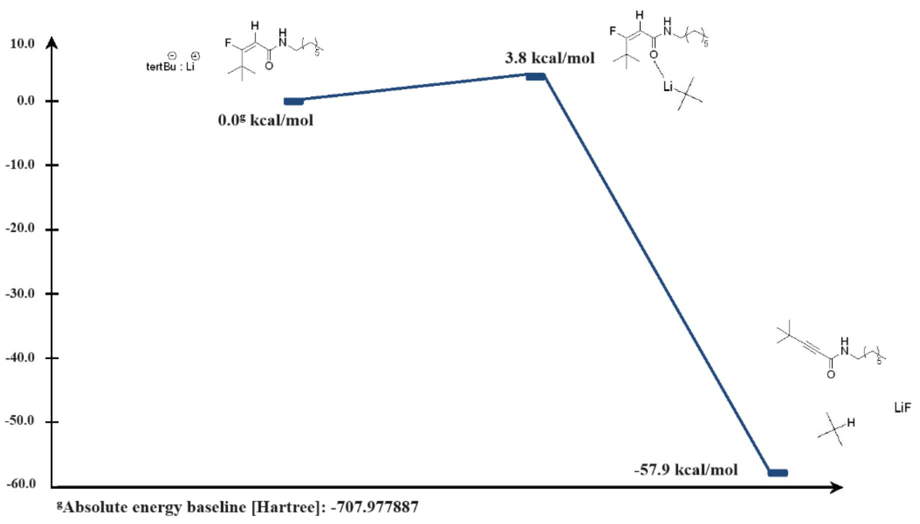

Figure S21. DFT Gibbs free energy calculation of possible reaction pathway of *N*-heptylhept-2-ynamide (**14a**) formation.

Optimized structures of substrates, transition state and products are depicted in Figure S22 (coordinates of those structures are included in Table 11).

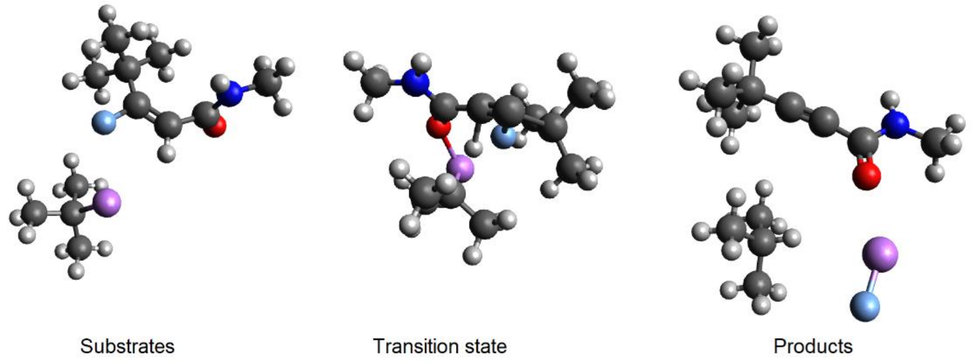

Figure S22. Optimized structures of substrates, transition state and products of (*E*)-3-fluoro-*N*-heptyl-4,4-dimethylpent-2-enamide reaction with *tert*-BuLi.

During our computational exploration, we tested various initial geometries to locate the transition states of each reaction step. However, in many cases, these calculations did not converge to a transition state. The structure presented in the manuscript reflects the only viable transition state obtained from our geometry optimizations, where the

positioning of the lithium atom was dictated by the energetically preferred arrangement for each reaction step.

Table 1. Coordinates of substrates, transition state and products of 2,3,3,3-tetrafluoro-*N*-heptylpropanamide **1a** reaction with n-BuLi

	Substrates			Transition state			Products		
Atom	x	y	z	x	y	z	x	y	z
C	-0.78580	-0.21700	-0.51699	-0.83611	-0.17032	-0.48355	-1.31107	0.17706	-0.54292
C	-0.69565	1.06908	0.29679	-0.68651	1.11129	0.25469	-0.53666	1.09144	0.23404
N	-0.73054	2.21185	-0.38297	-0.65831	2.25238	-0.43929	-0.26454	2.31340	-0.27867
O	-0.58009	1.00386	1.52718	-0.49483	1.07025	1.49101	-0.00388	0.72653	1.33363
C	-0.57421	3.49784	0.27624	-0.33706	3.52338	0.18316	0.73194	3.18968	0.30069
F	-0.84277	0.05805	-1.87063	-0.88775	0.03203	-1.86138	-1.79825	0.71313	-1.73816
H	0.09707	-0.83013	-0.27548	0.14829	-0.86662	-0.02191	2.20066	-4.12521	-0.56155
Li	0.54108	-2.11482	2.66776	0.06090	-0.67483	1.98734	1.14294	-2.06342	0.94254
C	2.07234	-2.97678	1.63692	1.45622	-1.51598	0.64201	3.18065	-4.03682	-0.07176
C	2.93991	-1.89105	0.97905	2.48817	-0.46336	0.22365	3.86541	-2.70206	-0.38467
C	4.17095	-2.37658	0.19885	3.89290	-0.98697	-0.09867	5.21981	-2.55236	0.30918
C	4.97528	-1.24772	-0.44773	4.86693	0.11127	-0.52671	5.87619	-1.20222	0.02696
C	-2.03489	-1.02828	-0.15886	-2.06304	-0.95860	-0.09054	-2.34980	-0.65004	0.10159
F	-1.97318	-1.43878	1.12348	-1.96589	-1.32579	1.22634	-2.69303	-0.63608	0.90260
F	-3.15949	-0.30998	-0.30294	-3.23195	-0.29960	-0.21158	-3.06720	-0.92555	-1.03319
F	-2.13320	-2.10882	-0.93299	-2.16868	-2.08302	-0.80236	-0.69290	2.55247	-1.15983
H	-0.80626	2.16240	-1.38889	-0.80231	2.19520	-1.43687	0.65479	4.16714	-0.17959
H	-0.66248	4.28361	-0.47431	-1.06240	3.76185	0.96555	0.55921	3.30525	1.37293
H	-1.34951	3.62993	1.03491	0.66088	3.48991	0.63067	1.74752	2.80122	0.15424
H	0.40523	3.56208	0.75833	-0.36382	4.30054	-0.58170	3.23351	-1.84670	-0.10141
H	3.28410	-1.17615	1.74600	2.57856	0.30778	1.00794	3.07022	-4.20139	1.01360
H	2.69868	-3.49443	2.38654	1.78806	-1.98005	1.59697	3.99007	-2.61786	-1.47087
H	2.34404	-1.27177	0.27604	2.11813	0.07855	-0.66563	5.88437	-3.36483	-0.01388
H	3.83849	-3.08525	-0.57299	3.81220	-1.74184	-0.89278	3.78125	-4.87901	-0.42852
H	1.86479	-3.76417	0.88581	1.46856	-2.35473	-0.07063	5.08610	-2.67365	1.39302
H	4.81324	-2.95056	0.88070	4.28809	-1.51116	0.78260	6.03663	-1.05851	-1.04794
H	4.35649	-0.68034	-1.15545	4.50472	0.62864	-1.42406	5.24498	-0.38037	0.38468
H	5.33969	-0.54169	0.30961	4.98392	0.86338	0.26392	6.84746	-1.11953	0.52517
H	5.84559	-1.62737	-0.99591	5.86054	-0.29259	-0.75254	-0.40932	-3.00798	1.76975

Table 2. Coordinates of substrates, transition state and products of 2,3,3-trifluoro-*N*-heptylacrylamide reaction with n-BuLi

	Substrates			Transition state			Products		
Atom	x	y	z	x	y	z	x	y	z
C	1.51825	0.35589	0.34720	-1.52537	0.63179	0.36869	0.54570	1.24938	-0.06019

C	0.53654	1.17782	-0.38852	-1.61833	-0.79530	-0.00451	1.59523	0.24444	-0.29423
N	0.06577	2.23634	0.28082	-2.83870	-1.34495	0.03799	2.73289	0.42478	0.38854
O	0.19366	0.89678	-1.54824	-0.59207	-1.43060	-0.30476	1.44207	-0.71154	-1.07723
C	-0.96122	3.10550	-0.26718	-3.02033	-2.78332	-0.09731	3.81929	-0.53668	0.32537
C	2.15310	-0.71658	-0.12962	-0.84142	1.60765	-0.34802	-0.70186	1.24495	-0.55207
H	0.34079	2.34983	1.24610	-3.60901	-0.77256	0.35164	2.78840	1.20016	1.03255
H	-1.95702	2.67760	-0.11430	-2.29844	-3.31674	0.52629	4.11555	-0.70292	-0.71311
H	-0.89917	4.07558	0.22862	-4.03269	-3.03054	0.22405	4.66784	-0.13634	0.88109
H	-0.78994	3.23366	-1.33685	-2.88423	-3.09608	-1.13568	3.51843	-1.49190	0.76838
C	-2.91897	-1.14817	1.21023	2.50324	1.58378	0.61832	-1.37155	0.27254	-1.46136
C	-1.23769	-2.96406	0.49241	4.21067	-1.88898	-0.15113	-2.48444	-0.32641	1.69021
C	-4.16069	-1.84506	0.65489	2.65701	0.14554	0.19117	-2.04266	-0.93436	-0.76779
C	-1.81984	-2.12859	1.64650	4.10214	-0.38068	0.08227	-3.08487	-0.59692	0.30766
H	-1.96643	-3.74495	0.21648	3.74237	-2.17856	-1.10055	-1.87344	0.58197	1.71189
H	-0.37433	-3.53451	0.88400	3.70873	-2.44735	0.64955	-1.85341	-1.16763	1.99764
H	-2.51298	-0.47906	0.43310	5.25605	-2.21798	-0.18089	-3.27621	-0.19901	2.43664
H	-4.58353	-2.52897	1.40142	2.17005	-0.06370	-0.78607	-1.29265	-1.59635	-0.31572
H	-4.94036	-1.12627	0.37472	2.17200	-0.48068	0.94559	-2.52120	-1.50751	-1.57126
H	-3.90932	-2.43887	-0.23086	2.73042	1.72158	1.68232	-0.63788	-0.09053	-2.18506
H	-1.01738	-1.53643	2.12053	4.60581	0.16185	-0.72882	-3.70334	0.25260	-0.01430
H	-2.23934	-2.73875	2.47050	4.61911	-0.09109	1.00463	-3.76075	-1.45562	0.39346
H	-3.21310	-0.50725	2.05610	3.16468	2.22071	0.01790	-2.12869	0.84467	-2.01064
Li	-0.40034	-2.18353	-0.66177	0.45588	0.09094	0.25748	0.81759	-2.42817	-0.66773
F	2.05396	-1.16166	-1.35597	0.38059	1.34028	-0.72755	0.03905	-3.20450	0.53655
F	2.98308	-1.42681	0.58571	-1.64588	2.55285	-0.71247	-1.48401	2.26340	-0.16735
F	1.76463	0.71021	1.62698	-2.61368	1.15741	0.99256	0.91037	2.24890	0.78236

Table 3. Coordinates of substrates, transition state and products of (*Z*)-2,3-difluoro-*N*-heptylhept-2-enamide **9a** reaction with n-BuLi

	Substrates			Transition state			Products		
Atom	x	y	z	x	y	z	x	y	z
C	1.68129	-0.50101	0.55637	1.04776	0.58969	0.57466	0.40555	-0.95686	0.35027
C	3.04196	-0.30450	-0.02195	2.23610	1.02491	-0.15089	1.86609	-1.02336	0.12733
N	3.70054	-1.44645	-0.31395	2.88240	2.10696	0.36206	2.33793	-2.19646	-0.32344
O	3.50506	0.81466	-0.21280	2.63126	0.43863	-1.16023	2.63855	-0.07756	0.35538
C	5.03371	-1.40861	-0.88591	4.08118	2.62031	-0.26827	3.75687	-2.41640	-0.53080
C	0.87077	0.47107	0.97900	0.04652	-0.19851	0.11177	-0.40959	0.08302	0.57896
H	3.25987	-2.33370	-0.12466	2.50966	2.56263	1.17949	1.68410	-2.94939	-0.47429
H	5.73510	-0.92482	-0.19981	3.87176	2.97004	-1.28426	4.30940	-2.28151	0.40370
H	5.35969	-2.43238	-1.07476	4.45858	3.45334	0.32818	3.89956	-3.43632	-0.89018
H	5.03054	-0.84947	-1.82580	4.84896	1.84296	-0.32754	4.15070	-1.71436	-1.27093
C	1.04227	1.94824	1.00003	0.06843	-1.13136	-1.04427	-0.02031	1.54511	0.56216
C	-1.15203	1.19497	-1.50892	1.28971	-4.56321	0.43662	-0.22981	0.87302	-2.72928
C	0.56662	2.67397	-0.27474	0.91950	-2.39308	-0.82262	0.68728	2.13464	-0.67216

C	-0.90302	2.44436	-0.65582	0.47966	-3.27002	0.35026	-0.13657	2.19511	-1.96475
H	-0.70861	0.28345	-1.08330	2.35826	-4.34826	0.55626	-0.73262	0.09511	-2.14840
H	-0.69844	1.30717	-2.49893	1.17192	-5.16426	-0.47313	0.77188	0.50199	-2.98049
H	-2.22631	1.02462	-1.64440	0.97339	-5.17680	1.28723	-0.78422	0.99940	-3.66599
H	1.22381	2.38744	-1.10419	1.96415	-2.08707	-0.69664	1.62139	1.60112	-0.88132
H	0.73538	3.74198	-0.09801	0.87365	-2.97972	-1.74990	0.97327	3.15123	-0.37903
H	2.10181	2.16468	1.15125	0.46311	-0.57863	-1.89995	0.58631	1.78556	1.45026
H	-1.53136	2.42910	0.24852	0.58679	-2.71269	1.28910	-1.14477	2.57034	-1.74053
H	-1.26374	3.30807	-1.22477	-0.58842	-3.50881	0.25180	0.32120	2.94288	-2.62474
H	0.48699	2.32012	1.86993	-0.97106	-1.40043	-1.26227	-0.94556	2.11712	0.70428
F	-0.34202	0.04940	1.46520	-1.44227	1.29297	1.14083	3.47838	3.94416	2.34730
F	1.26240	-1.78489	0.64234	-0.87561	-0.55942	1.11115	-0.14153	-2.22223	0.31905
C	-5.12585	0.00152	0.64149	0.66588	1.40471	1.65879	-1.85659	-0.19752	0.91826
C	-3.86281	-2.74630	-0.03150	-1.67206	1.40804	-0.88959	-4.74315	-1.35029	0.70484
C	-5.66747	-0.94734	-0.44415	-5.38603	0.56139	0.06802	-2.81167	0.03968	-0.26232
C	-5.35534	-2.43147	-0.19139	-3.08687	0.84753	-1.05061	-4.28963	-0.00413	0.13815
H	-3.49164	-2.46263	0.96280	-3.92274	0.97024	0.23229	-4.25709	-1.57533	1.66042
H	-3.26591	-2.23006	-0.79923	-5.45942	-0.47601	-0.27821	-4.50968	-2.16671	0.01036
H	-3.65664	-3.81769	-0.13424	-5.88211	1.19569	-0.67515	-5.82438	-1.35688	0.87851
H	-5.23127	-0.68560	-1.42607	-5.94156	0.64410	1.00876	-2.61885	-0.71817	-1.03317
H	-6.76116	-0.87410	-0.59270	-3.03858	-0.21903	-1.32010	-2.59591	1.01380	-0.71831
H	-5.41190	1.03341	0.37155	-3.65358	1.33211	-1.86583	-2.13141	0.48042	1.73809
H	-5.88161	-2.76209	0.71485	-1.07538	1.24821	-1.79211	-4.48815	0.78938	0.87150
H	-5.75404	-3.03523	-1.01858	-3.48087	0.33625	1.02196	-4.89299	0.23580	-0.74623
H	-5.67257	-0.20598	1.58010	-3.88163	2.01449	0.58732	-1.97446	-1.21760	1.28880
Li	-3.15998	-0.30839	0.75334	-1.72710	2.51206	-0.77382	3.98974	2.72086	1.38436

Table 4. Coordinates of substrates, transition state and products of 3,3,3-trifluoro-*N*-heptylpropanamide **2a** reaction with n-BuLi

	Substrates			Transition state			Products		
Atom	x	y	z	x	y	z	x	y	z
C	-0.87476	-0.20182	-0.70204	-0.90007	-0.14949	-0.66270	-1.35280	-1.29970	0.20884
C	-0.75518	1.07264	0.11020	-0.74582	1.12655	0.07402	-0.29148	-1.24171	-0.81047
N	-0.74235	2.23388	-0.55652	-0.60713	2.27275	-0.61546	0.48985	-2.33526	-0.89818
O	-0.65207	1.02934	1.34312	-0.64155	1.11961	1.32159	-0.10317	-0.26995	-1.55358
C	-0.54362	3.50155	0.12674	-0.26593	3.52406	0.03574	1.61560	-2.38818	-1.81494
H	-0.89055	-0.02585	-1.78029	-0.83735	-0.06739	-1.74901	-1.46636	-2.17871	0.83210
H	-0.00820	-0.82981	-0.43518	0.04615	-0.83847	-0.14881	3.36951	4.45979	-0.05495
Li	0.61060	-1.71783	2.86562	-0.17203	-0.65837	1.82453	-1.36237	3.94324	-1.36817
C	2.20758	-2.58028	1.93312	1.33738	-1.50279	0.59870	3.84749	3.55410	0.33540
C	3.09834	-1.49450	1.30842	2.39188	-0.48655	0.15176	3.12786	3.05567	1.58748
C	4.35944	-1.97834	0.57579	3.80094	-1.03781	-0.09929	3.70146	1.74815	2.13449
C	5.18926	-0.84892	-0.03724	4.79725	0.02624	-0.56162	2.97323	1.24388	3.37979
C	-2.11505	-0.99000	-0.34079	-2.11838	-0.92724	-0.29542	-2.20559	-0.30072	0.43288

F	-2.09900	-1.40359	0.94706	-2.08792	-1.32560	1.02651	-1.59529	5.55770	-1.25435
F	-3.24334	-0.26974	-0.50479	-3.29279	-0.26889	-0.43232	-2.24254	0.84411	-0.19038
F	-2.22434	-2.08511	-1.10665	-2.21976	-2.05282	-1.01949	-3.14350	-0.35738	1.34848
H	-0.82757	2.22423	-1.56101	-0.74036	2.25397	-1.61417	0.34673	-3.09709	-0.25313
H	-0.56746	4.30167	-0.61382	-0.13722	4.29083	-0.72987	2.08151	-3.37100	-1.73438
H	-1.33400	3.66375	0.86398	-1.05638	3.83167	0.72634	1.27650	-2.23025	-2.84161
H	0.42186	3.50819	0.64002	0.66549	3.41675	0.59905	2.35128	-1.61651	-1.56745
H	3.41276	-0.77843	2.08690	2.46599	0.32669	0.89363	2.05670	2.92237	1.37668
H	2.80648	-3.10305	2.70135	1.64288	-1.91524	1.58546	3.83531	2.78741	-0.45267
H	2.52716	-0.87754	0.58330	2.05443	0.00790	-0.77816	3.17351	3.82927	2.36440
H	4.05792	-2.68666	-0.20919	3.73688	-1.83768	-0.84995	4.76585	1.89278	2.36432
H	2.02012	-3.36168	1.17023	1.35532	-2.37848	-0.06891	4.89933	3.77710	0.54974
H	4.97514	-2.55300	1.28123	4.16615	-1.51251	0.82199	3.66408	0.97969	1.34862
H	4.59890	-0.27940	-0.76758	4.46587	0.49372	-1.49768	3.02495	1.98190	4.18853
H	5.52379	-0.14411	0.73482	4.89783	0.82222	0.18718	1.91114	1.06458	3.16991
H	6.08039	-1.22759	-0.55190	5.79353	-0.39694	-0.73462	3.40708	0.30911	3.75302

Table 5. Coordinates of substrates, transition state and products of 3,3-difluoro-*N*-heptylacrylamide reaction with n-BuLi

	Substrates			Transition state			Products		
Atom	x	y	z	x	y	z	x	y	z
C	1.57797	-0.41533	-0.68774	-1.52537	0.63179	0.36869	-1.08117	-0.95167	-0.36997
C	0.67278	-1.28151	0.08600	-1.61833	-0.79530	-0.00451	-2.20554	-0.04870	-0.04725
N	0.14954	-2.32362	-0.58445	-2.83870	-1.34495	0.03799	-3.43619	-0.49831	-0.36386
H	1.67023	-0.53680	-1.76022	-0.59207	-1.43060	-0.30476	-1.18950	-1.62700	-1.21232
O	0.39139	-1.08408	1.28054	-3.02033	-2.78332	-0.09731	-2.05644	1.07984	0.45101
C	-0.82460	-3.21252	0.02453	-0.84142	1.60765	-0.34802	-4.62509	0.30490	-0.14178
C	2.26745	0.58491	-0.14494	-3.60901	-0.77256	0.35164	0.32363	-1.08049	0.27620
H	0.35233	-2.42395	-1.56728	-2.29844	-3.31674	0.52629	-3.53571	-1.44513	-0.69642
H	-1.76550	-2.68682	0.21459	-4.03269	-3.03054	0.22405	-4.51448	1.27879	-0.62514
H	-1.00578	-4.04570	-0.65598	-2.88423	-3.09608	-1.13568	-5.48219	-0.21570	-0.57171
H	-0.43825	-3.59625	0.97129	2.50324	1.58378	0.61832	-4.79592	0.46602	0.92697
C	-3.35579	2.16206	-0.71501	4.21067	-1.88898	-0.15113	0.47076	0.32999	0.72649
C	-1.60466	3.87316	0.09567	2.65701	0.14554	0.19117	4.17992	0.82085	1.92522
C	-4.53669	2.77804	0.03456	4.10214	-0.38068	0.08227	1.68698	0.54285	1.65740
C	-2.28413	3.19147	-1.10524	3.74237	-2.17856	-1.10055	3.03007	0.64712	0.93568
H	-2.29718	4.61968	0.52051	3.70873	-2.44735	0.64955	4.24135	-0.05933	2.57340
H	-0.76184	4.47938	-0.28679	5.25605	-2.21798	-0.18089	4.03875	1.71066	2.55274
H	-2.89401	1.39114	-0.07600	2.17005	-0.06370	-0.78607	5.13761	0.92723	1.40369
H	-5.01165	3.55918	-0.57234	2.17200	-0.48068	0.94559	1.74144	-0.28572	2.37213
H	-5.29987	2.02905	0.27914	2.73042	1.72158	1.68232	1.50560	1.46888	2.21942
H	-4.20330	3.24392	0.96851	4.60581	0.16185	-0.72882	-0.45090	0.60474	1.24964
H	-1.52459	2.66337	-1.70783	4.61911	-0.09109	1.00463	3.20446	-0.26687	0.36142
H	-2.76074	3.90187	-1.80990	3.16468	2.22071	0.01790	3.00405	1.48891	0.22745

H	-3.72901	1.64447	-1.61249	0.45588	0.09094	0.25748	0.55367	0.97695	-0.15704
Li	-0.99437	2.46231	1.42834	0.38059	1.34028	-0.72755	-1.30200	5.88361	-0.60164
F	2.31707	0.89903	1.12591	-1.64588	2.55285	-0.71247	-0.04364	5.08714	0.10250
F	3.03892	1.38476	-0.84103	-2.61368	1.15741	0.99256	1.27482	-1.46878	-0.59992

Table 6. Coordinates of substrates, transition state and products of (*E*)-3-fluoro-*N*-heptylhept-2-enamide **11a** reaction with n-BuLi

	Substrates			Transition state			Products		
Atom	x	y	z	x	y	z	x	y	z
C	-0.40063	-1.40650	-0.72479	1.08216	-1.61790	-0.27403	1.56832	-1.62988	0.13017
C	-1.59104	-1.27571	0.13693	2.30873	-0.89762	-0.67448	2.79765	-0.86776	0.10031
N	-2.77955	-1.32622	-0.49699	3.11854	-0.47079	0.34733	3.85590	-1.47229	-0.46485
H	-0.53397	-1.33669	-1.79951	2.62924	-0.71574	-1.84733	-0.01986	1.34394	-0.88593
O	-1.53460	-1.10785	1.36624	4.37019	0.20261	0.07563	2.87263	0.27460	0.56552
C	-4.02739	-1.07157	0.19976	0.08098	-1.16474	0.51851	5.14508	-0.81041	-0.57747
C	0.85861	-1.52932	-0.29161	2.97781	-0.89240	1.25223	0.51341	-2.21858	0.17639
H	-2.79162	-1.39477	-1.50290	4.19698	1.03045	-0.61694	3.73806	-2.41046	-0.81743
H	-4.08816	-0.02598	0.51893	4.77088	0.59846	1.01265	5.58351	-0.64434	0.41078
H	-4.85447	-1.29284	-0.47657	5.10878	-0.46780	-0.38079	5.80823	-1.44509	-1.16641
H	-4.09901	-1.71244	1.08114	-0.00205	-0.02964	1.51572	5.03239	0.15600	-1.07554
C	2.09393	-1.53534	-1.12399	1.08056	3.33201	-0.24852	-0.79779	-2.85996	0.23804
C	4.49343	1.27553	0.22242	0.67864	1.31734	1.21914	-4.44757	-1.47758	-0.01466
C	2.89349	-0.21718	-1.04636	0.36870	1.98601	-0.11916	-1.93802	-1.84364	0.05791
C	3.64213	0.00783	0.26781	2.16778	3.20471	-0.18150	-3.31778	-2.49278	0.15566
H	3.86428	2.15625	0.05101	0.77508	4.02362	0.54673	-4.38651	-0.97929	-0.98894
H	5.23883	1.22716	-0.58134	0.86041	3.80614	-1.21071	-4.39640	-0.70126	0.75690
H	5.02623	1.42619	1.16709	1.76231	1.20542	1.30225	-5.42757	-1.96043	0.05691
H	2.21435	0.62498	-1.22974	0.37390	1.99366	2.03035	-1.82528	-1.34975	-0.91473
H	3.61672	-0.23156	-1.87255	0.40023	-0.41113	2.46523	-1.83919	-1.05875	0.81667
H	1.79526	-1.71973	-2.16084	0.68219	1.33470	-0.94221	-0.85824	-3.64027	-0.53070
H	2.92477	0.09097	1.09073	-0.71447	2.13039	-0.22352	-3.40800	-3.27710	-0.60828
H	4.27914	-0.86308	0.47780	-1.06601	0.15939	1.69625	-3.41762	-2.99276	1.12865
H	2.72686	-2.37517	-0.80959	-2.24614	-1.85640	0.54295	-0.89508	-3.36501	1.20770
Li	-0.10998	1.79683	1.83296	-0.66613	-2.29524	1.19135	2.54382	2.13197	0.66887
F	1.11032	-1.64300	1.03170	0.87652	-2.49606	-0.87957	2.68458	3.74314	0.41940
C	0.48801	3.54644	0.96377	-1.59636	-0.76087	-0.97938	-0.18162	2.10679	-0.11279
C	-2.81433	4.00200	-1.12748	-5.11097	0.79525	-0.08235	-4.08733	2.41657	-0.29873
C	-0.40198	3.80614	-0.26552	-3.12081	-0.49855	-1.05519	-1.55752	2.75649	-0.26013
C	-1.88938	3.91662	0.08716	-3.61919	0.48238	0.01522	-2.71462	1.76459	-0.14307
H	-2.72283	3.10127	-1.75084	-5.71478	-0.11614	0.01001	-4.18172	2.90369	-1.27658
H	-2.55894	4.86171	-1.75884	-5.34717	1.24995	-1.05091	-4.24764	3.18229	0.46953
H	-3.86794	4.10314	-0.83755	-5.42947	1.49035	0.70178	-4.89417	1.67974	-0.21261
H	-0.29050	2.98371	-0.99635	-3.70818	-1.44059	-0.96359	-1.61484	3.26570	-1.23147
H	-0.13413	4.72237	-0.82841	-3.42085	-0.11574	-2.04325	-1.67033	3.53879	0.50200

H	1.54372	3.57160	0.64105	-1.30639	-1.55744	-1.68086	0.60461	2.86829	-0.19424
H	-2.17569	3.05446	0.70728	-3.40101	0.09488	1.02831	-2.59459	0.97952	-0.90254
H	-2.03611	4.80009	0.72413	-3.03319	1.40644	-0.06250	-2.65700	1.25940	0.83168
H	0.39080	4.41781	1.63805	-1.06494	0.13891	-1.27981	-0.09110	1.60915	0.86335

Table 7. Coordinates of substrates, transition state and products of 2,3,3,3-tetrafluoro-*N*-heptylpropanamide **1a** reaction with *tert*-BuLi.

	Substrates			Transition state			Products		
Atom	x	y	z	x	y	z	x	y	z
C	0.64300	-0.33800	0.56100	-0.50300	0.34900	0.51400	-1.50600	-0.44700	0.44600
C	1.21300	0.88300	-0.14700	-1.27600	-0.72000	-0.18100	-0.59200	-1.24200	-0.25800
N	2.26600	1.47100	0.41200	-2.31500	-1.27200	0.45000	-0.13900	-2.38100	0.33900
O	0.67100	1.28700	-1.18500	-0.86900	-1.12100	-1.29300	-0.14700	-0.90000	-1.40700
C	2.86000	2.67400	-0.14800	-3.03300	-2.40900	-0.09800	0.69600	-3.35100	-0.33300
F	1.26200	-0.53600	1.78100	-0.97100	0.56000	1.80900	-1.75100	-0.73100	1.77400
H	-0.44600	-0.17200	0.67100	0.67800	-0.04600	0.37500	1.69986	1.26431	-0.26770
Li	-2.37561	0.73704	-1.43224	0.92100	-0.54500	-1.58200	-0.49817	3.64270	-2.58993
C	-3.78078	0.47838	-0.02242	2.21600	-0.66300	0.09000	2.76786	1.44631	-0.45370
C	-3.65561	1.57704	1.14876	2.05600	-1.86800	1.01200	3.49586	0.11431	-0.27270
C	-4.88061	1.00604	-0.92324	3.05800	-1.08300	-1.12800	2.95686	1.96931	-1.88370
C	-4.23661	-0.78696	0.65476	2.94900	0.46000	0.81700	3.23186	2.49031	0.56330
C	0.83800	-1.61400	-0.26400	-0.54000	1.67900	-0.20800	-2.01100	0.79200	-0.03800
F	0.17300	-1.51800	-1.43400	0.07800	1.55200	-1.42300	-1.47899	4.53492	-1.35631
F	2.13200	-1.83100	-0.55100	-1.77600	2.15100	-0.46000	-2.55600	0.79100	-1.27600
F	0.37600	-2.67800	0.38800	0.11500	2.62300	0.46900	-2.80300	1.44400	0.79100
H	2.63600	1.07000	1.26300	-2.57100	-0.89300	1.35000	-0.58200	-2.64500	1.20600
H	3.30000	2.46500	-1.12600	-3.51900	-2.14100	-1.04000	0.12900	-4.24700	-0.61200
H	2.09800	3.44900	-0.26300	-2.34500	-3.23900	-0.28400	1.08700	-2.89400	-1.24300
H	3.63600	3.02600	0.53300	-3.78900	-2.72100	0.62200	1.53300	-3.64400	0.30800
H	-3.37561	2.56904	0.76076	1.56400	-2.71100	0.50300	3.13486	-0.62869	-0.99170
H	-4.60761	1.71604	1.70676	3.02300	-2.24400	1.39900	4.57686	0.23831	-0.41870
H	-2.90161	1.30604	1.90576	1.44200	-1.62200	1.89200	3.33286	-0.28669	0.73330
H	-5.07361	0.27804	-1.72924	3.20400	-0.25800	-1.85000	2.38386	2.89331	-2.06270
H	-5.85661	1.16004	-0.41224	4.07800	-1.41000	-0.85000	4.00686	2.22531	-2.06770
H	-4.64461	1.97204	-1.40324	2.62700	-1.94200	-1.67700	2.69986	1.20631	-2.63470
H	-4.37261	-1.57396	-0.10124	3.10300	1.33500	0.16900	2.68786	3.43431	0.44230
H	-3.52161	-1.17996	1.39576	2.38600	0.80900	1.69500	3.06786	2.13731	1.58630
H	-5.20561	-0.68696	1.19176	3.94600	0.14700	1.18300	4.30286	2.69631	0.44430

Table 8. Coordinates of substrates, transition state and products of 2,3,3-trifluoro-*N*-heptylacrylamide reaction with *tert*-BuLi.

	Substrates			Transition state			Products		
Atom	x	y	z	x	y	z	x	y	z

C	-1.35293	0.73399	-0.22040	-0.87977	1.07996	-0.16854	-0.54980	0.63755	0.29946
C	-1.37471	-0.61098	0.38961	-1.61423	-0.09190	0.30886	-1.63949	-0.15402	0.00208
N	-1.99888	-1.55334	-0.32327	-2.73667	-0.40527	-0.35573	-2.79237	0.45797	-0.45199
F	-1.96204	0.86427	-1.41742	-1.22773	1.57464	-1.38018	-0.69670	2.01007	0.13772
O	-0.85319	-0.83182	1.49433	-1.19546	-0.76788	1.26813	-1.65322	-1.44277	0.12179
C	-1.97282	-2.94798	0.08395	-3.47176	-1.62360	-0.06725	-4.06989	-0.21219	-0.31509
C	-0.75826	1.81415	0.28973	0.22176	1.57227	0.42155	0.84932	0.25482	0.57953
F	-0.12605	1.84560	1.43671	0.52521	1.37600	1.69714	0.84140	-0.88520	1.53391
F	-0.75611	2.97575	-0.30456	0.93887	2.52621	-0.10184	1.44928	1.24703	1.28551
H	-2.32904	-1.31499	-1.24761	-2.98000	0.14183	-1.16842	-2.80086	1.46333	-0.35868
H	-2.27674	-3.03404	1.12875	-3.68410	-1.68629	1.00205	-4.38818	-0.32019	0.73257
H	-2.66993	-3.50511	-0.54278	-4.41145	-1.60041	-0.62064	-4.82428	0.36578	-0.85553
H	-0.96616	-3.36300	-0.03015	-2.89629	-2.50715	-0.36274	-4.00908	-1.20728	-0.75857
C	2.25593	-0.40558	-0.04085	1.94505	-0.89630	-0.16549	1.79433	-0.18697	-0.56004
C	1.65233	-1.34430	-1.09212	1.06092	-1.44385	-1.28126	1.28027	-1.47926	-1.21006
C	3.61637	-0.98446	0.36114	2.65705	-2.08134	0.50623	3.22058	-0.40401	-0.03216
C	2.50656	0.94362	-0.71444	3.00205	0.01859	-0.76418	1.79208	0.93717	-1.60940
H	1.45003	-2.35059	-0.68949	0.31036	-2.16347	-0.91175	1.38648	-2.34004	-0.53504
H	2.32437	-1.48998	-1.96887	1.64043	-1.97989	-2.06482	1.88295	-1.70408	-2.09666
H	0.70300	-0.96052	-1.50263	0.51104	-0.64495	-1.80295	0.23470	-1.39683	-1.51876
H	4.30062	-1.10055	-0.51121	3.25515	-2.68650	-0.21012	3.86505	-0.73596	-0.85413
H	4.14315	-0.34334	1.08376	3.35553	-1.75377	1.29118	3.64236	0.51793	0.37713
H	3.52903	-1.98293	0.81846	1.95737	-2.80460	0.97121	3.24937	-1.16954	0.74967
H	1.58343	1.39460	-1.11422	2.55940	0.85567	-1.32140	0.79726	1.06943	-2.04601
H	3.20076	0.86046	-1.58204	3.66264	-0.51758	-1.48164	2.49259	0.68446	-2.41300
H	2.95343	1.67694	-0.02702	3.65846	0.45356	0.00298	2.10376	1.89083	-1.17292
Li	1.01156	-0.44011	1.57366	0.70943	-0.87372	1.44885	-0.42331	-2.16236	1.13315

Table 9. Coordinates of substrates, transition state and products of 3,3,3-trifluoro-*N*-heptylpropanamide **2a** reaction with *tert*-BuLi

	Substrates			Transition state			Products		
Atom	x	y	z	x	y	z	x	y	z
C	-0.72155	-0.36953	-0.75425	0.57056	0.39224	-0.73363	2.00470	-0.51720	-0.68354
C	-1.32825	0.81745	-0.03506	1.43288	-0.59140	-0.02429	0.98085	-1.28099	0.04010
N	-2.38475	1.41717	-0.59760	2.50903	-1.09336	-0.65413	0.66593	-2.48611	-0.47463
O	-0.83963	1.22302	1.02932	1.10278	-1.00812	1.10649	0.38993	-0.85637	1.05000
C	-3.01179	2.58321	0.00316	3.33032	-2.13476	-0.06339	-0.39660	-3.30024	0.08765
H	-1.11230	-0.50905	-1.76508	0.82666	0.55260	-1.78265	2.20728	-0.72608	-1.72674
H	0.36980	-0.19248	-0.78304	-0.55538	-0.08321	-0.56698	-1.56971	0.15551	-0.40400
Li	1.78193	0.57707	1.14927	-0.72892	-0.56377	1.40349	-0.01332	1.02973	1.02209
C	3.28432	0.52230	-0.12708	-2.12353	-0.75568	-0.11289	-2.56862	0.55541	-0.18022
C	3.23184	1.55720	-1.28441	-2.19008	-2.25499	0.19179	-2.88507	1.59102	-1.25875
C	4.40308	0.94218	0.80201	-3.06488	-0.00682	0.83674	-3.55140	-0.61701	-0.19826
C	3.80660	-0.81343	-0.83167	-2.58000	-0.51826	-1.54494	-2.53918	1.21125	1.20566

C	-0.93190	-1.65689	0.01239	0.48256	1.71552	-0.04208	2.62781	0.53340	-0.14716
F	-0.33022	-1.62592	1.22450	-0.07089	1.59544	1.20852	0.43563	2.17164	-0.08825
F	-2.23985	-1.90826	0.23571	1.66908	2.33765	0.15023	2.57454	0.89595	1.10732
F	-0.43176	-2.70354	-0.65486	-0.29838	2.57150	-0.72015	3.47604	1.27503	-0.80809
H	-2.77509	1.02854	-1.44226	2.79525	-0.67342	-1.52474	1.18185	-2.82809	-1.27065
H	-3.47203	2.32476	0.96093	4.04029	-2.48698	-0.81345	-0.20587	-3.50295	1.14473
H	-2.26470	3.36235	0.17258	3.87726	-1.75900	0.80693	-1.35995	-2.78910	-0.00075
H	-3.77614	2.95632	-0.67945	2.70035	-2.96826	0.25663	-0.43829	-4.24319	-0.45954
H	2.93407	2.53844	-0.88265	-1.88758	-2.50827	1.22726	-2.93582	1.12371	-2.24923
H	4.20083	1.71329	-1.80988	-3.21283	-2.67084	0.08077	-3.85323	2.07227	-1.06540
H	2.50172	1.30052	-2.07090	-1.53198	-2.83196	-0.47220	-2.10556	2.35918	-1.28176
H	4.57325	0.20122	1.59972	-3.07857	1.07085	0.62857	-3.31755	-1.34670	0.58718
H	5.39201	1.10158	0.31536	-4.11161	-0.36806	0.77589	-4.57751	-0.26514	-0.02999
H	4.15393	1.89894	1.29258	-2.79949	-0.11498	1.91061	-3.53212	-1.13535	-1.16438
H	3.92779	-1.61820	-0.09185	-2.55481	0.54930	-1.80745	-2.17119	0.51547	1.97443
H	3.11371	-1.19299	-1.60057	-1.93664	-1.04385	-2.26693	-1.92641	2.12307	1.19936
H	4.79049	-0.69544	-1.33897	-3.61494	-0.87061	-1.72699	-3.54791	1.51460	1.51118

Table 10. Coordinates of substrates, transition state and products of 3,3-difluoro-*N*-heptylacrylamide reaction with *tert*-BuLi

	Substrates			Transition state			Products		
Atom	x	y	z	x	y	z	x	y	z
C	-1.34982	0.45001	-0.23758	0.96008	1.08957	0.58377	0.41954	0.22329	-1.31313
C	-1.57150	-1.00689	-0.04748	1.79876	0.05582	-0.03181	1.63833	-0.08512	-0.51280
N	-0.46992	-1.76412	-0.31128	2.99104	-0.17761	0.54897	2.37491	0.95947	-0.10975
H	-0.37801	0.82379	-0.54236	1.05604	1.31988	1.63774	0.57022	0.20141	-2.39014
O	-2.64094	-1.49451	0.29865	1.43185	-0.61495	-1.01632	2.00900	-1.25083	-0.29409
C	-0.46431	-3.19015	-0.06444	3.87935	-1.22537	0.07888	3.58535	0.79504	0.67612
C	-2.29750	1.36317	-0.05527	-0.07175	1.63578	-0.06737	-0.81922	0.45186	-0.87032
H	0.41875	-1.30238	-0.44773	3.28847	0.42010	1.30475	2.01526	1.88456	-0.29142
H	-1.42036	-3.61348	-0.37906	3.35785	-2.18602	0.06998	4.28401	0.13474	0.15686
H	0.34215	-3.64770	-0.64265	4.23341	-1.01260	-0.93416	4.04677	1.77356	0.81526
H	-0.32191	-3.41832	0.99942	4.73304	-1.28561	0.75547	3.35452	0.35816	1.65293
C	3.34873	0.37445	0.08401	-1.98317	-0.82298	0.04849	-1.44877	0.54182	0.49972
C	3.01347	1.86992	0.12419	-1.57771	-0.90199	1.51511	-1.87652	2.00770	0.72001
C	2.59155	-0.29692	1.23602	-2.19432	-2.26224	-0.45280	-0.49030	0.10787	1.61529
C	2.82072	-0.18675	-1.23514	-3.30821	-0.08050	-0.07002	-2.68027	-0.38781	0.53206
H	3.33102	2.34948	1.06179	-0.59986	-1.39413	1.65481	-1.00967	2.68033	0.72802
H	1.91615	2.03940	0.04758	-2.30630	-1.47575	2.13073	-2.38188	2.09432	1.68801
H	3.47208	2.43148	-0.70285	-1.50306	0.09371	1.97684	-2.57057	2.34609	-0.05532
H	1.50039	-0.07865	1.20190	-2.96688	-2.81179	0.12996	-1.00663	0.21504	2.57518
H	2.69471	-1.39259	1.22692	-2.53017	-2.29840	-1.50345	-0.22481	-0.94860	1.50834
H	2.92947	0.05180	2.22320	-1.27871	-2.87762	-0.37960	0.41248	0.72726	1.66365
H	3.32061	0.24948	-2.11170	-3.23612	0.95499	0.29144	-3.41796	-0.10533	-0.22444

H	1.73654	0.03363	-1.38128	-4.12046	-0.56418	0.51907	-3.15442	-0.30342	1.51673
H	2.93626	-1.27861	-1.30834	-3.66482	-0.03198	-1.10977	-2.36773	-1.42823	0.38246
Li	5.34044	0.14383	0.34755	-0.45196	-0.74582	-1.29054	0.65964	-2.59593	-0.23418
F	-3.53694	1.16038	0.30716	-0.25717	1.59817	-1.37665	-0.84813	-2.96048	0.29443
F	-2.08864	2.65796	-0.23201	-0.91137	2.47085	0.48666	-1.72896	0.68497	-1.84824

Table 11. Coordinates of substrates, transition state and products of (*E*)-3-fluoro-*N*-heptyl-4,4-dimethylpent-2-enamide reaction with *tert*-BuLi

	Substrates			Transition state			Products		
Atom	x	y	z	x	y	z	x	y	z
C	-1.55354	0.85530	0.83823	0.97800	-0.69700	-0.66300	0.89866	-1.17464	0.09069
C	-2.78245	1.15376	0.02238	0.22200	-1.61100	0.18700	-0.26799	-2.02873	0.07970
N	-3.92497	0.59147	0.48752	0.08700	-2.83700	-0.34700	-0.14726	-3.18979	-0.58488
H	-1.29535	1.60937	1.57769	-0.07900	0.08800	0.24300	-2.06379	1.59885	-0.77816
O	-2.74799	1.89643	-0.94570	-0.31500	-1.31200	1.26200	-1.32151	-1.71489	0.64854
C	-5.19664	0.80799	-0.17804	-0.79700	-3.82800	0.24000	-1.24721	-4.13467	-0.67761
C	-0.74229	-0.18279	0.69676	1.33500	-0.05900	-1.68600	1.81936	-0.39159	0.13999
H	-1.97573	-2.62788	1.07443	0.56000	-3.03000	-1.21800	0.73499	-3.39560	-1.02919
H	-5.19865	0.35496	-1.17496	-0.81800	-4.70200	-0.41300	-1.54937	-4.46933	0.31846
H	-5.98859	0.36063	0.42499	-0.44100	-4.12300	1.23100	-2.10930	-3.67132	-1.16511
H	-5.38631	1.87881	-0.28798	-1.80900	-3.42500	0.33900	-0.91698	-4.99284	-1.26409
C	-0.68408	-1.39947	-0.19309	2.20000	1.44300	-0.59200	2.91684	0.58483	0.21976
C	-1.71477	-1.30961	-1.32745	3.56100	1.28100	-1.27300	2.67964	1.68370	-0.83469
C	-0.96032	-2.65149	0.66305	2.32100	0.96400	0.86800	4.25411	-0.12897	-0.05022
C	0.72600	-1.49437	-0.81274	1.66400	2.86700	-0.61300	2.91988	1.20587	1.63025
H	-2.74175	-1.29964	-0.95029	3.84900	0.22800	-1.34300	2.65015	1.26413	-1.84548
H	-1.60860	-2.19227	-1.96624	4.30800	1.80000	-0.65700	3.49766	2.41106	-0.78574
H	-1.56285	-0.42039	-1.94609	3.56400	1.71400	-2.27400	1.73758	2.20880	-0.65215
H	-0.86554	-3.54833	0.04145	3.11700	1.56900	1.31900	5.07395	0.59490	0.01526
H	-0.25232	-2.73990	1.49299	1.41700	1.16500	1.44400	4.43442	-0.91988	0.68501
H	-3.87178	-0.02976	1.28010	2.61500	-0.08700	0.95200	4.26806	-0.57440	-1.05033
H	1.01652	-0.56847	-1.32327	1.55800	3.24200	-1.63300	1.97375	1.71495	1.83796
H	0.74338	-2.29358	-1.55985	2.38500	3.49800	-0.07500	3.73022	1.93993	1.70154
H	1.49541	-1.74829	-0.07455	0.71900	2.94400	-0.06500	3.07761	0.44164	2.39784
Li	1.63948	2.78082	1.19571	-0.85700	0.38700	1.92900	-3.64786	-1.11977	1.26962
F	0.35447	-0.16891	1.57345	0.90900	0.52200	-0.01600	-4.77771	-0.15465	1.96346
C	3.46228	3.05198	0.37475	-1.74600	0.58300	-0.03000	-1.75376	2.64834	-0.90274
C	3.47601	2.48343	-1.04796	-2.24700	-0.14300	-1.27900	-0.87223	2.70516	-2.15257
C	4.54821	2.32489	1.17492	-2.58400	0.21700	1.19900	-3.02822	3.47830	-1.05972
C	3.85607	4.52966	0.28782	-1.70800	2.09800	-0.23100	-0.98192	3.08856	0.34546
H	3.23691	1.40789	-1.07404	-2.27300	-1.23000	-1.12300	-1.42359	2.38285	-3.04405
H	4.47286	2.59129	-1.53236	-1.59600	0.05900	-2.13700	0.00960	2.06116	-2.04741
H	2.75643	2.99190	-1.70928	-3.26300	0.18000	-1.53800	-0.52096	3.73054	-2.33067
H	5.55466	2.43669	0.71037	-2.62500	-0.87300	1.35200	-3.60182	3.16363	-1.94000
H	4.63612	2.70935	2.20263	-2.18600	0.69700	2.10200	-3.66004	3.36653	-0.17242

H	4.36165	1.24191	1.25040	-3.62200	0.55200	1.07800	-2.78152	4.54161	-1.18210
H	3.14523	5.11635	-0.31399	-2.71800	2.49500	-0.40200	-0.60340	4.11177	0.21632
H	4.85566	4.66894	-0.18361	-1.08800	2.36000	-1.09500	-0.11898	2.43673	0.53239
H	3.91151	5.00527	1.27868	-1.28700	2.59100	0.65200	-1.63630	3.07842	1.22589

Table 12. The H-C-C-F torsion angles and corresponding J_{HF} coupling constants for compounds **9a** and **11a**.

Compound	Newman projection	ϕ_{H1F}	J_{H1F}	ϕ_{H2F}	J_{H2F}
**9a**	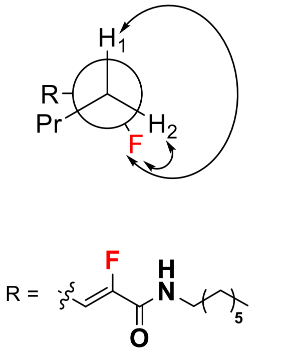	144.5°	26.8 Hz	28.9°	-[a]
**11a**	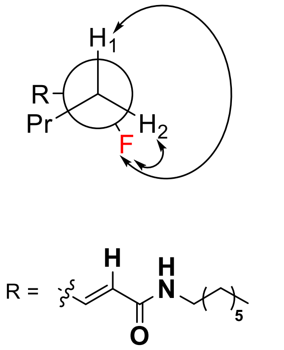	168.0°	26.1 Hz	52°	-[a]

[a] Not observed in ^{1}H and ^{19}F NMR spectra.

4. References

(1) Bilska-Markowska, M.; Patyk-Kaźmierczak, E.; Lusina, A., Synthesis of Fluorinated Amides Starting from Carbohydrates Based on the Claisen Rearrangement. *Eur. J. Org. Chem.* **2022,** *2022*, e202101378.

(2) Chai, J.-D.; Head-Gordon, M. Long-Range Corrected Hybrid Density Functionals with Damped Atom–Atom Dispersion Corrections. *Phys. Chem. Chem. Phys.* **2008**, *10* (44), 6615–6620. https://doi.org/10.1039/B810189B.

(3) Petersson, G. A.; Bennett, A.; Tensfeldt, T. G.; Al-Laham, M. A.; Shirley, W. A.; Mantzaris, J. A Complete Basis Set Model Chemistry. I. The Total Energies of Closed-shell Atoms and Hydrides of the First-row Elements. *J. Chem. Phys.* **1988**, *89* (4), 2193–2218. https://doi.org/10.1063/1.455064.

(4) Bregier-Jarzębowska, R.; Malczewska-Jaskóła, K.; Jankowski, W.; Jasiewicz, B.; Hoffmann, M.; Gąsowska, A.; Jastrząb, R. Experimental and Quantum-Chemical Studies of Anabasine Complexes with Copper(II) and Zinc(II) Ions. *Polyhedron* **2015**, *85*, 841–848. https://doi.org/10.1016/j.poly.2014.10.008.

(5) Malczewska-Jaskóła, K.; Jankowski, W.; Warżajtis, B.; Jasiewicz, B.; Hoffmann, M.; Rychlewska, U. Chalcogenated (S)-(−)-Nicotine Derivatives as Chiral Linkers for 1D Coordination Polymers. *Polyhedron* **2015**, *100*, 404–411. https://doi.org/10.1016/j.poly.2015.08.027.

(6) Jankowski, W.; Kurek, J.; Barczyński, P.; Hoffmann, M. Quantum-Chemical, NMR, FT IR, and ESI MS Studies of Complexes of Colchicine with Zn(II). *J Mol Model* **2017**, *23* (4), 127. https://doi.org/10.1007/s00894-017-3306-z.

(7) Musumeci, C.; Wałęsa-Chorab, M.; Gorczyński, A.; Markiewicz, G.; Bogucki, A.; Świetlik, R.; Hnatejko, Z.; Jankowski, W.; Hoffmann, M.; Orgiu, E.; Stefankiewicz, A. R.; Patroniak, V.; Ciesielski, A.; Samorì, P. Generation of Low-Dimensional Architectures through the Self-Assembly of Pyromellitic Diimide Derivatives. *ACS Omega* **2017**, *2* (4), 1672–1678. https://doi.org/10.1021/acsomega.7b00286.

(8) Jankowski, W.; Długosz, R.; Pruski, B.; Koroniak, H.; Hoffmann, M. On the Stabilization of Organic Peroxides by Plasticizers: Quantum Chemical Study on Interactions of 2,2-Dihydroperoxybutane with Dimethyl Phthalate. *Journal of Molecular Structure* **2022**, *1261*. https://doi.org/10.1016/j.molstruc.2022.132864.

(9) Peng, C.; Bernhard Schlegel, H. Combining Synchronous Transit and Quasi-Newton Methods to Find Transition States. *Israel Journal of Chemistry* **1993**, *33* (4), 449–454. https://doi.org/10.1002/ijch.199300051.

(10) Fukui, K. The Path of Chemical Reactions - the IRC Approach. *Acc. Chem. Res.* **1981**, *14* (12), 363–368. https://doi.org/10.1021/ar00072a001.

(11) Tomasi, J.; Mennucci, B.; Cammi, R. Quantum Mechanical Continuum Solvation Models. *Chem. Rev.* **2005**, *105* (8), 2999–3094. https://doi.org/10.1021/cr9904009.

(12) Frisch, M. J.; Trucks, G. W.; Schlegel, H. B.; Scuseria, G. E.; Robb, M. A.; Cheeseman, J. R.; Scalmani, G.; Barone, V.; Petersson, G. A.; Nakatsuji, H.; Li, X.; Caricato, M.; Marenich, A. V.; Bloino, J.; Janesko, B. G.; Gomperts, R.; Mennucci, B.;

Hratchian, H. P.; Ortiz, J. V.; Izmaylov, A. F.; Sonnenberg, J. L.; Williams-Young, D.; Ding, F.; Lipparini, F.; Egidi, F.; Goings, J.; Peng, B.; Petrone, A.; Henderson, T.; Ranasinghe, D.; Zakrzewski, V. G.; Gao, J.; Rega, N.; Zheng, G.; Liang, W.; Hada, M.; Ehara, M.; Toyota, K.; Fukuda, R.; Hasegawa, J.; Ishida, M.; Nakajima, T.; Honda, Y.; Kitao, O.; Nakai, H.; Vreven, T.; Throssell, K.; Montgomery, J. A., Jr.; Peralta, J. E.; Ogliaro, F.; Bearpark, M. J.; Heyd, J. J.; Brothers, E. N.; Kudin, K. N.; Staroverov, V. N.; Keith, T. A.; Kobayashi, R.; Normand, J.; Raghavachari, K.; Rendell, A. P.; Burant, J. C.; Iyengar, S. S.; Tomasi, J.; Cossi, M.; Millam, J. M.; Klene, M.; Adamo, C.; Cammi, R.; Ochterski, J. W.; Martin, R. L.; Morokuma, K.; Farkas, O.; Foresman, J. B.; Fox, D. J. Gaussian˜16 Revision C.01, 2016.
